# Combining signal and sequence to detect RNA polymerase initiation in ATAC-seq data

**DOI:** 10.1371/journal.pone.0232332

**Published:** 2020-04-30

**Authors:** Ignacio J. Tripodi, Murad Chowdhury, Margaret Gruca, Robin D. Dowell

**Affiliations:** 1 Computer Science, University of Colorado, Boulder, Colorado, United States of America; 2 BioFrontiers Institute, University of Colorado, Boulder, Colorado, United States of America; 3 Molecular, Cellular and Developmental Biology, University of Colorado, Boulder, Colorado, United States of America; University of Cyprus, CYPRUS

## Abstract

The assay for transposase-accessible chromatin followed by sequencing (ATAC-seq) is an inexpensive protocol for measuring open chromatin regions. ATAC-seq is also relatively simple and requires fewer cells than many other high-throughput sequencing protocols. Therefore, it is tractable in numerous settings where other high throughput assays are challenging to impossible. Hence it is important to understand the limits of what can be inferred from ATAC-seq data. In this work, we leverage ATAC-seq to predict the presence of nascent transcription. Nascent transcription assays are the current gold standard for identifying regions of active transcription, including markers for functional transcription factor (TF) binding. We combine mapped short reads from ATAC-seq with the underlying peak sequence, to determine regions of active transcription genome-wide. We show that a hybrid signal/sequence representation classified using recurrent neural networks (RNNs) can identify these regions across different cell types.

## Introduction

Transcription is a critical first step in transmitting the information in the DNA into usable material. Transcription occurs at specific times and locations controlling both cell type and cellular response to almost all perturbations. A large fraction of the genome (50-70%) is transcribed in a cell [1], but only a small fraction of this transcription can be readily detected by steady state assays such as RNA-seq and microarrays. Transcribed units, regardless of they are stable or not, offer critical information about cellular state [2, 3]. Nascent transcription assays [4, 5], by virtue of directly measuring transcription, can detect immediate changes (times as short as 10 minutes) in response to perturbations [6]. Consequently, nascent transcription is a rich source of information on both regulation and cell state.

However, nascent transcription experiments such as global run-on sequencing (GRO-seq) and precision run-on sequencing (PRO-seq) are quite laborious, expensive, and require a large number of cells. In contrast, the assay for transposase-accessible chromatin, followed by high-throughput sequencing (ATAC-seq) has rapidly gained popularity since its inception, due to its ease of execution, small cell count requirements, and short time expenditure. Yet, ATAC-seq measures chromatin accessibility, not RNA polymerase activity. Most sites of RNA polymerase activity co-occur with open chromatin regions (OCRs) detectable by ATAC-seq [6–8]. Unfortunately, only a fraction of open chromatin regions harbor RNA polymerase activity [9]. Reasoning that the presence of RNA polymerase may itself alter chromatin state in some subtle fashion, we wondered whether signal exists within ATAC-seq, which could be utilized to discriminate peaks that overlap RNA polymerase activity from other open chromatin regions unrelated to active transcription.

Machine learning is a natural tool to classify data derived from genomics assays, particularly ATAC-seq. A wide range of machine learning applications for ATAC-seq datasets have been developed, from classifying types of chronic lymphocytic leukemia cells [10], to TF motif discovery [11], discriminating among brain cell types [12], and identifying gene enhancer regions using ATAC-seq peaks [13]. Given regions of polymerase initiation are dense with transcription factor binding motifs and have a characteristics sequence bias [6], we reasoned that any predictor would benefit from leveraging sequence information. Likewise, RNA polymerase may induce particular signatures within ATAC-seq peaks. Therefore, we approach the problem of classifying ATAC-seq peaks as a signal processing task, where we employ both sequence and ATAC signal features in our data representation scheme. In this work, we utilize this hybrid encoding to examine the ability of ATAC-seq data to identify sites of overlapping nascent transcription.

## Materials and methods

### Datasets

We utilized a collection of quality-assessed samples, or short-read runs (SRRs) originating from different human cell lines and labs. We obtained SRRs from lung adenocarcinoma (A549), myeloid B-cells (GM12878), human embryonic stem cells (H1), colon carcinoma (HCT116), leukemia lymphoblasts (K562), prostate carcinoma (LNCaP), invasive ductal carcinoma (MCF7), and childhood acute monocytic leukemia cells derived from peripheral blood (THP1). All SRRs were retrieved from the Gene Expression Omnibus (GEO [14]), and are listed with accession and quality evaluation details on S1 Table. for ATAC-seq, and S2 Table. for GRO-seq/PRO-seq. For each SRR evaluated, we used a minimum depth cutoff of 12 million reads post-trimming and mapping, greater than 10% genomic base-pair coverage for ATAC-seq samples, and a minimum of a predicted 5 million unique reads per 50 million sequenced for nascent samples (determined using preseq [15]). Samples were further evaluated using other metrics including read duplication, read distributions, and GC content using both the RSeQC [16] and FastQC tools.

### Data processing

Both ATAC-seq SRRs and nascent transcription (GRO/PRO-seq) SRRs were processed using Nextflow-based [17] pipelines [18, 19]. A full pipeline report of the run, workflow diagram, and quality control report generated by MultiQC (v. 1.7) [20], including trimming (BBDuk, BBMap Suite), mapping (HISAT2), read distribution (RseQC), coverage (pileup, BBMap Suite), G/C content (Picard Tools [21]), and complexity metrics (preseq), are included in the S1 File. Additional QC metrics for ATAC-seq SRRs were assessed using ATACseqQC [22] and its output is also included in S2 File. SRRs were de-duplicated using Picard Tools prior to peak call-ing. Peak calls were generated using MACS2 narrowPeak using the q-value default (< 0.05). Blacklisted regions (those having artificially high signal and read mapping, obtained from http://mitra.stanford.edu/kundaje/akundaje/release/blacklists/hg38-human/) were removed using BEDTools intersect [23]. Training files (required for FStitch [7]) used in nascent data processing for each cell type and output from application of both FStitch and Tfit [24] (using default pipeline settings), are included in the S3 File. Some SRRs were discarded due to low complexity using the aforementioned criteria which strongly affects both FStitch and Tfit in modeling regions of active transcription (see MultiQC reports in S1 File). Genome browser track figures were generated using DeepTools [25] pyGenomeTracks.

All SRRs and sequences were analyzed with respect to the GRCh38 human reference genome. The ATAC-seq peaks for all SRRs from the same cell type (generally replicates) were combined into a single cell-type-specific data file, which was subsequently used for training and testing. These peaks were combined by taking the union of all peak regions across SRRs from the same cell type (directly overlapping in genomic coordinates), and averaging the number of mapped ATAC-seq reads (previously normalized by millions mapped) at each nucleotide. Individual peaks within these files are referred to as OCRs.

Similarly, the coverage files from Nascent-Flow (in bedGraph format) were combined into a single per-cell-type data file. We leveraged the combined output of two tools to detect nascent transciption, FStitch [7] and Tfit [24]. FStitch identifies all transcribed regions within a nascent transcription experiment, but cannot necessarily distinguish individual transcripts in densely transcribed regions. Tfit identifies individual transcripts based on the expected behavior of RNA polymerase II. If either of the tools detected a region of active transcription (FStitch) or bidirectional transcription indicative of functional transcription factor binding (Tfit) in a region that overlapped with an OCR, the OCR was labeled as “positive”. Otherwise the OCR is labeled “negative”. This resulted in approximately 29% of all OCRs labeled as positive. It’s worth pointing out that, while nascent transcription is commonly seen at transcription start sites (TSSs) for active genes, most transcription (estimated at 72% [24]) actually occurs at other loci throughout the genome, for example due to binding of regulatory proteins (S1 Fig).

### Data encoding

**Fig 1:**
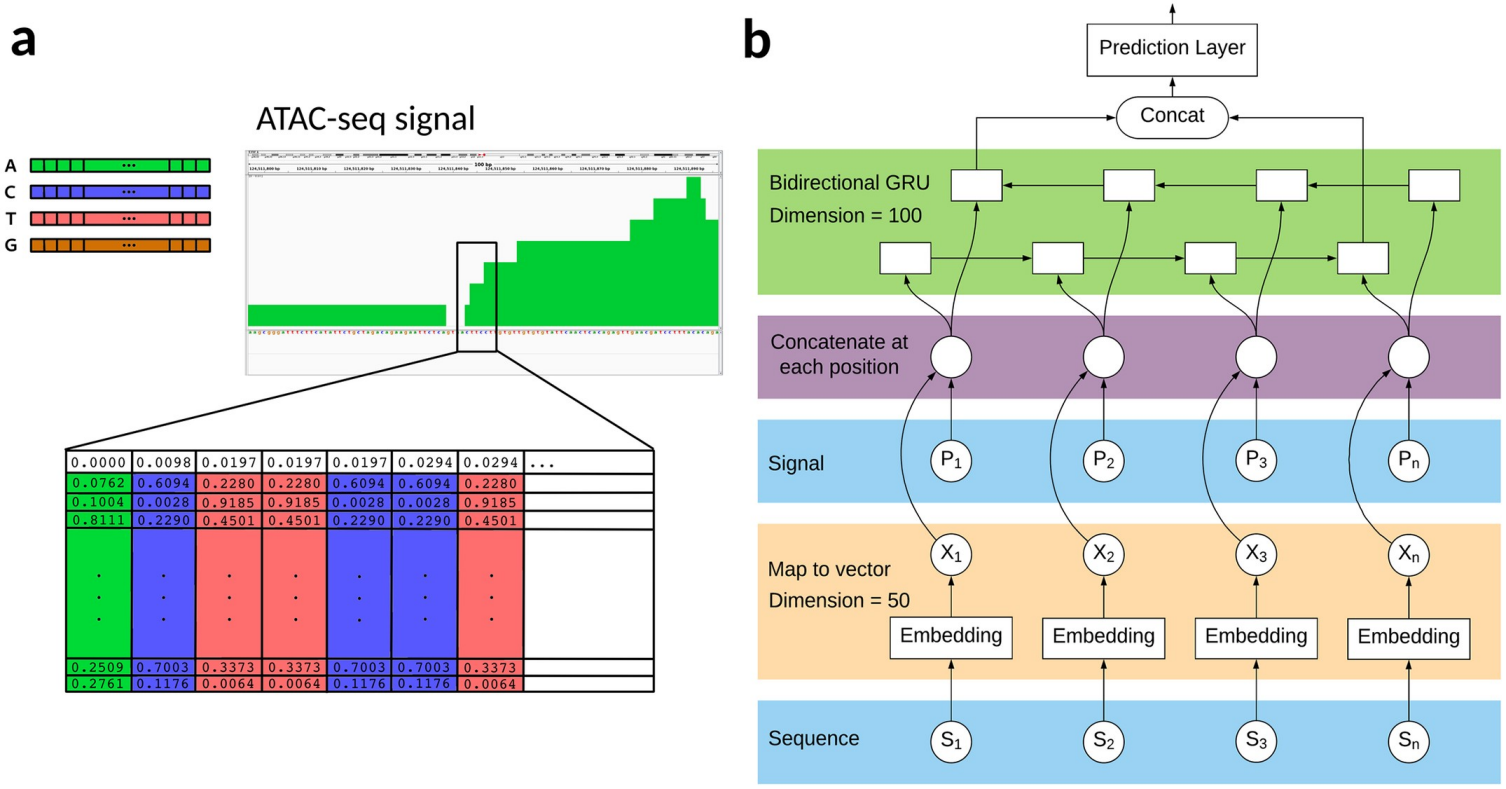
Hybrid encoding and RNN model architecture. (**a**) A vector embedding was trained for each nucleotide (top left, also including other base symbols following the IUPAC convention). For our signal/sequence hybrid model, we generated a 50-dimension training vector for each peak by combining nucleotide information (a vector embedding based on neighboring nucleotides) and the normalized number of ATAC-seq reads mapped for that nucleotide (by millions mapped). In this example, we show how a small portion of an OCR detected with ATAC-seq (top right, green) with the sequence ACTTCCT would be represented in two dimensions (bottom, one nucleotide per column), with the first row reflecting the normalized read coverage for each of those nucleotides and the rest of each column consisting of the nucleotide’s dense vector representation. (**b**) Nucleotides in the 1kbp evaluation window are extracted from the reference genome (bottom blue layer) were passed to an embedding layer (orange) to generate a dense vector representation from each. The peak signal level associated to each nucleotide (middle blue layer; i.e., the number of mapped ATAC-seq reads normalized by millions mapped) is then combined with the nucleotide embedding vector (purple layer, vector representation shown in panel **a**). Each vector is passed to a gated recurrent unit in each direction (green layer) to capture the long- and short-term relations between nucleotides, and the outputs from the last forward and reverse gates are concatenated to be used or the final prediction.

We developed a hybrid encoding of sequence and signal that summarizes each OCR into a 1kbp dense vector encoding (Fig 1a). The window size of 1kbp was chosen to account for most OCR sizes, and include flanking regions in the analysis. Signal is captured at nucleotide resolution by the number of mapped ATAC-seq reads, normalized by millions mapped. Sequence-derived features are encoded using the Hill et. al. approach, which maps an input sequence of nucleotides to a sequence of vectors using an embedding layer [26]. This embedding layer consists of a dense vector representation of each nucleotide, trained on the sequences corresponding to every peak in an ATAC-seq SRR. The 2-dimensional input feature matrix is the result of stacking the normalized number of mapped ATAC-seq reads (the OCR’s “signal”) with the vector embedding of each nucleotide (Fig 1a), both in the same 1kbp peak evaluation window. This hybrid encoding representation could alternatively be considered as a way to weight each nucleotide by its level of accessibility.

The choice of a hybrid encoding scheme for each OCR fixed window resulted from a previous study [27], where we evaluated the performance of many different data encoding schemes and machine learning classifiers. OCRs were evaluated using only the signal at each nucleotide, only the underlying sequence, or a combination of both. The hybrid signal/sequence representation, in combination with a recurrent neural network model, yielded the best performance in detecting both underlying RNA polymerase activity and histone marks associated with transcriptional activity at each OCR tested. This manuscript focuses only on the underlying RNA polymerase activity for each region of accessible chromatin.

### Classifiers

We developed a recurrent neural network (RNN) model to classify ATAC-seq peaks represented by our hybrid encoding, utilizing the Keras framework. Given the sequential nature of our data, an RNN presented the most suitable choice. Since both the ATAC-seq signal or the underlying nucleotide sequence may be read in either direction (sense or anti-sense), we implemented this classifier with bidirectional gated recurrent units (GRUs). We combined our sequence embedding and signal into a single vector representation (Fig 1a) that is utilized as input to the GRUs (Fig 1b). A learning rate of 0.0001, a dropout date of 0.1, an embedding layer size of 50 and a hidden layer size of 100 were selected after hyperparameter optimization, from a grid of embedding dimensions [15, 50, 100], dropout rates [0.1, 0.2, 0.3], learning rates [0.001, 0.0005, 0.0001] and hidden sizes [100, 200, 350, 500]. All instances of the RNN models were executed with the aid of a GPU for increased computational performance. Specifically, the Tesla K80 GPU on Elastic Cloud Computing from Amazon Web Services.

In a previous study [27] we evaluated a variety of classifiers and encodings for our RNN and found that the RNN outperformed all other methods at predicting histone marks associated with OCRs that are related to active transcription. For completeness, we briefly summarize the earlier study. We examined a variety of simpler machine learning classifiers such as random forests, support vector machines, and ADABoost, as well as traditional signal processing approaches to detect signatures in ATAC-seq signal in each 1kbp evaluation window. In addition we considered an RNN model with only signal-derived features, only sequence-derived features, and a combination of both. Different encodings of both the signal (autoencoder, first-level wavelet decomposition) and sequence (wavelet decomposition of electron-ion interaction potential) were considered. Overall, the hybrid encoding of both sequence and signal to the RNN model performed the best.

### Model evaluation

We sought out a binary classification for every OCR in the test set, where a positive label (value of 1) represented nascent transcription at said OCR, and a negative label (value of 0) denoted no nascent transcription. We used the area under the curve (AUC) from receiver operating characteristics (ROC) curves to compare the performance of this binary classification task, as well as weighted F1-scores to evaluate the balance of predictive precision and recall.

Each test was performed using a “leave-one-out” training (LOOT) strategy in a very conservative performance evaluation fashion, in order to remove cell type-specific characteristics from training, as well as ensuring that the genomic coordinates tested do not exist within the training set. This is important because, for example, 3,833 OCRs are common (that is, they overlap in coordinates) to all data sets. We used only OCRs from specific chromosomes during training (chr1 thru chr11) and OCRs from the remaining chromosomes for the test set (chr12 through chr22, chrX and chrY), from each specific cell type (see S2 Fig for an example). Since chromosomes are numbered from largest to smallest, this split provided a reasonable number of training and test OCRs for each cell type-specific classification run.

We segregated a portion of our data for validation purposes only, to be used while training the RNN. This consisted of all OCRs from the HCT116 datasets in chromosomes chr1 thru chr11. These OCRs were therefore excluded from any training or test set, and prevented further bias during the model training step. The remaining OCRs from HCT116 cells were discarded.

We sought to determine if we could classify ATAC-seq peaks based on their co-occurrence with transcription, as measured in cell type matched nascent transcription assays. To this end, we identified high quality datasets within public repositories where both ATAC-seq and nascent transcription data (either GRO-seq or PRO-seq) were available for the same cell type and condition (in every case, these were labeled as “untreated” or “DMSO” in the SRR metadata). Under this criteria of matching assays and conditions, we obtained 9 sets (each a distinct cell type) of matched data. The depth of the obtained datasets varied, between 11.1 and 192.9 million reads for ATAC-seq SRRs, and between 14.8 and 213.4 million for nascent transcription SRRs. Across the 9 distinct cell types, nearly half a million open chromatin regions were identified. All OCRs were labeled as transcribed (positive) or negative, based on the output from the Tfit and FStitch tools on the same region (as described in data processing).

**Fig 2:**
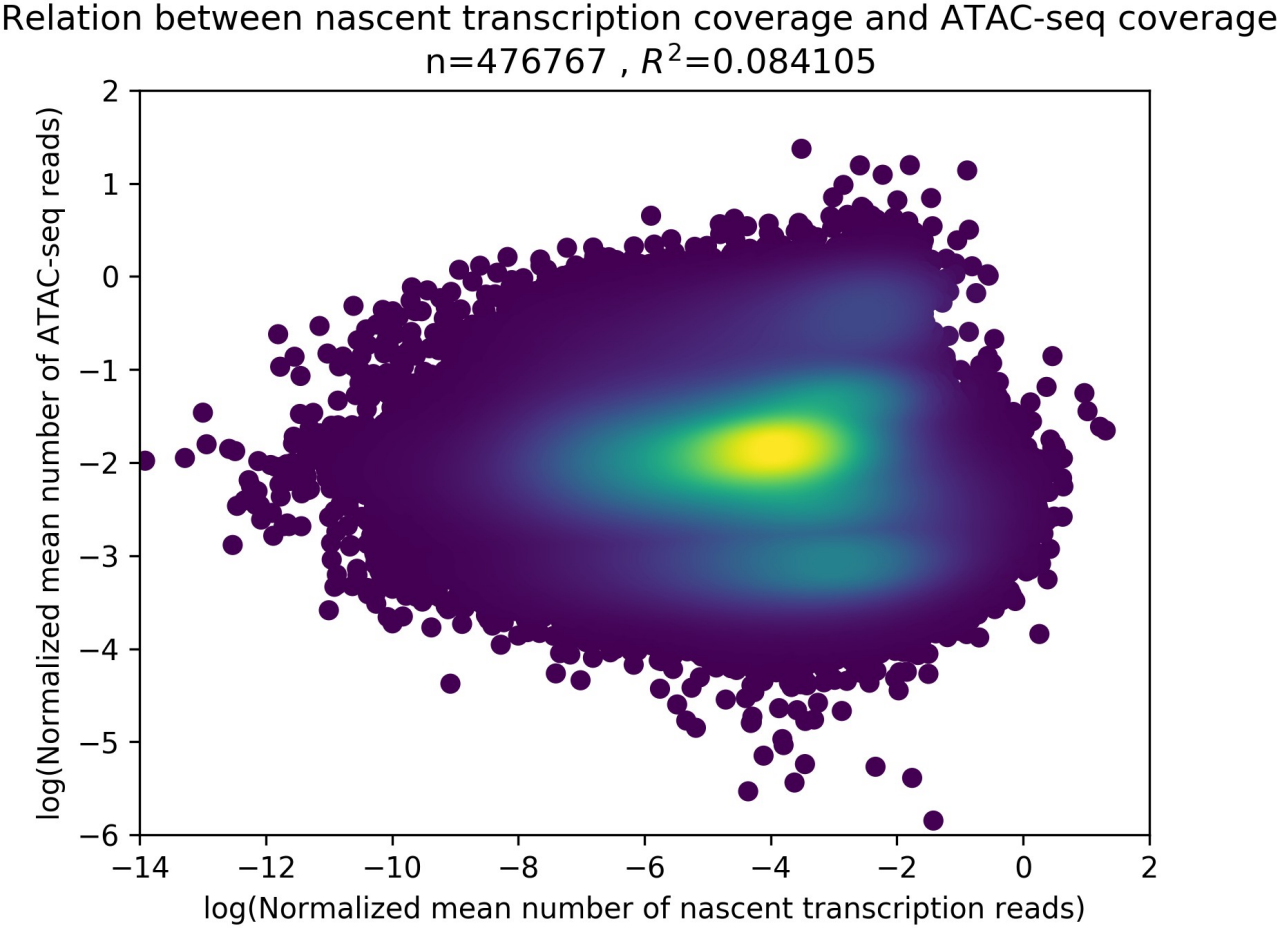
Accessibility vs. transcription. Each point in this scatter plot is an ATAC-seq peak, where we compare the mean number of mapped ATAC-seq reads in its 1kbp evaluation window (y-axis) to the mean number of mapped nascent transcription reads on that same window (x-axis). There is essentially no correlation (*r*^2^ = 0.084) between the two, making this average peak metric not sufficient to predict active transcription.

We first asked whether there was a relationship between accessibility, as measured in ATAC-seq, and nascent transcription. While there is a very loose positive correlation (*r*^2^ = 0.084) between read coverage in both ATAC-seq (accessibility) and nascent transcription (Fig 2), but the correspondence was far from diagnostic. Therefore, we turned to machine learning as a means of classifying open chromatin regions as to whether they harbor transcription.

We developed our RNN approach using a hybrid data representation that captures both sequence and signal features within ATAC-seq data. We reasoned that sequence features are likely to be critical to transcription initiation, as transcription factors recognize primary sequence and regulatory regions (enhancers and promoters) have a known positional sequence bias [6]. However, ATAC-seq signal (read depth) is also informative as there is a weak correlation between accessibility and transcription (Fig 2, S3 Fig for specific cell types) and the presence of RNA polymerase II may leave distinct signatures within the accessibility profiles.

We first sought to determine a baseline performance for this classification task. To this end, we used a naive approach that looked at the distribution of mean ATAC-seq coverage per OCRs labeled as “positive” (overlapping bidirectional nascent transcription) or “negative”. We used a kernel density estimator with Gaussian kernels to define an empirical distribution of mapped ATAC-seq reads for each case, and based on an odds ratio we predicted whether an OCR overlapped transcription. This baseline classifier displayed a performance barely better than random calls, with an F1-score of 0.550 and an ROC AUC of 0.554 (S4 Fig) using a random 10% of all OCRs for testing.

**Fig 3:**
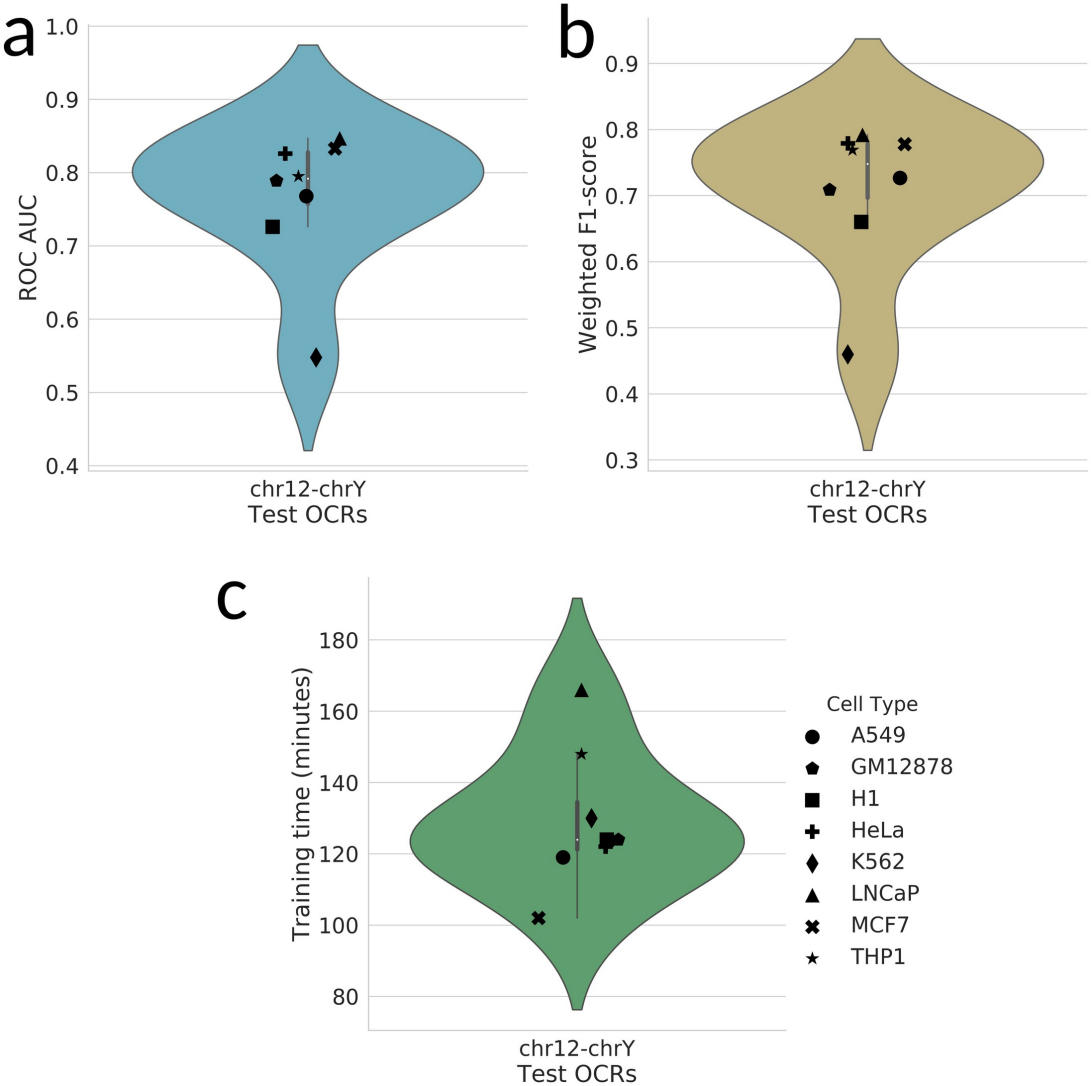
Classifier performance across cell types. **(a)** Receiver operating characteristic (ROC) area under the curve (AUC, light blue), **(b)** F1-score (tan), and **(a)** RNN training time (green) for LOOT-based performance evaluation. OCRs from each cell type tested are displayed using the same marker (see key).

**Fig 4:**
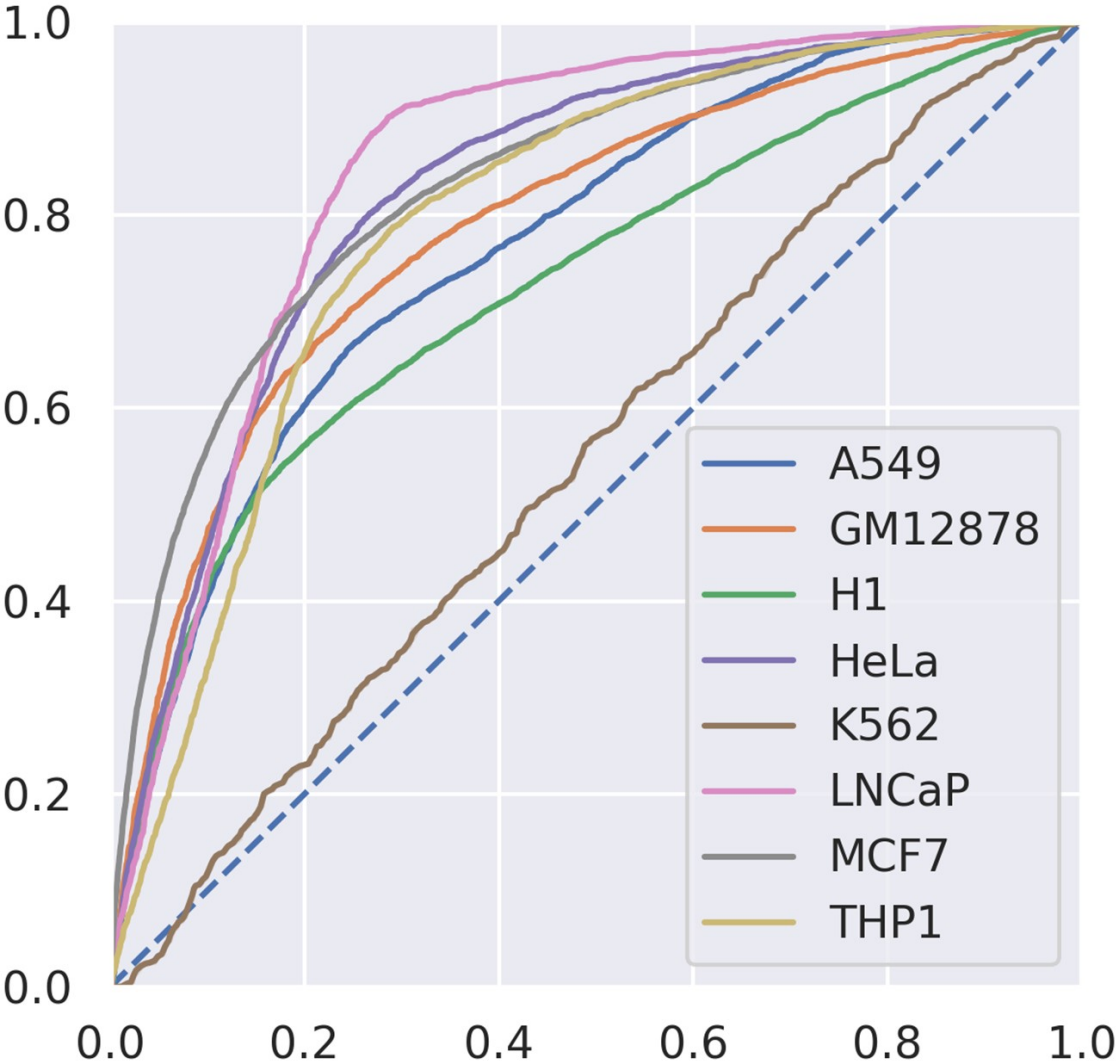
Cell type focused strategy results. ROC curves resulting from testing on the different OCRs corresponding to each cell type, in a leave-one-out fashion.

Our RNN model using the hybrid signal/sequence encoding greatly surpassed the baseline performance. The test OCRs from each cell type (chr12-chrY, as described earlier) were evaluated separately. The results of this LOOT strategy are shown in Figs 3 and 4. K562 cells generally presented much lower performance, which could be related to the quality and complexity of the dataset (see S1 File). The SRR quality was above our cutoffs, but still the lowest compared to the other datasets. The number of OCRs that could be detected was also significantly lower than SRRs from other cell types. Overall, however, the performance of the classifier with AUC values between 0.548 (K562, an outlier) and 0.847, and a median of 0.792, indicates that the classifier is recognizing general features of transcribed OCRs rather than specific features of the cell type/experiment or genomic region.

**Fig 5:**
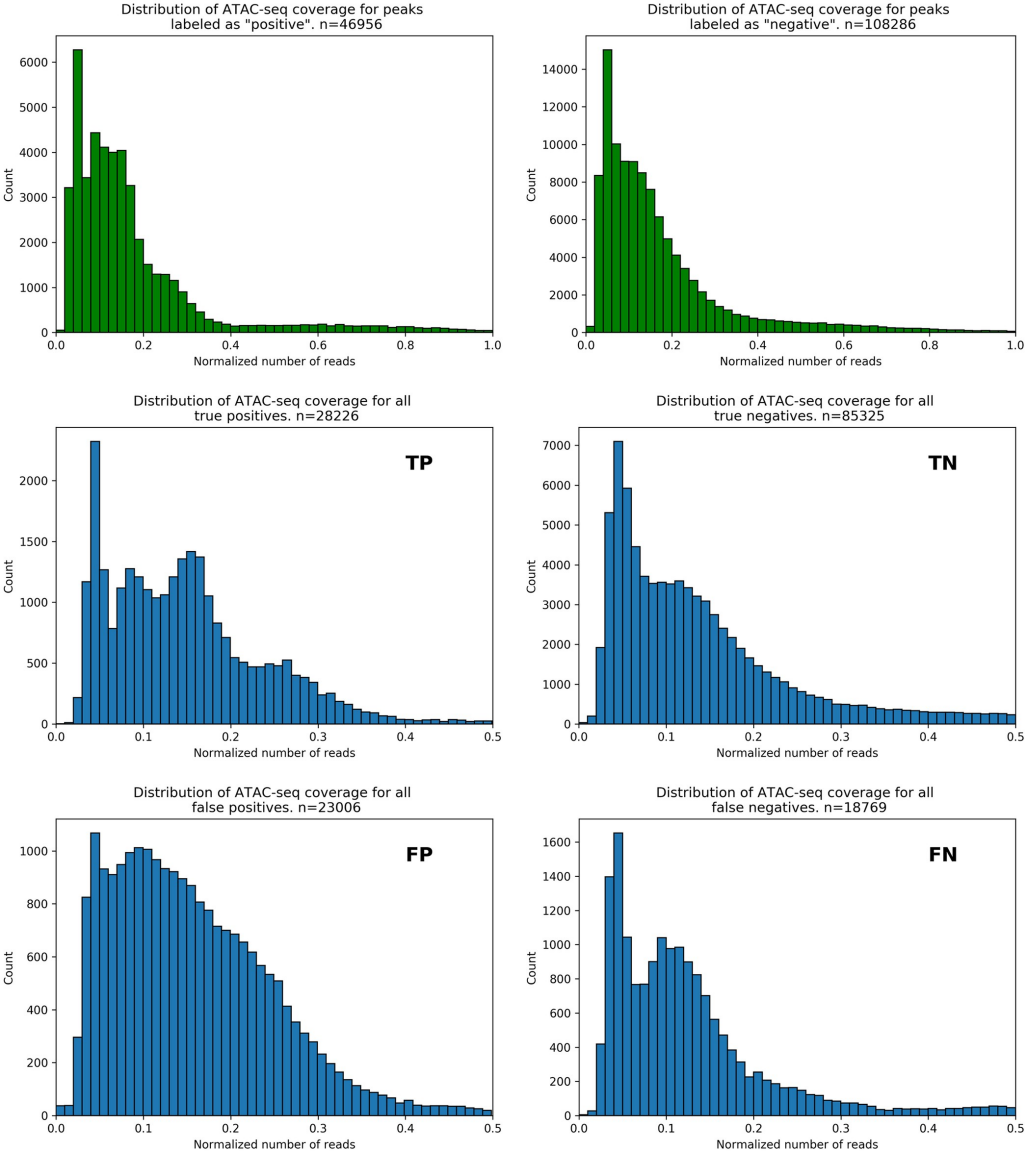
Distribution of ATAC-seq reads for classification results. Distribution of mapped reads from ATAC-seq SRRs, for OCRs corresponding to the training set (green histograms, top) and each classification metric (blue histograms, metric noted in upper right corner of each panel). Note the difference in y-axis scales among plots, as the size of each set differs.

**Fig 6:**
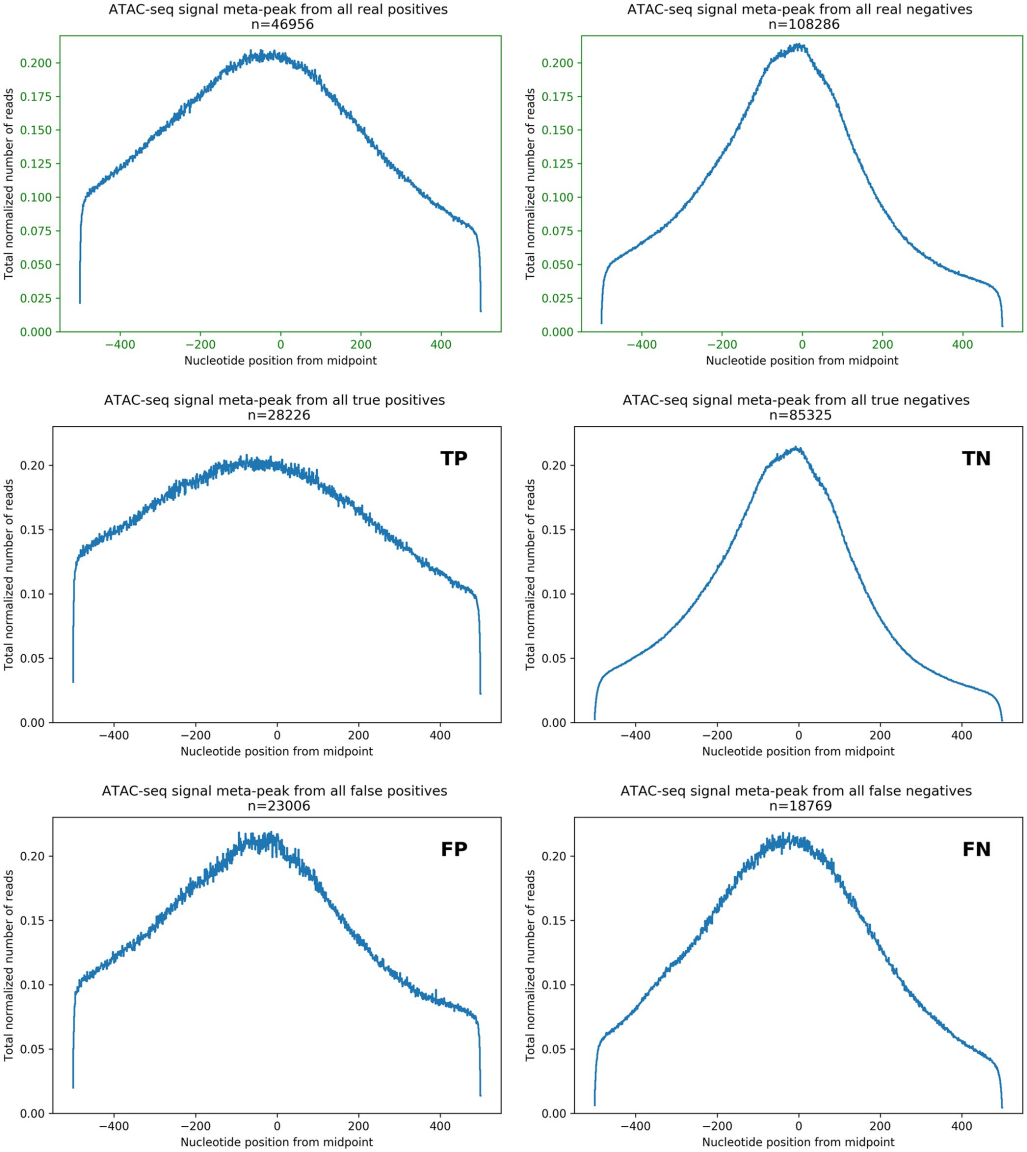
Meta-peaks from ATAC-seq signal at OCRs. Meta-peak plot generated by combining the ATAC-seq signal at each 1kbp evaluation window centered at OCRs for the entire training set (top row, green axis) and each classification metric: true positives (mid left), true negatives (mid right), false positives bottom left) and false negatives (bottom right). Note the difference in scales among plots, to emphasize the characteristic shape in each scenario.

Next we sought to understand the error characteristics of the classifier. To this end, we examined the ATAC-seq data profiles in the correctly classified and incorrectly classified peaks. The distribution of the mean number of ATAC-seq reads for each OCR is similar for true positives and negatives (Fig 5, top in green). Yet our classifier showed a tendency to mis-classify higher read depths as positive, exemplified by the slight shift in the false positive curve relative to the true positives. An examination of “meta-peak” signals (that is, the aggregated signal of all peaks involved in that subset) in each error class shows that negative-labeled OCRs have a generally narrower peak shape within the ATAC-seq data (Fig 6, green axis figures on the top row) compared to positive-labeled OCRs. Overall, OCRs overlapping nascent transcription appear to be significantly wider than those which don’t, which suggests a signature in the peak’s shape that is indicative of active transcription. The classifier clearly struggles with intermediate width cases, leading to errors (Fig 6, in blue). This would suggest that the local context, beyond each nucleotide point-wise information, is informative and may explain why the bidirectional GRU architecture was helpful to identify these regions.

**Fig 7:**
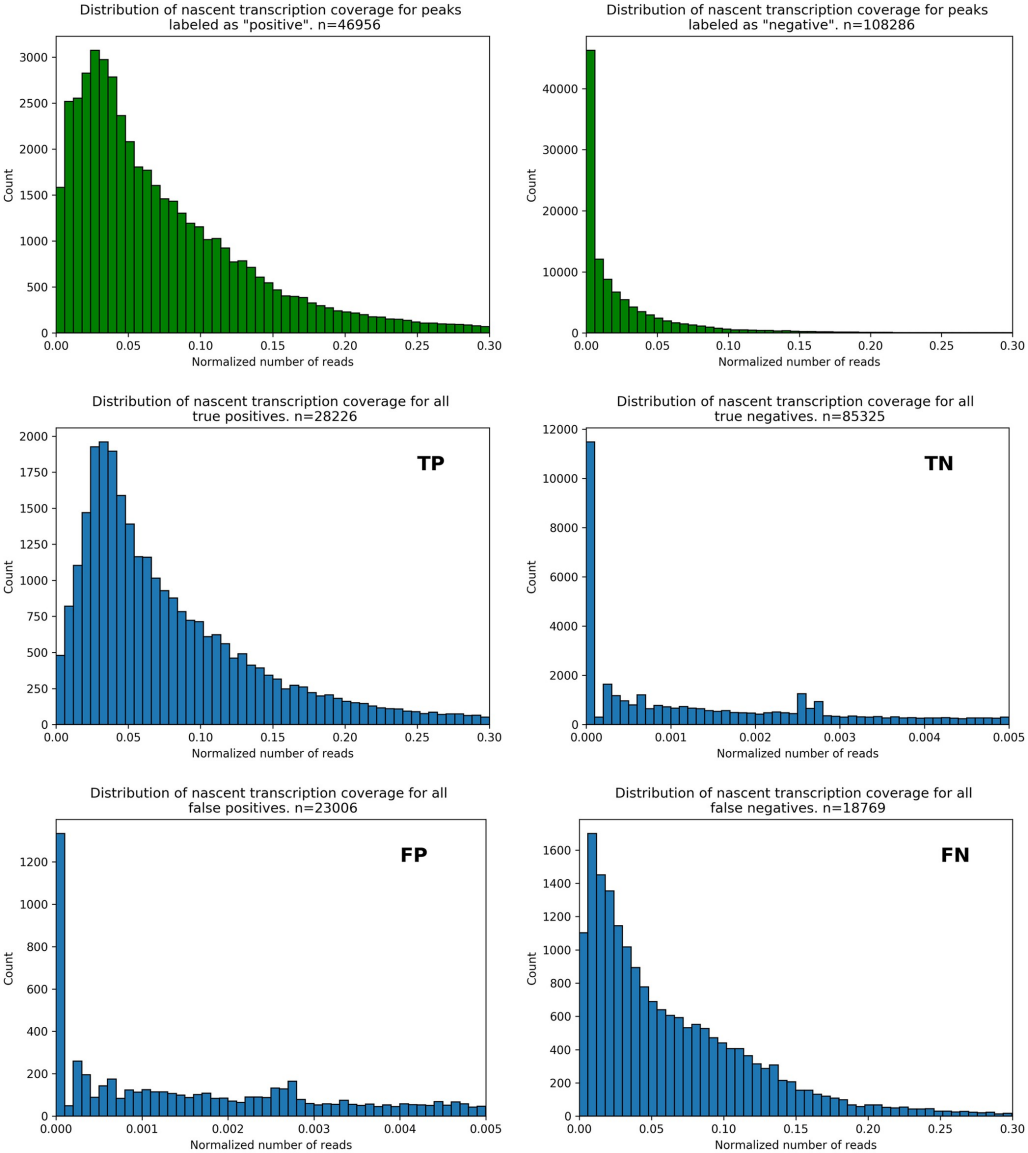
Distribution of nascent transcription reads for classification results. Distribution of mapped reads from nascent transcription SRRs, for OCRs corresponding to the training set (green histograms, top) and each classification metric (blue histograms, metric noted in upper right corner of each panel). Note the difference in scales among plots, to better appreciate the distribution of coverage in each scenario. The leftmost bin in the “positive”, “TP”, and “NF” panels correspond to very low levels of nascent transcription rather than no transcription, which are generally associated to regulatory regions.

**Fig 8:**
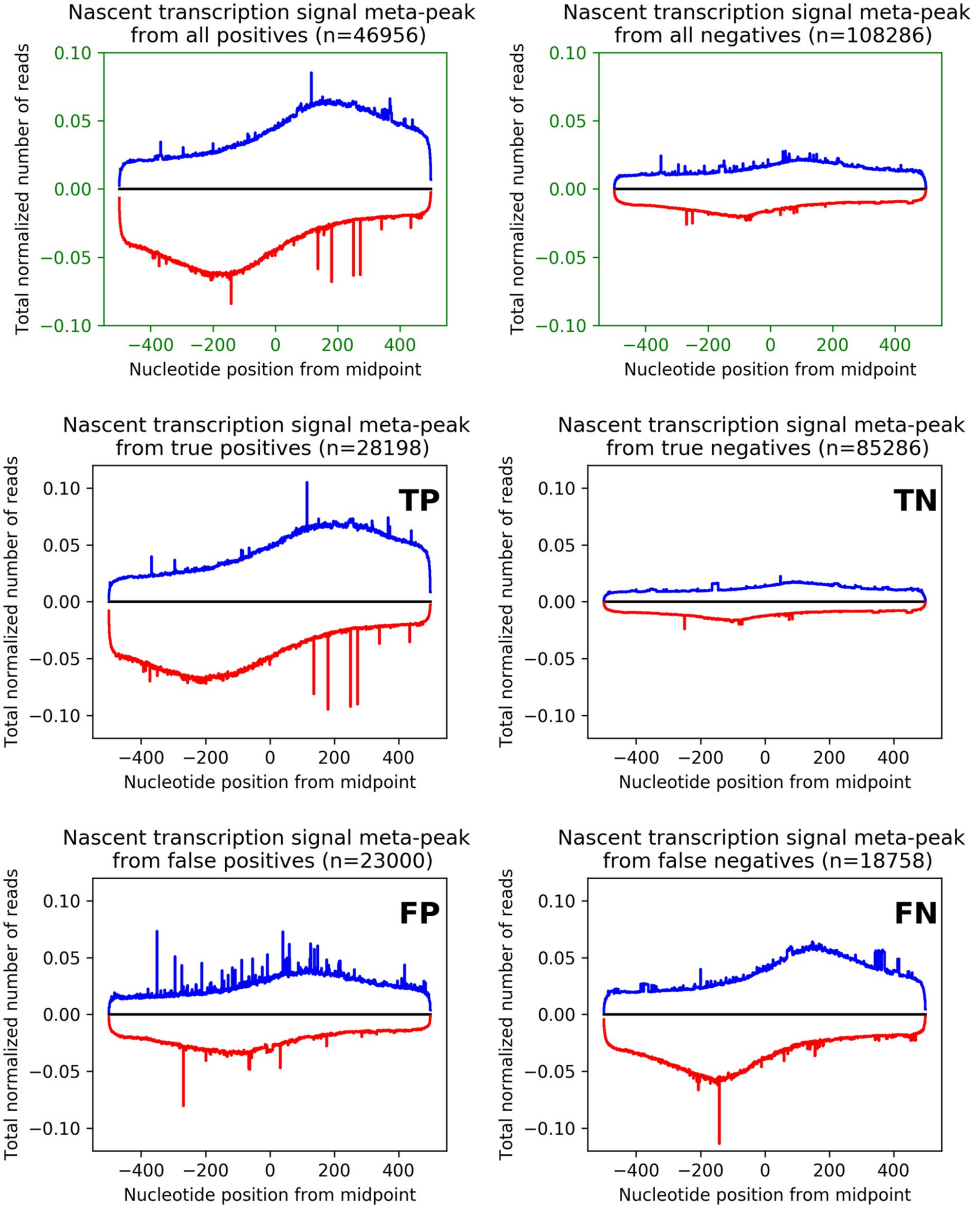
Meta-peaks from nascent transcription signal at OCRs. Meta-peak plot generated by combining the nascent transcription signal at each 1kbp evaluation window centered at OCRs for training data (top row, green axis) and each classification metric (middle and bottom). Signal is color coded by strand (blue is positive strand; red negative strand). Notice the differences in scale among plots, with TPs and FNs sharing the same scale, but distinct from TN and FP.

Because the general signal of the ATAC classifications well mimicked the training input (Fig 5), we next examined the distribution of mean nascent transcription across these same regions (Figs 7 and 8). As expected, positive-labeled OCR regions have generally higher levels of transcription than their negative-labeled counterparts (Fig 7, top in green). Importantly, not all negative-labeled regions have zero read coverage, as some noise is inherent in any sequencing protocol. Likewise, some positive-labeled regions do have very low read coverage because they may correspond to regulatory regions like enhancers, which typically are lowly transcribed. The classifier’s errors appear to be regions with low levels of transcription. An examination of the corresponding “meta-gene” signal for each category (Fig 8) further supports this conclusion. Intriguingly, the negative-labeled training data shows a slight, but noticeable bidirectional signal similar to the positive-labeled truth dataset. Given our reliance on two different nascent analysis tools (FStitch and Tfit), which each have their own bias and accuracy at different regions of the genome, it is possible that some of the OCRs characterized as false negatives may indeed be regions of active transcription. The meta-gene curve for false negatives reinforces this hypothesis, as there is a considerable level of bidirectional transcription at these regions. This suggests that the performance of our classifier is arguably a lower-bound, which could be improved with the availability of a “gold standard” transcription dataset.

**Fig 9:**
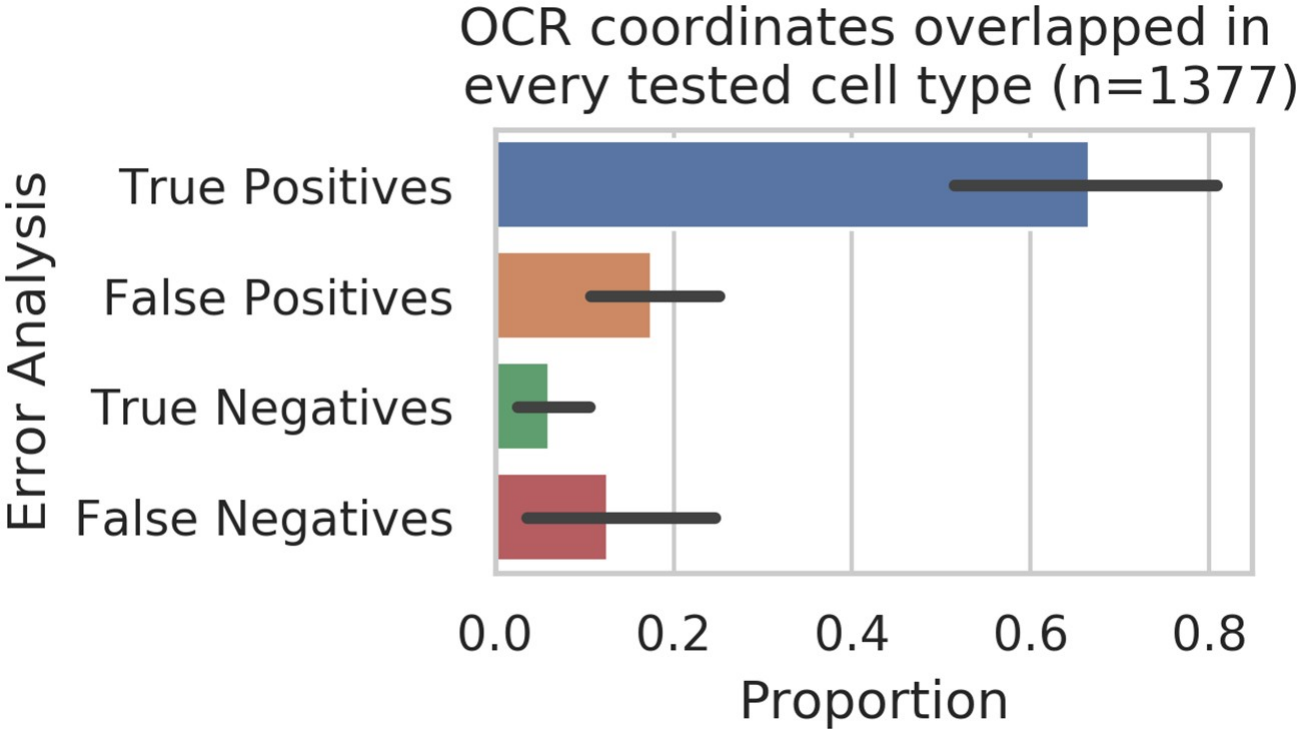
Commonly observed OCRs dominate performance. Proportion of OCRs common to every cell type (overlapping in genomic coordinates) categorized in the different performance metrics.

**Fig 10:**
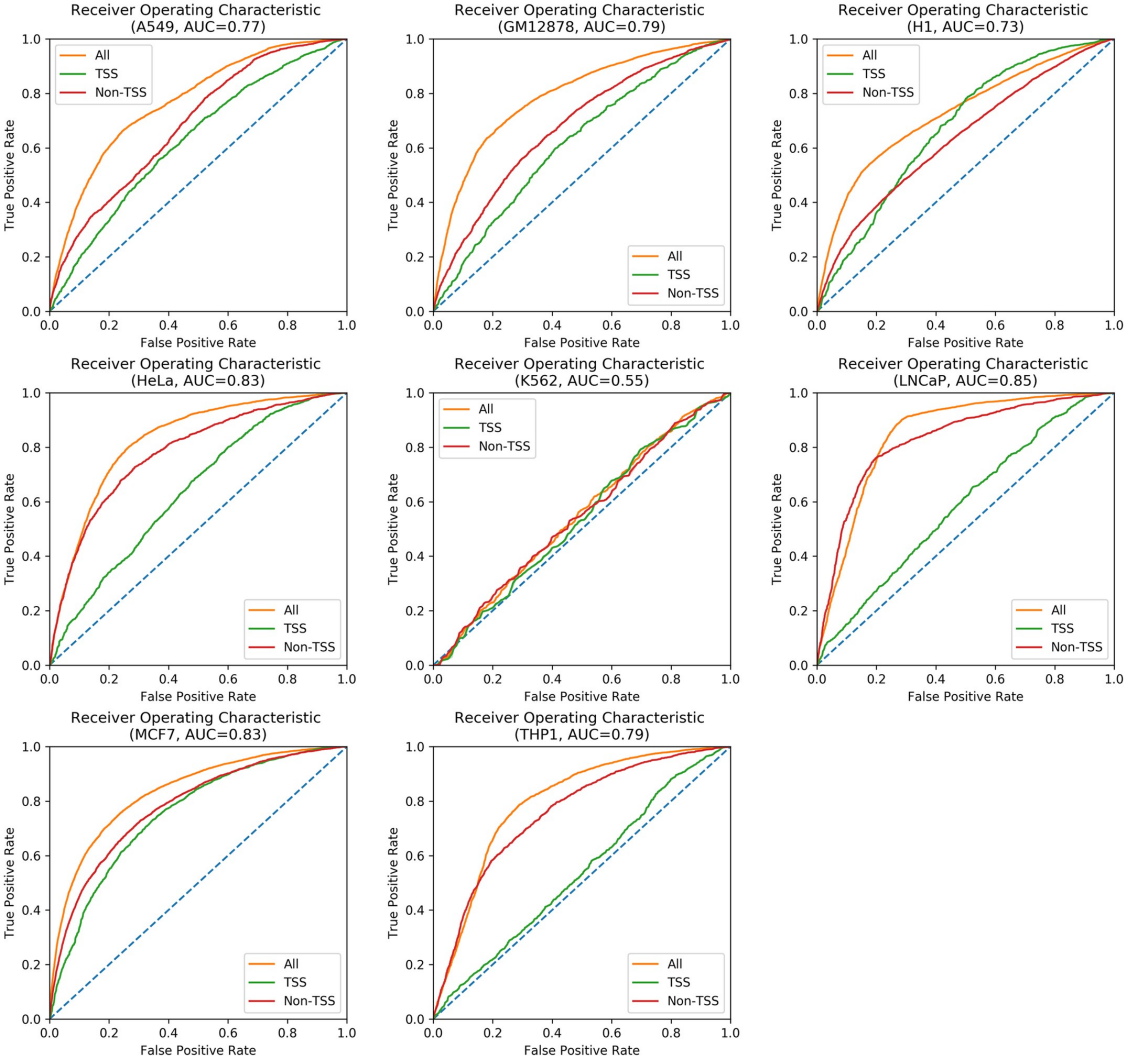
TSS regions are generally harder to classify than non-TSS ones (regulatory sites). ROC curves for OCRs overlapping TSSs (green) and non-TSS OCRs (red), for each test set. The orange curves correspond to all OCRs for that test set.

We next wanted to determine which classes of OCRs were driving performance. First we examined the common peaks (e.g. those present across all cell lines, at overlapping genomic coordinates). While common peaks are a minority (8,424/471,799 overall, and 1,377/155,242 across all test sets), we observed that these peaks are correctly classified in general (Fig 9). S6, S7 and S8 Figs provide classification statistics for OCRs unique to each cell type, shared among only two cell types, and among only three cell types, respectively. Next we examined OCRs based on their overlap with TSS (or ot). Intriguingly, we find that TSS are generally harder to classify (Fig 10, S5 Fig). This is perhaps unsurprising since transcription start sites represent only a small fraction of the overall transcribed regions [24]. Interestingly, only approximately 19% of the common OCRs overlap TSSs (Table 1). Given that common OCRs are generally easier to predict, this may also contribute to the lower performance of TSSs. Finally, there may be some previously undetected bias in our labeling tools that could impact the TSS performance.

**Table 1:** OCR-related statistics per cell type.

Cell Type	Number of OCRs	OCRs on transcription	OCRs on TSSs
A549	53,131	11,906	12,809
GM12878	46,722	17,323	10,949
H1	69,101	37,467	14,609
HCT116	33,279	15,863	7,986
HeLa	46,815	10,141	11,172
K562	4,968	3,749	2,966
LNCaP	25,420	8,627	7,354
MCF7	138,717	18,350	13,860
THP1	58,614	11,463	10,522

## Discussion

The goal of our study was to detect whether specific ATAC-seq peaks denoting OCRs overlapped nascent transcription, using a machine learning model and a hybrid signal/sequence representation of each OCR. Using matched ATAC-seq and nascent transcription data for the same cell type and conditions, we ensured diversity of tissue types, experimental sources, and data quality in general for our training, validation, and test sets. Overall, the performance of our hybrid RNN model is solid, with the classifier reaching a maximum AUC score of 0.847 and maximum F1-score of 0.792.

Generally, the quality of both data types (nascent and ATAC) likely influences the overall performance of our classifier. While the datasets utilized here were selected, in part, based on the fact that they pass certain quality standards, it is interesting to note that the lowest performing cell line (K562) was arguably the poorest quality ATAC-seq dataset. This cell line had the fewest detected OCRs, and appeared by visual inspection of lower complexity. While we discarded datasets based on an arbitrary depth cutoff of 10 million reads, issues of SRR complexity are not well captured by simple depth cutoffs.

Our initial hypothesis was that the presence of RNA polymerase II may leave characteristic signatures within ATAC-seq data. However, generally negatives were easier to classify than positives, indicating regions without transcription may follow a more consistent pattern across cell types. Likely several things contribute to the lower performance in positives. First, while we leveraged state of the art nascent analysis tools for our labeling, our error analysis suggests that, even in the high quality datasets utilized here, some lowly transcribed regions may have been missed. Enhancer RNAs are generally lowly transcribed and therefore are inherently challenging to detect even in the best of circumstances. Second, our classifier seeks to identify a singular ATAC pattern for RNA polymerase II presence. Yet nascent transcription assays survey all sites of transcription regardless of which polymerase is involved. Cells have three major forms of RNA polymerase (I, II, and III) and RNA polymerase II is a large multi-protein complex that exists in many forms, e.g. distinct component sets. If each RNA polymerase complex leaves a distinct signature in ATAC, the result would be a mixture of signals and ultimately a reduced performance of our model on the positives, as observed.

A number of extensions are possible that could improve the performance of the classifier. First, we could account for differences between datasets in quality and/or depth by weighting the training inputs based on confidence in the nascent transcription data. Second, we may be able to improve performance by including more signal information into the classifier, as the distinct shapes observed in the meta plots suggests more information on local shape could be informative. Third, our classifier could be extended to utilize annotation or additional input data in order to produce multi-labels (example: TSS versus non-TSS as well as transcribed or not). Even without these extensions, the machine learning features from our hybrid representation of signal and sequence depicted in Fig 1a will likely be applicable to other experimental assays and classification tasks, such as inferring underlying histone modifications. Here we demonstrate that this RNN hybrid model classifier adequately predicts the presence of nascent transcription signal.

## Conclusion

Because of its relative simplicity and utility across a broad range of cell types and cell counts, it is advantageous to maximize the information obtained from ATAC-seq. Here we demonstrated that a recurrent neural network model using a combination of ATAC-seq signal and underlying sequence can accurately classify open chromatin regions as transcribed or not.

## Software availability

The code that implements this model is publicly available at https://github.com/Dowell-Lab/OCR_transcription_detection.

## Supporting information

S1 Table Public sources for ATAC-seq datasets. (PDF)

S2 Table Public sources for nascent transcription datasets. (PDF)

S1 Fig Examples of OCRs overlapping TSSs and at other non-TSS regions. Screenshots illustrating examples of (a) an OCR denoted by ATAC-seq peaks that overlap a TSS, and (b) one that is not over a TSS and is likely related to regulatory regions.
(PDF)

S2 Fig Example test scenario of our leave-one-out-training (LOOT) configuration. This example that tests on OCRs from HeLa cells illustrates which chromosomes did we take OCRs from, for training (green), validation (blue) and testing (purple). In every scenario, OCRs from HCT116 cells from chromosomes chr1 thru chr11 were used for validation (the rest for this cell type were discarded), and we only trained on OCRs from those same chromosomes from all other cell types, testing only on OCRs from chromosomes chr12 thru chrY. This ensures the test set is truly novel every time, and prevents introducing any protocol or cell type-specific bias during training, as well as training on regions that could overlap in coordinates with those OCRs tested.
(PDF)

S3 Fig Chromatin accessibility vs transcription coverage per cell type. Relation between the mean number of ATAC-seq reads versus the mean number of nascent transcription reads for the same OCR, for each of the nine cell types evaluated in this study.
(PDF)

S4 Fig Baseline performance. To determine the baseline performance of the classifier, we predicted whether an OCR had underlying transcription based on the likelihood that the mean number of ATAC-seq reads belonged to the distribution of training positives or negatives.
(PDF)

S5 Fig Classifier performance across the different test sets. Precision/recall curves for OCRs overlapping TSSs (orange) and non-TSS OCRs (green). The blue curves correspond to all OCRs for that test set.
(PDF)

S6 Fig Classification outcomes for unique OCRs per cell type. Proportion of OCRs unique to every cell type (not overlapping in genomic coordinates with OCRs from any other cell type) categorized in the different performance metrics.
(PDF)

S7 Fig Classification outcomes for OCRs shared by only two cell types. Proportion of OCRs shared by just two cell types (overlapping in genomic coordinates) categorized in the different performance metrics.
(PDF)

S8 Fig Classification outcomes for OCRs shared by only three cell types. Proportion of OCRs shared by just three cell types (overlapping in genomic coordinates) categorized in the different performance metrics.
(PDF)

S1 File (ZIP)

S2 File (ZIP)

S3 File (ZIP)
