# Supplementary material for: Combining signal and sequence to detect RNA polymerase initiation in ATAC-seq data
Source: PLoS One. 2020 Apr 30;15(4):e0232332. doi: 10.1371/journal.pone.0232332 (PMC7192442; doi:10.1371/journal.pone.0232332)
Supplement: S1 Table — (PDF) [file pone.0232332.s001.pdf]

Supplemental Table S1

Accession	Cell Type	FastQC Red Flags	Nr. Reads
SRR7140571 [1]	A549	Sequence Duplication Levels	66.5M
SRR7140572 [1]	A549	Sequence Duplication Levels	72.6M
SRR7140573 [1]	A549	Sequence Duplication Levels	84.2M
SRR1822165 [2]	GM12878	Sequence Duplication Levels	192.9M
SRR1822166 [2]	GM12878	Sequence Duplication Levels	56.6M
SRR1822167 [2]	GM12878	Sequence Duplication Levels	85.2M
SRR1822168 [2]	GM12878	Sequence Duplication Levels	62.8M
SRR5007258	H1	Sequence Duplication Levels	56.9M
SRR5007259	H1	Sequence Duplication Levels	51.4M
SRR5876158 [3]	HCT116	Sequence Duplication Levels	44.7M
SRR5876159 [3]	HCT116	Sequence Duplication Levels	31.7M
SRR6216226 [4]	HeLa		13.2M
SRR6216227 [4]	HeLa		11.1M
SRR5128074 [5]	K562	Sequence Duplication Levels	36.8M
SRR3622817 [6]	LNCaP		67.7M
SRR3622818 [6]	LNCaP	Per Base N Content<br>Sequence Duplication Levels	35.7M
SRR3622819 [6]	LNCaP	Sequence Duplication Levels	64.5M
SRR9684005	MCF7		68.1M
SRR9684006	MCF7		23.3M
SRR9684007	MCF7		14.1M
SRR9684008	MCF7		14.1M
SRR9684009	MCF7		22.2M
SRR9684010	MCF7		13.5M
SRR9684011	MCF7		13.5M
SRR9684012	MCF7		23.6M
SRR9684013	MCF7		14.6M
SRR9684014	MCF7		14.3M
SRR8932925	THP1	Sequence Duplication Levels	134.2M
SRR8932927	THP1	Sequence Duplication Levels	101.5M

Public ATAC-seq datasets used for this study, which originate from a variety of cell types. The number of reads reflects depth in millions (M). The FastQC input consisted on the fastq files after trimming the adapter content, before other post-processing steps such as removing duplicate reads. The "Per Base Sequence Content" errors were ignored for these datasets since that is a metric for DNA content.

Cell Type	Number of OCRs not overlapping nascent transcription	Number of OCRs overlapping nascent transcription	Total number of OCRs
A549	41,225	11,906	53,131
GM12878	29,399	17,323	46,722
H1	31,634	37,467	69,101
HCT116	17,416	15,863	33,279
HeLa	36,674	10,141	46,815
K562	1,219	3,749	4,968
LNCaP	16,793	8,627	25,420
MCF7	120,367	18,350	138,717
THP1	47,151	11,463	58,614

OCR counts per cell type, discriminating those that overlap nascent transcription from those that do not.

References

[1] An Integrated Encyclopedia of DNA Elements in the Human Genome. Nature. 2012;489(7414):57–74. doi:10.1038/nature11247.

[2] Schep AN, Buenrostro JD, Denny SK, Schwartz K, Sherlock G, Greenleaf WJ. Structured nucleosome fingerprints enable high-resolution mapping of chromatin architecture within regulatory regions. Genome Research. 2015;25(11):1757–1770. doi:10.1101/gr.192294.115.

[3] Kelso TWR, Porter DK, Amaral ML, Shokhirev MN, Benner C, Hargreaves DC. Chromatin accessibility underlies synthetic lethality of SWI/SNF subunits in ARID1A-mutant cancers. eLife. 2017;6. doi:10.7554/eLife.30506.

[4] Cho SW, Xu J, Sun R, Mumbach MR, Carter AC, Chen YG, et al. Promoter of lncRNA Gene PVT1 Is a Tumor-Suppressor DNA Boundary Element. Cell. 2018;173(6):1398–1412.e22. doi:10.1016/j.cell.2018.03.068.

[5] Fuglerud BM, Lemma RB, Wanichawan P, Sundaram AYM, Eskeland R, Gabrielsen OS. A c-Myb mutant causes deregulated differentiation due to impaired histone binding and abrogated pioneer factor function. Nucleic Acids Research. 2017;45(13):7681–7696. doi:10.1093/nar/gkx364.

[6] Liu Y, Yu S, Dhiman VK, Brunetti T, Eckart H, White KP. Functional assessment of human enhancer activities using whole-genome STARR-sequencing. Genome Biology. 2017;18(1):219. doi:10.1186/s13059-017-1345-5.
