# Supplementary material for: Combining signal and sequence to detect RNA polymerase initiation in ATAC-seq data
Source: PLoS One. 2020 Apr 30;15(4):e0232332. doi: 10.1371/journal.pone.0232332 (PMC7192442; doi:10.1371/journal.pone.0232332)
Supplement: S2 Table — (PDF) [file pone.0232332.s002.pdf]

Supplemental Table S2

Accession	Cell Type	FastQC Red Flags	Nr. Reads
SRR5109937[1]	A549		22.0M
SRR1552485[2]	GM12878		205.6M
SRR1745515[3]	H1		29.0M
SRR1745516[3]	H1		37.0M
SRR1745523[3]	H1		30.9M
SRR1745524[3]	H1		39.9M
SRR1745527[3]	H1		32.0M
SRR1745528[3]	H1		40.7M
SRR574824[4]	H1		98.2M
SRR574825[4]	H1	Sequence Duplication Levels	99.5M
SRR574826[4]	H1		133.7M
SRR1105736[5]	HCT116		69.4M
SRR1105737[5]	HCT116		142.6M
SRR1224573[5]	HCT116		205.2M
SRR2084584[6]	HCT116		18.6M
SRR2084585[6]	HCT116		14.8M
SRR2084586[6]	HCT116	Sequence Quality Histograms	20.7M
SRR2084587[6]	HCT116		16.4M
SRR2084588[6]	HCT116		32.6M
SRR2084589[6]	HCT116		26.6M
SRR2084590[6]	HCT116	Sequence Quality Histograms	35.8M
SRR2084591[6]	HCT116		29.9M
SRR828695[7]	HCT116	Per Sequence GC Content<br>Sequence Duplication Levels	69.4M
SRR828696[7]	HCT116	Sequence Duplication Levels	213.4M
SRR828729[7]	HCT116	Per Sequence GC Content<br>Sequence Duplication Levels	142.6M
SRR10004434[8]	HeLa	Sequence Duplication Levels	35.3M
SRR10004435[8]	HeLa	Sequence Duplication Levels	35.2M
SRR10004436[8]	HeLa	Sequence Duplication Levels	35.7M
SRR10004437[8]	HeLa	Sequence Duplication Levels	35.1M
SRR1823901[9]	K562		23.6M
SRR1823902[9]	K562		20.2M
SRR1648886[10]	LNCaP	Sequence Duplication Levels	29.4M
SRR1648890[10]	LNCaP	Sequence Duplication Levels	27.4M
SRR1648896[10]	LNCaP		17.9M
SRR1648897[10]	LNCaP		38.2M
SRR1648909[10]	LNCaP	Sequence Duplication Levels	53.6M
SRR4090102[11]	MCF7		39.8M
SRR4090103[11]	MCF7		50.0M
SRR10400216	MCF7	Per Sequence GC Content	50.6M
SRR10400217	MCF7	Per Sequence GC Content	42.2M
SRR5109947[1]	THP1		23.6M

Public nascent transcription samples used to assess transcription at 1kbp regions centered at the ATAC-seq peaks. The number of reads reflects depth in millions (M). The FastQC input consisted on the fastq files after trimming the adapter content, before other post-processing steps such as removing duplicate reads. The "Per Base Sequence Content" errors were ignored for these datasets since that is a metric for DNA content.

References

[1] Bouvy-Liivrand M, Hernndez de Sande A, Plnen P, Mehtonen J, Vuorenmaa T, Niskanen H, et al. Analysis of primary microRNA loci from nascent transcriptomes reveals regulatory domains governed by chromatin architecture. Nucleic Acids Research. 2017;45(17):9837–9849. doi:10.1093/nar/gkx680.

[2] Core LJ, Martins AL, Danko CG, Waters CT, Siepel A, Lis JT. Analysis of nascent RNA identifies a unified architecture of initiation regions at mammalian promoters and enhancers. Nature Genetics. 2014;46(12):1311–1320. doi:10.1038/ng.3142.

[3] Estars C, Benner C, Jones KA. SMADs and YAP compete to control elongation of B-catenin:LEF-1-recruited RNAPII during hESC differentiation. Molecular Cell. 2015;58(5):780–793. doi:10.1016/j.molcel.2015.04.001.

[4] Sigova AA, Mullen AC, Molinie B, Gupta S, Orlando DA, Guenther MG, et al. Divergent transcription of long noncoding RNA/mRNA gene pairs in embryonic stem cells. Proceedings of the National Academy of Sciences of the United States of America. 2013;110(8):2876–2881. doi:10.1073/pnas.1221904110.

[5] Allen MA, Andrysik Z, Dengler VL, Mellert HS, Guarnieri A, Freeman JA, et al. Global analysis of p53-regulated transcription identifies its direct targets and unexpected regulatory mechanisms. eLife. 2014;3:e02200. doi:10.7554/eLife.02200.

[6] Chen Y, Wang Y, Xuan Z, Chen M, Zhang MQ. De novo deciphering three-dimensional chromatin interaction and topological domains by wavelet transformation of epigenetic profiles. Nucleic Acids Research. 2016;44(11):e106–e106. doi:10.1093/nar/gkw225.

[7] Galbraith MD, Allen MA, Bensard CL, Wang X, Schwinn MK, Qin B, et al. HIF1A employs CDK8-mediator to stimulate RNAPII elongation in response to hypoxia. Cell. 2013;153(6):1327–1339. doi:10.1016/j.cell.2013.04.048.

[8] Bahat A, Lahav O, Plotnikov A, Leshkowitz D, Dikstein R. Targeting Spt5-Pol II by Small-Molecule Inhibitors Uncouples Distinct Activities and Reveals Additional Regulatory Roles. Molecular Cell. 2019;doi:10.1016/j.molcel.2019.08.024.

[9] Niskanen EA, Malinen M, Sutinen P, Toropainen S, Paakinaho V, Vihervaara A, et al. Global SUMOylation on active chromatin is an acute heat stress response restricting transcription. Genome Biology. 2015;16:153. doi:10.1186/s13059-015-0717-y.

[10] Puc J, Kozbial P, Li W, Tan Y, Liu Z, Suter T, et al. Ligand-dependent enhancer activation regulated by topoisomerase-I activity. Cell. 2015;160(3):367–380. doi:10.1016/j.cell.2014.12.023.

[11] Andrysik Z, Galbraith MD, Guarnieri AL, Zaccara S, Sullivan KD, Pandey A, et al. Identification of a core TP53 transcriptional program with highly distributed tumor suppressive activity. Genome Research. 2017;27(10):1645–1657. doi:10.1101/gr.220533.117.
