# Supplementary material for: Combining signal and sequence to detect RNA polymerase initiation in ATAC-seq data
Source: PLoS One. 2020 Apr 30;15(4):e0232332. doi: 10.1371/journal.pone.0232332 (PMC7192442; doi:10.1371/journal.pone.0232332)
Supplement: S1 Fig — Screenshots illustrating examples of (a) an OCR denoted by ATAC-seq peaks that overlap a TSS, and (b) one that is not over a TSS and is likely related to regulatory regions. (PDF) [file pone.0232332.s003.pdf]

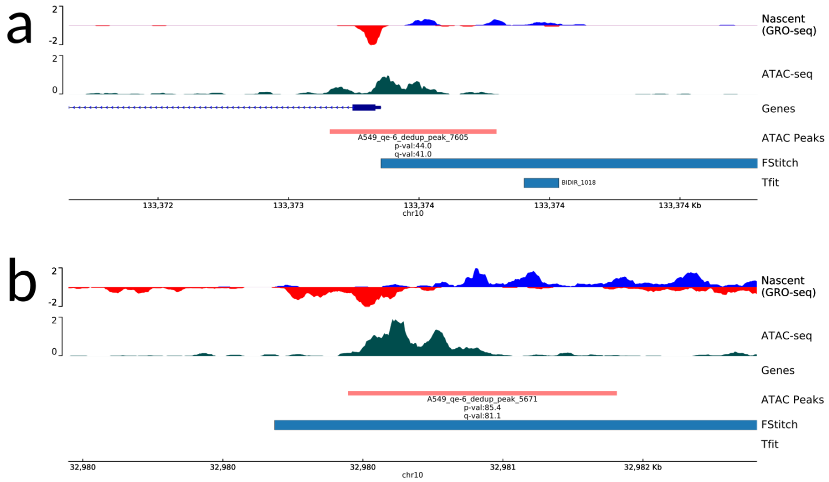

Figure 1: **Examples of OCRs overlapping TSSs and at other non-TSS regions.** Screenshots illustrating examples of **(a)** an OCR denoted by ATAC-seq peaks that overlap a TSS, and **(b)** one that is not over a TSS and is likely related to regulatory regions.
