# Supplementary material for: Combining signal and sequence to detect RNA polymerase initiation in ATAC-seq data
Source: PLoS One. 2020 Apr 30;15(4):e0232332. doi: 10.1371/journal.pone.0232332 (PMC7192442; doi:10.1371/journal.pone.0232332)
Supplement: S2 Fig — This example that tests on OCRs from HeLa cells illustrates which chromosomes did we take OCRs from, for training (green), validation (blue) and testing (purple). In every scenario, OCRs from HCT116 cells from chromosomes chr1 thru chr11 were used for validation (the rest for this cell type were discarded), and we only trained on OCRs from those same chromosomes from all other cell types, testing only on OCRs from chromosomes chr12 thru chrY. This ensures the test set is truly novel every time, and prevents introducing any protocol or cell type-specific bias during training, as well as training on regions that could overlap in coordinates with those OCRs tested. (PDF) [file pone.0232332.s004.pdf]

Cell Type	chr1	chr2	...	chr11	chr12	...	chr21	chr22	chrX	chrY
A549			...			...				
GM12878			...			...				
H1			...			...				
HeLa			...			...				
LNCaP			...			...				
MCF7			...			...				
THP1			...			...				
HCT116			...			...				

Figure 2: **Example test scenario of our leave-one-out-training (LOOT) configuration.** This example that tests on OCRs from HeLa cells illustrates which chromosomes did we take OCRs from, for training (green), validation (blue) and testing (purple). In every scenario, OCRs from HCT116 cells from chromosomes chr1 thru chr11 were used for validation (the rest for this cell type were discarded), and we only trained on OCRs from those same chromosomes from all other cell types, testing only on OCRs from chromosomes chr12 thru chrY. This ensures the test set is truly novel every time, and prevents introducing any protocol or cell type-specific bias during training, as well as training on regions that could overlap in coordinates with those OCRs tested.
