# Supplementary material for: Combining signal and sequence to detect RNA polymerase initiation in ATAC-seq data
Source: PLoS One. 2020 Apr 30;15(4):e0232332. doi: 10.1371/journal.pone.0232332 (PMC7192442; doi:10.1371/journal.pone.0232332)
Supplement: S3 Fig — Relation between the mean number of ATAC-seq reads versus the mean number of nascent transcription reads for the same OCR, for each of the nine cell types evaluated in this study. (PDF) [file pone.0232332.s005.pdf]

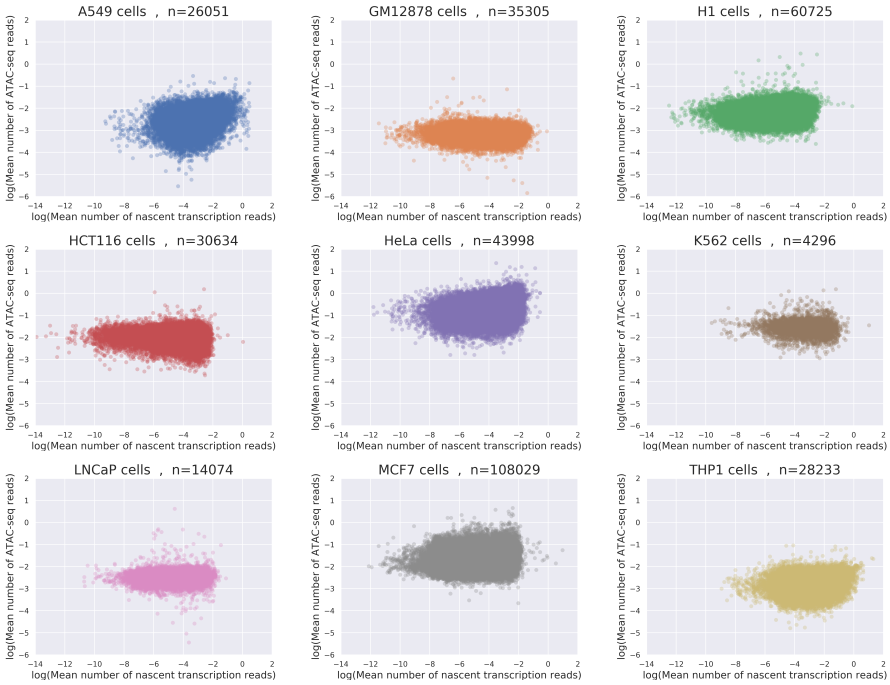

Figure 3: **Chromatin accessibility vs transcription coverage per cell type.** Relation between the mean number of ATAC-seq reads versus the mean number of nascent transcription reads for the same OCR, for each of the nine cell types evaluated in this study.
