# Supplementary material for: Combining signal and sequence to detect RNA polymerase initiation in ATAC-seq data
Source: PLoS One. 2020 Apr 30;15(4):e0232332. doi: 10.1371/journal.pone.0232332 (PMC7192442; doi:10.1371/journal.pone.0232332)
Supplement: S4 Fig — To determine the baseline performance of the classifier, we predicted whether an OCR had underlying transcription based on the likelihood that the mean number of ATAC-seq reads belonged to the distribution of training positives or negatives. (PDF) [file pone.0232332.s006.pdf]

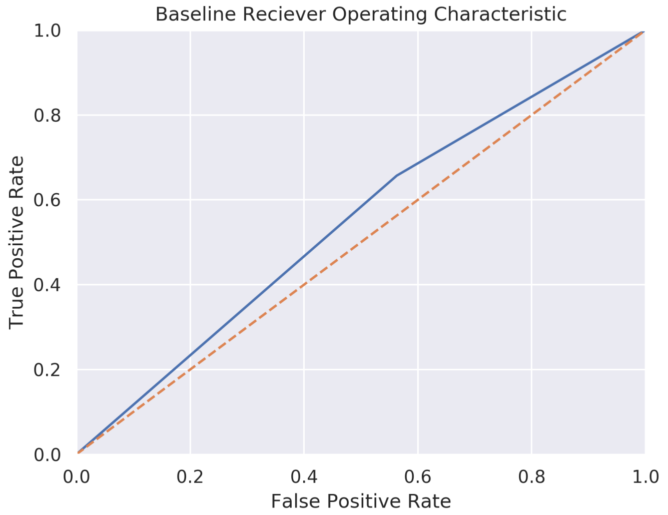

Figure 4: **Baseline performance.** To determine the baseline performance of the classifier, we predicted whether an OCR had underlying transcription based on the likelihood that the mean number of ATAC-seq reads belonged to the distribution of training positives or negatives.
