# Supplementary material for: Combining signal and sequence to detect RNA polymerase initiation in ATAC-seq data
Source: PLoS One. 2020 Apr 30;15(4):e0232332. doi: 10.1371/journal.pone.0232332 (PMC7192442; doi:10.1371/journal.pone.0232332)
Supplement: S5 Fig — Precision/recall curves for OCRs overlapping TSSs (orange) and non-TSS OCRs (green). The blue curves correspond to all OCRs for that test set. (PDF) [file pone.0232332.s007.pdf]

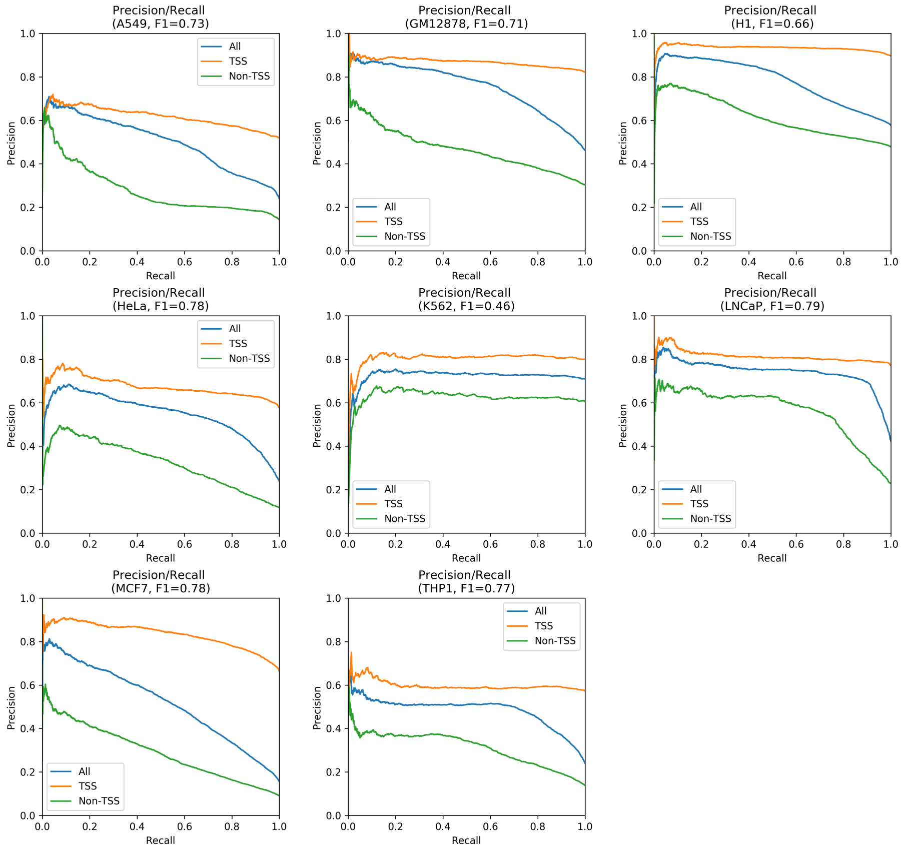

Figure 5: **Classifier performance across the different test sets.** Precision/recall curves for OCRs overlapping TSSs (orange) and non-TSS OCRs (green). The blue curves correspond to all OCRs for that test set.
