# Supplementary material for: Combining signal and sequence to detect RNA polymerase initiation in ATAC-seq data
Source: PLoS One. 2020 Apr 30;15(4):e0232332. doi: 10.1371/journal.pone.0232332 (PMC7192442; doi:10.1371/journal.pone.0232332)
Supplement: S6 Fig — Proportion of OCRs unique to every cell type (not overlapping in genomic coordinates with OCRs from any other cell type) categorized in the different performance metrics. (PDF) [file pone.0232332.s008.pdf]

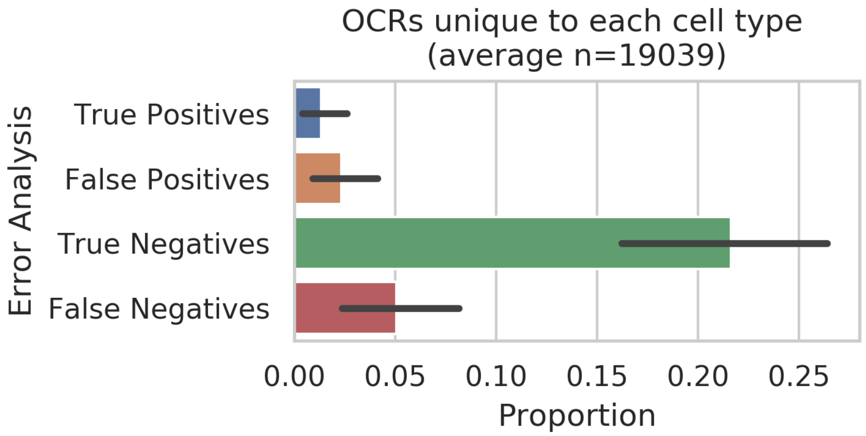


Figure 6: **Classification outcomes for unique OCRs per cell type.** Proportion of OCRs unique to every cell type (not overlapping in genomic coordinates with OCRs from any other cell type) categorized in the different performance metrics.
