# Supplementary material for: Combining signal and sequence to detect RNA polymerase initiation in ATAC-seq data
Source: PLoS One. 2020 Apr 30;15(4):e0232332. doi: 10.1371/journal.pone.0232332 (PMC7192442; doi:10.1371/journal.pone.0232332)
Supplement: S7 Fig — Proportion of OCRs shared by just two cell types (overlapping in genomic coordinates) categorized in the different performance metrics. (PDF) [file pone.0232332.s009.pdf]

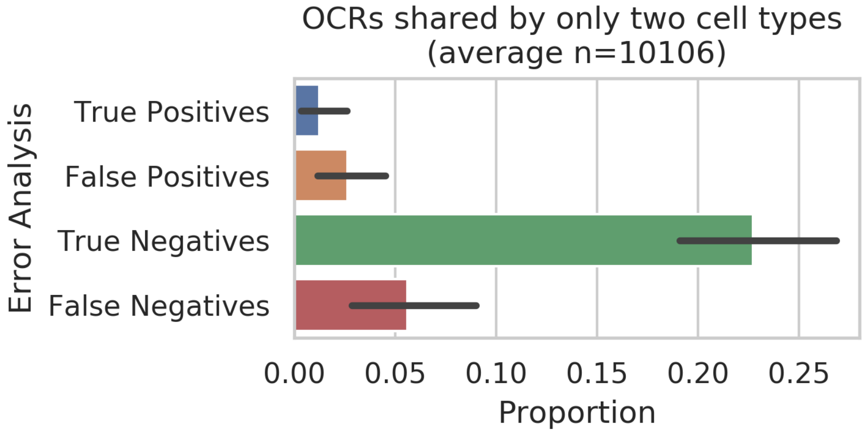

Figure 7: **Classification outcomes for OCRs shared by only two cell types.** Proportion of OCRs shared by just two cell types (overlapping in genomic coordinates) categorized in the different performance metrics.
