# Supplementary figures and images for: Combining signal and sequence to detect RNA polymerase initiation in ATAC-seq data (part 2 of 2)
Source: PLoS One. 2020 Apr 30;15(4):e0232332. doi: 10.1371/journal.pone.0232332 (PMC7192442; doi:10.1371/journal.pone.0232332)

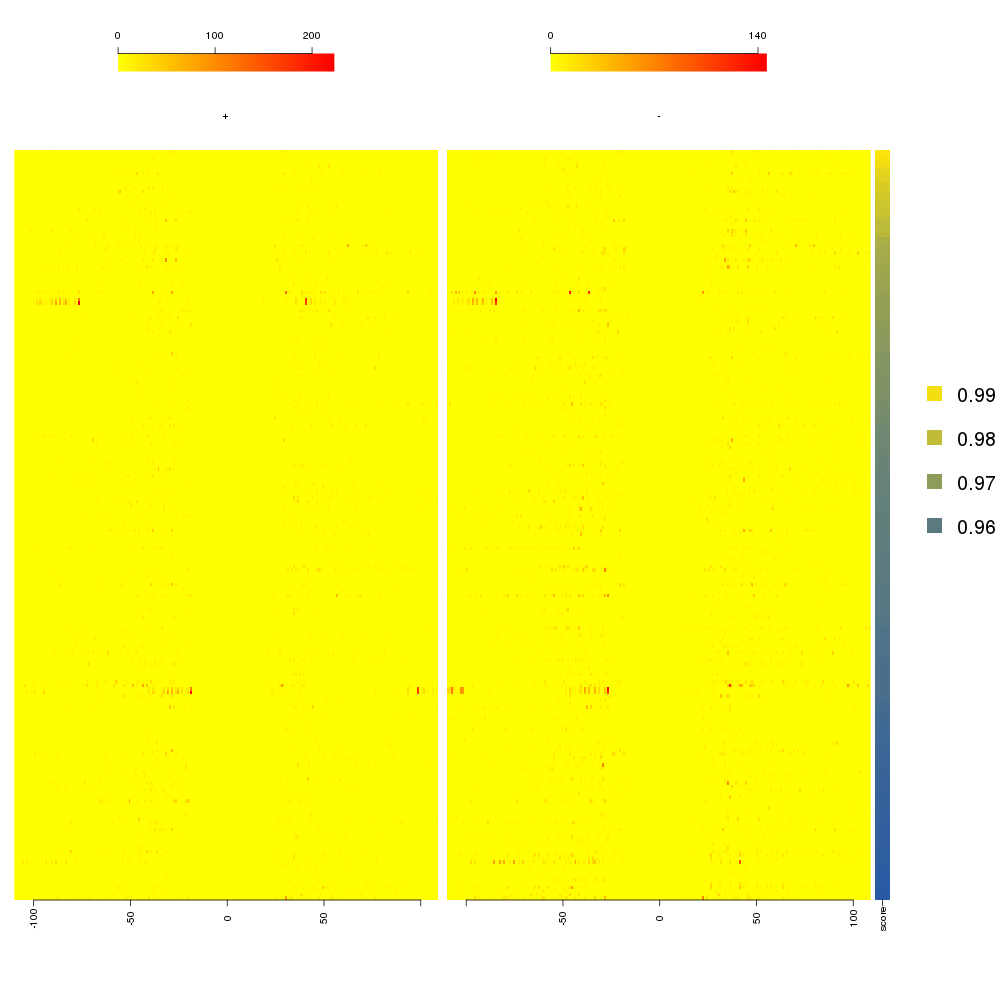

Supplement: S2 File — (ZIP) [file pone.0232332.s012.zip › nucleosome_positioning/SRX6443489_feature_aligned_heatmap.png]

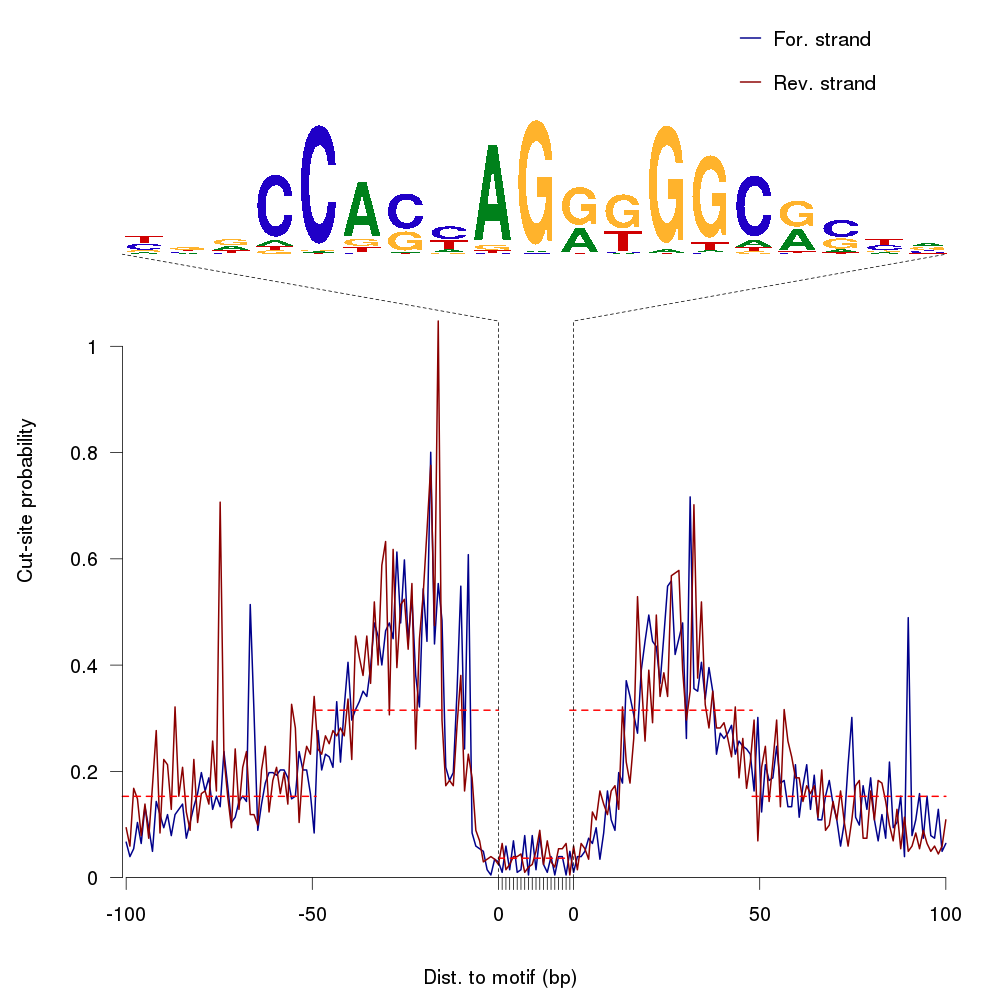

Supplement: S2 File — (ZIP) [file pone.0232332.s012.zip › nucleosome_positioning/SRR5063984_footprint_plot.png]

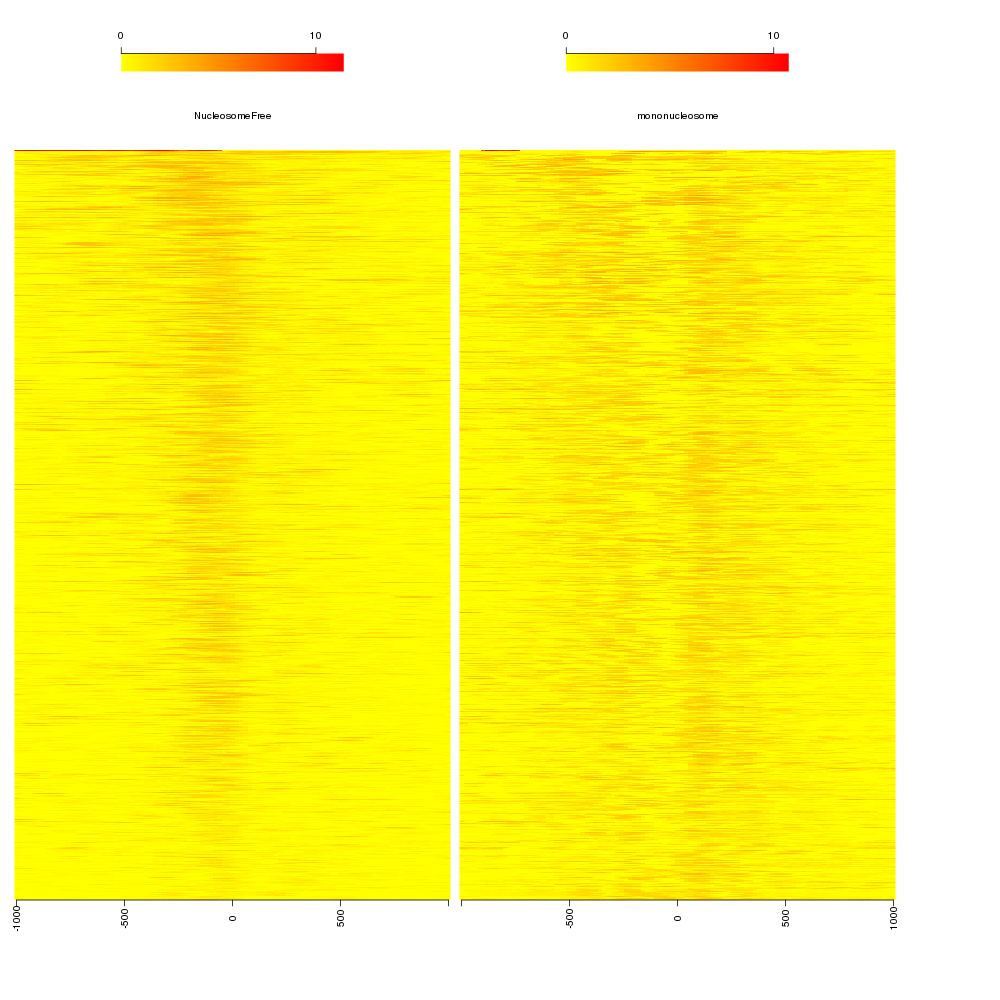

Supplement: S2 File — (ZIP) [file pone.0232332.s012.zip › nucleosome_positioning/SRR5063985_nucleosome_heatmap.png]

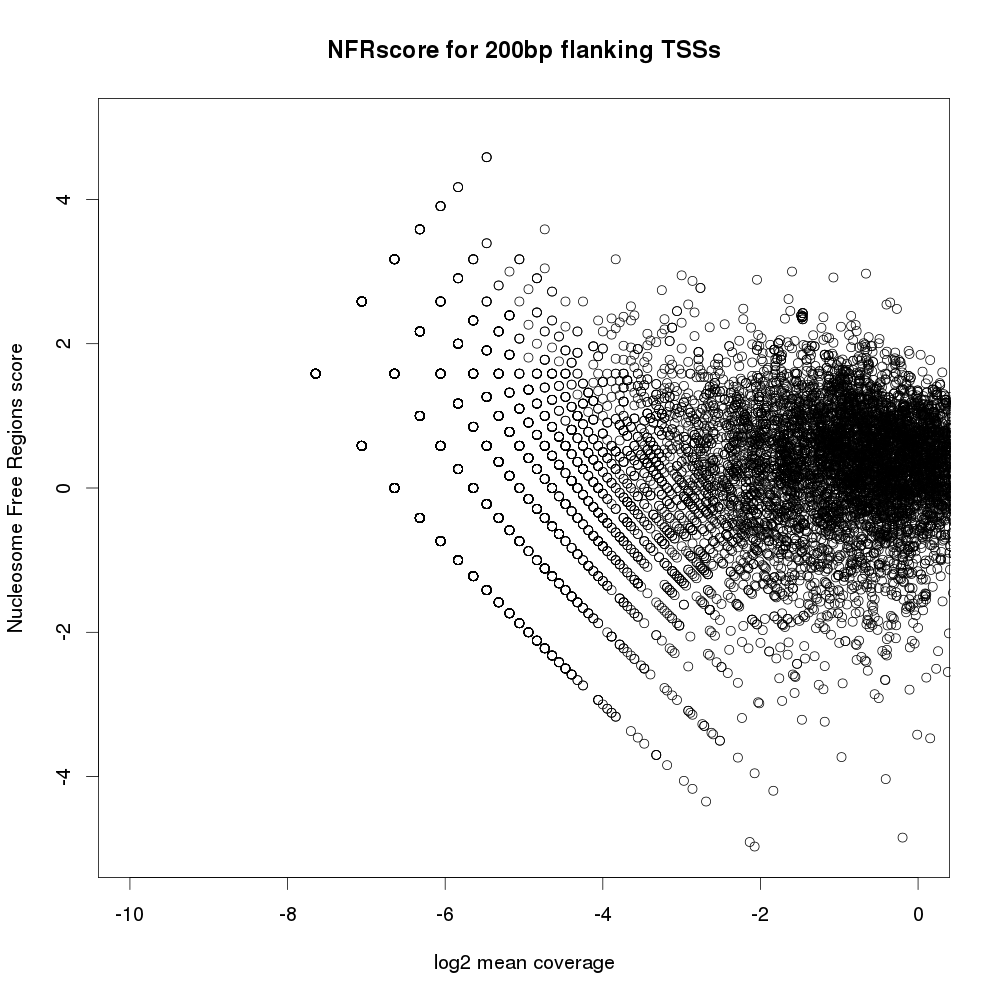

Supplement: S2 File — (ZIP) [file pone.0232332.s012.zip › nucleosome_positioning/SRR5007259_NFRscore.png]

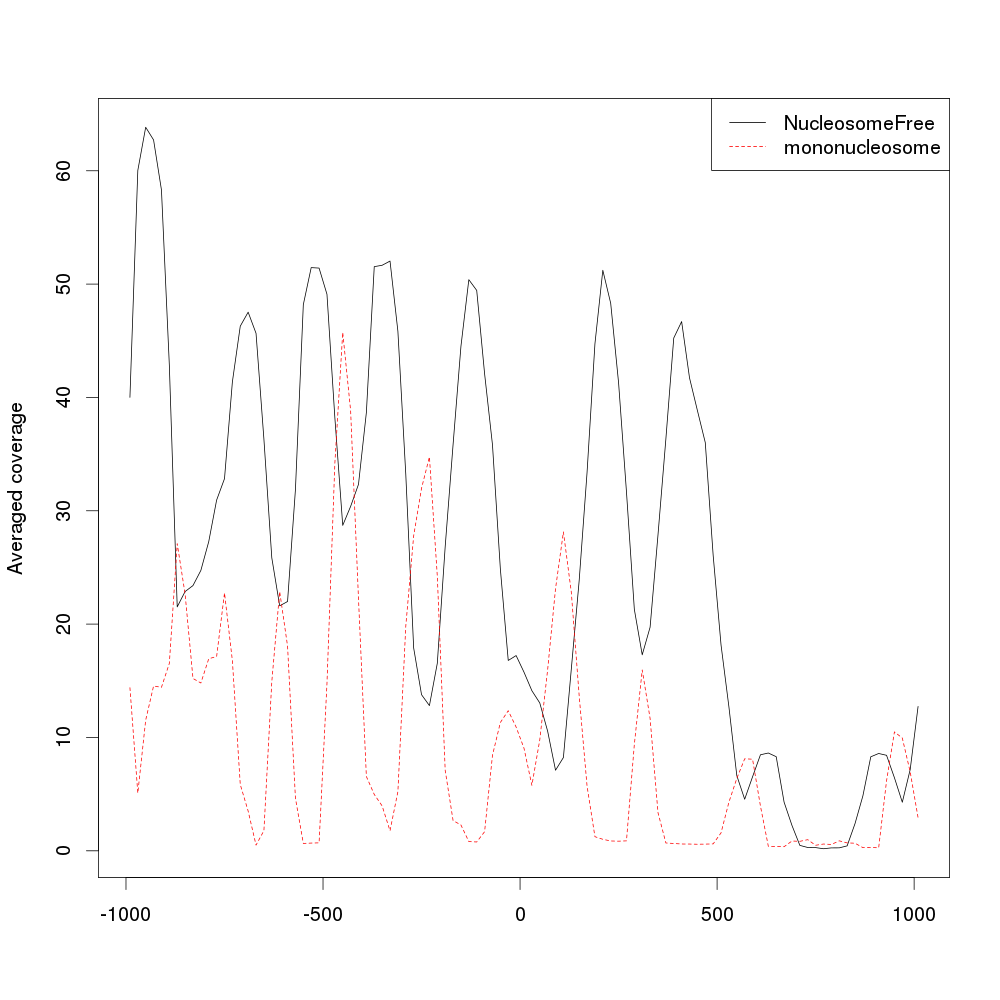

Supplement: S2 File — (ZIP) [file pone.0232332.s012.zip › nucleosome_positioning/SRR1822168_nucleosome_distribution.png]

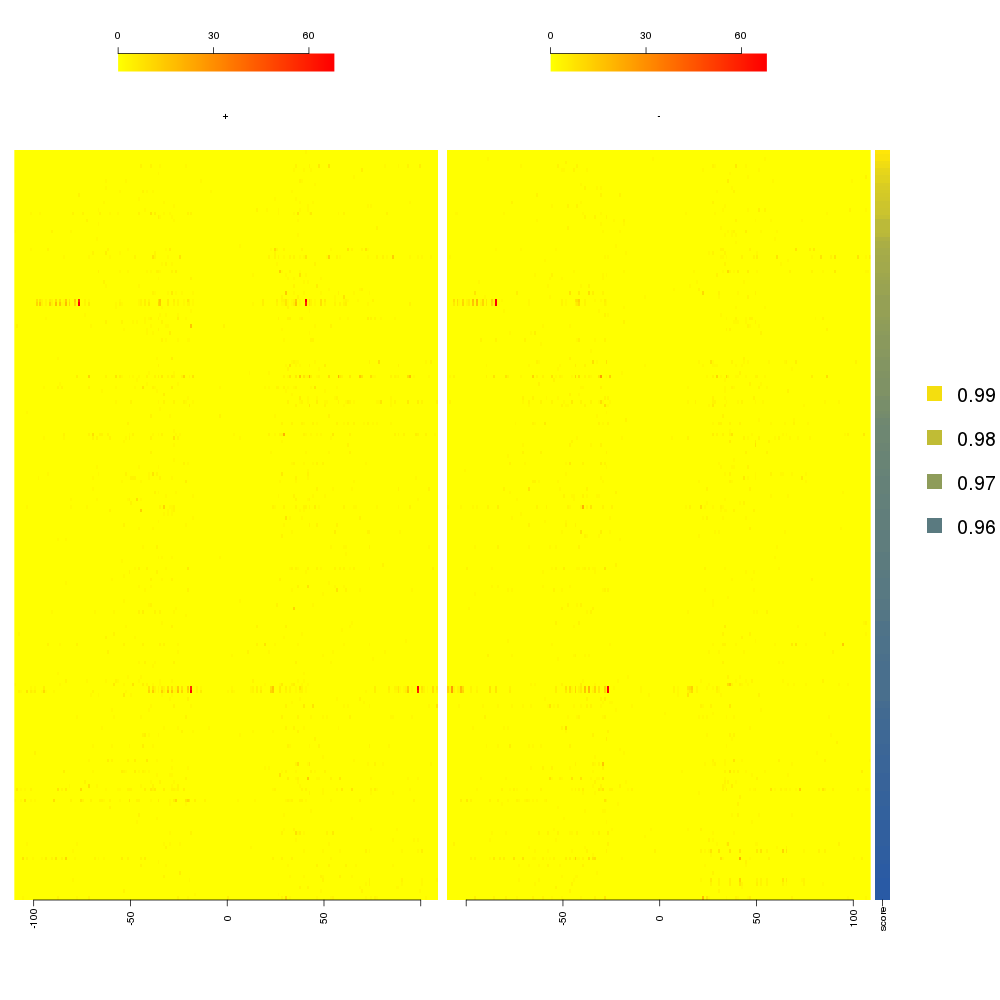

Supplement: S2 File — (ZIP) [file pone.0232332.s012.zip › nucleosome_positioning/SRR8932925_feature_aligned_heatmap.png]

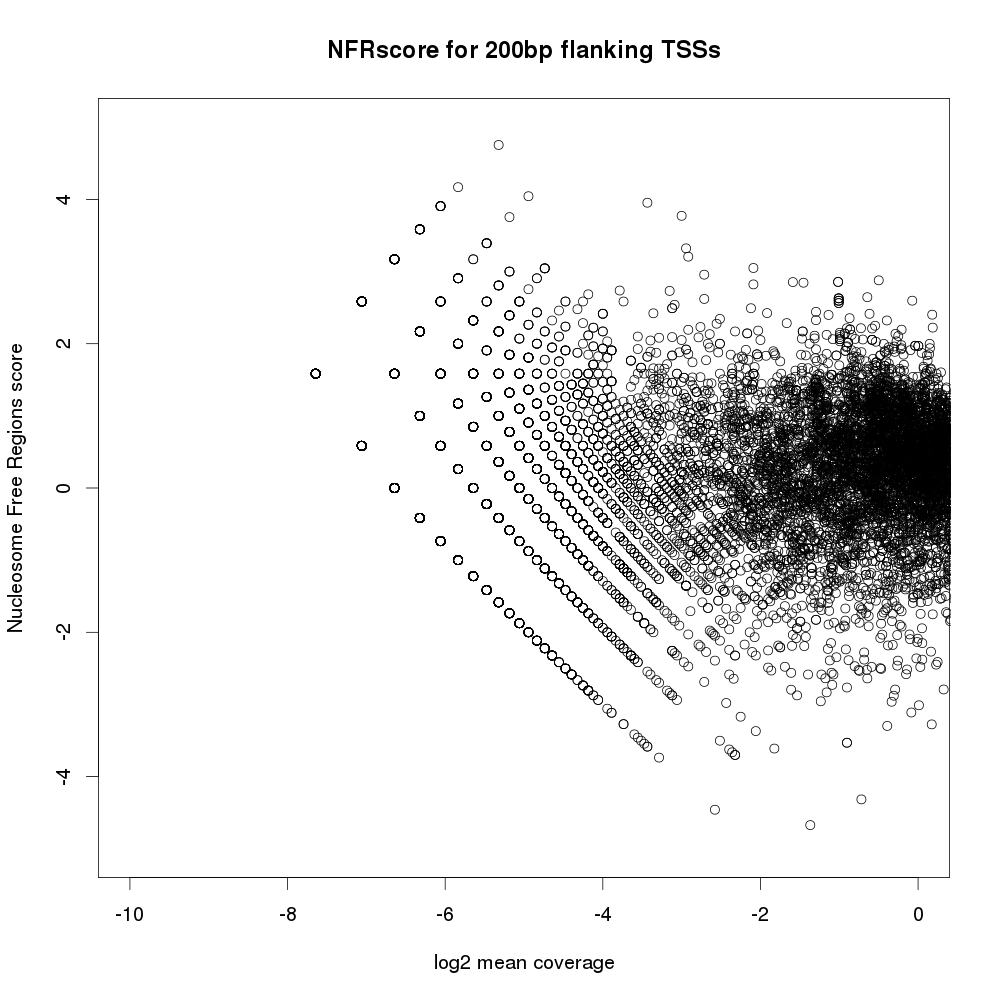

Supplement: S2 File — (ZIP) [file pone.0232332.s012.zip › nucleosome_positioning/SRR5007258_NFRscore.png]

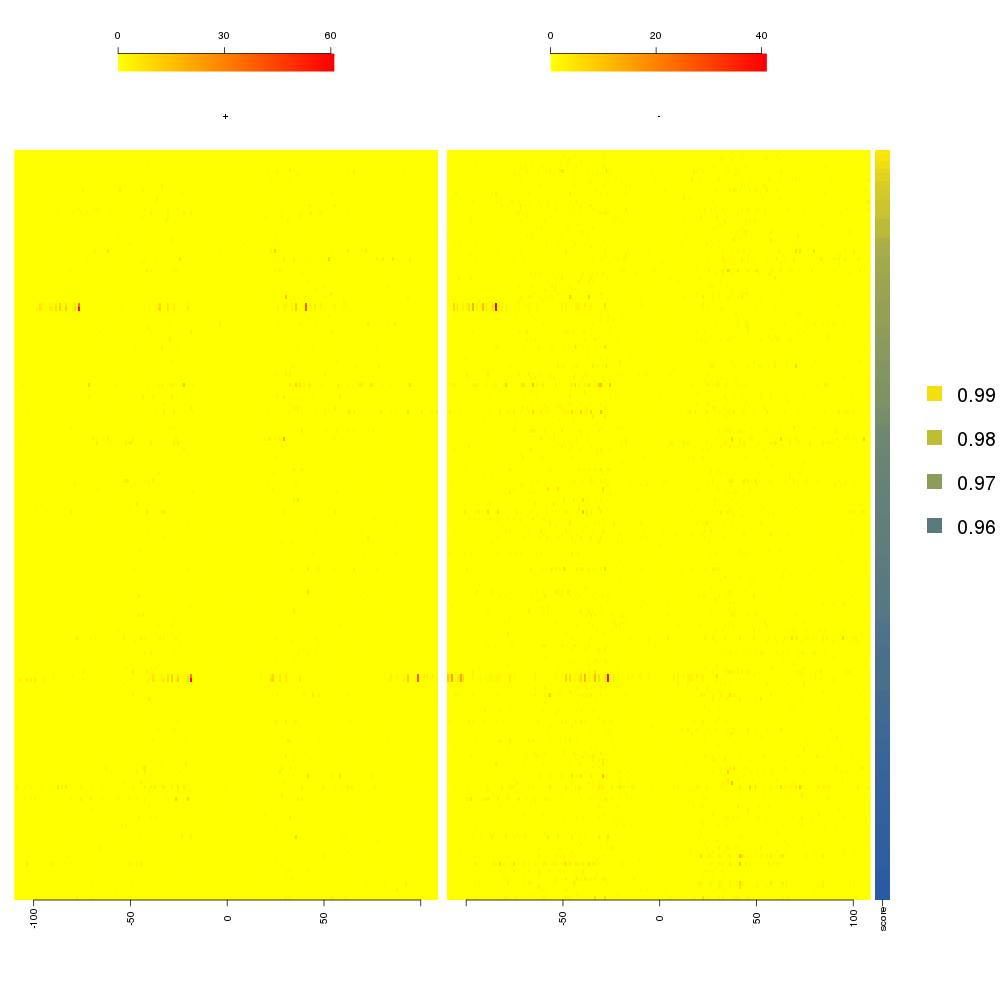

Supplement: S2 File — (ZIP) [file pone.0232332.s012.zip › nucleosome_positioning/SRR8932927_feature_aligned_heatmap.png]

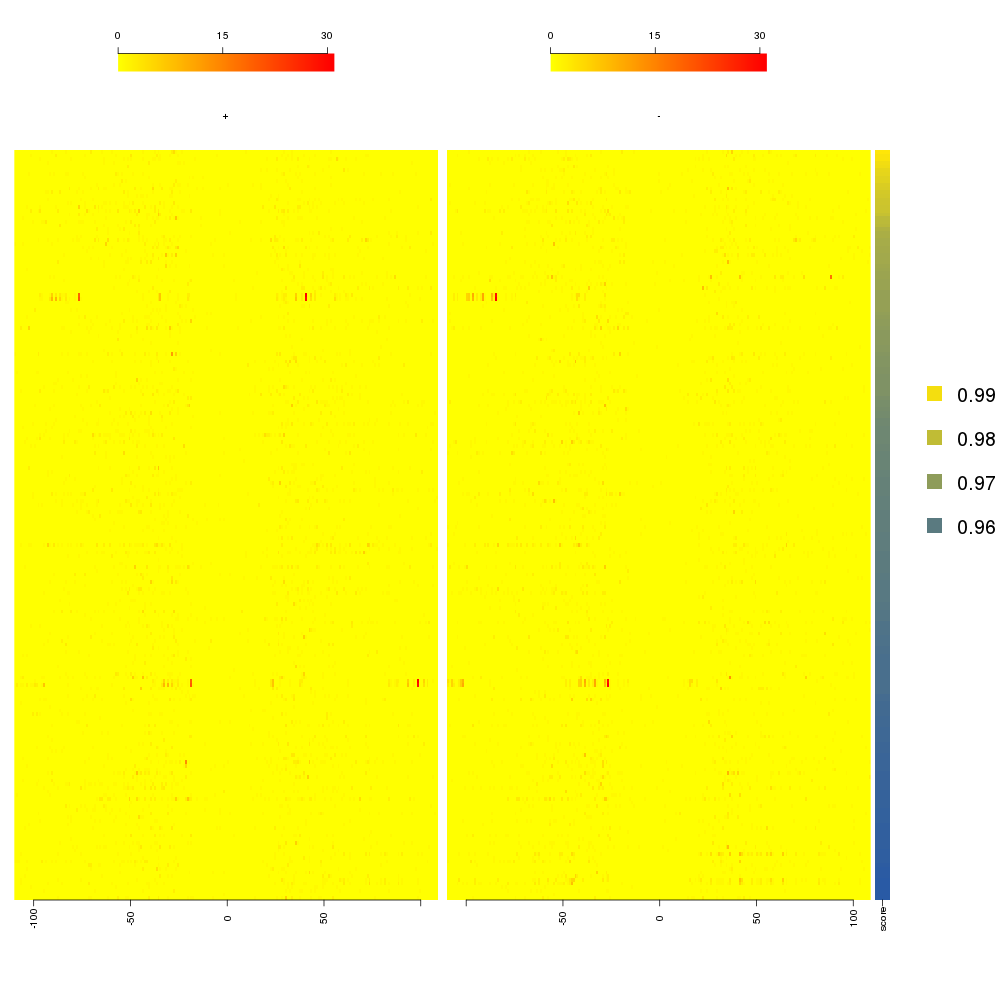

Supplement: S2 File — (ZIP) [file pone.0232332.s012.zip › nucleosome_positioning/SRR5063985_feature_aligned_heatmap.png]

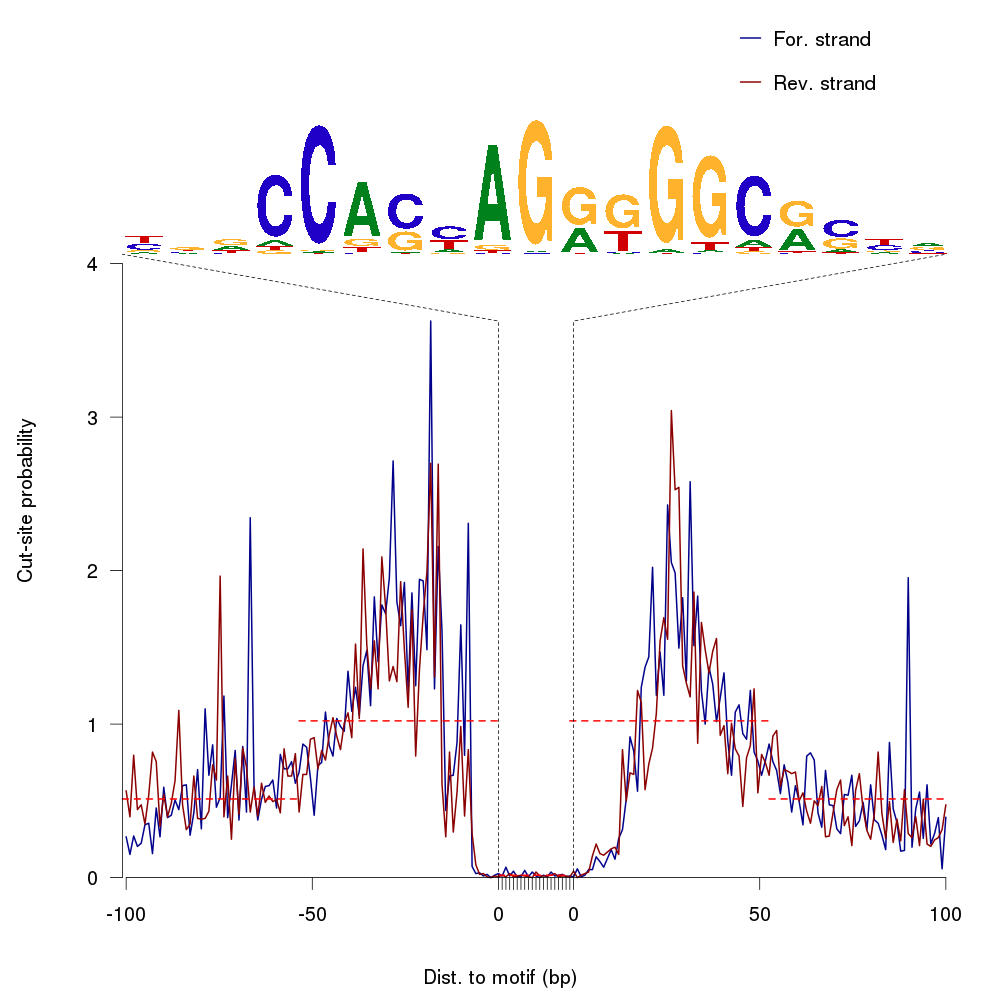

Supplement: S2 File — (ZIP) [file pone.0232332.s012.zip › nucleosome_positioning/SRX6443490_footprint_plot.png]

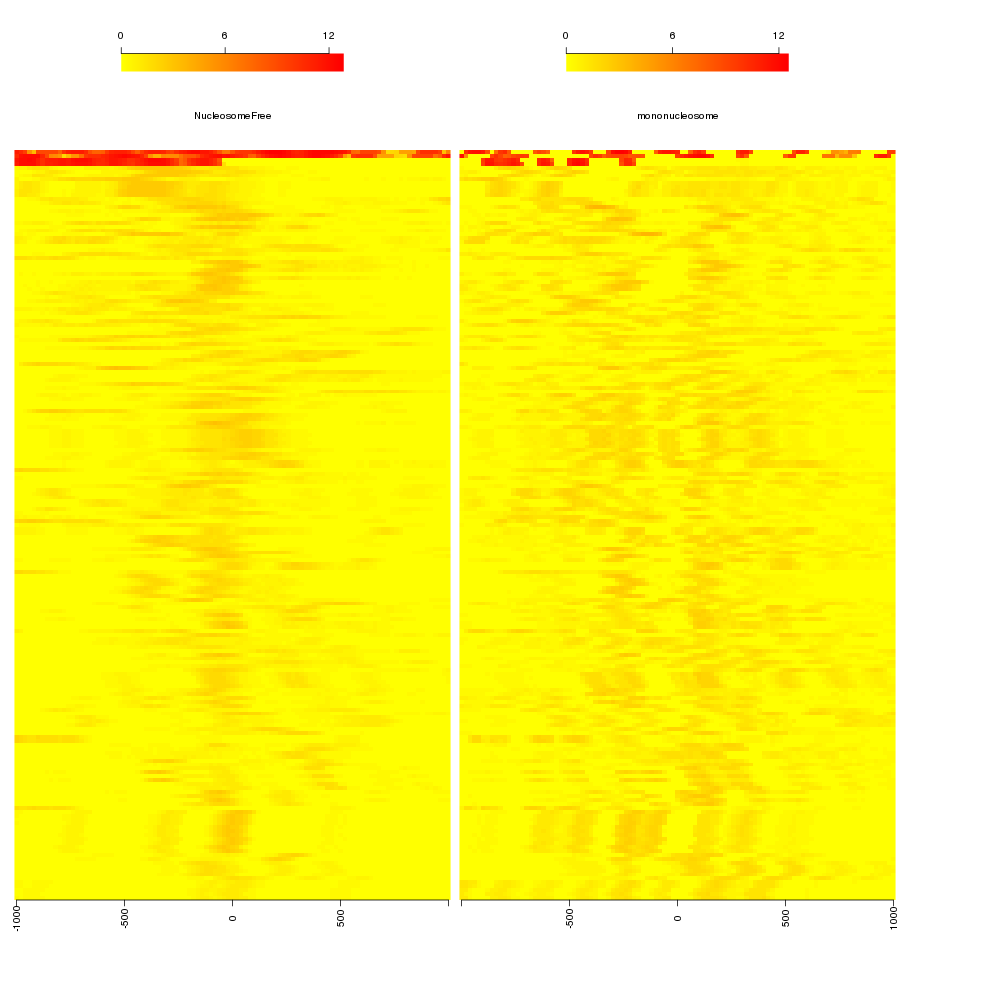

Supplement: S2 File — (ZIP) [file pone.0232332.s012.zip › nucleosome_positioning/SRR1822167_nucleosome_heatmap.png]

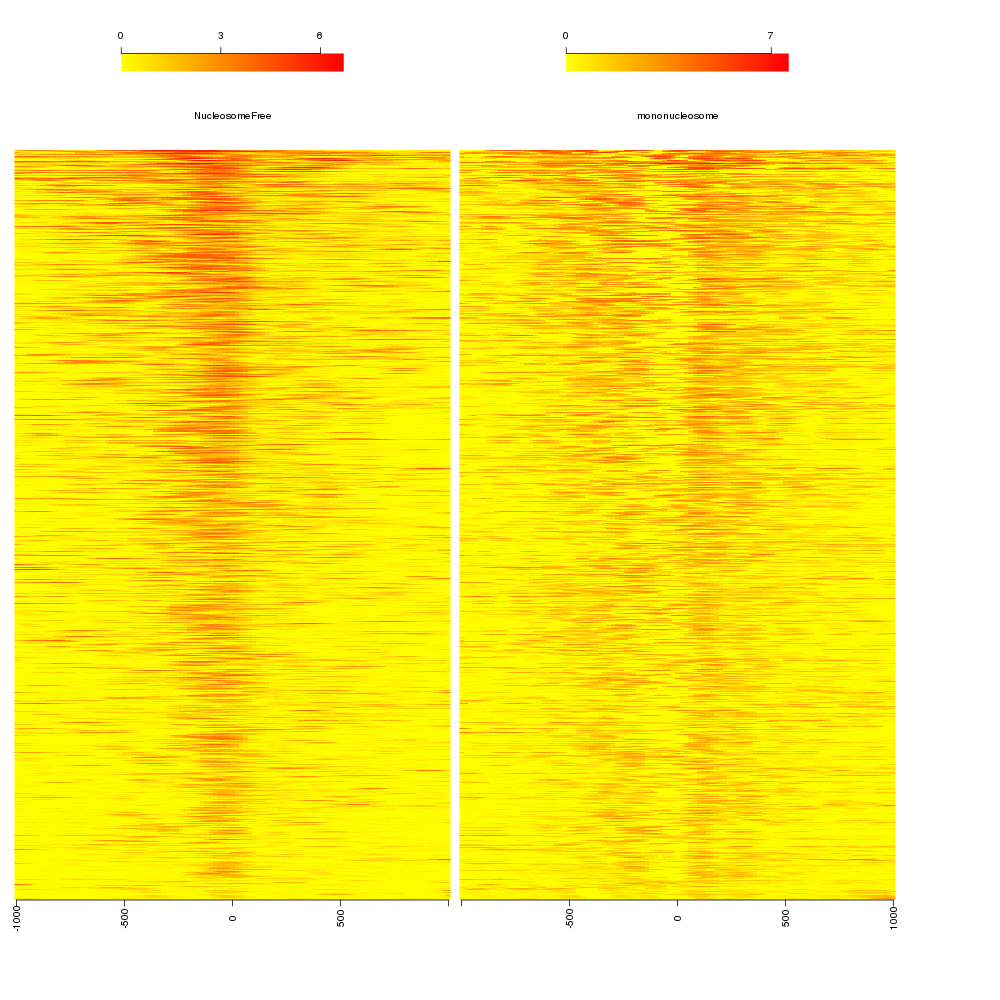

Supplement: S2 File — (ZIP) [file pone.0232332.s012.zip › nucleosome_positioning/SRX6443490_nucleosome_heatmap.png]

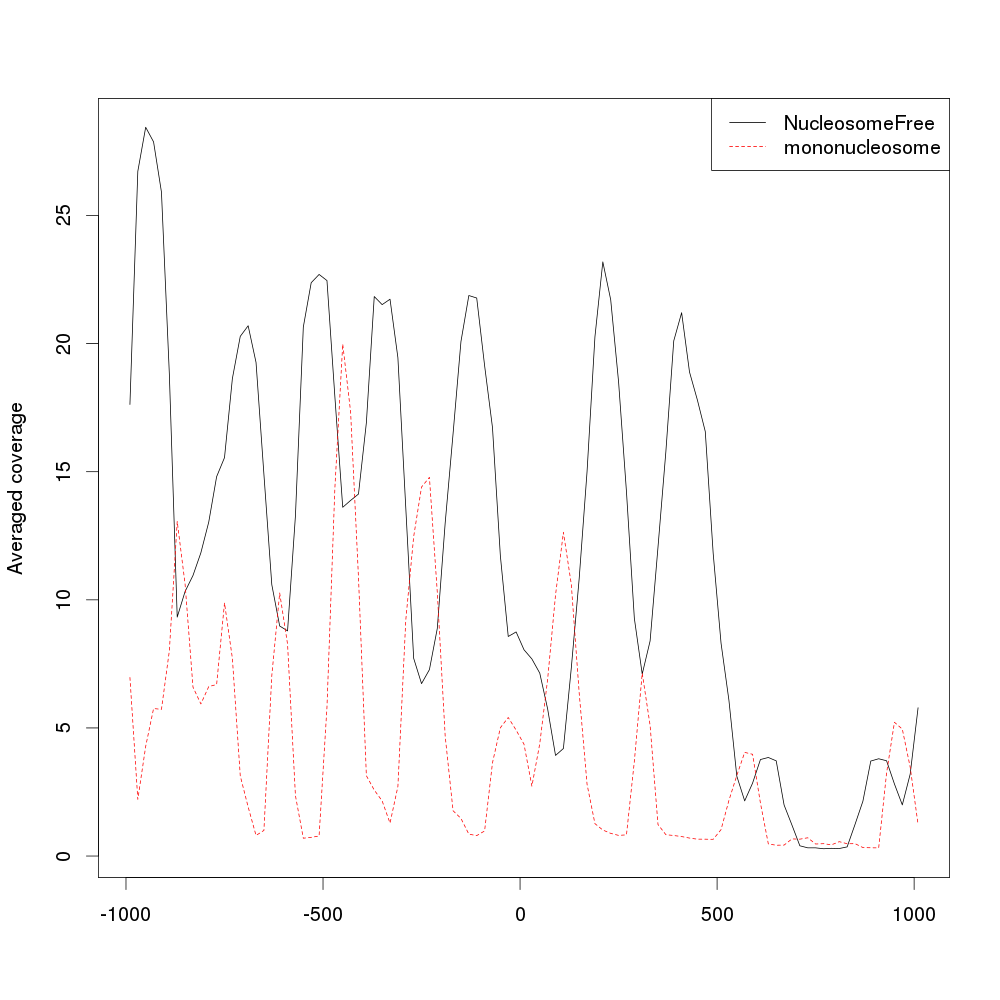

Supplement: S2 File — (ZIP) [file pone.0232332.s012.zip › nucleosome_positioning/SRR1822166_nucleosome_distribution.png]

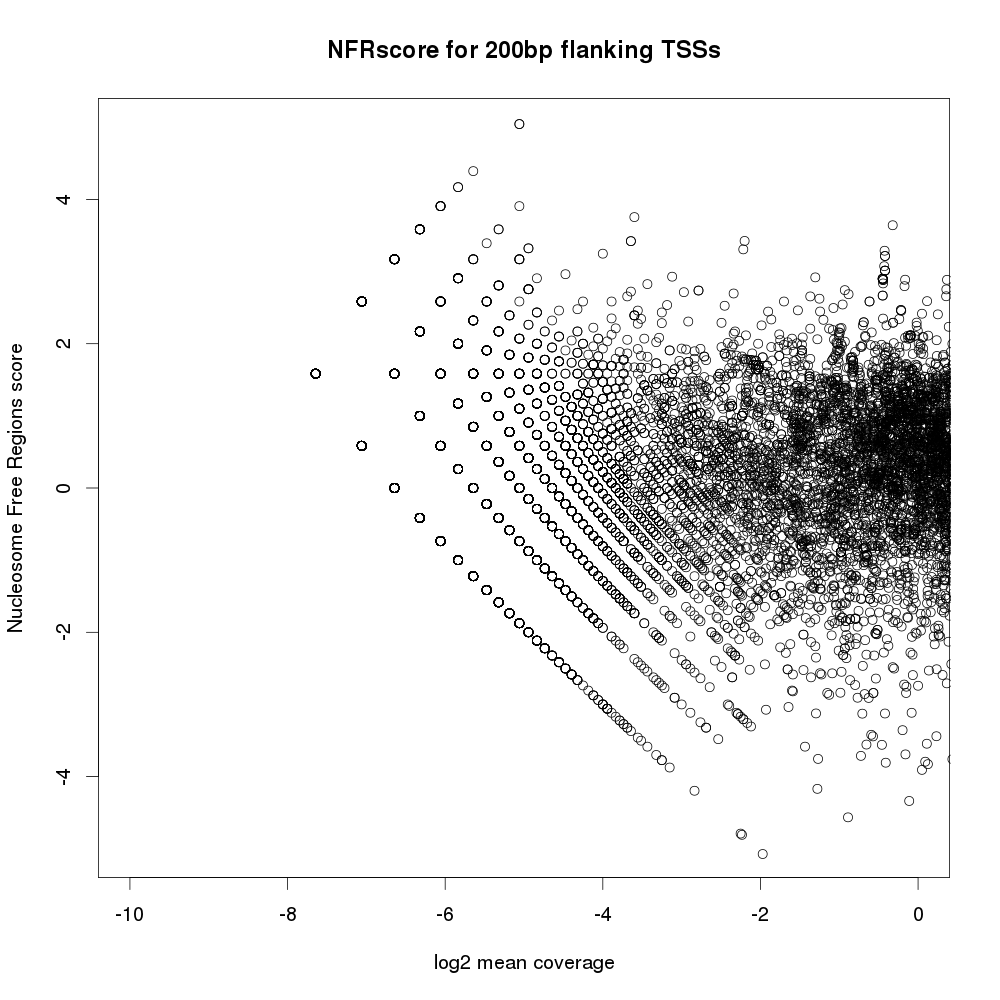

Supplement: S2 File — (ZIP) [file pone.0232332.s012.zip › nucleosome_positioning/SRX6443489_NFRscore.png]

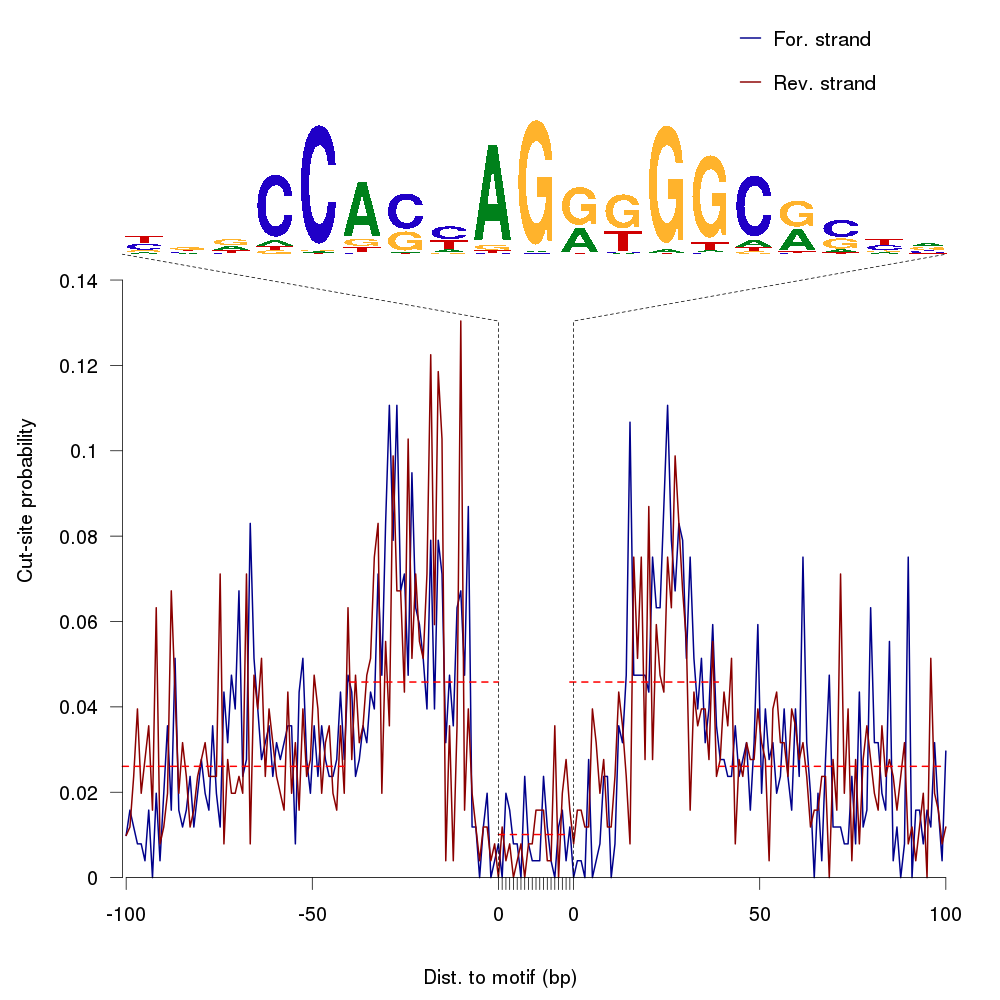

Supplement: S2 File — (ZIP) [file pone.0232332.s012.zip › nucleosome_positioning/SRR3622818_footprint_plot.png]

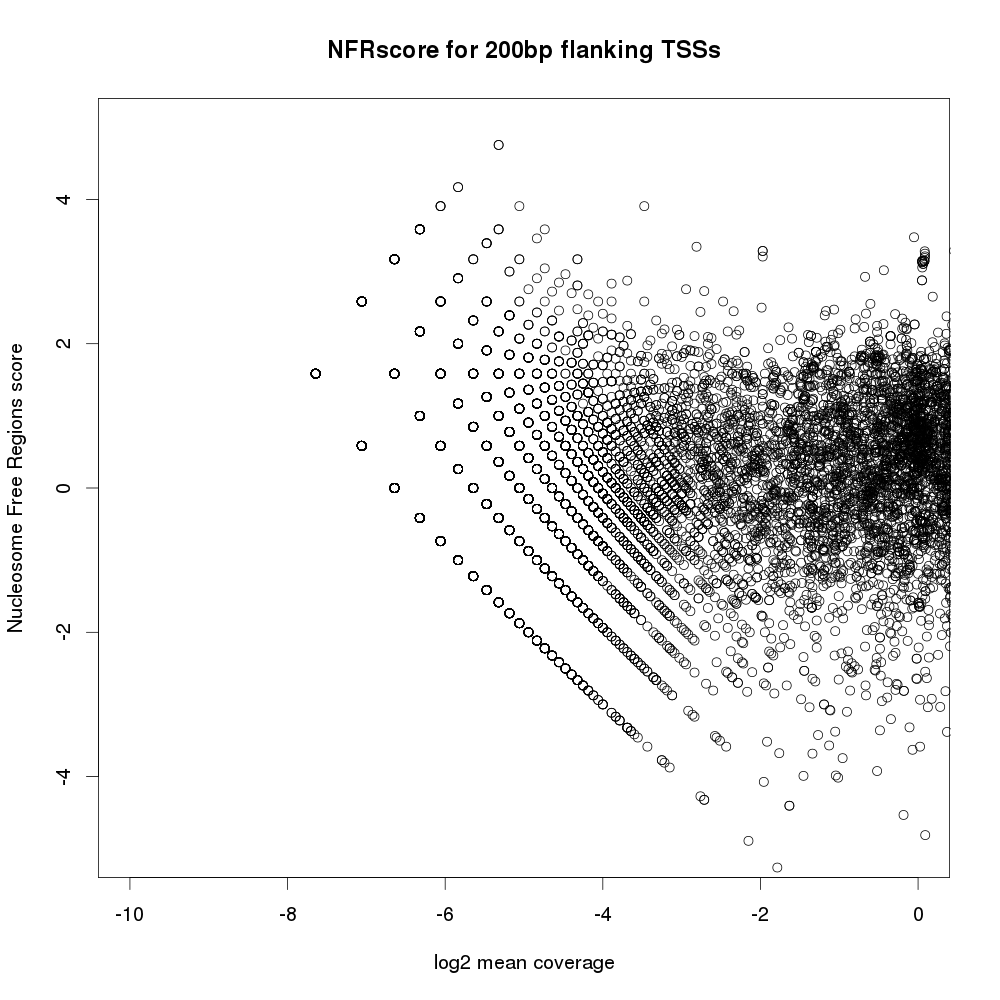

Supplement: S2 File — (ZIP) [file pone.0232332.s012.zip › nucleosome_positioning/SRX6443488_NFRscore.png]

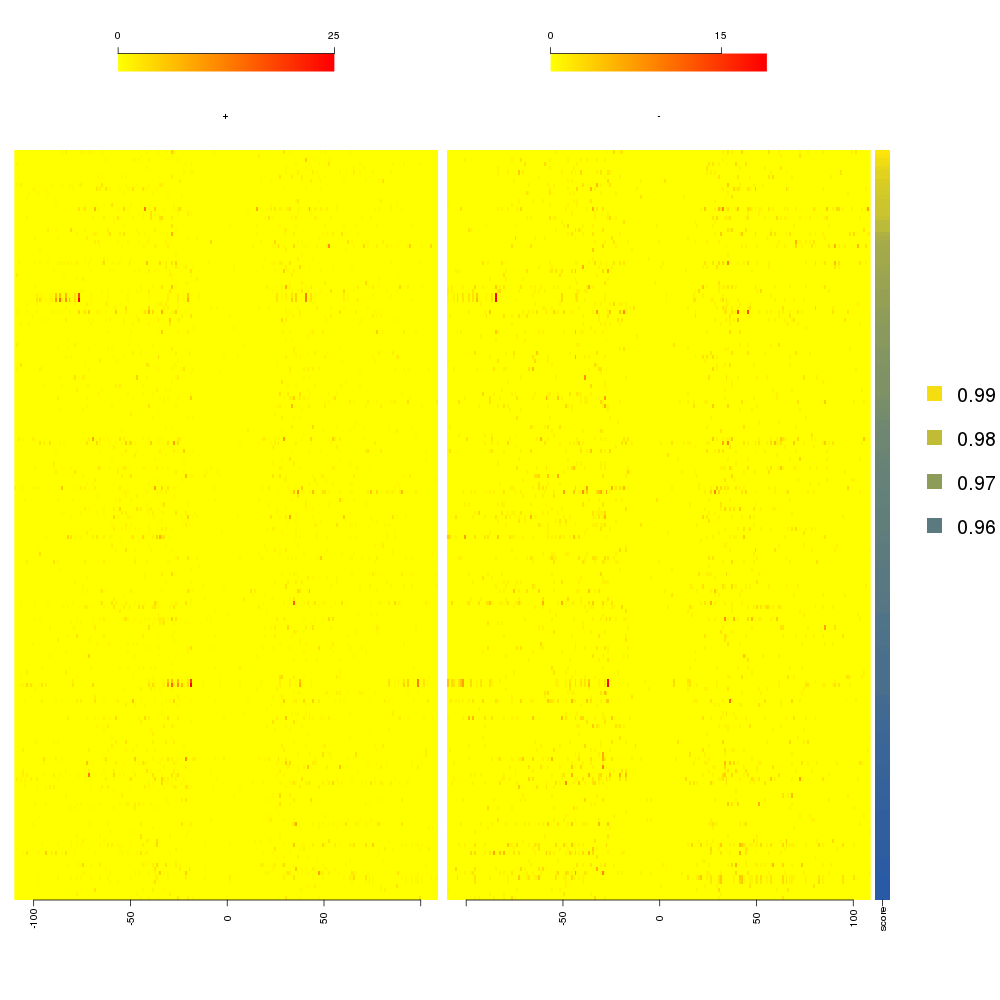

Supplement: S2 File — (ZIP) [file pone.0232332.s012.zip › nucleosome_positioning/SRR5876158_feature_aligned_heatmap.png]

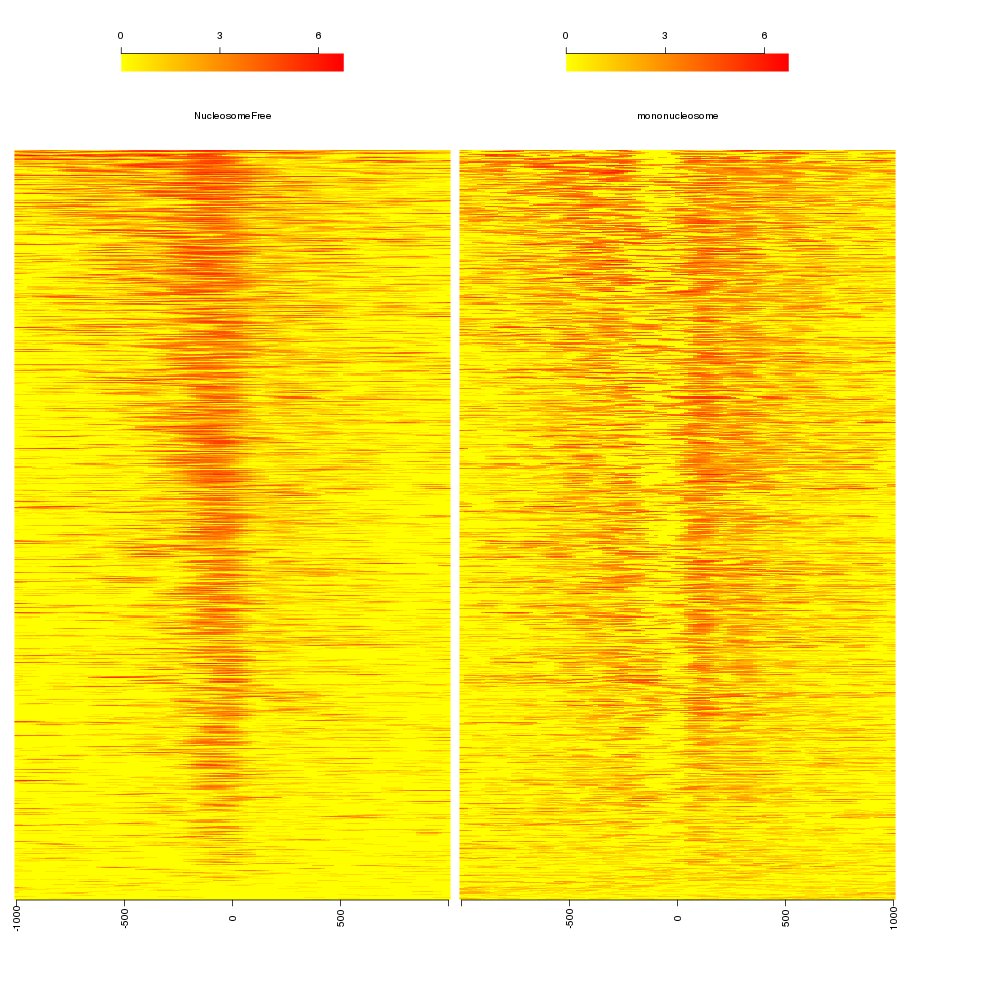

Supplement: S2 File — (ZIP) [file pone.0232332.s012.zip › nucleosome_positioning/SRR6216226_nucleosome_heatmap.png]

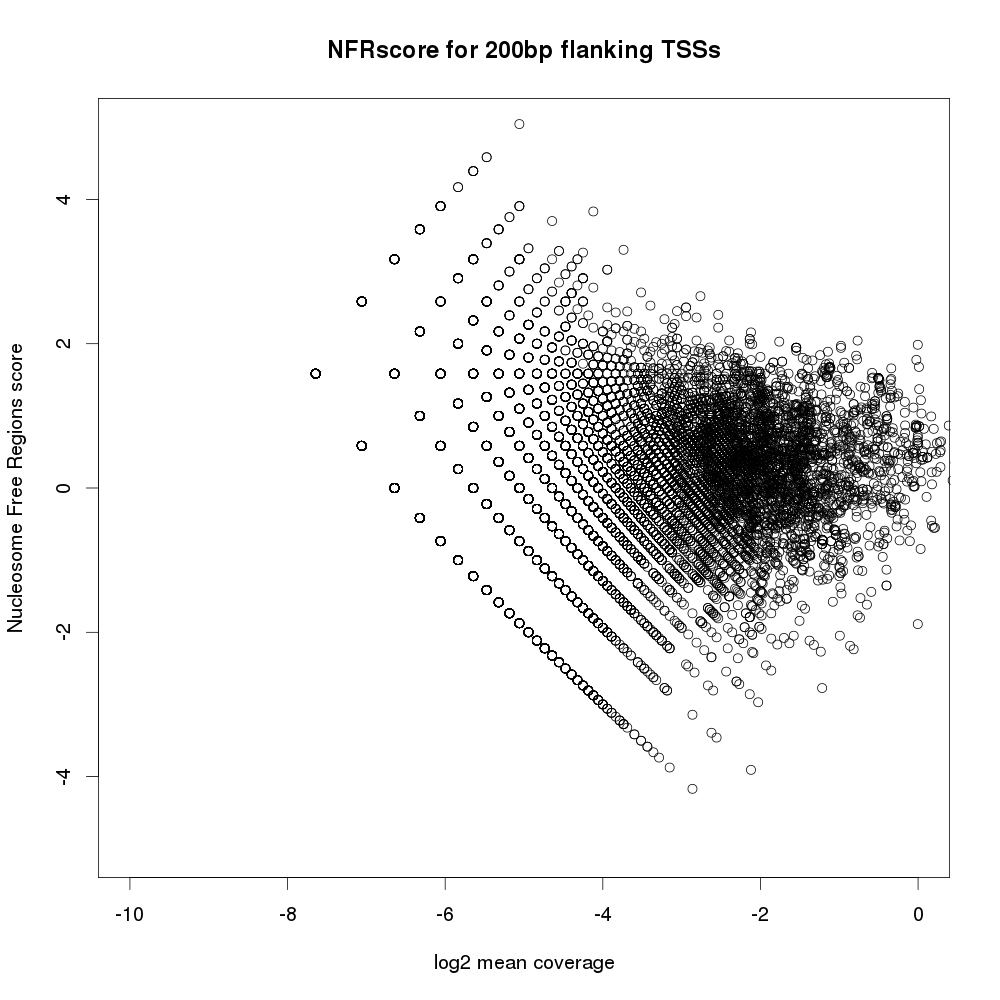

Supplement: S2 File — (ZIP) [file pone.0232332.s012.zip › nucleosome_positioning/SRR3622817_NFRscore.png]

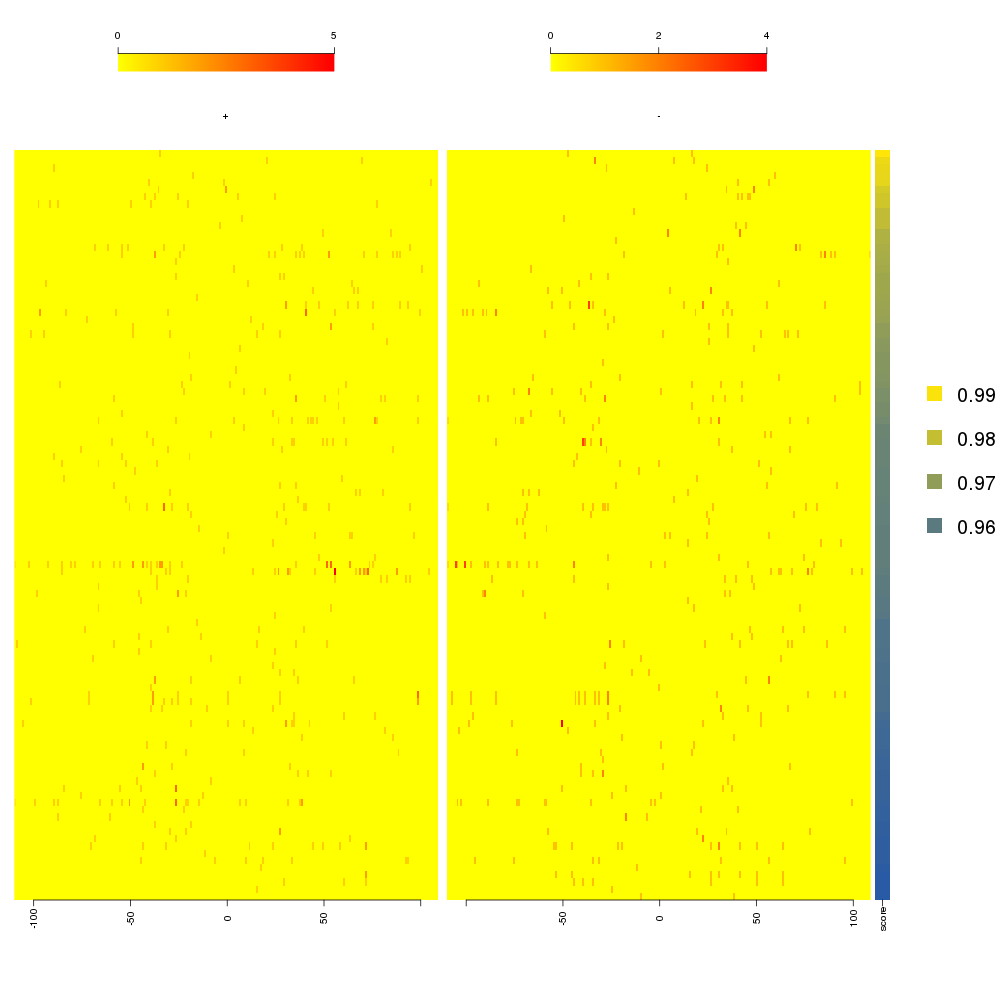

Supplement: S2 File — (ZIP) [file pone.0232332.s012.zip › nucleosome_positioning/SRR891276_feature_aligned_heatmap.png]

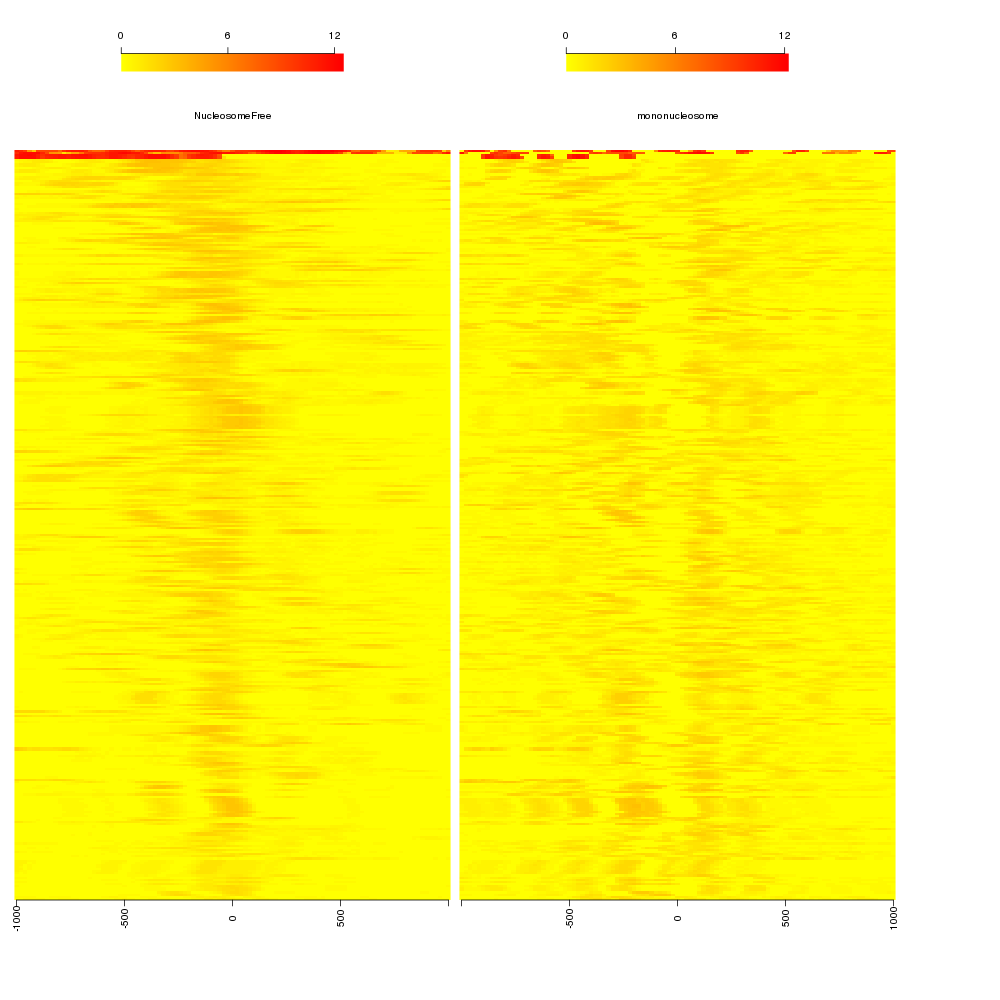

Supplement: S2 File — (ZIP) [file pone.0232332.s012.zip › nucleosome_positioning/SRR1822165_nucleosome_heatmap.png]

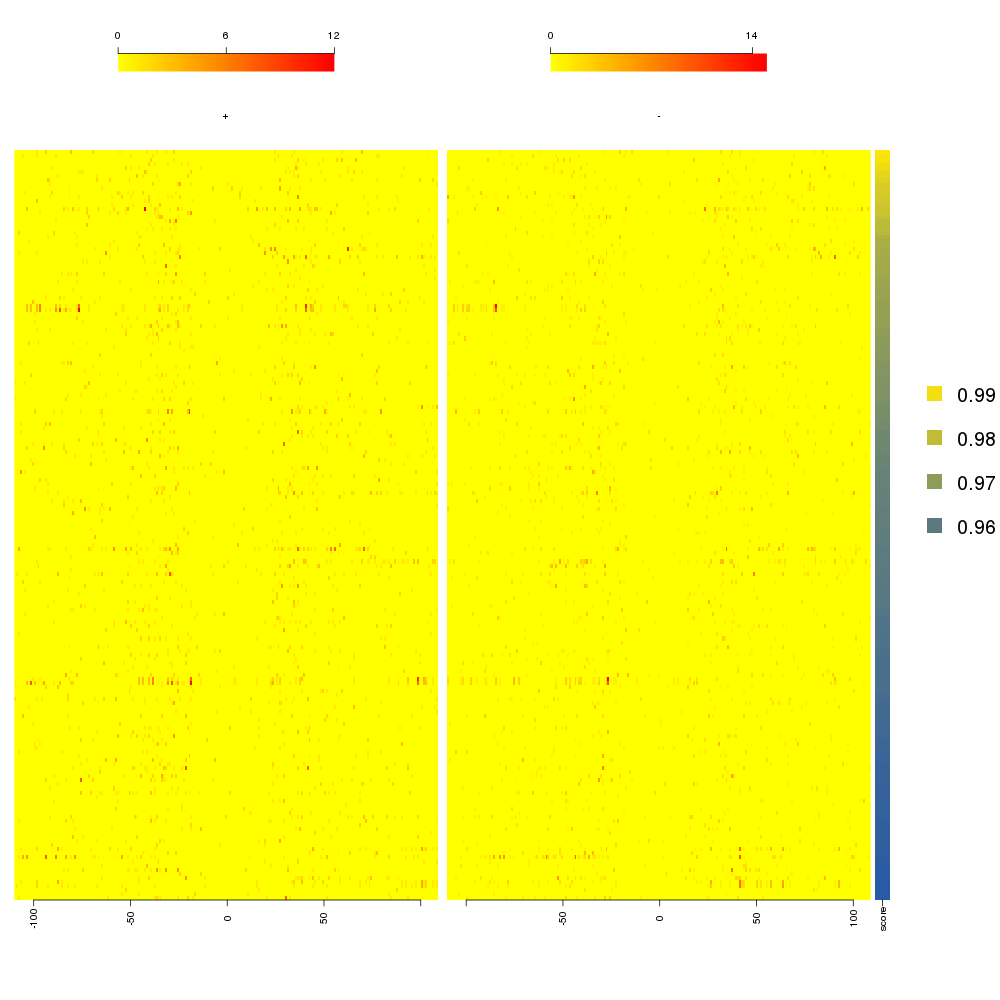

Supplement: S2 File — (ZIP) [file pone.0232332.s012.zip › nucleosome_positioning/SRR3622819_feature_aligned_heatmap.png]

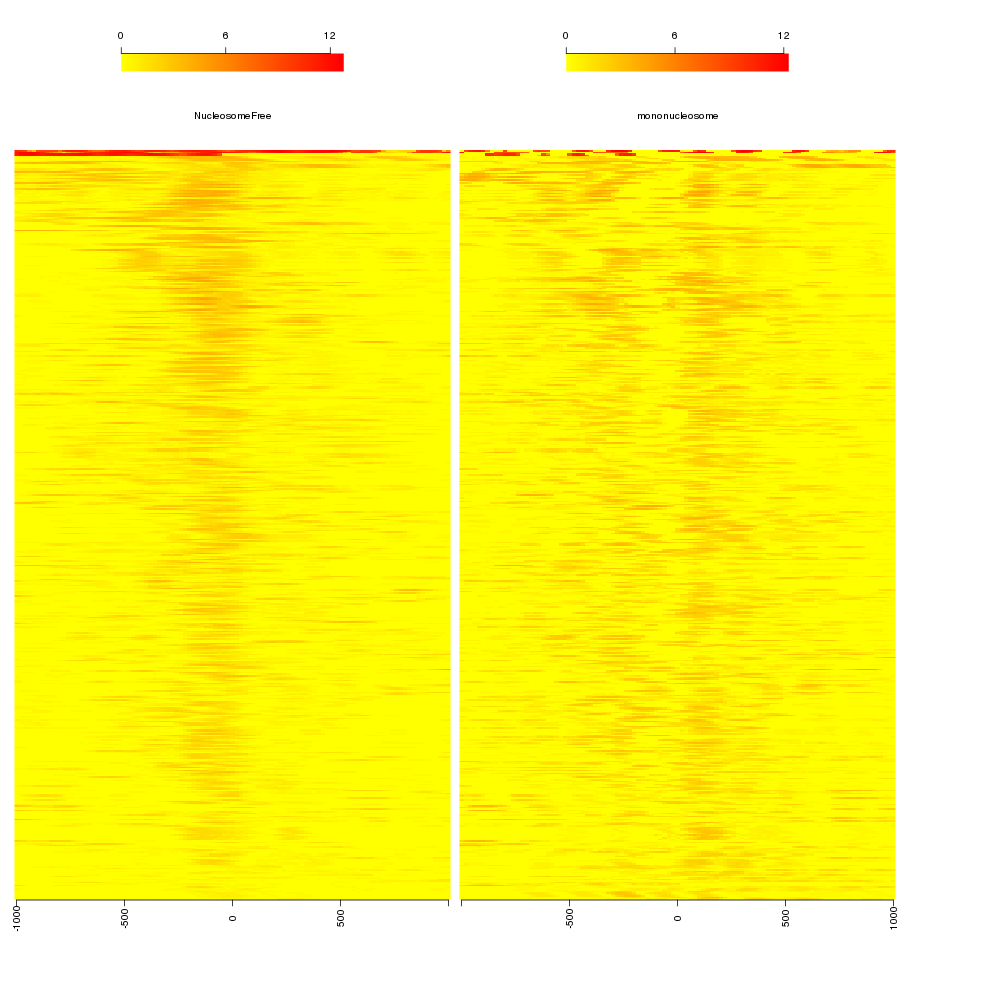

Supplement: S2 File — (ZIP) [file pone.0232332.s012.zip › nucleosome_positioning/SRR5876158_nucleosome_heatmap.png]

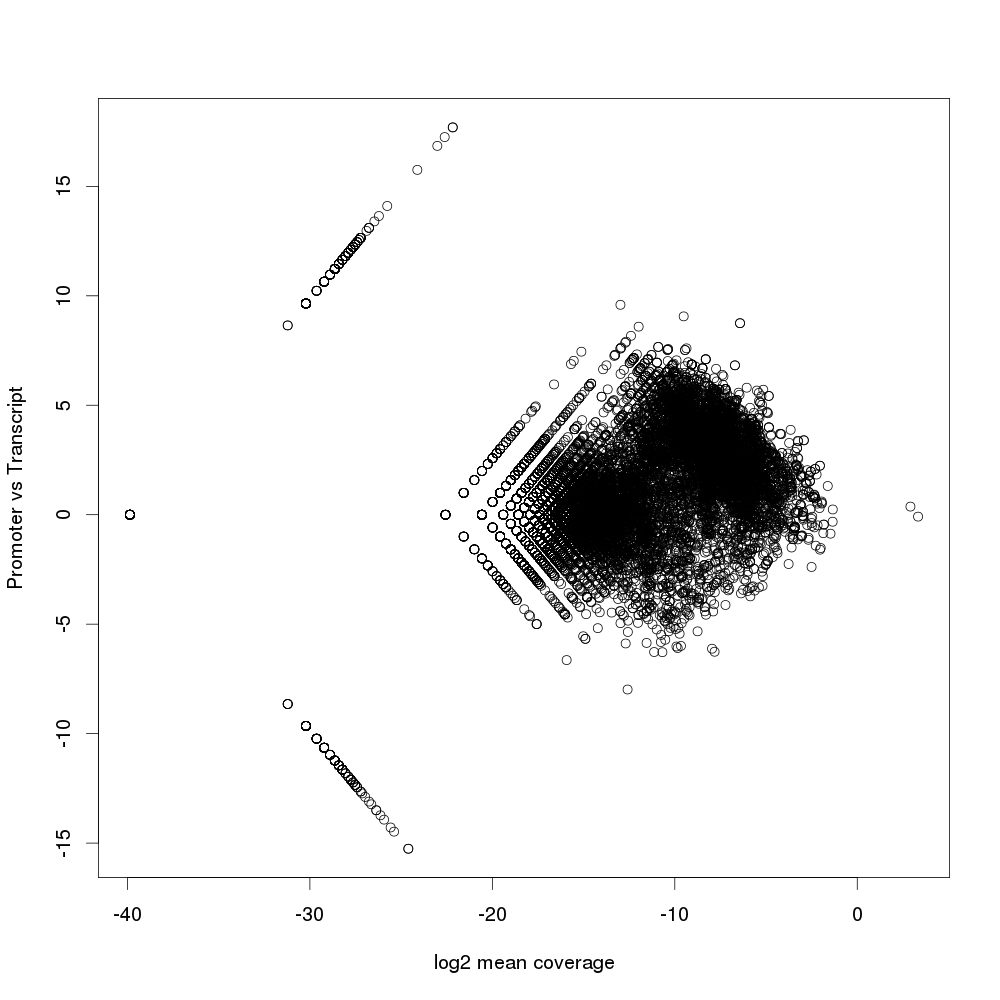

Supplement: S2 File — (ZIP) [file pone.0232332.s012.zip › nucleosome_positioning/SRR6216227_pt_score.png]

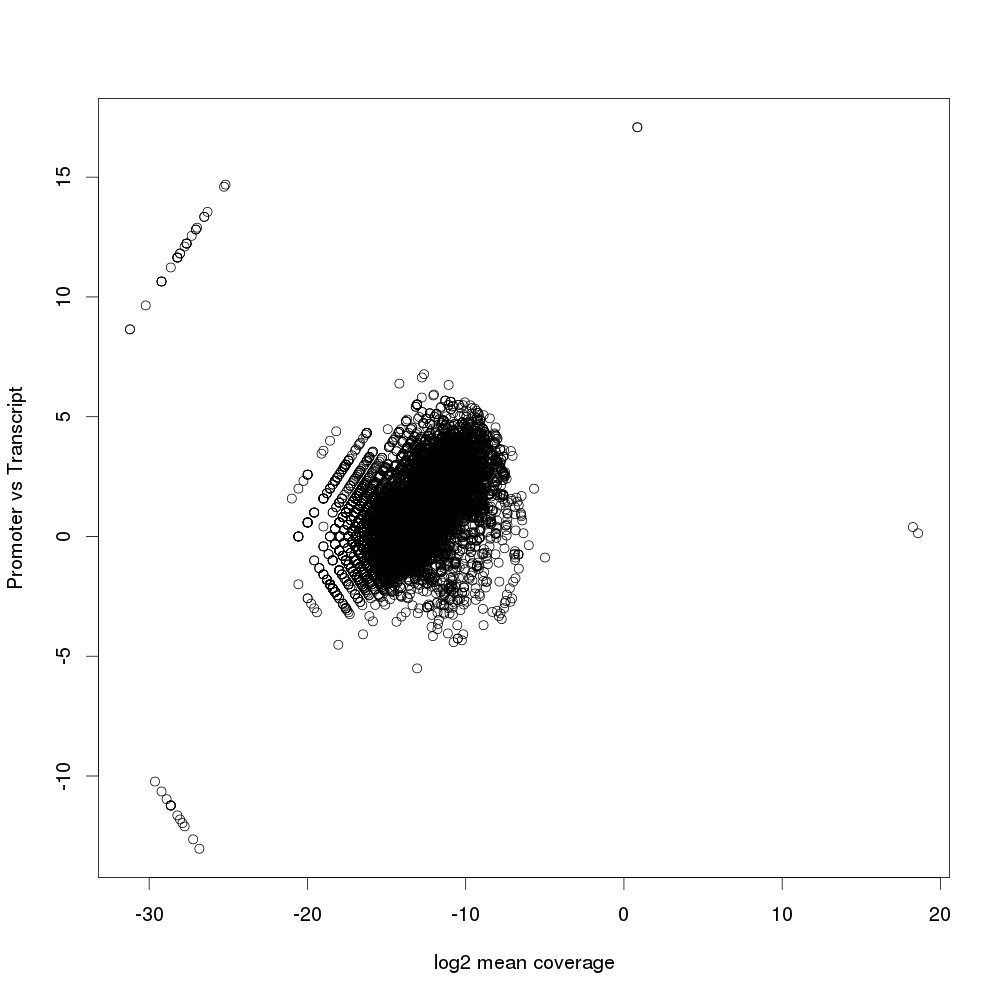

Supplement: S2 File — (ZIP) [file pone.0232332.s012.zip › nucleosome_positioning/SRR5128074_pt_score.png]

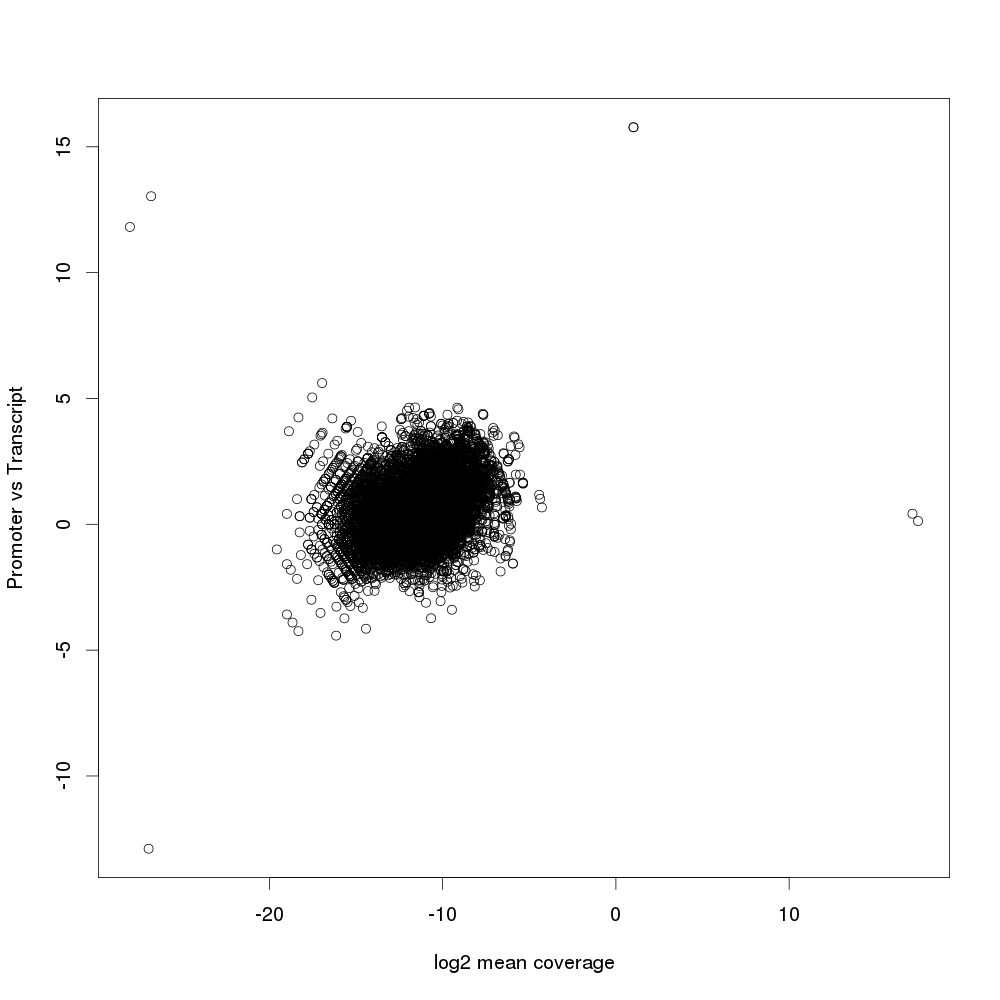

Supplement: S2 File — (ZIP) [file pone.0232332.s012.zip › nucleosome_positioning/SRR3622818_pt_score.png]

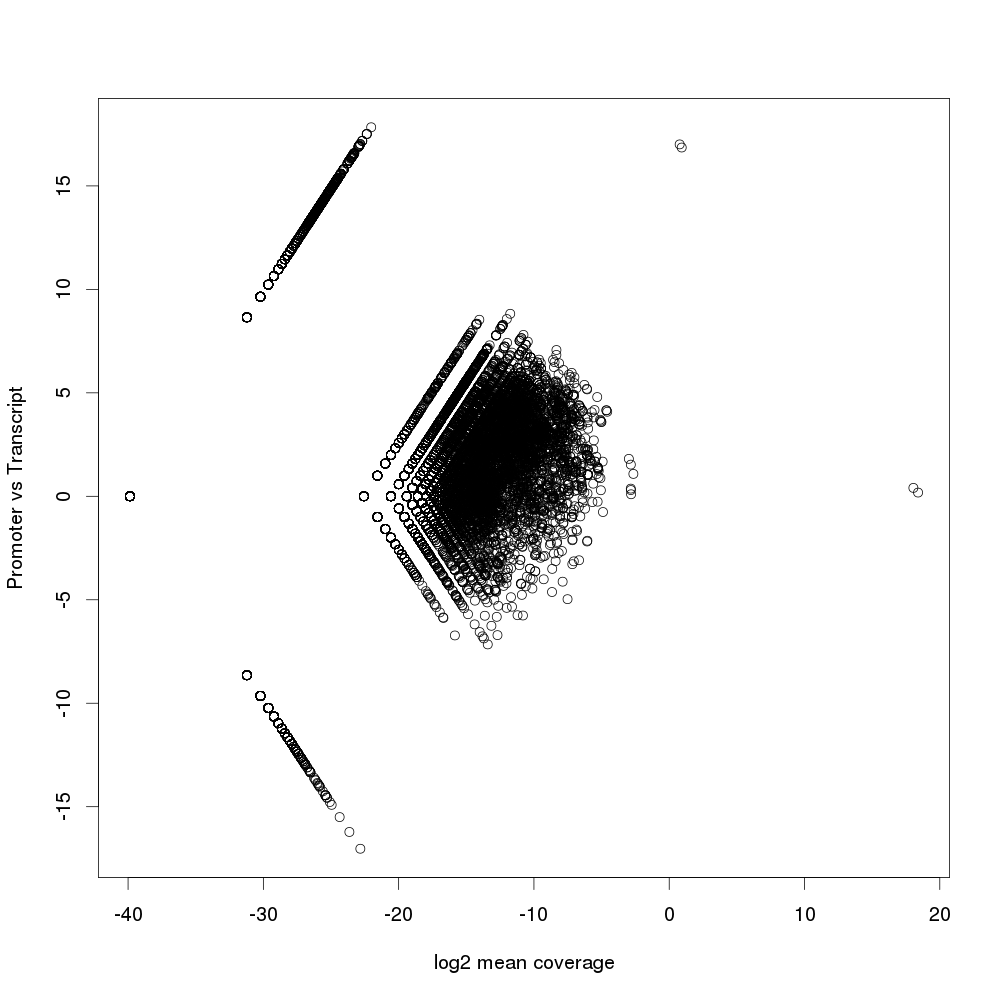

Supplement: S2 File — (ZIP) [file pone.0232332.s012.zip › nucleosome_positioning/SRR5876159_pt_score.png]

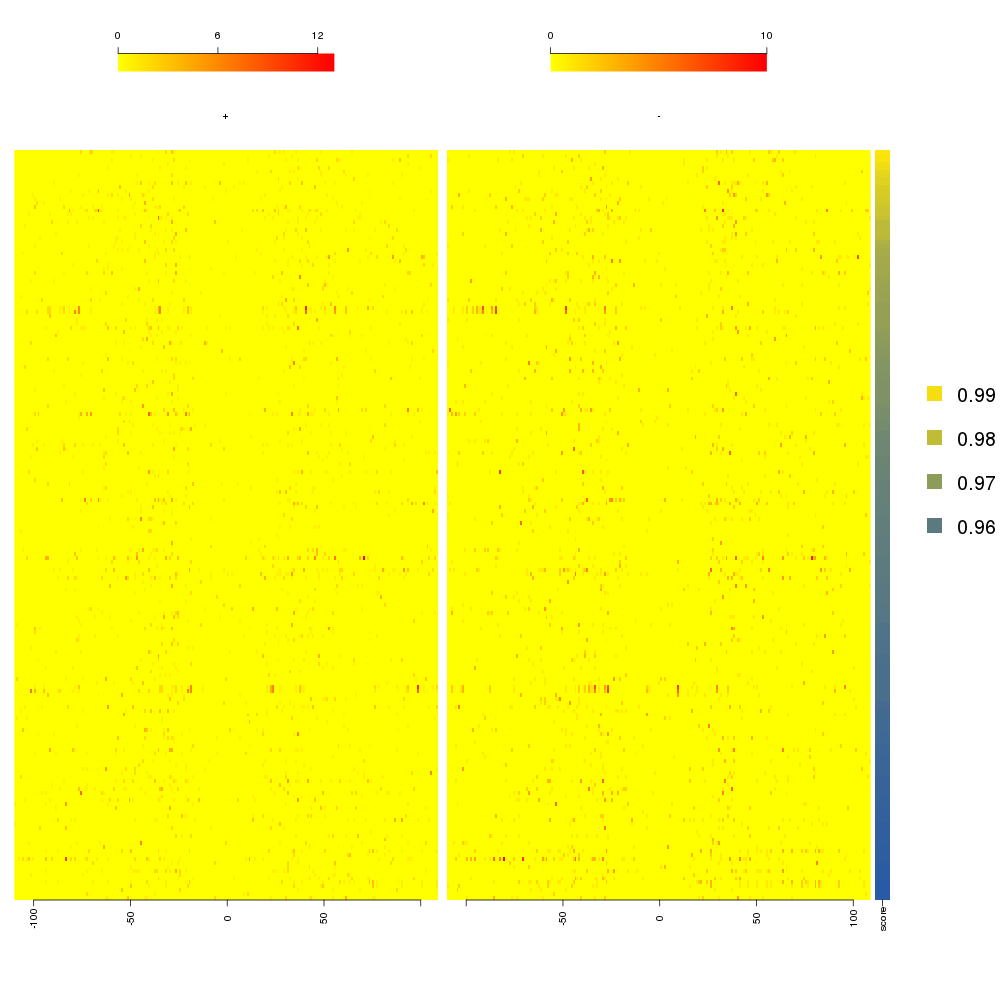

Supplement: S2 File — (ZIP) [file pone.0232332.s012.zip › nucleosome_positioning/SRR3622817_feature_aligned_heatmap.png]

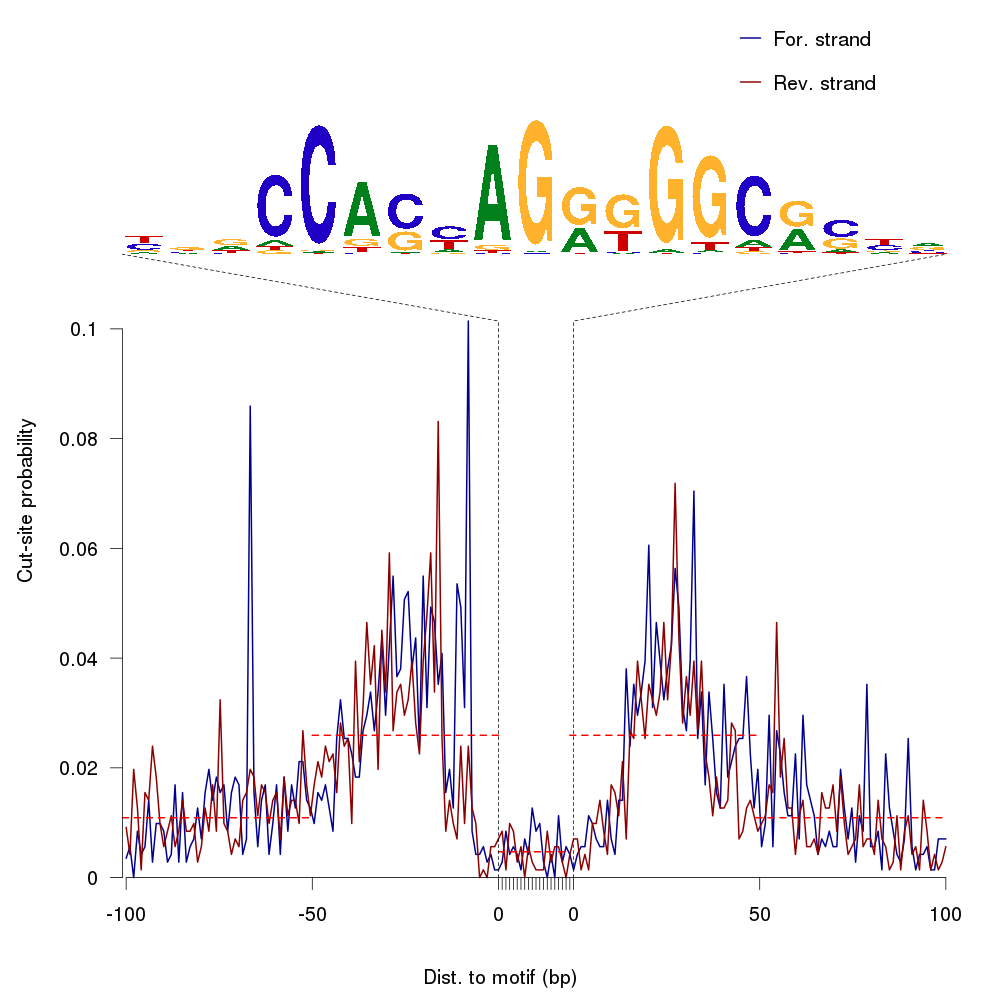

Supplement: S2 File — (ZIP) [file pone.0232332.s012.zip › nucleosome_positioning/SRR1822168_footprint_plot.png]

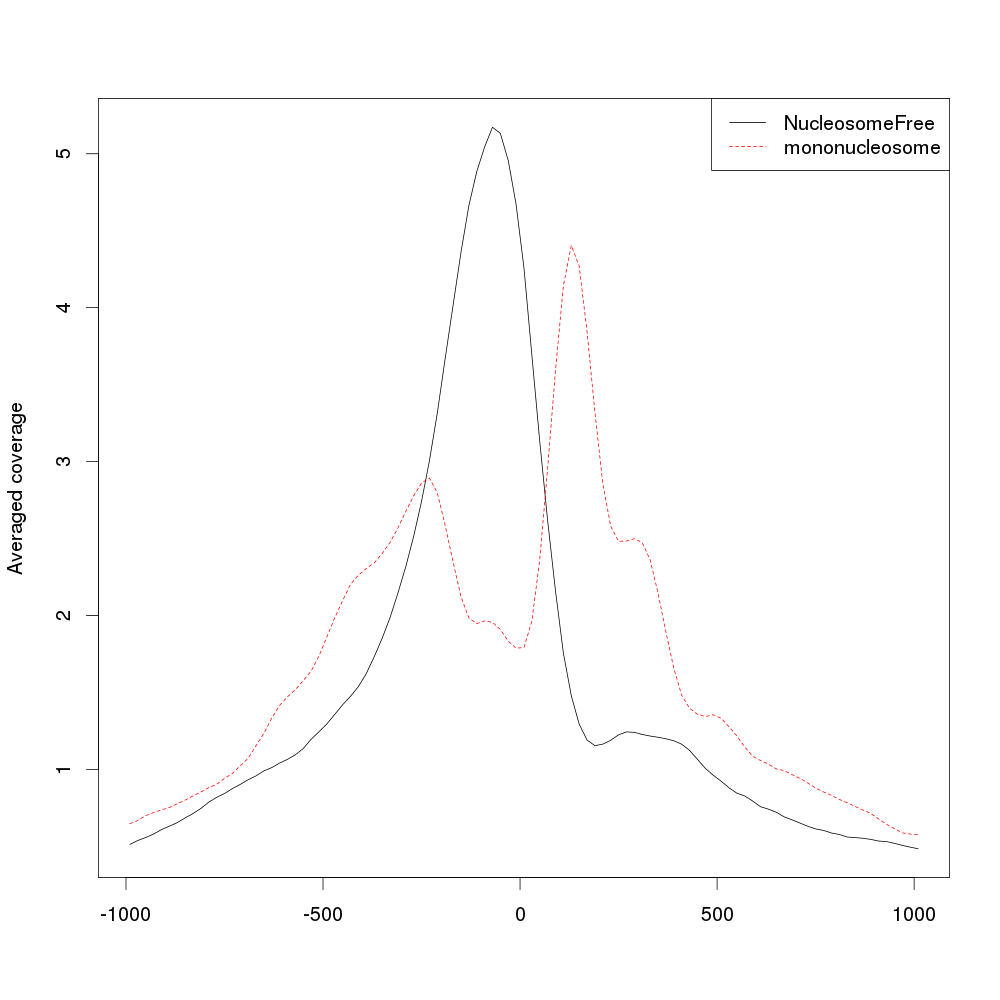

Supplement: S2 File — (ZIP) [file pone.0232332.s012.zip › nucleosome_positioning/SRX6443490_nucleosome_distribution.png]

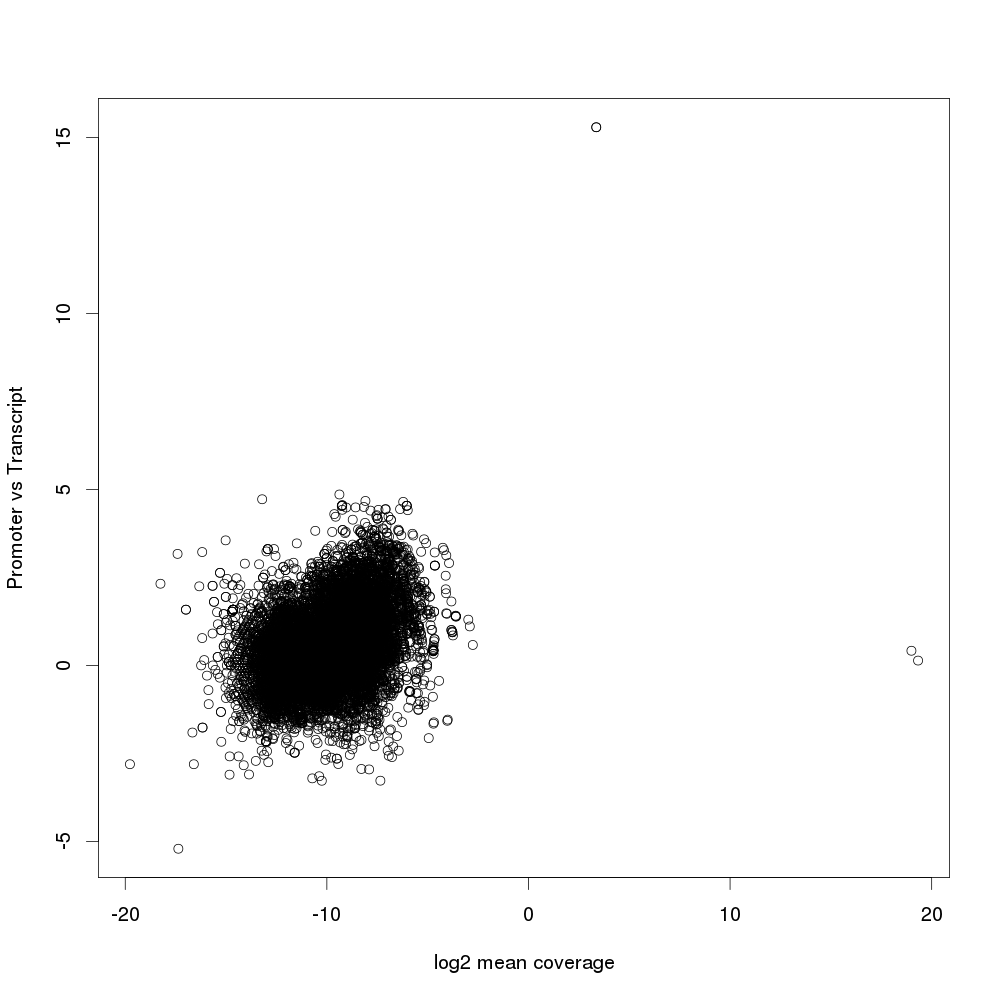

Supplement: S2 File — (ZIP) [file pone.0232332.s012.zip › nucleosome_positioning/SRR3622819_pt_score.png]

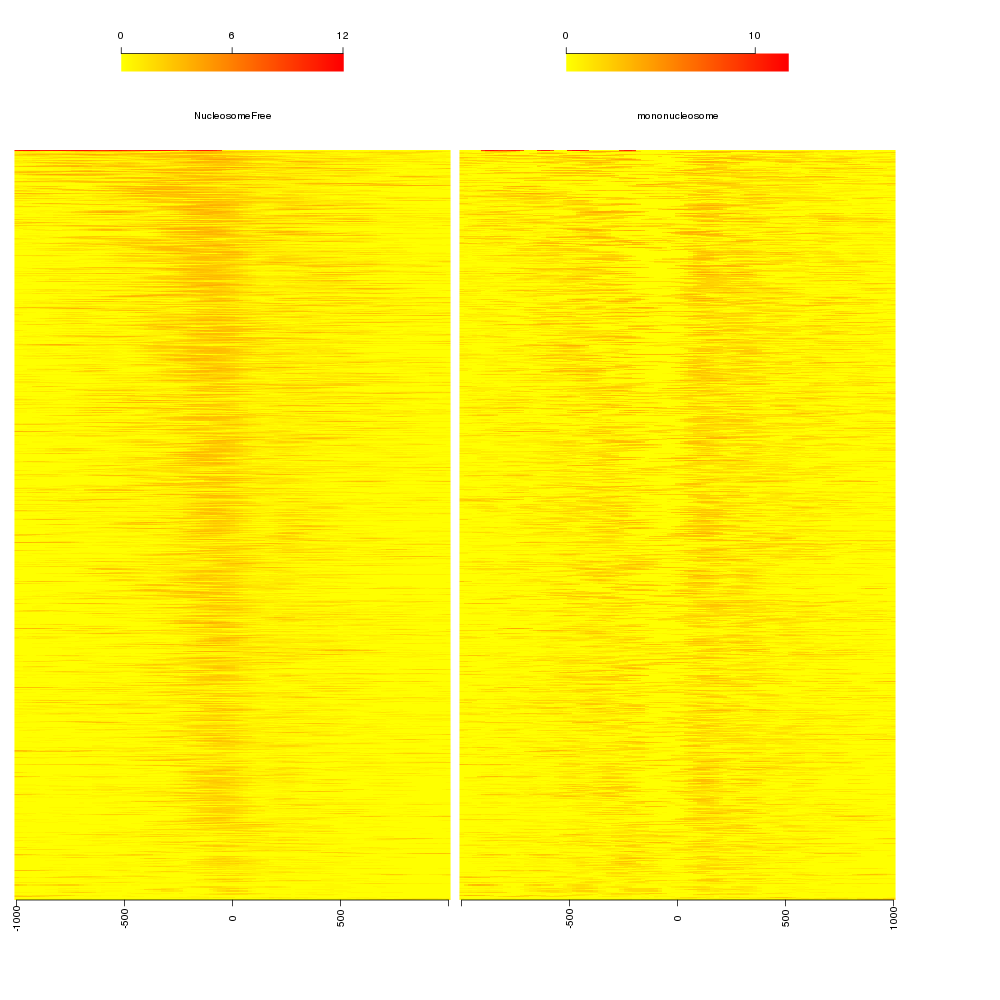

Supplement: S2 File — (ZIP) [file pone.0232332.s012.zip › nucleosome_positioning/SRR5007258_nucleosome_heatmap.png]

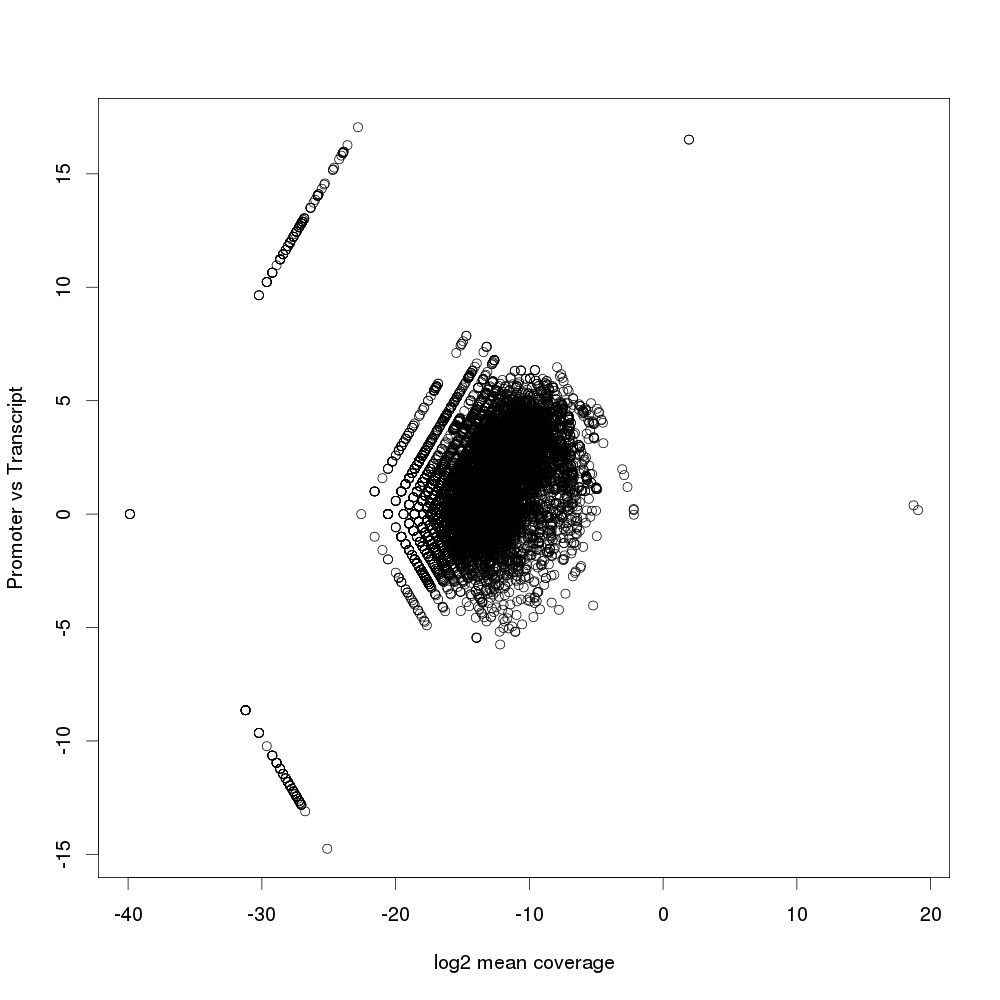

Supplement: S2 File — (ZIP) [file pone.0232332.s012.zip › nucleosome_positioning/SRR5876158_pt_score.png]

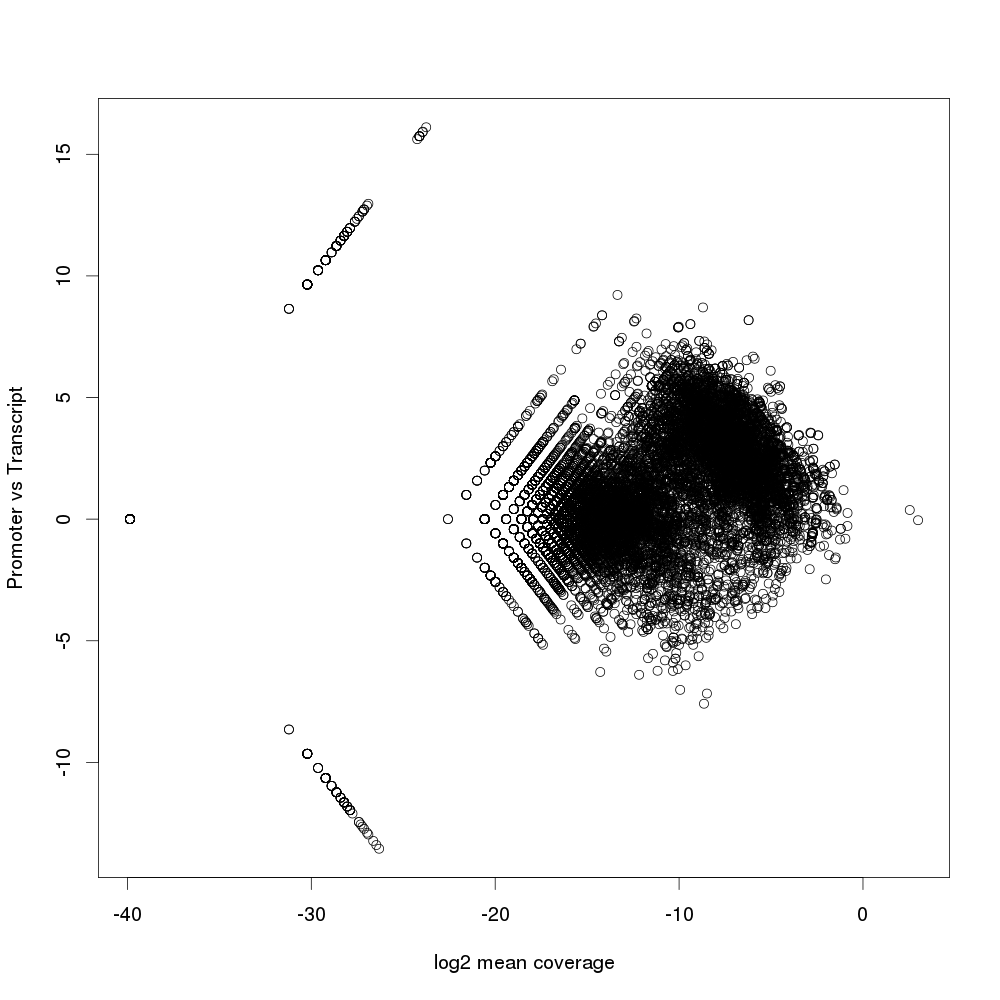

Supplement: S2 File — (ZIP) [file pone.0232332.s012.zip › nucleosome_positioning/SRR6216226_pt_score.png]

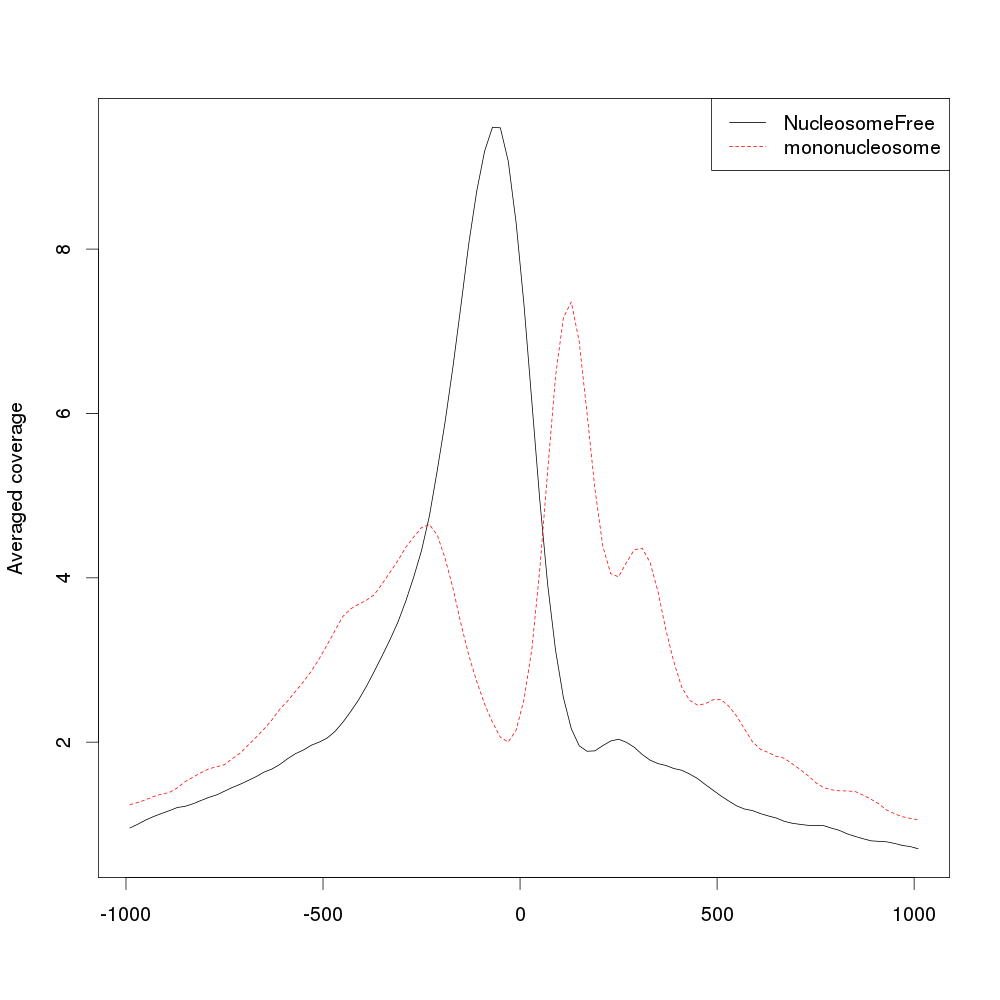

Supplement: S2 File — (ZIP) [file pone.0232332.s012.zip › nucleosome_positioning/SRR6216226_nucleosome_distribution.png]

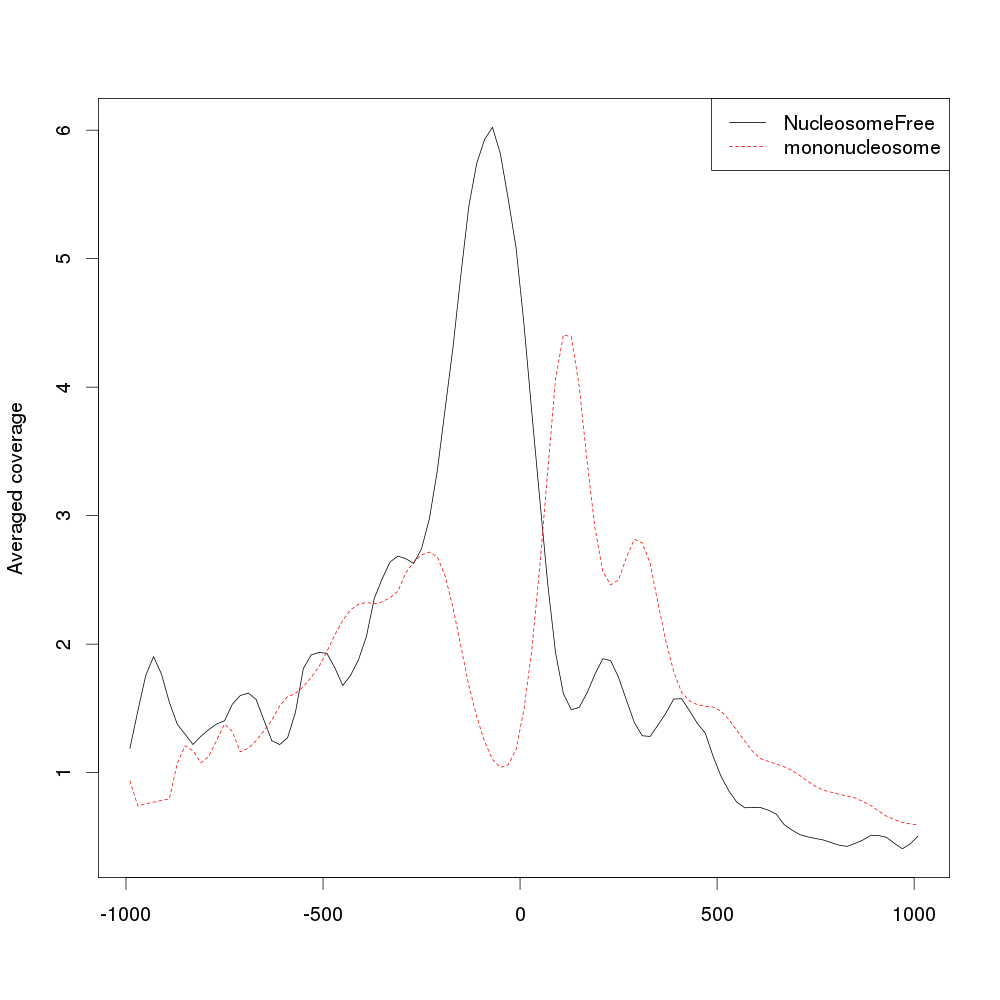

Supplement: S2 File — (ZIP) [file pone.0232332.s012.zip › nucleosome_positioning/SRR5063984_nucleosome_distribution.png]

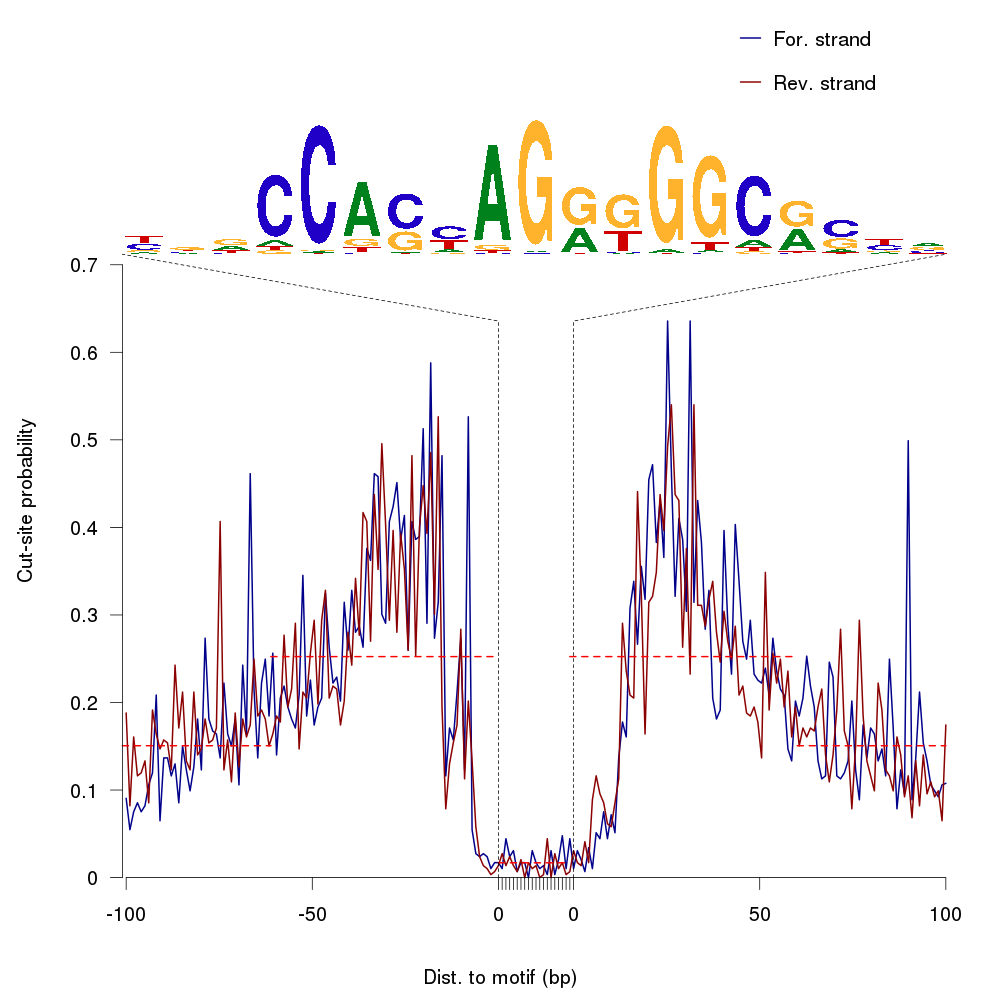

Supplement: S2 File — (ZIP) [file pone.0232332.s012.zip › nucleosome_positioning/SRR5007258_footprint_plot.png]

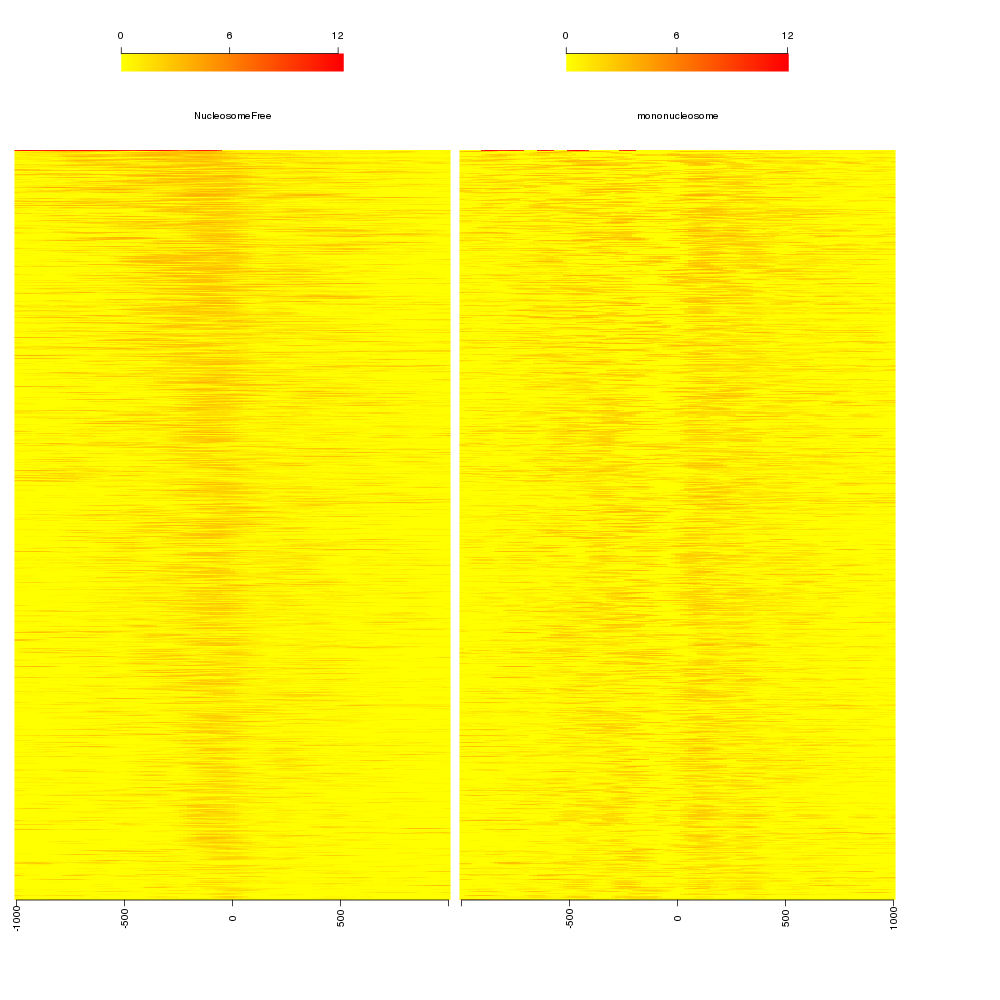

Supplement: S2 File — (ZIP) [file pone.0232332.s012.zip › nucleosome_positioning/SRR5007259_nucleosome_heatmap.png]

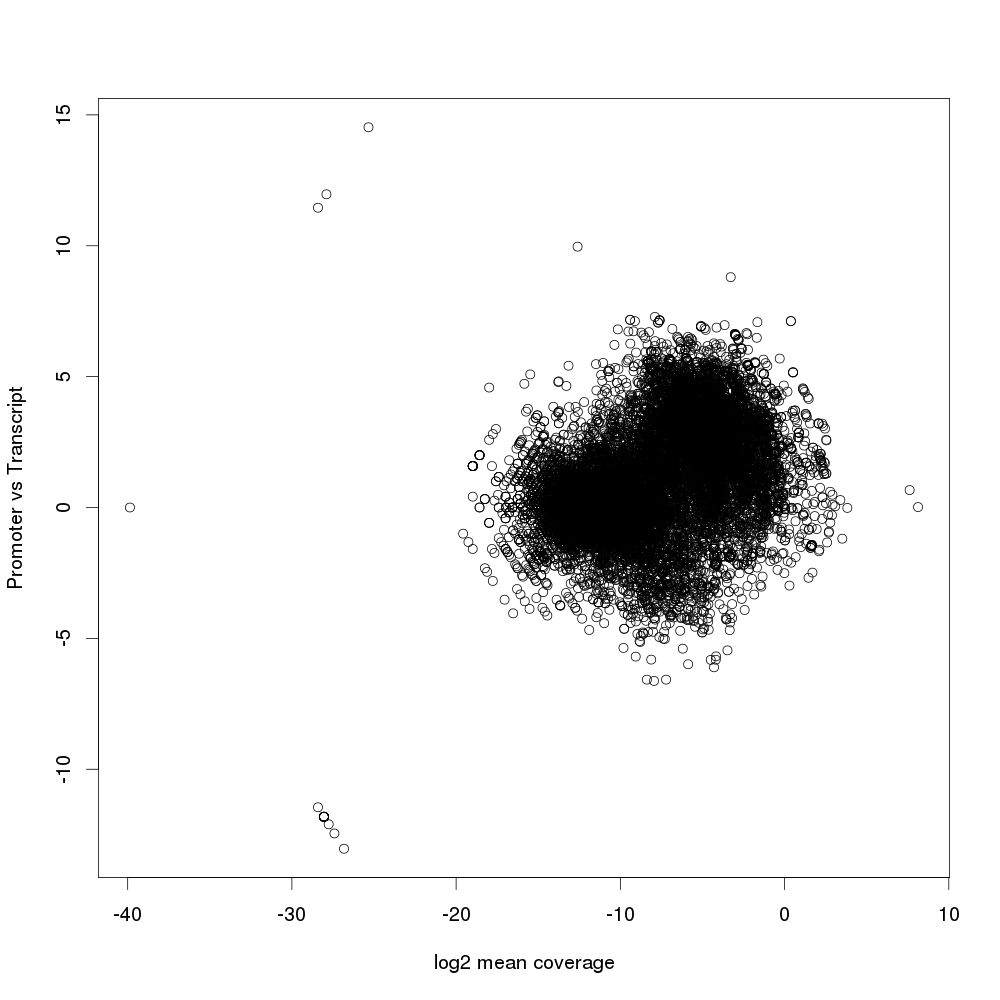

Supplement: S2 File — (ZIP) [file pone.0232332.s012.zip › nucleosome_positioning/SRX6443488_pt_score.png]

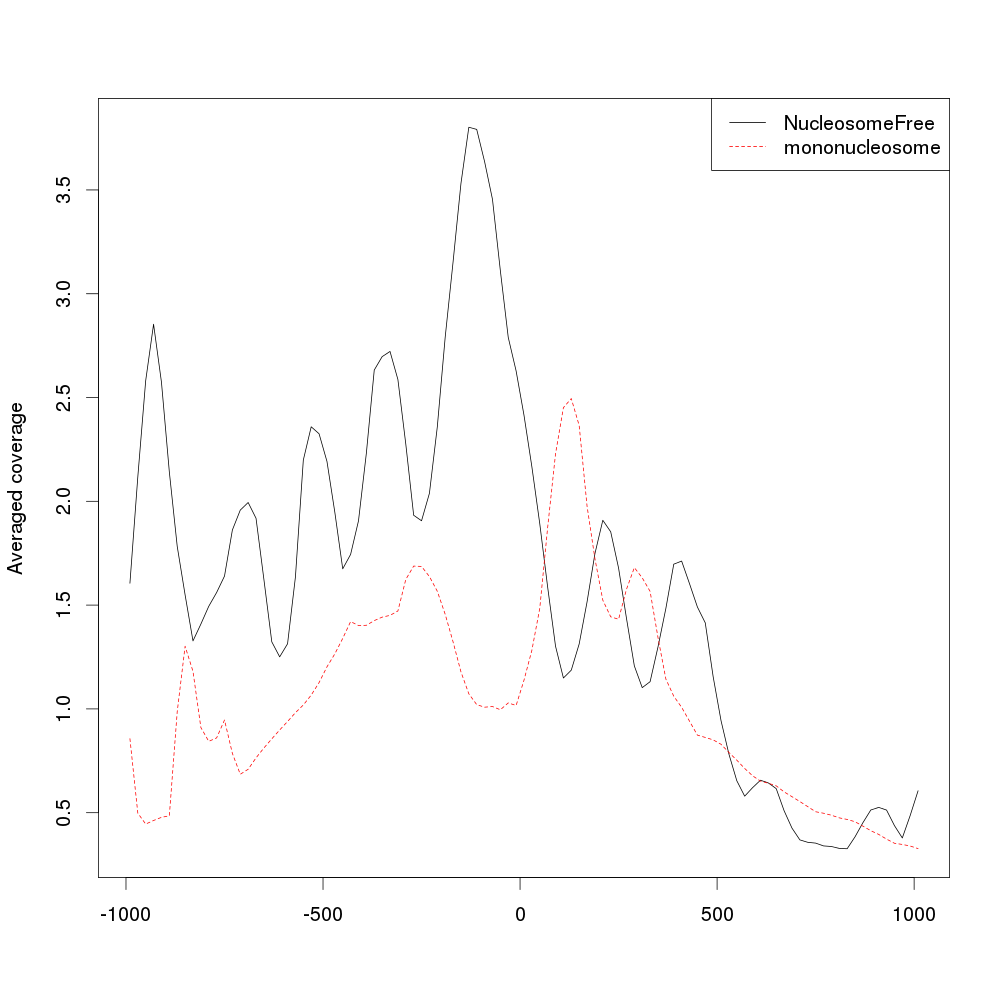

Supplement: S2 File — (ZIP) [file pone.0232332.s012.zip › nucleosome_positioning/SRR5063986_nucleosome_distribution.png]

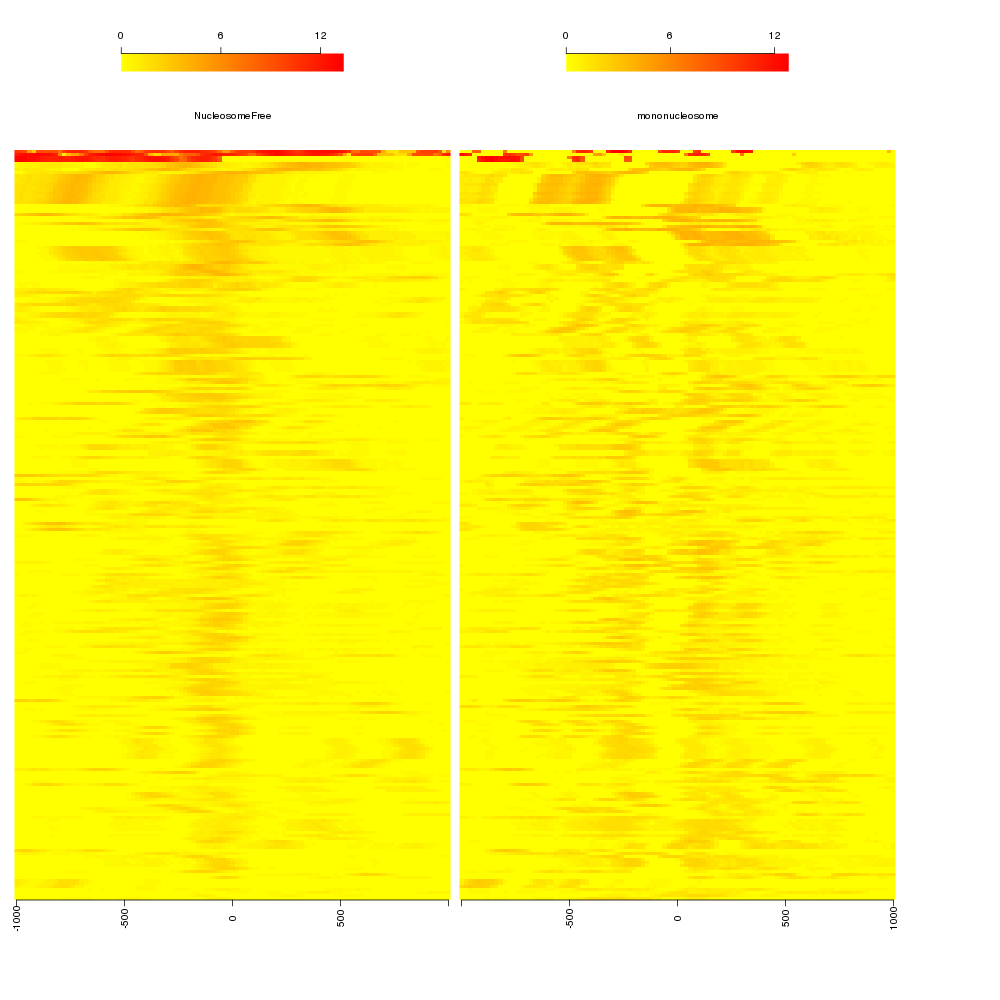

Supplement: S2 File — (ZIP) [file pone.0232332.s012.zip › nucleosome_positioning/SRR8932927_nucleosome_heatmap.png]

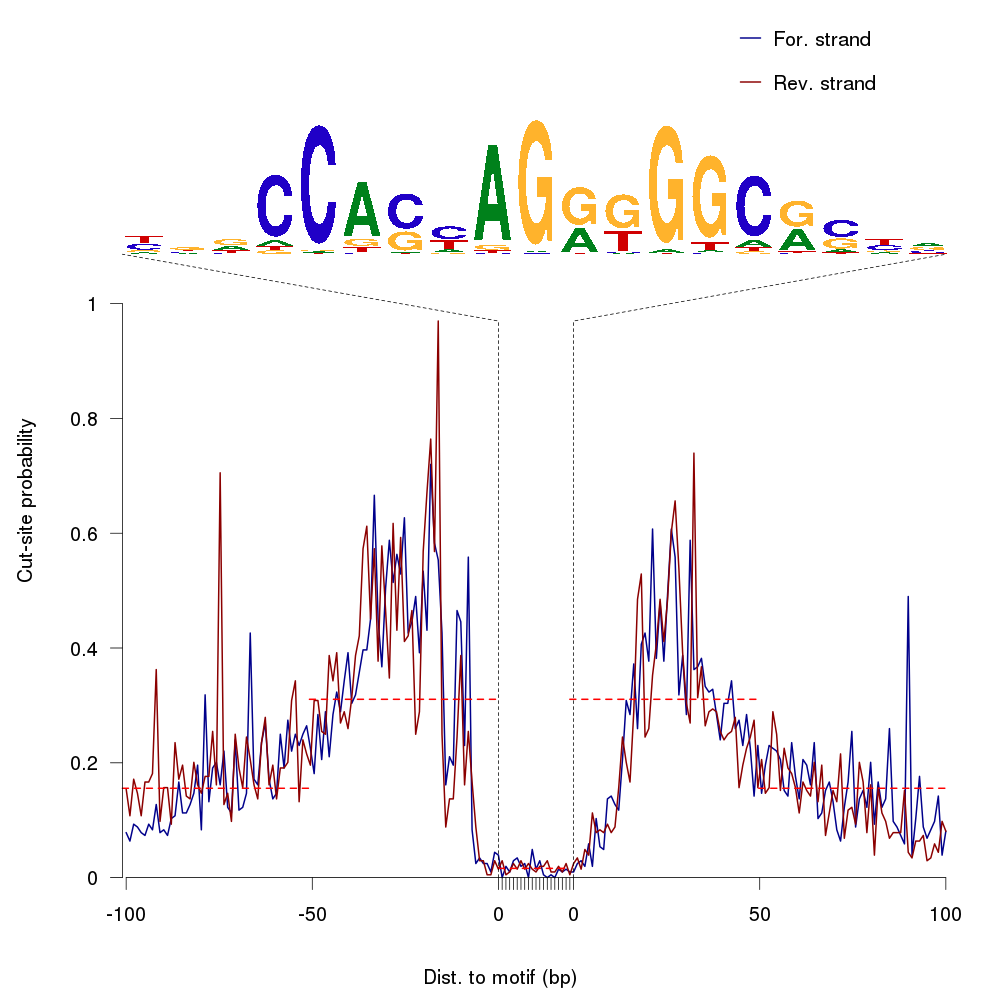

Supplement: S2 File — (ZIP) [file pone.0232332.s012.zip › nucleosome_positioning/SRR5063986_footprint_plot.png]

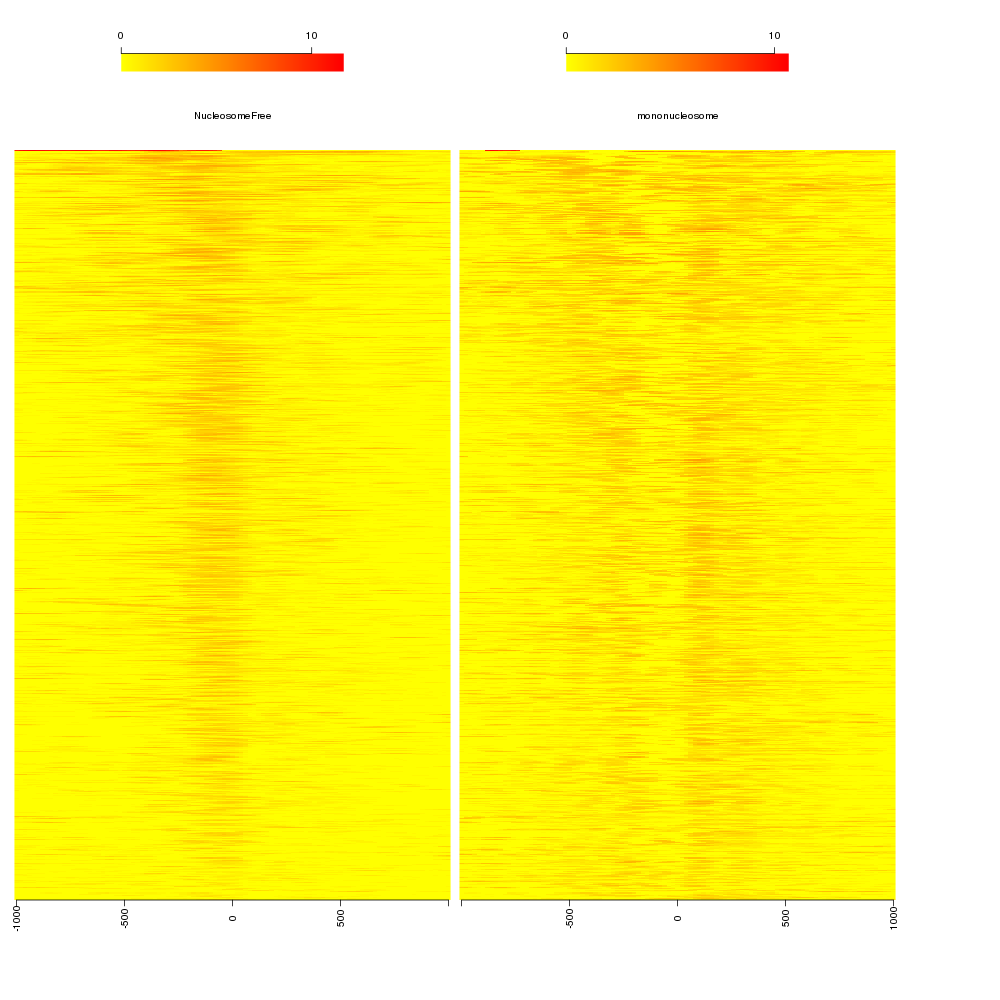

Supplement: S2 File — (ZIP) [file pone.0232332.s012.zip › nucleosome_positioning/SRR5063986_nucleosome_heatmap.png]

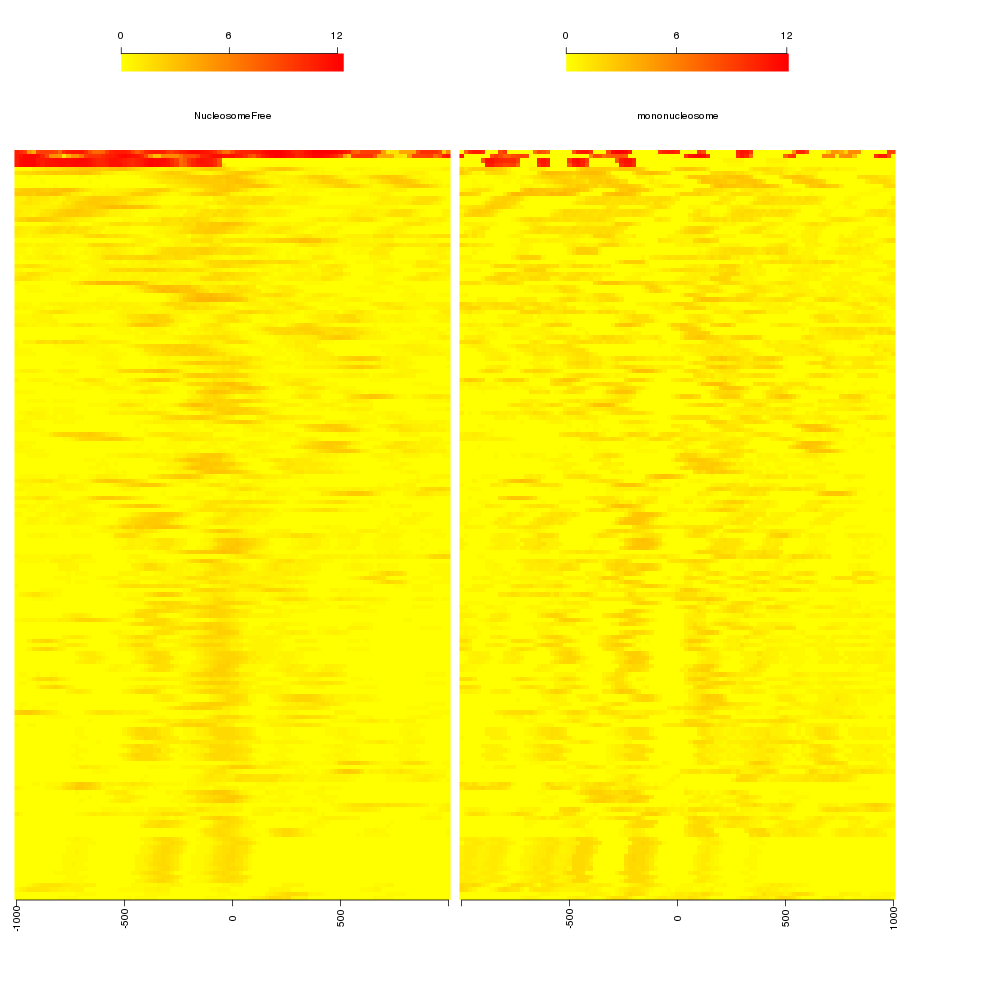

Supplement: S2 File — (ZIP) [file pone.0232332.s012.zip › nucleosome_positioning/SRR3622817_nucleosome_heatmap.png]

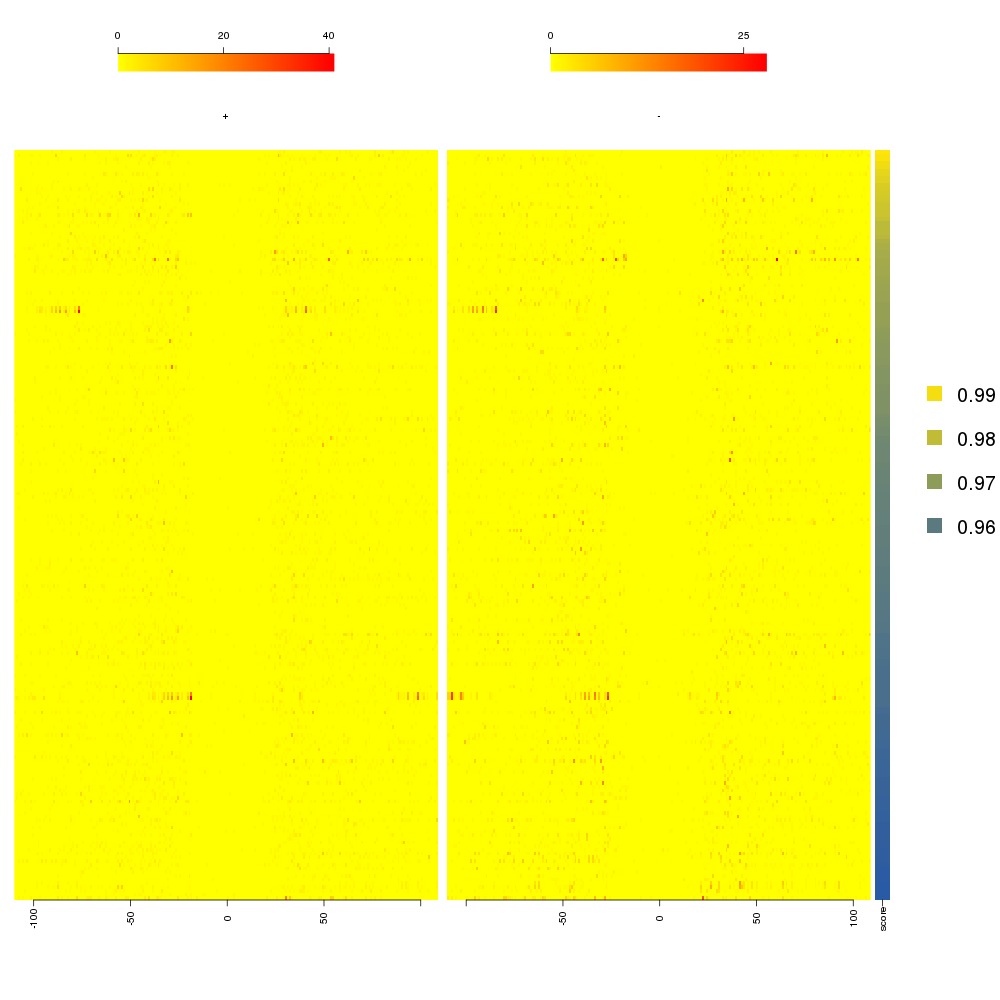

Supplement: S2 File — (ZIP) [file pone.0232332.s012.zip › nucleosome_positioning/SRR5007259_feature_aligned_heatmap.png]

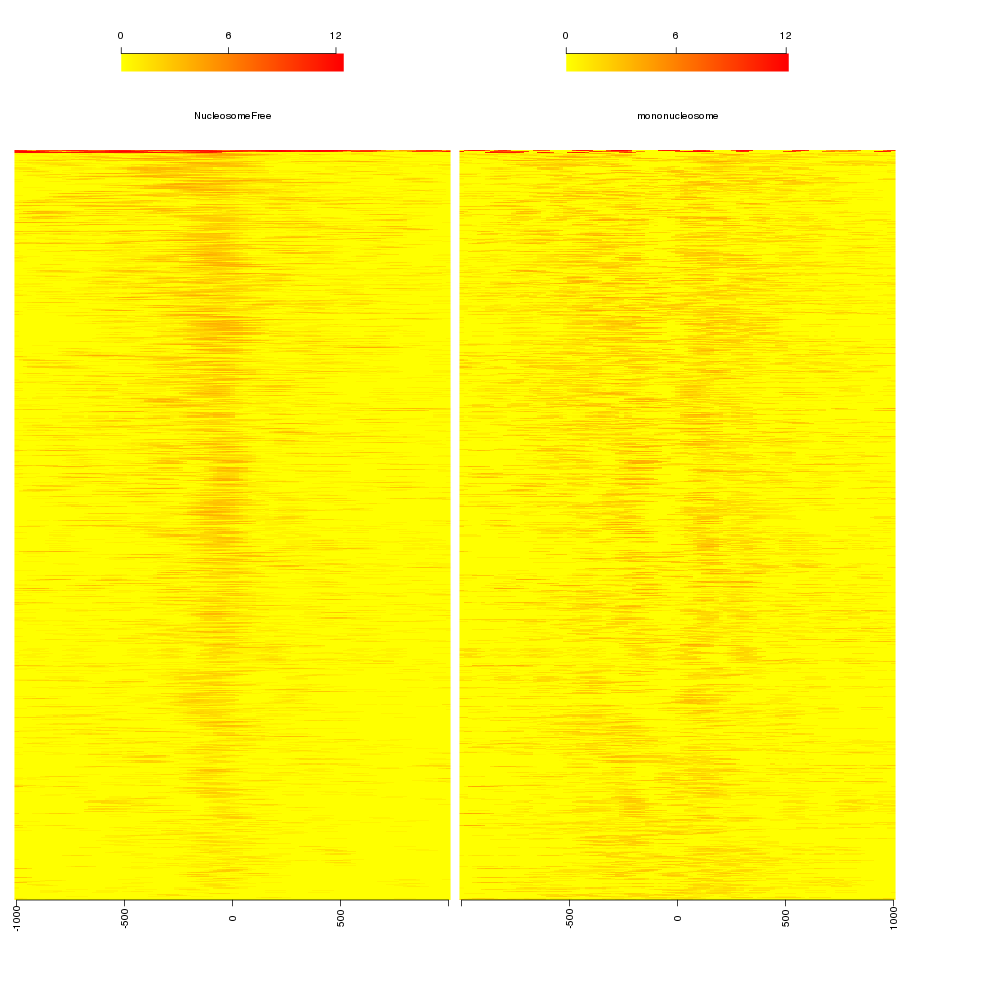

Supplement: S2 File — (ZIP) [file pone.0232332.s012.zip › nucleosome_positioning/SRR891276_nucleosome_heatmap.png]

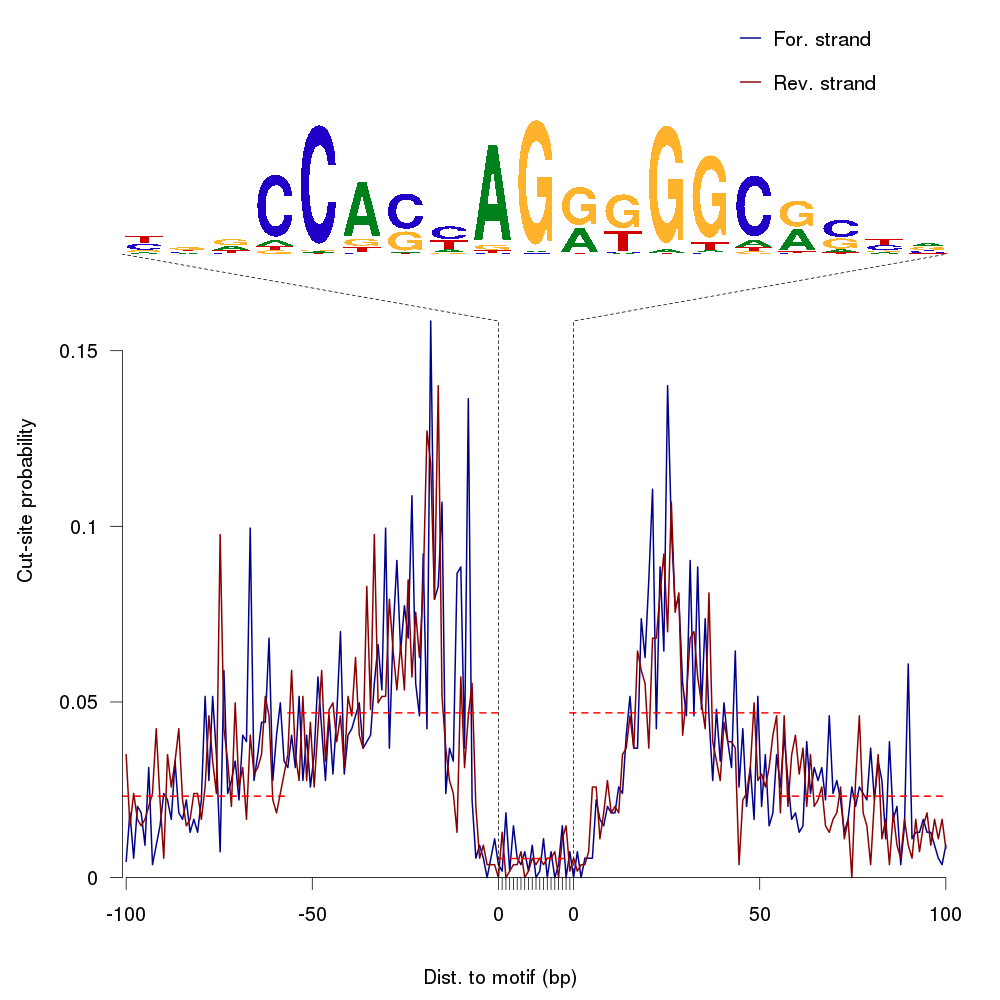

Supplement: S2 File — (ZIP) [file pone.0232332.s012.zip › nucleosome_positioning/SRR5876158_footprint_plot.png]

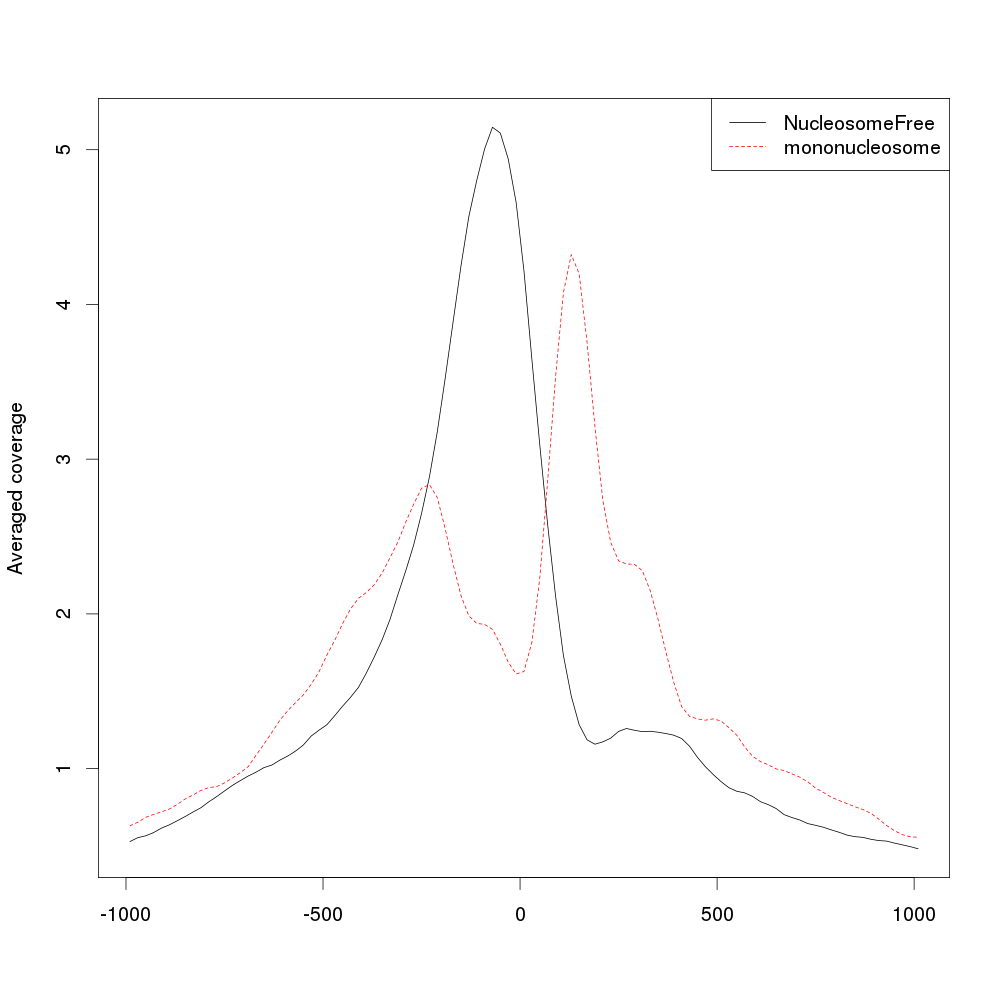

Supplement: S2 File — (ZIP) [file pone.0232332.s012.zip › nucleosome_positioning/SRX6443488_nucleosome_distribution.png]

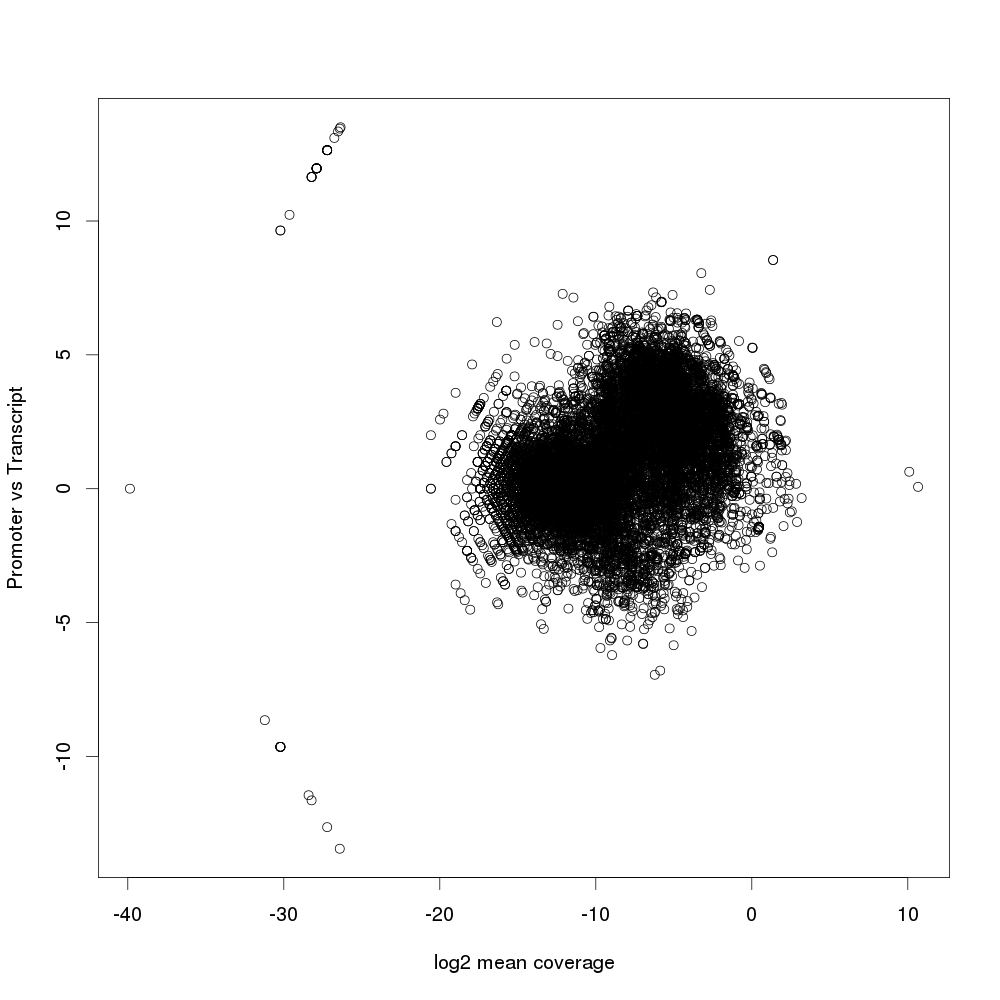

Supplement: S2 File — (ZIP) [file pone.0232332.s012.zip › nucleosome_positioning/SRX6443489_pt_score.png]
