# Supplementary figures and images for: Combining signal and sequence to detect RNA polymerase initiation in ATAC-seq data (part 1 of 2)
Source: PLoS One. 2020 Apr 30;15(4):e0232332. doi: 10.1371/journal.pone.0232332 (PMC7192442; doi:10.1371/journal.pone.0232332)

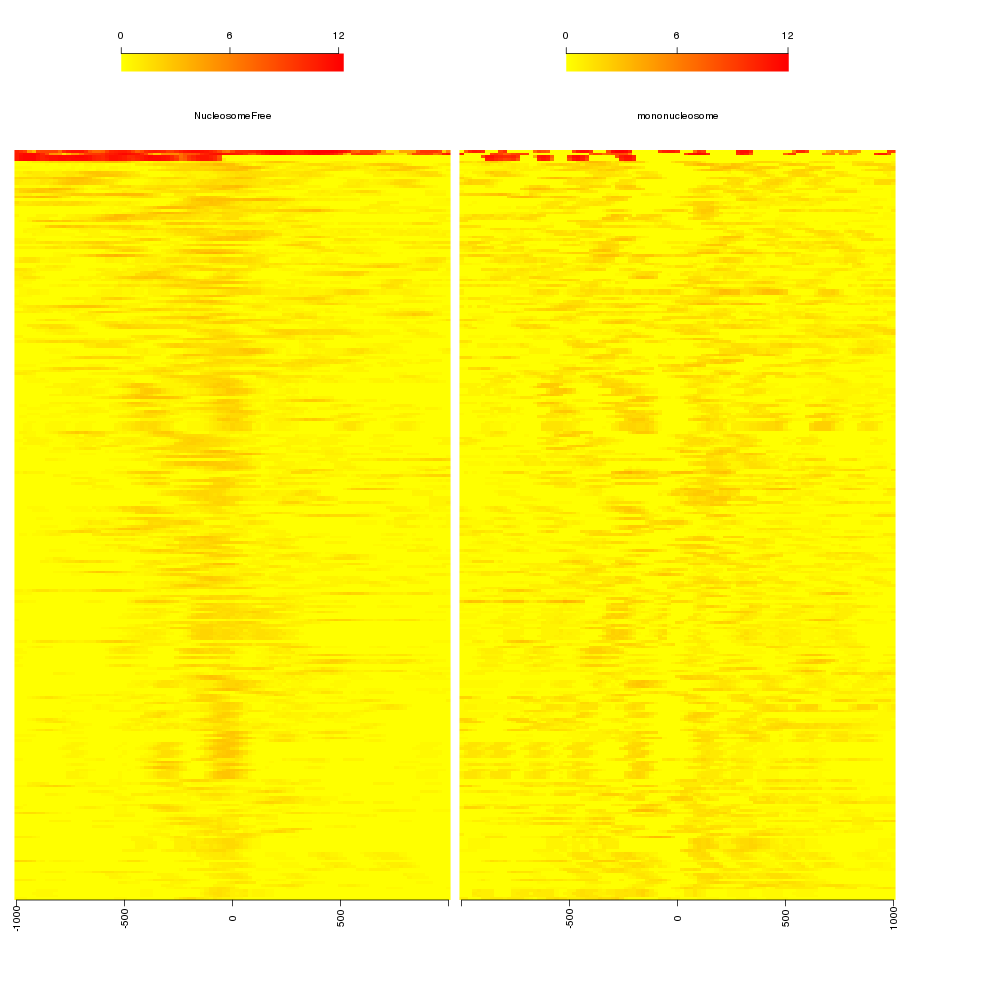

Supplement: S2 File — (ZIP) [file pone.0232332.s012.zip › nucleosome_positioning/SRR3622818_nucleosome_heatmap.png]

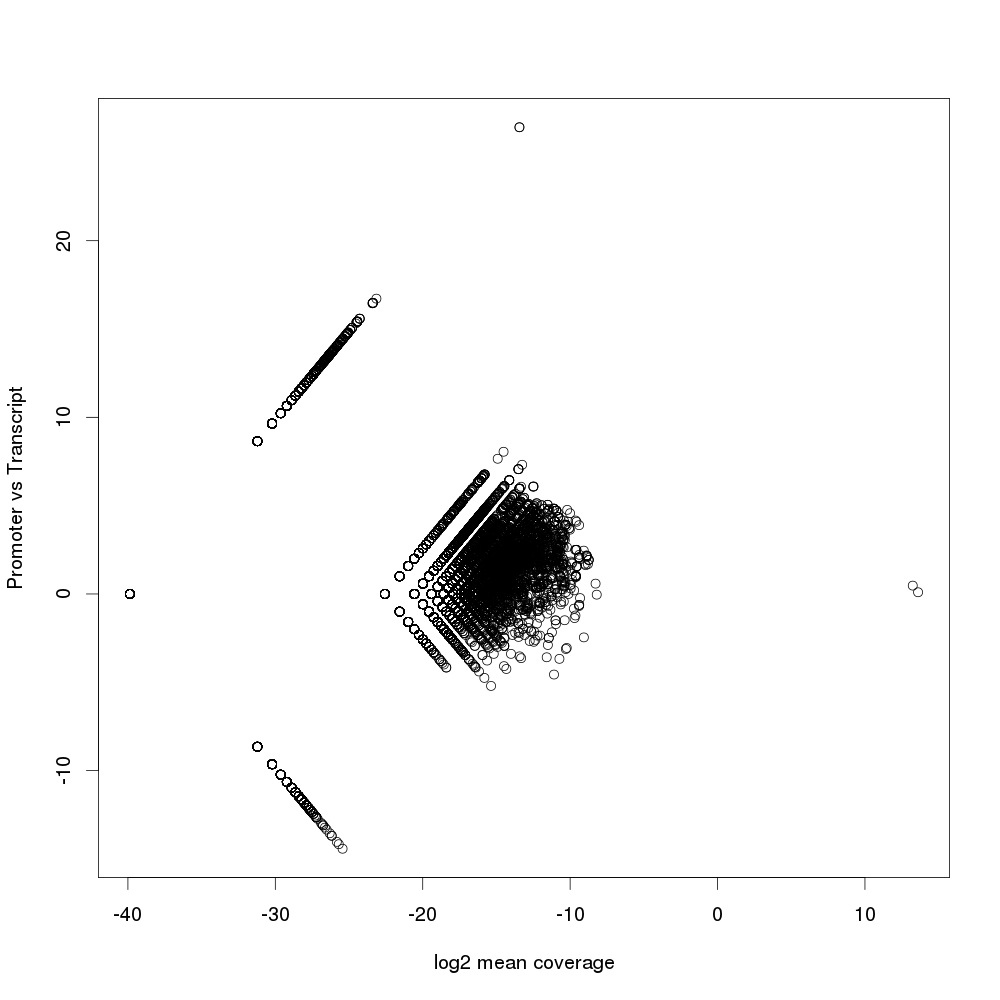

Supplement: S2 File — (ZIP) [file pone.0232332.s012.zip › nucleosome_positioning/SRR891275_pt_score.png]

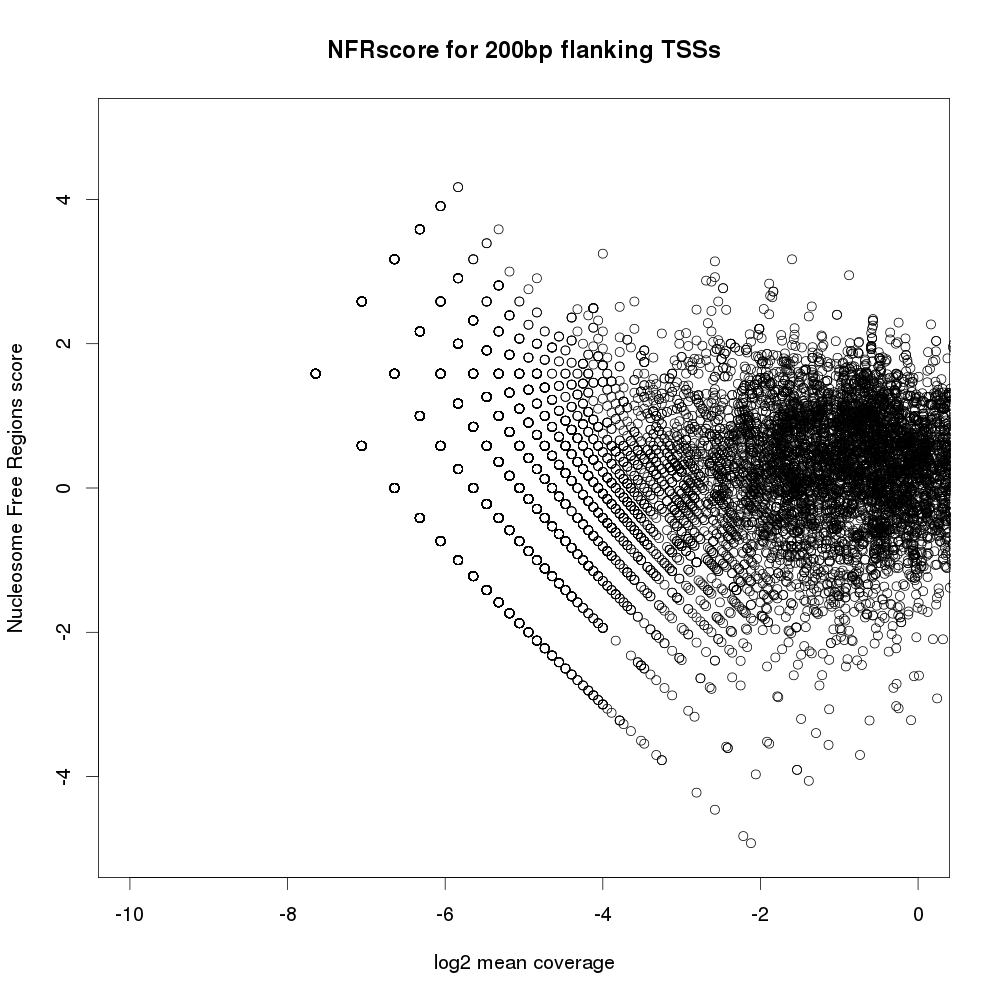

Supplement: S2 File — (ZIP) [file pone.0232332.s012.zip › nucleosome_positioning/SRR5063986_NFRscore.png]

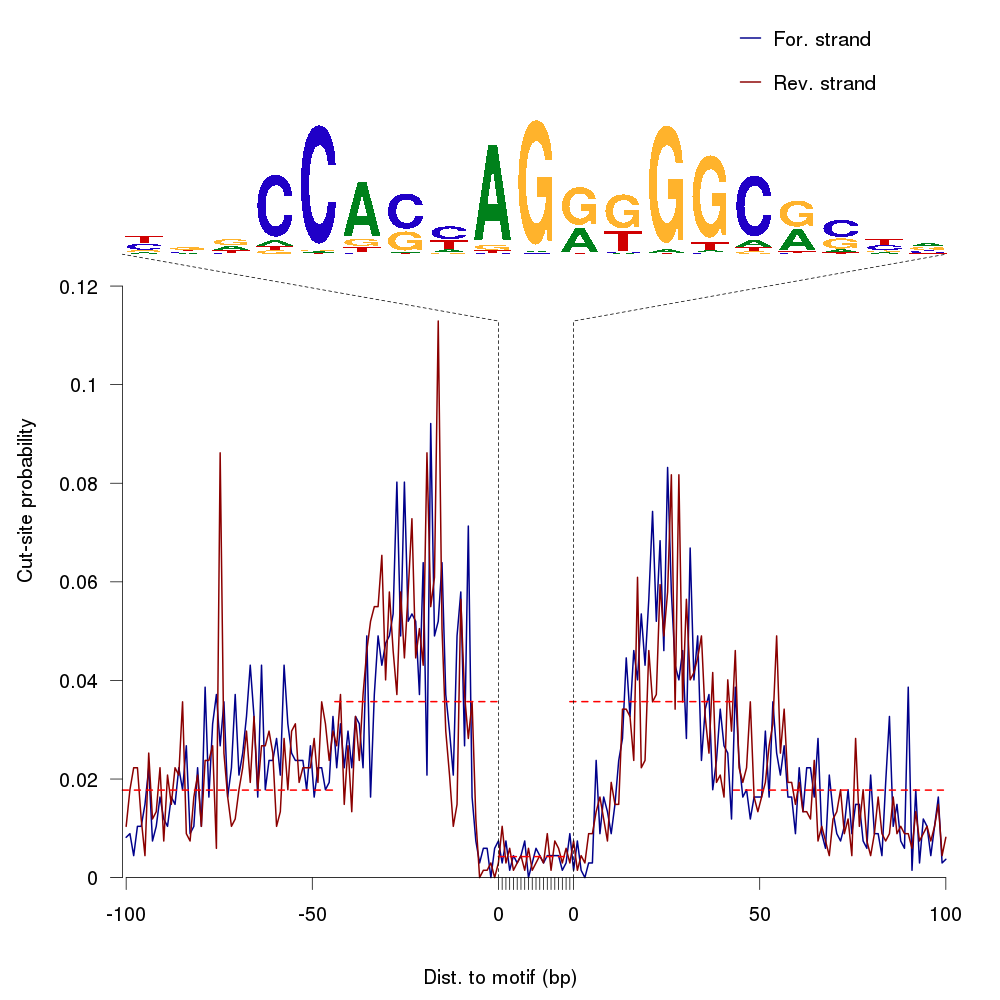

Supplement: S2 File — (ZIP) [file pone.0232332.s012.zip › nucleosome_positioning/SRR5876159_footprint_plot.png]

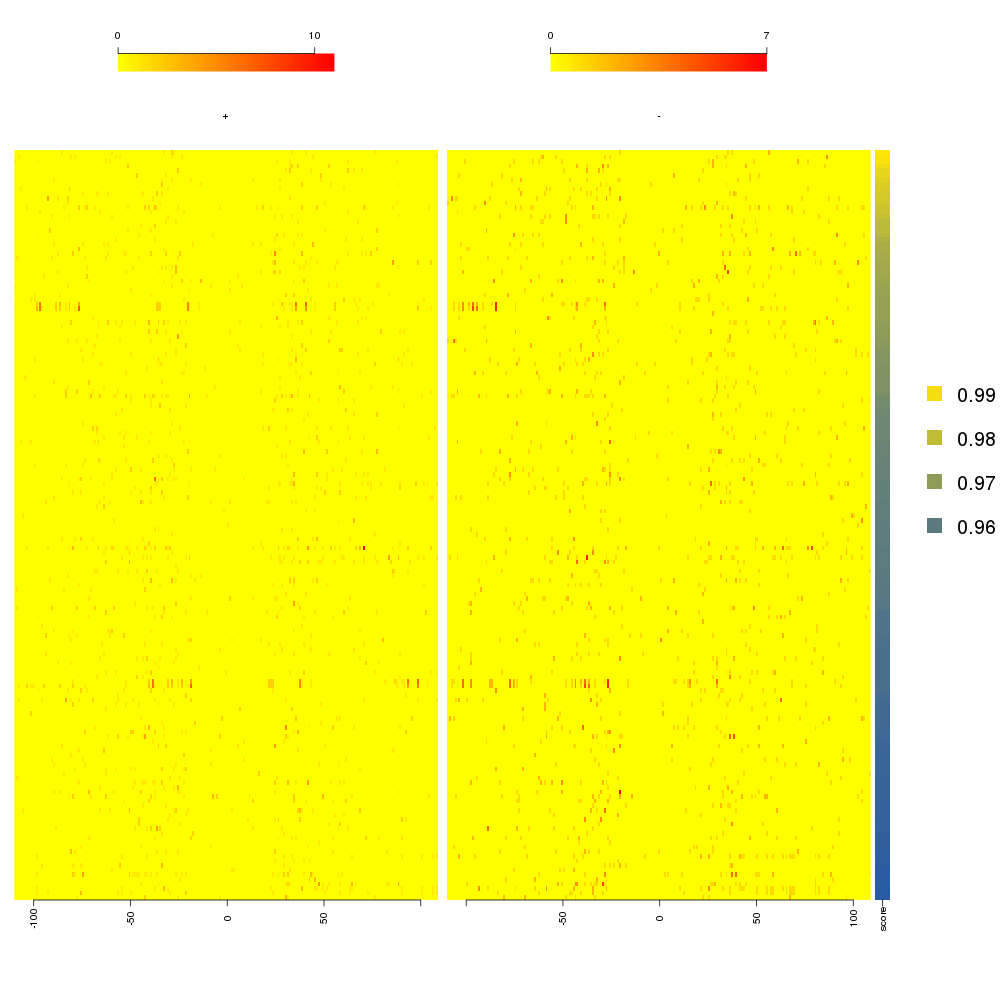

Supplement: S2 File — (ZIP) [file pone.0232332.s012.zip › nucleosome_positioning/SRR3622818_feature_aligned_heatmap.png]

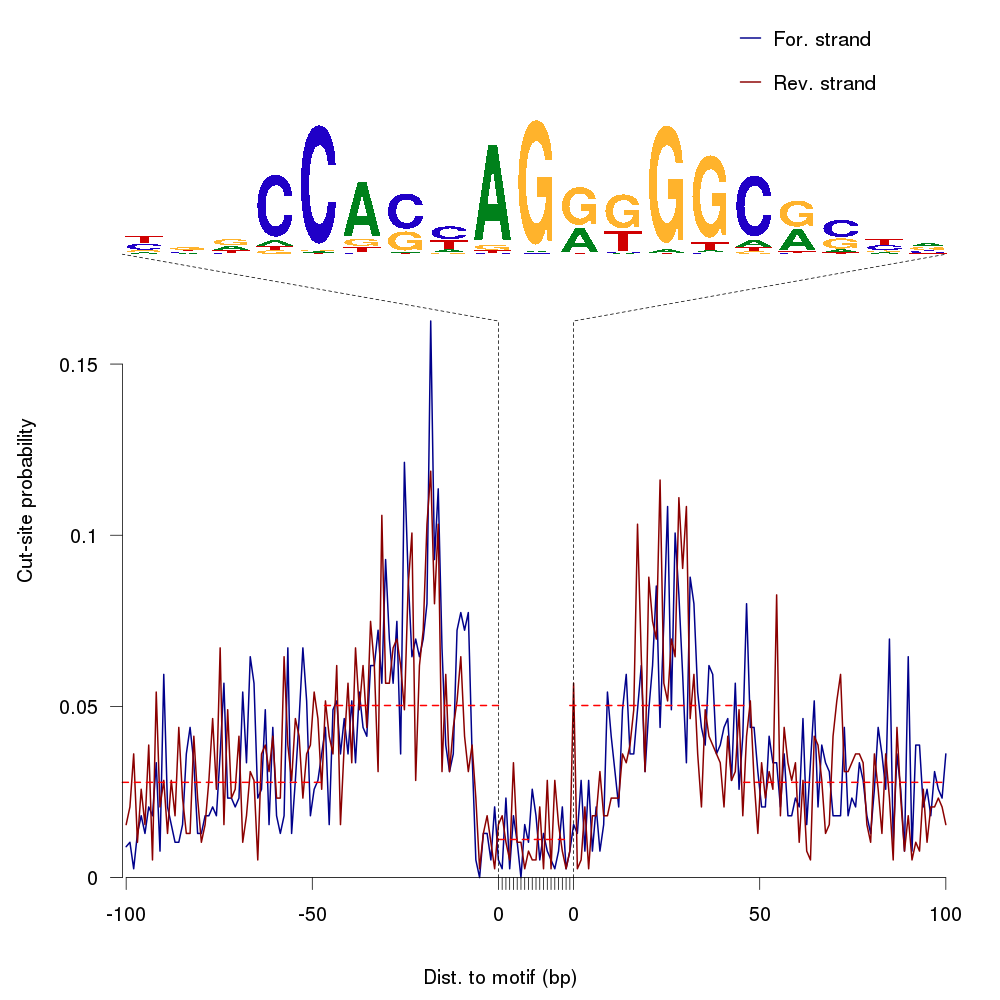

Supplement: S2 File — (ZIP) [file pone.0232332.s012.zip › nucleosome_positioning/SRR3622817_footprint_plot.png]

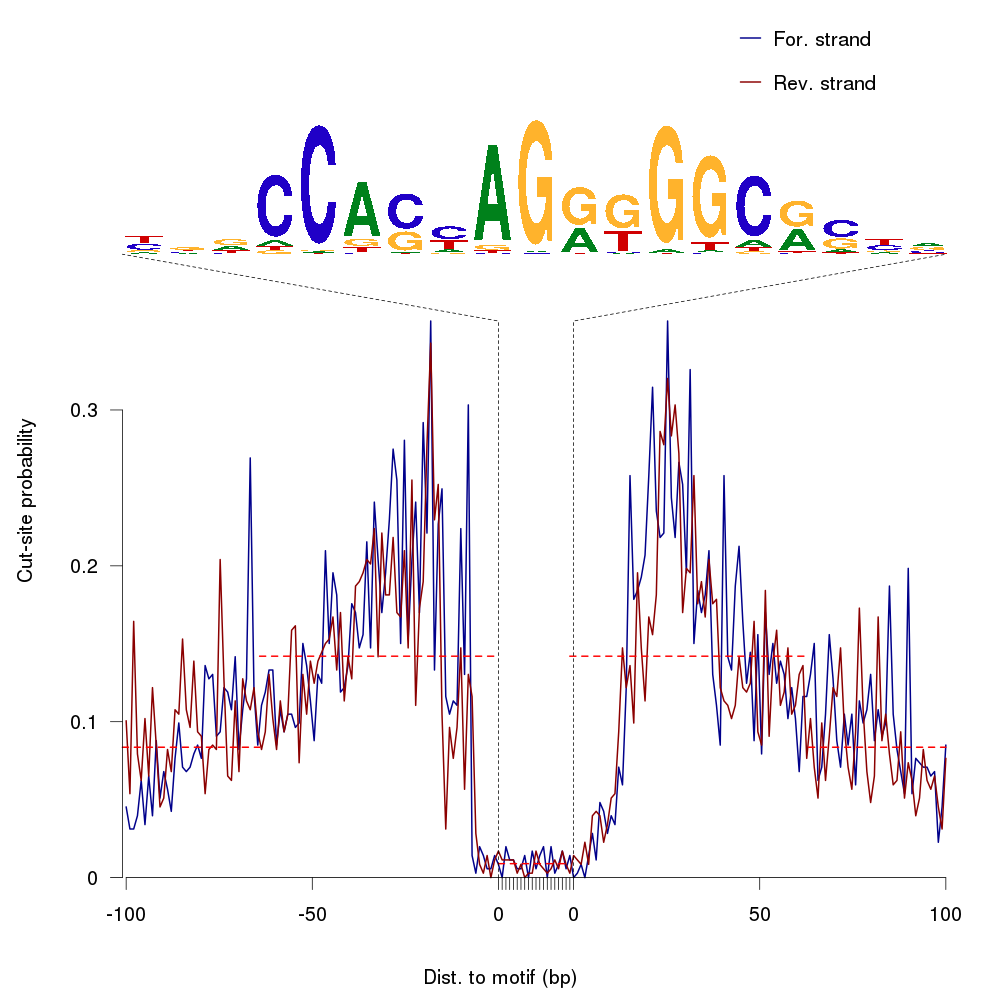

Supplement: S2 File — (ZIP) [file pone.0232332.s012.zip › nucleosome_positioning/SRR5007259_footprint_plot.png]

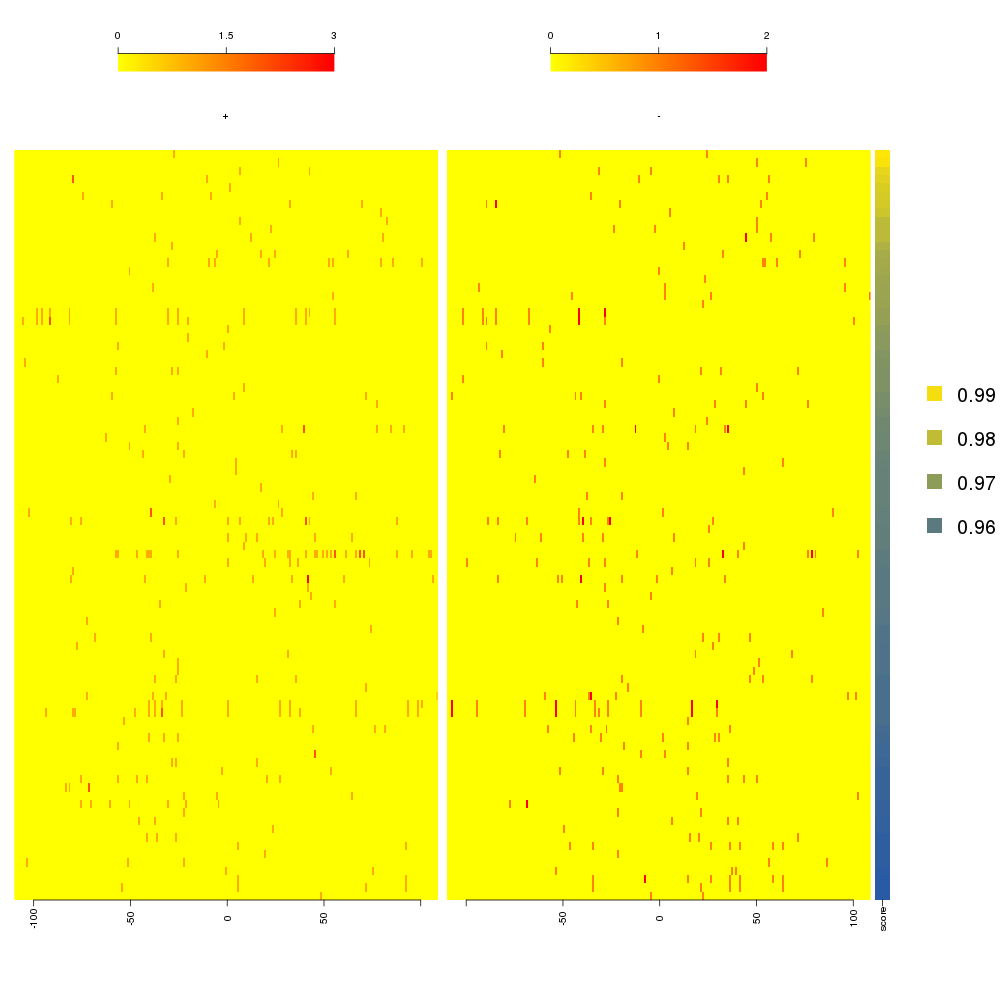

Supplement: S2 File — (ZIP) [file pone.0232332.s012.zip › nucleosome_positioning/SRR891275_feature_aligned_heatmap.png]

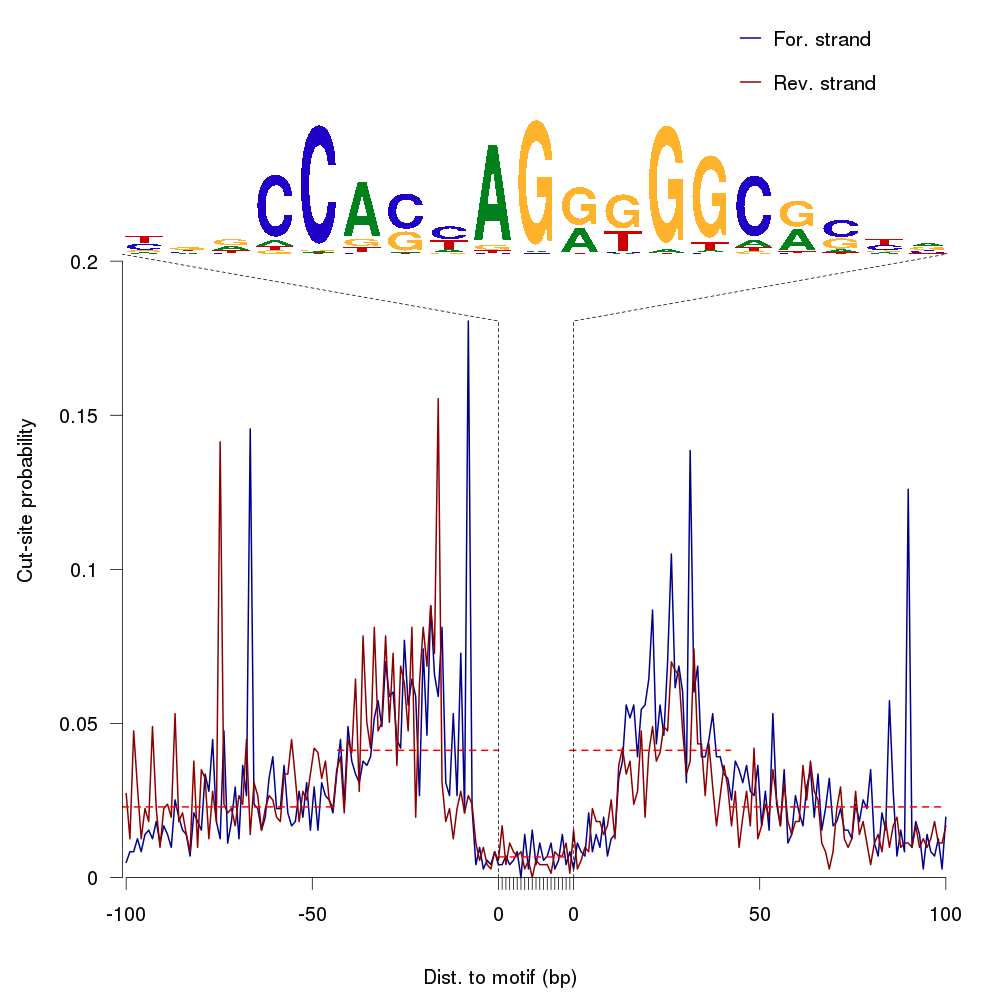

Supplement: S2 File — (ZIP) [file pone.0232332.s012.zip › nucleosome_positioning/SRR8932927_footprint_plot.png]

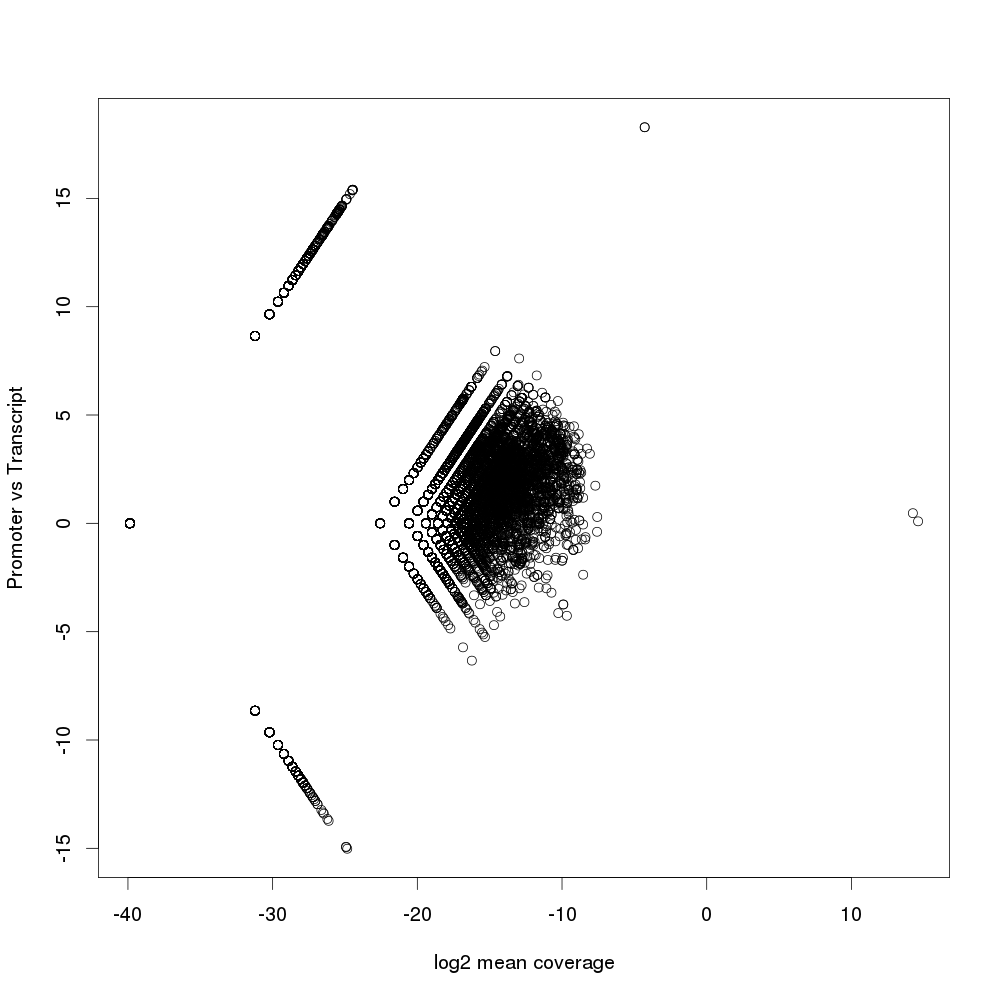

Supplement: S2 File — (ZIP) [file pone.0232332.s012.zip › nucleosome_positioning/SRR891276_pt_score.png]

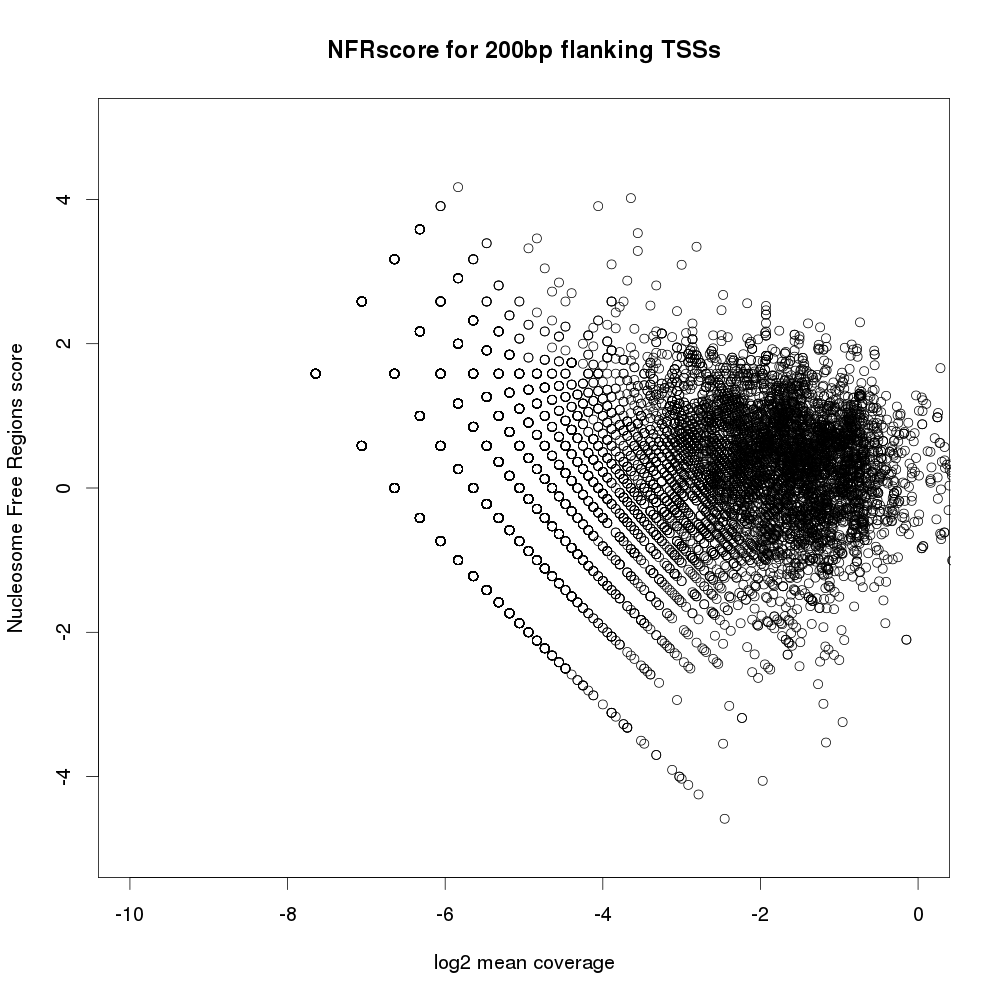

Supplement: S2 File — (ZIP) [file pone.0232332.s012.zip › nucleosome_positioning/SRR5063985_NFRscore.png]

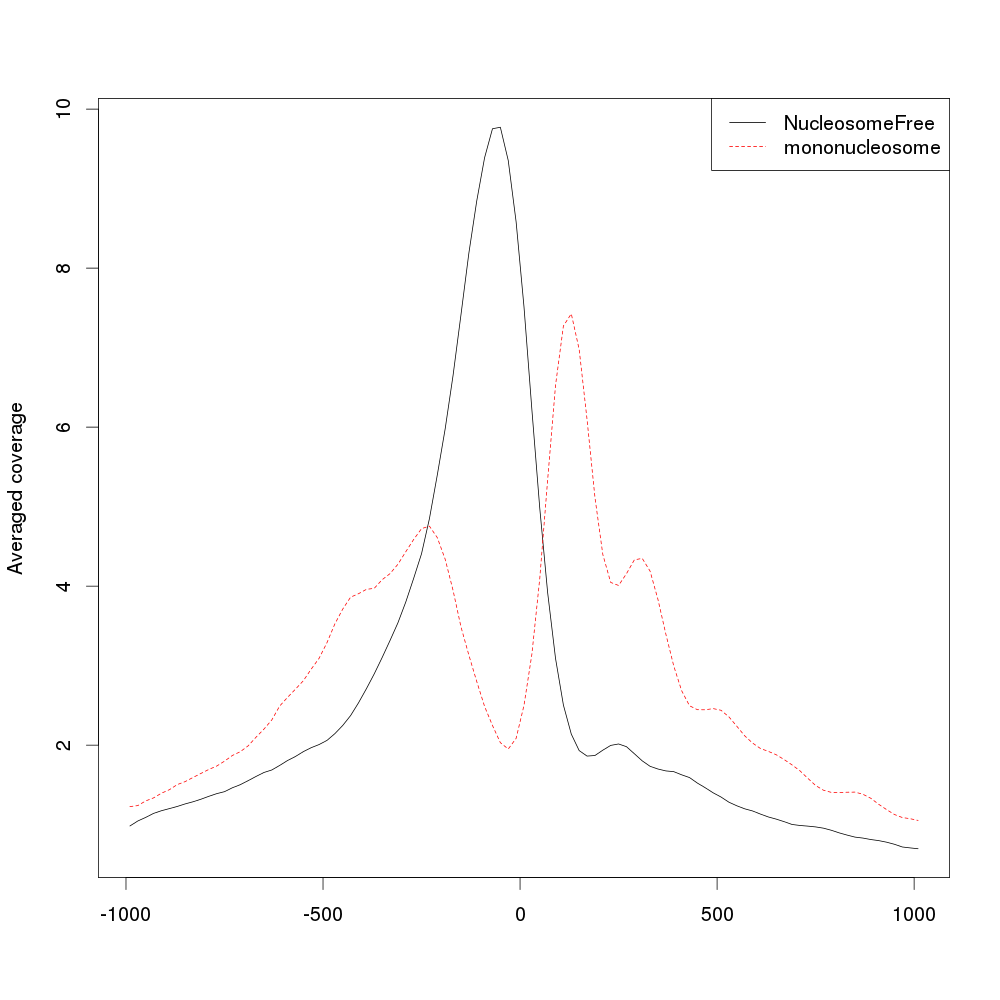

Supplement: S2 File — (ZIP) [file pone.0232332.s012.zip › nucleosome_positioning/SRR6216227_nucleosome_distribution.png]

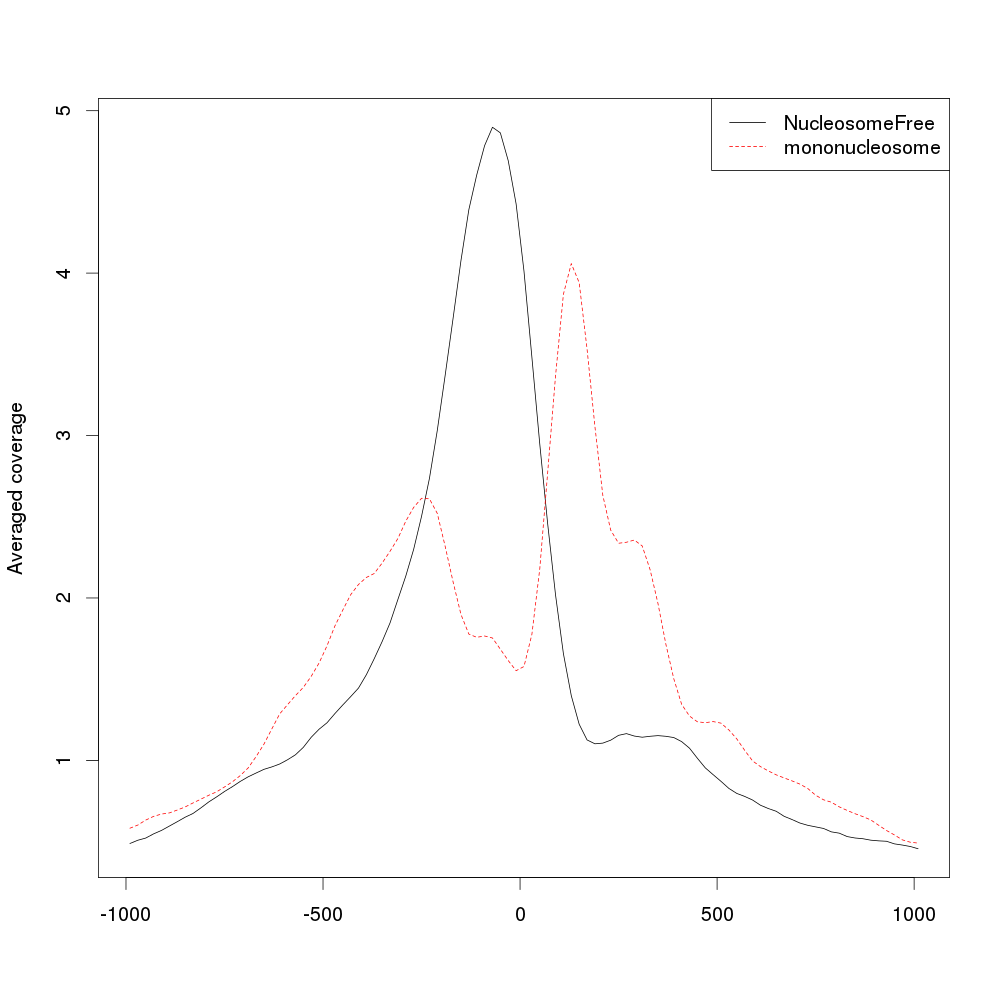

Supplement: S2 File — (ZIP) [file pone.0232332.s012.zip › nucleosome_positioning/SRX6443491_nucleosome_distribution.png]

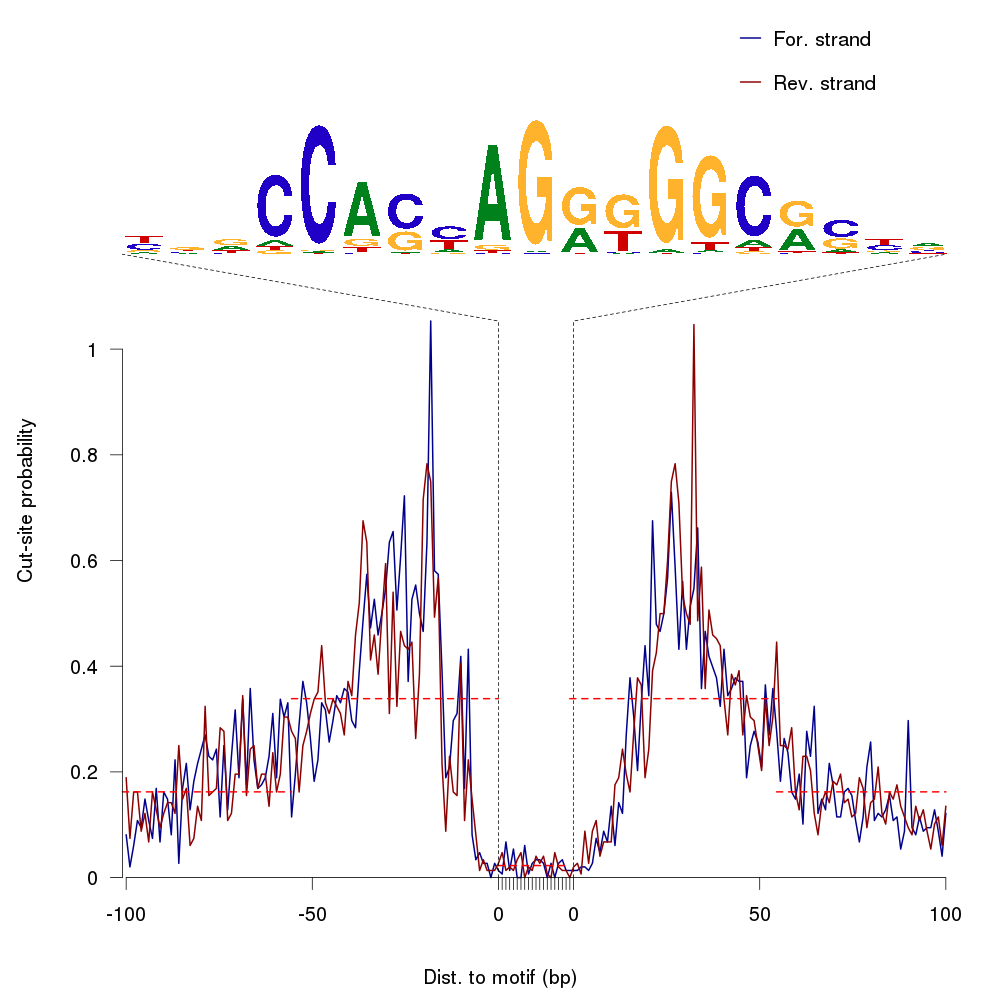

Supplement: S2 File — (ZIP) [file pone.0232332.s012.zip › nucleosome_positioning/SRR6216226_footprint_plot.png]

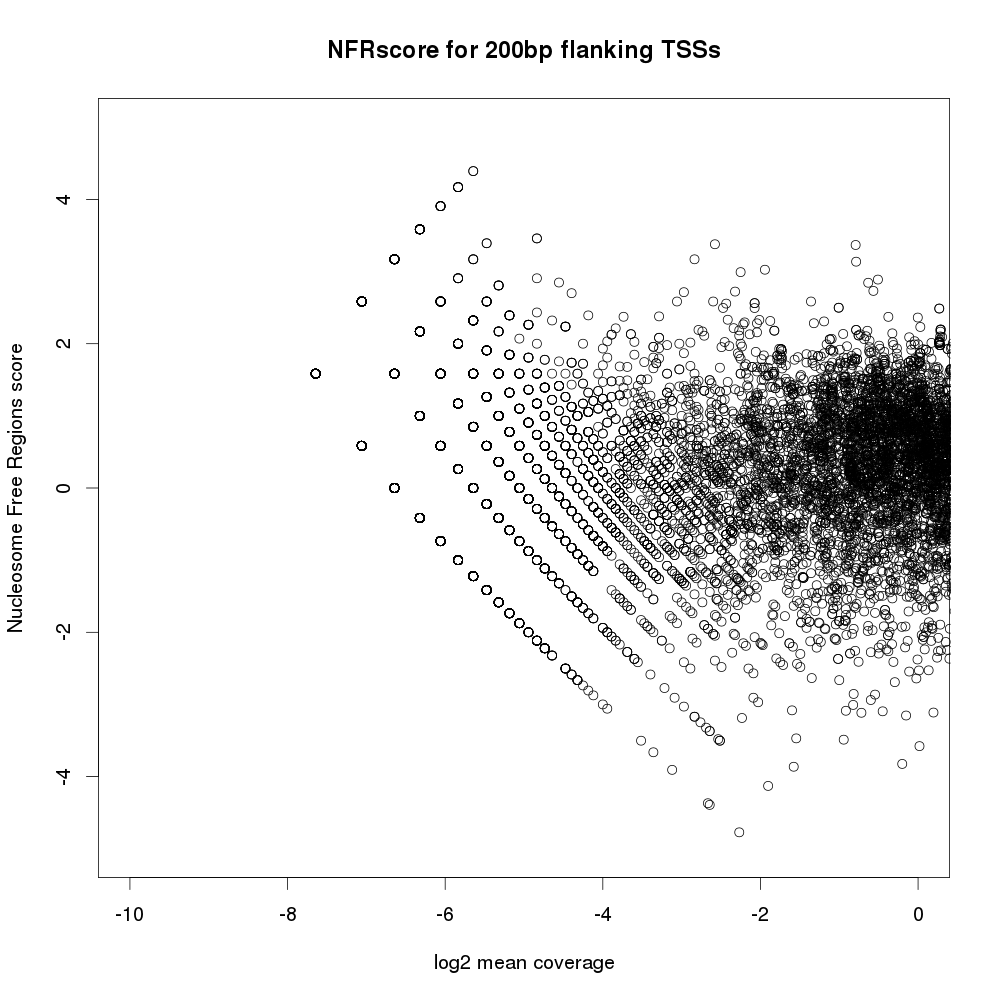

Supplement: S2 File — (ZIP) [file pone.0232332.s012.zip › nucleosome_positioning/SRR5063984_NFRscore.png]

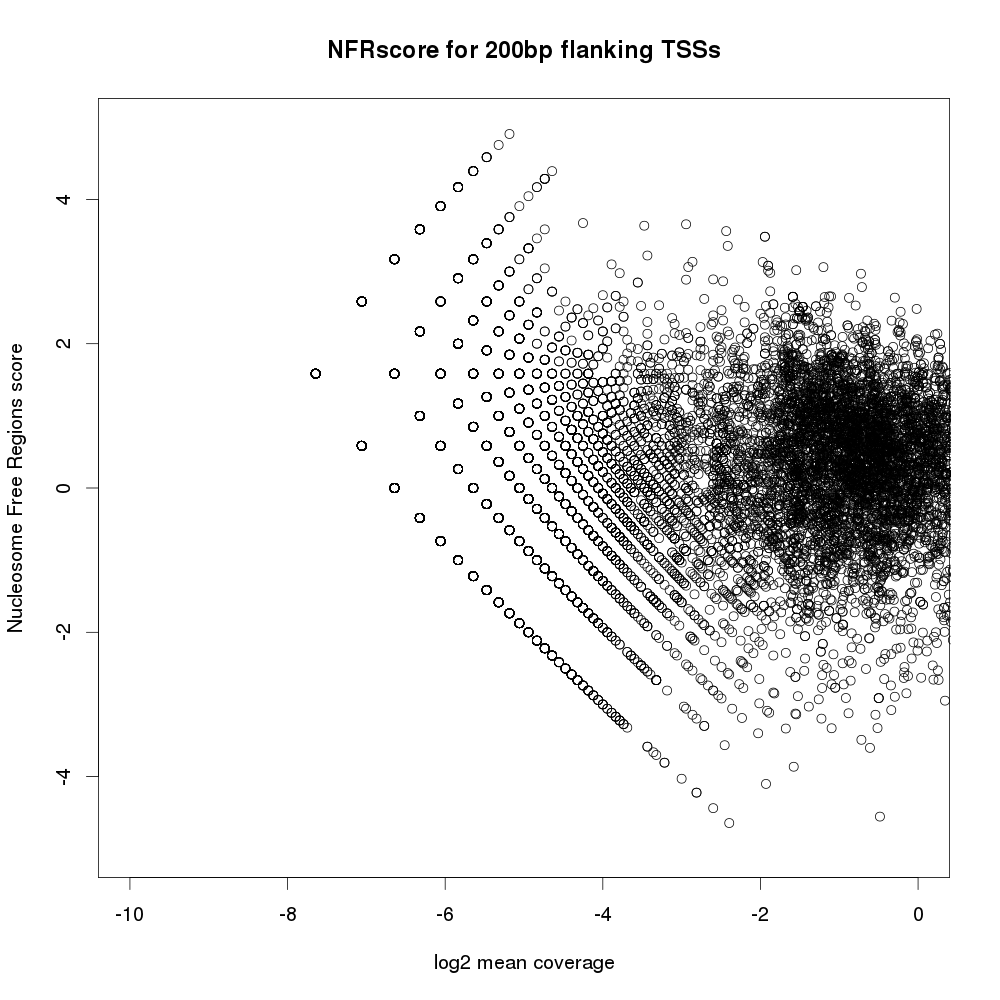

Supplement: S2 File — (ZIP) [file pone.0232332.s012.zip › nucleosome_positioning/SRR7140572_NFRscore.png]

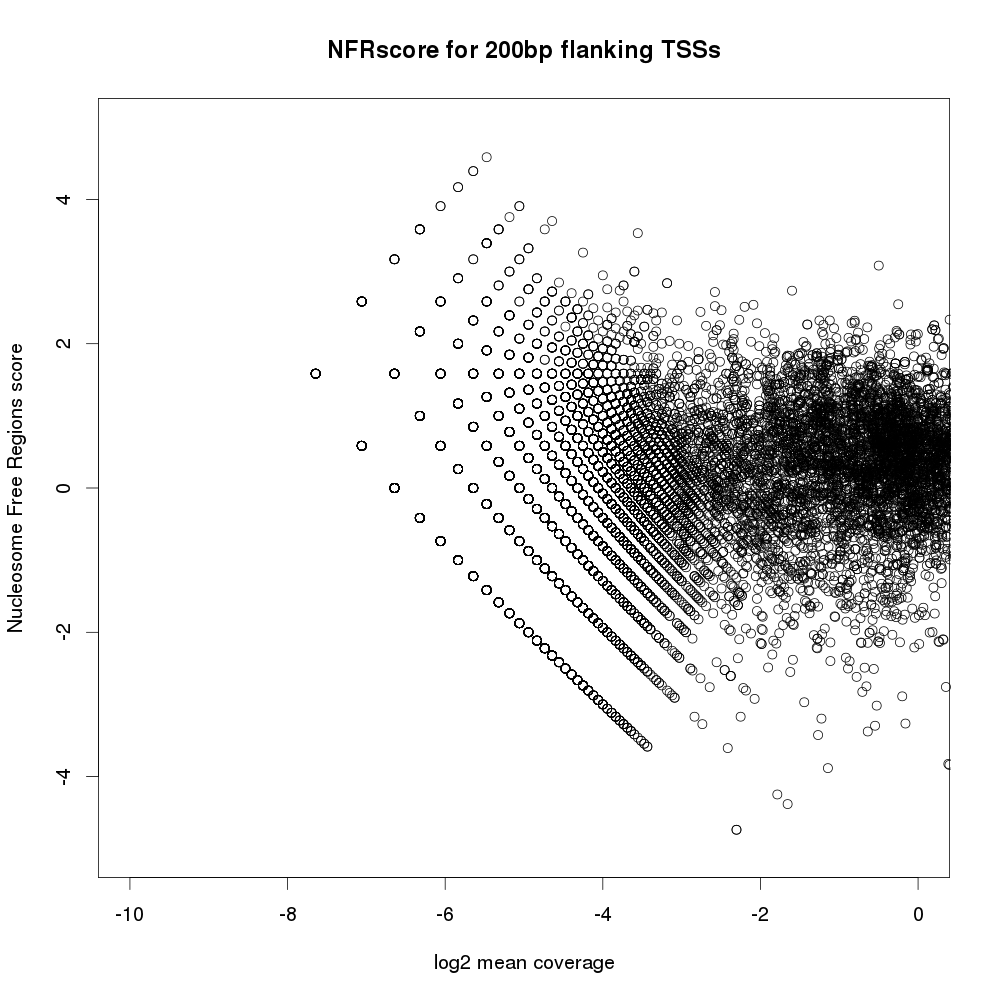

Supplement: S2 File — (ZIP) [file pone.0232332.s012.zip › nucleosome_positioning/SRR1822165_NFRscore.png]

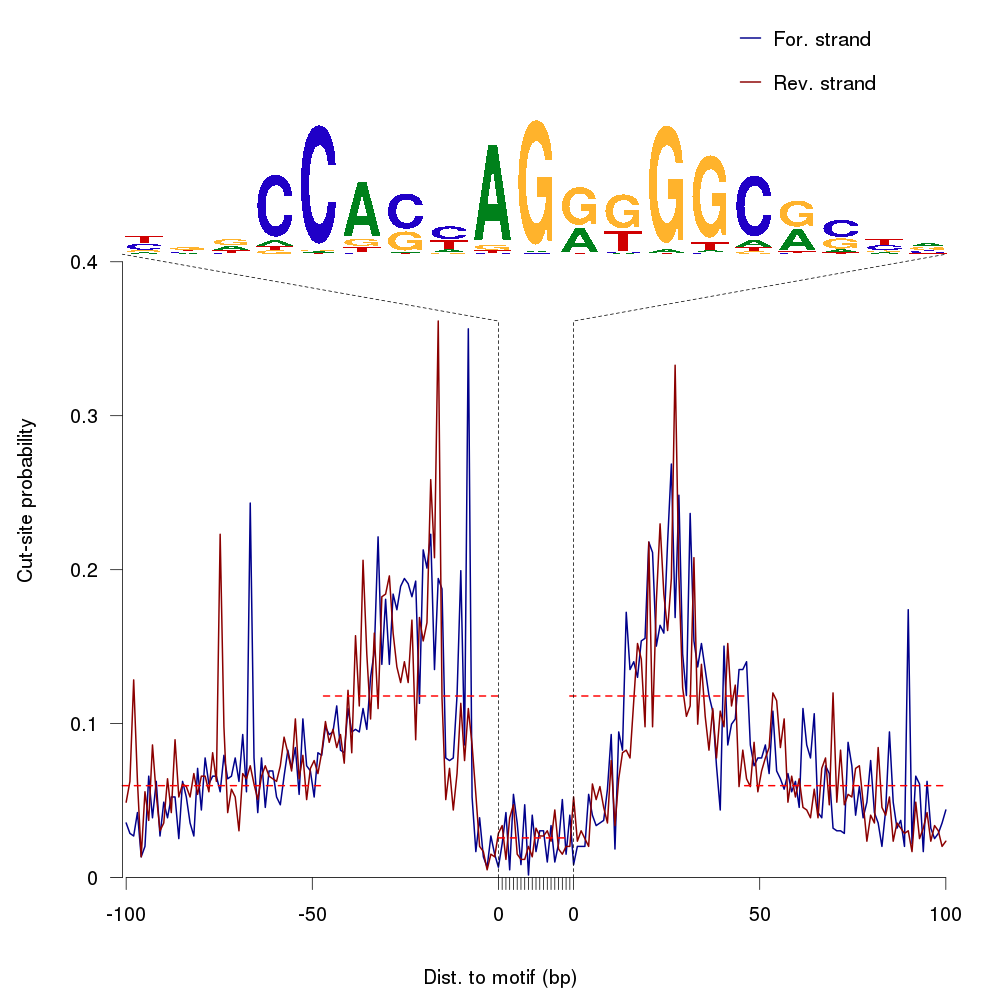

Supplement: S2 File — (ZIP) [file pone.0232332.s012.zip › nucleosome_positioning/SRR1822165_footprint_plot.png]

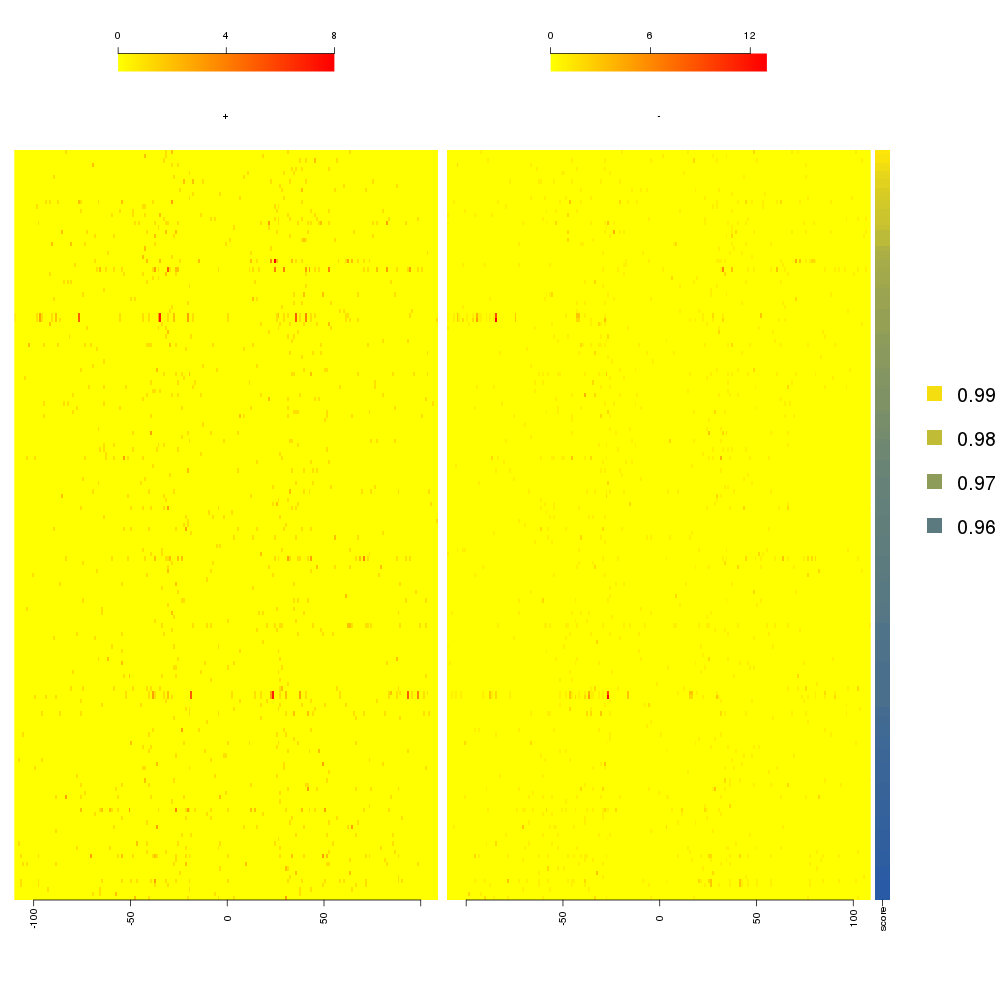

Supplement: S2 File — (ZIP) [file pone.0232332.s012.zip › nucleosome_positioning/SRR5128074_feature_aligned_heatmap.png]

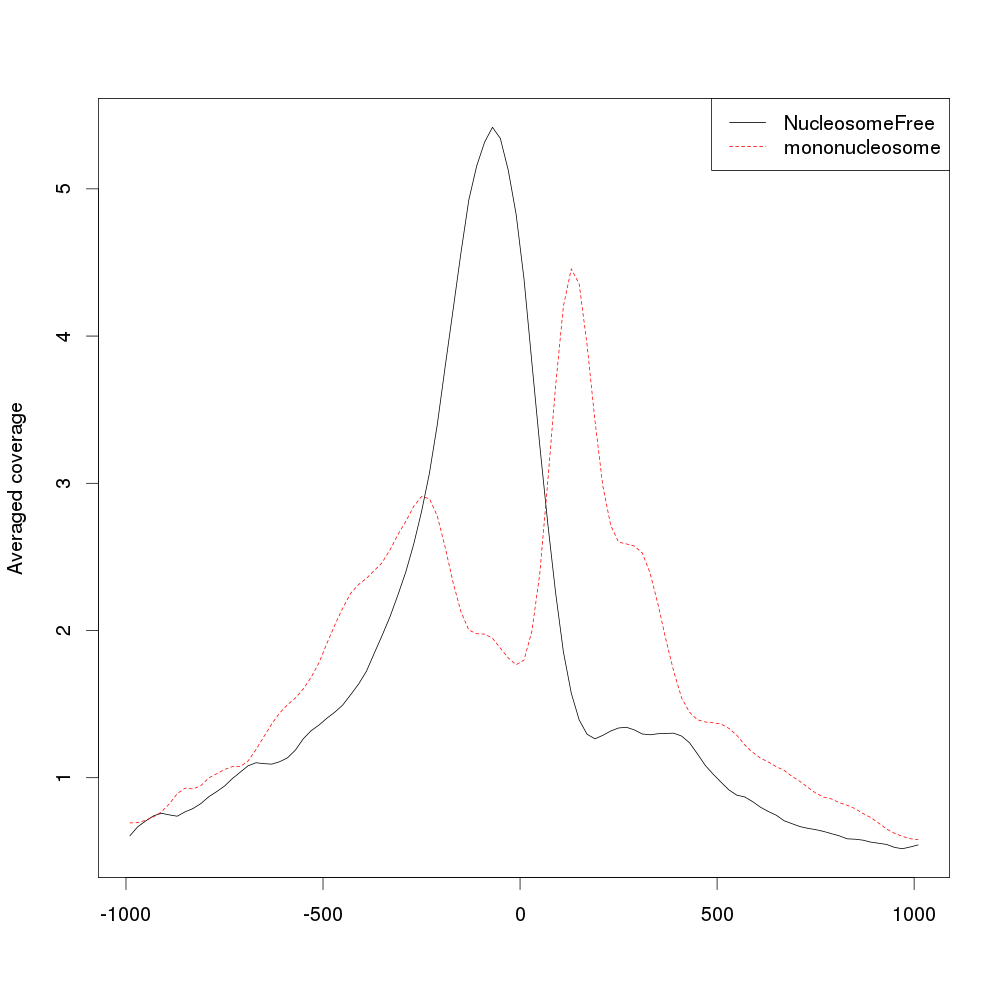

Supplement: S2 File — (ZIP) [file pone.0232332.s012.zip › nucleosome_positioning/SRX6443489_nucleosome_distribution.png]

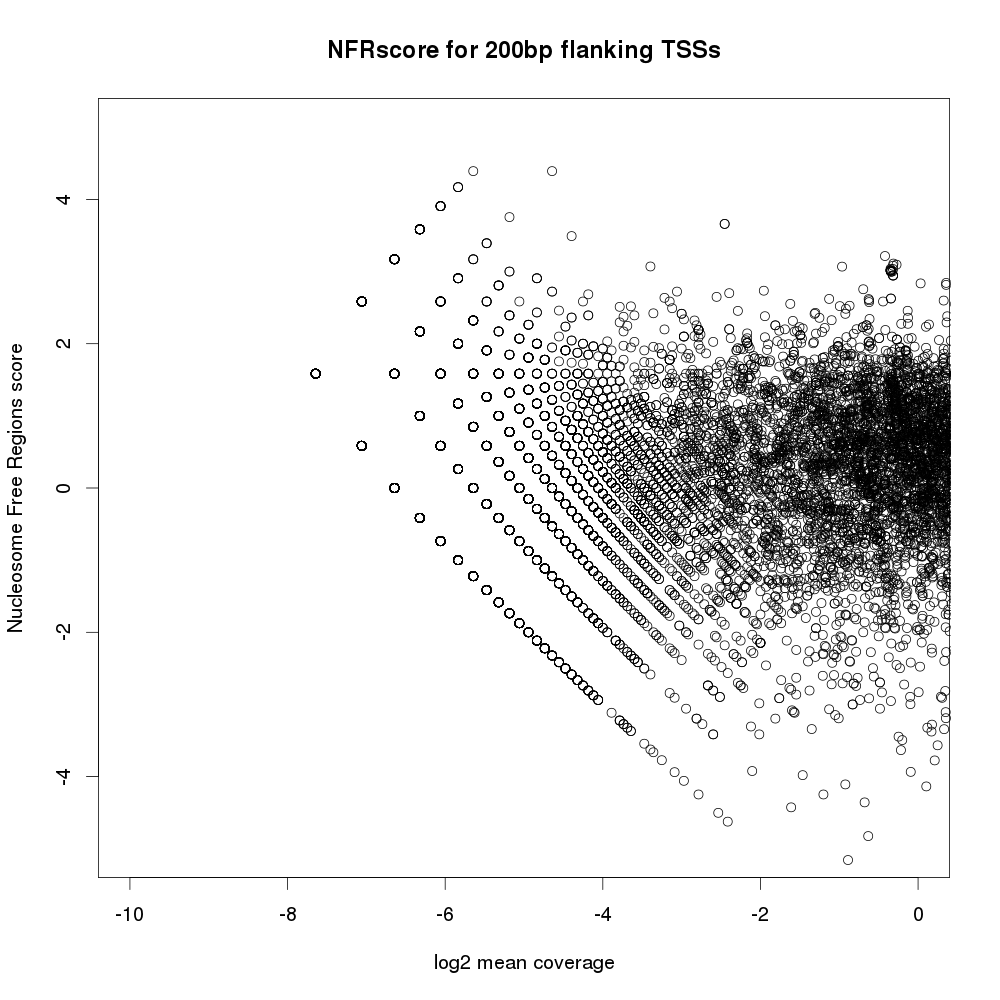

Supplement: S2 File — (ZIP) [file pone.0232332.s012.zip › nucleosome_positioning/SRX6443490_NFRscore.png]

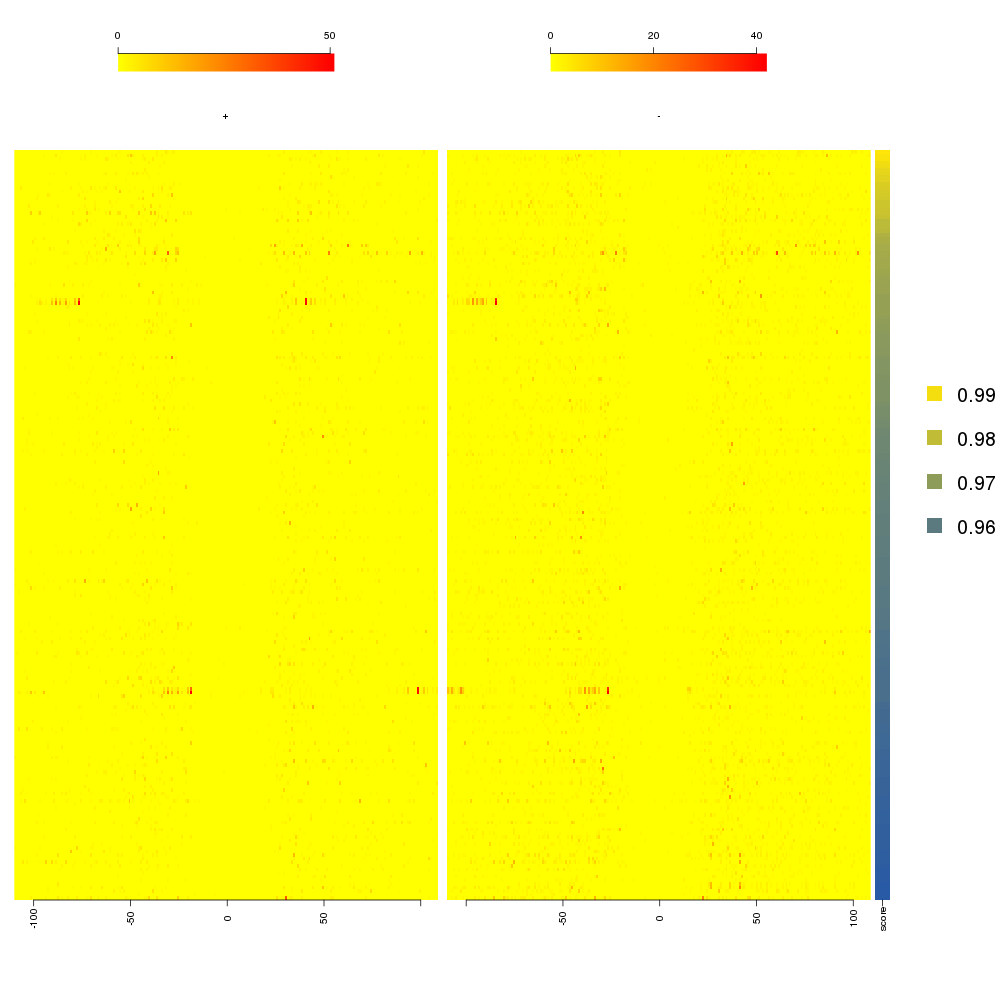

Supplement: S2 File — (ZIP) [file pone.0232332.s012.zip › nucleosome_positioning/SRR5007258_feature_aligned_heatmap.png]

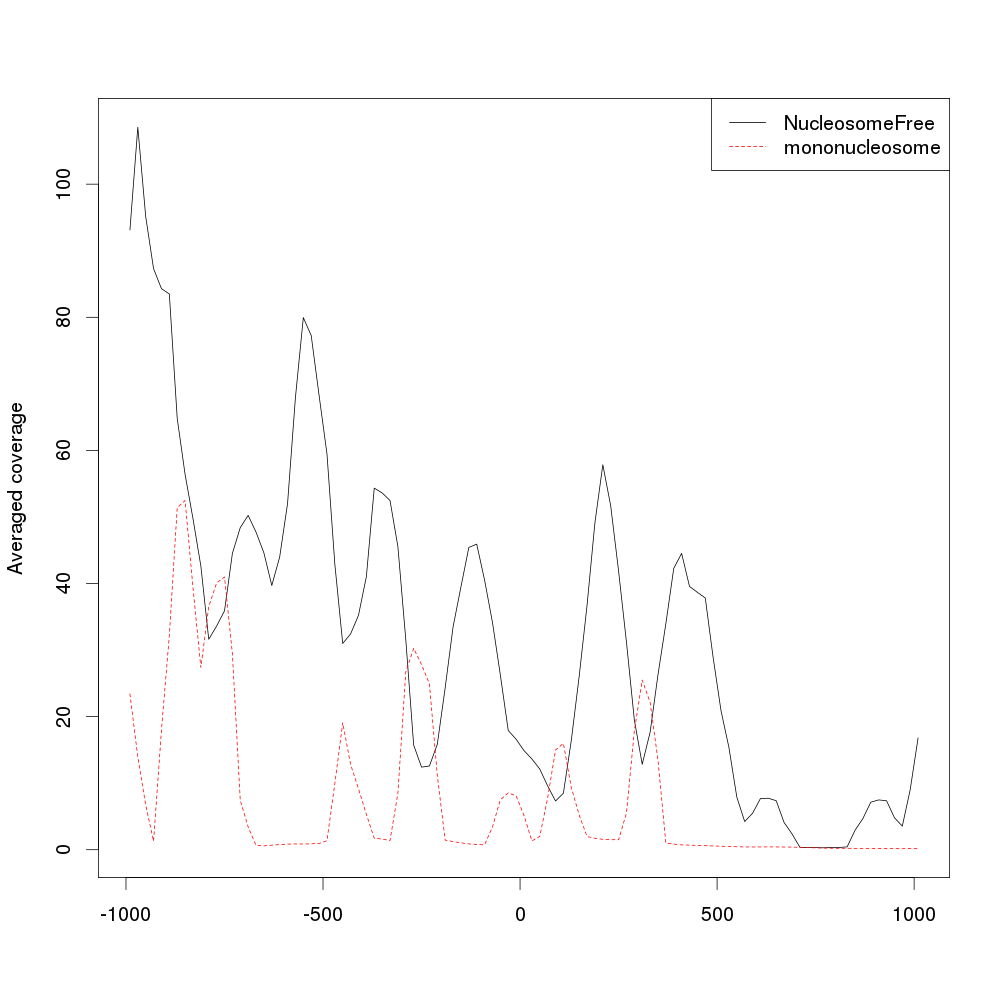

Supplement: S2 File — (ZIP) [file pone.0232332.s012.zip › nucleosome_positioning/SRR8932927_nucleosome_distribution.png]

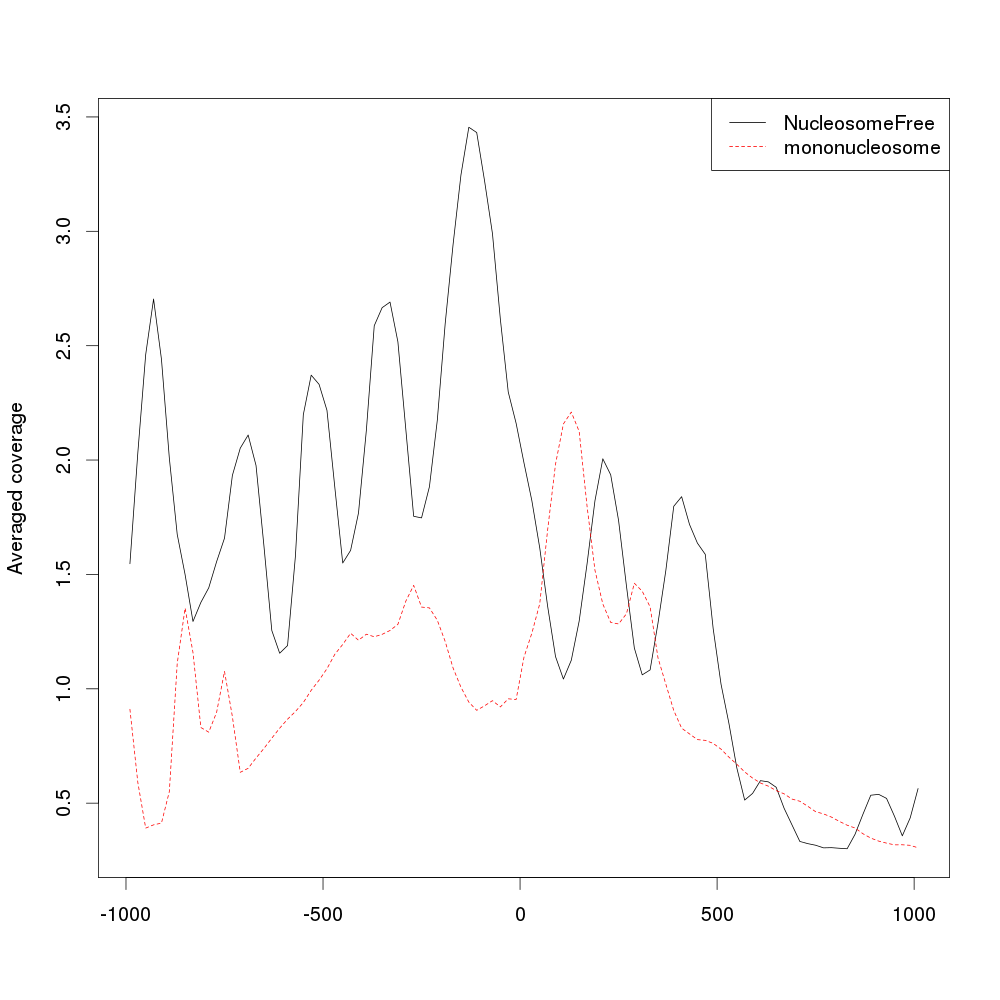

Supplement: S2 File — (ZIP) [file pone.0232332.s012.zip › nucleosome_positioning/SRR5063985_nucleosome_distribution.png]

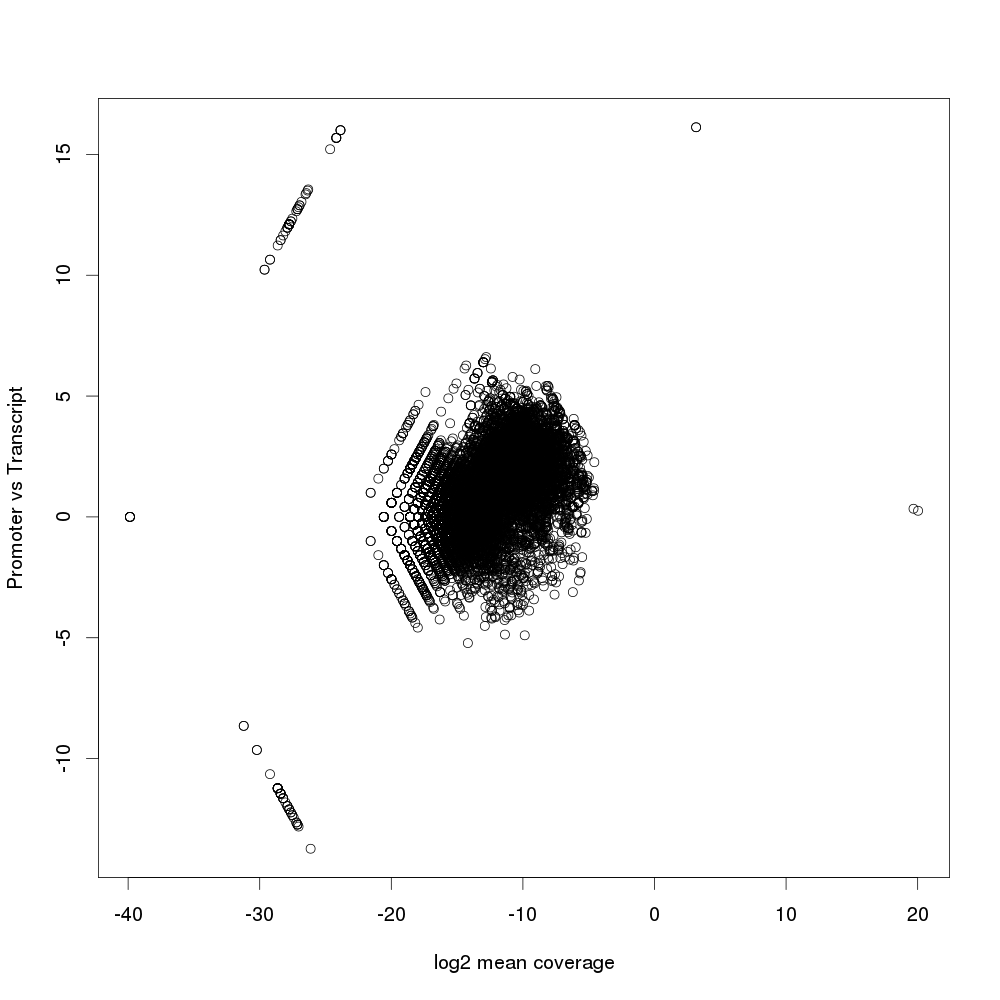

Supplement: S2 File — (ZIP) [file pone.0232332.s012.zip › nucleosome_positioning/SRR1822168_pt_score.png]

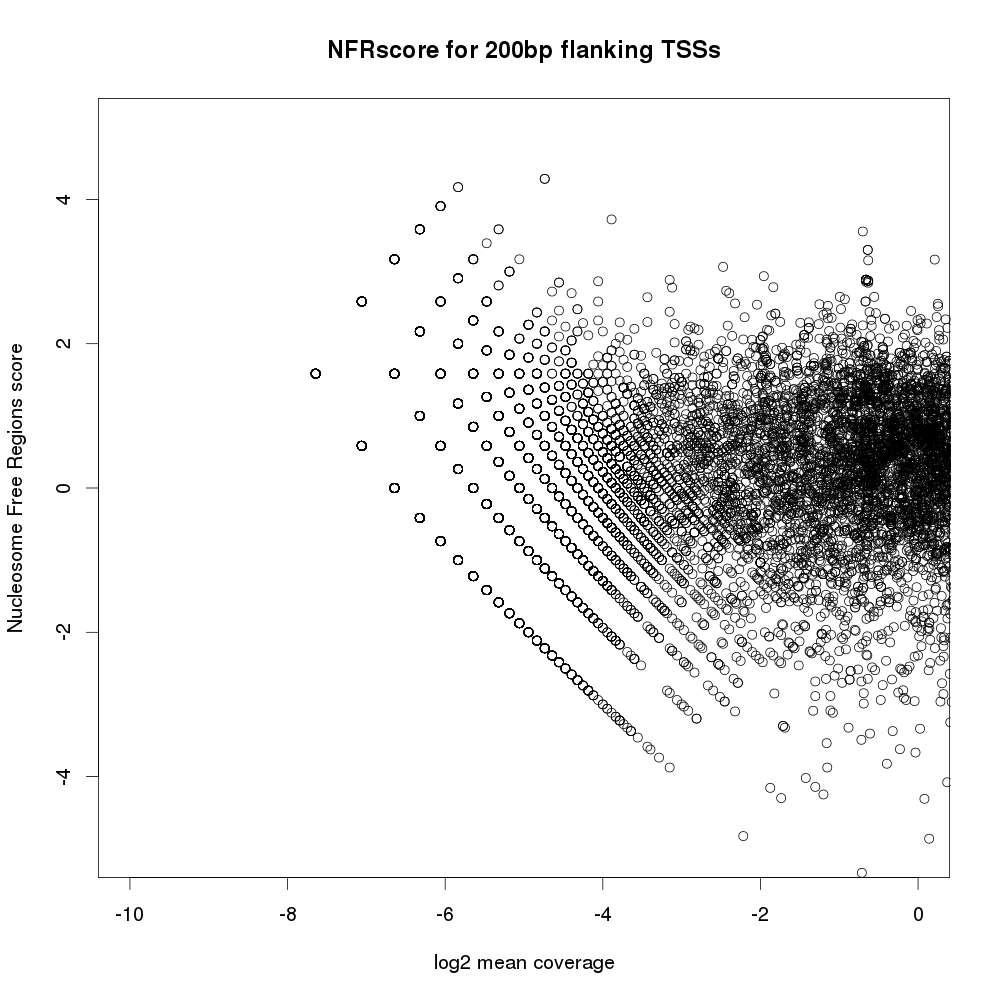

Supplement: S2 File — (ZIP) [file pone.0232332.s012.zip › nucleosome_positioning/SRX6443491_NFRscore.png]

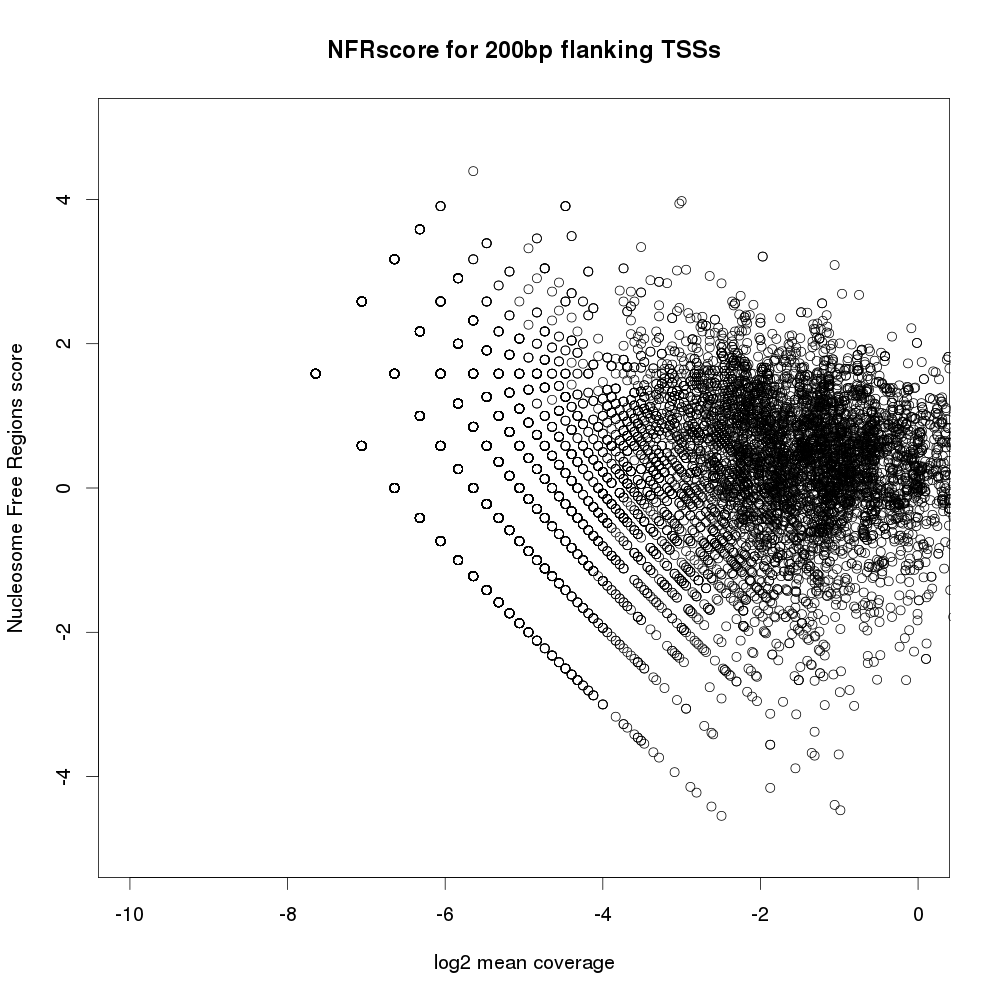

Supplement: S2 File — (ZIP) [file pone.0232332.s012.zip › nucleosome_positioning/SRR8932927_NFRscore.png]

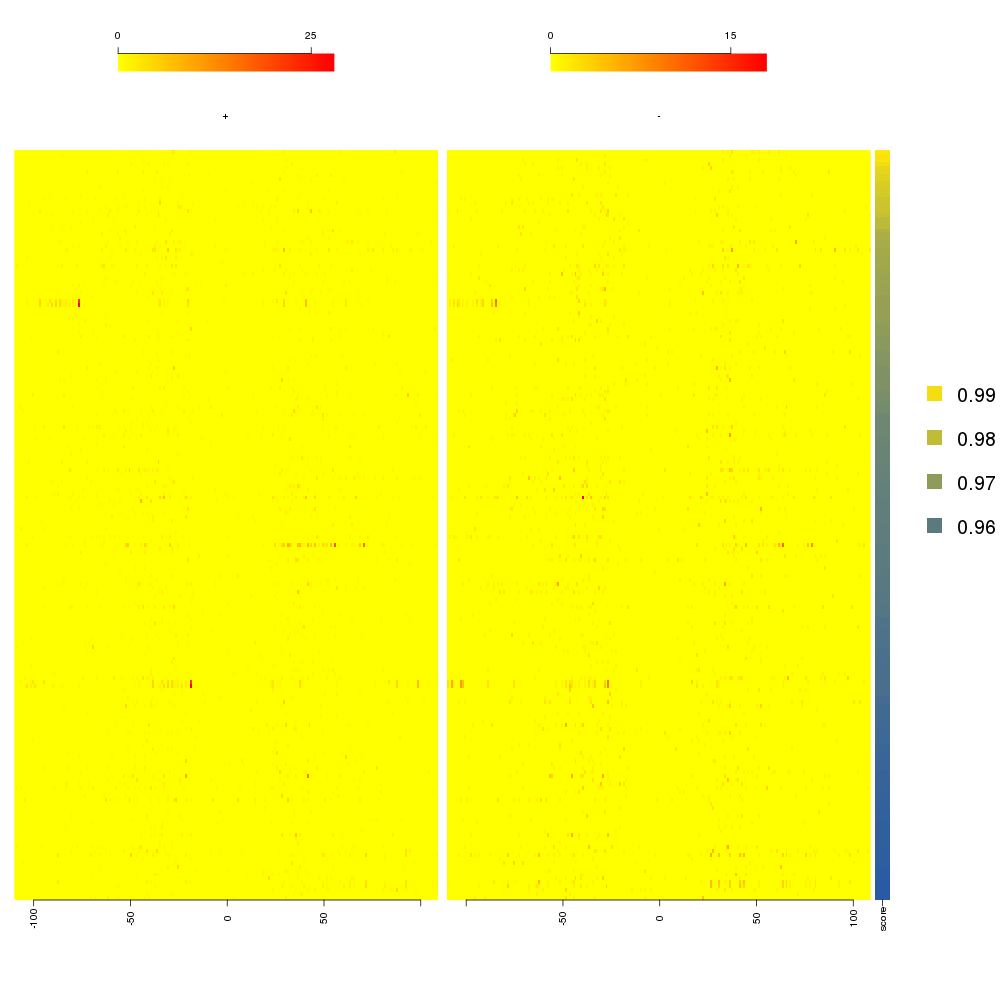

Supplement: S2 File — (ZIP) [file pone.0232332.s012.zip › nucleosome_positioning/SRR1822168_feature_aligned_heatmap.png]

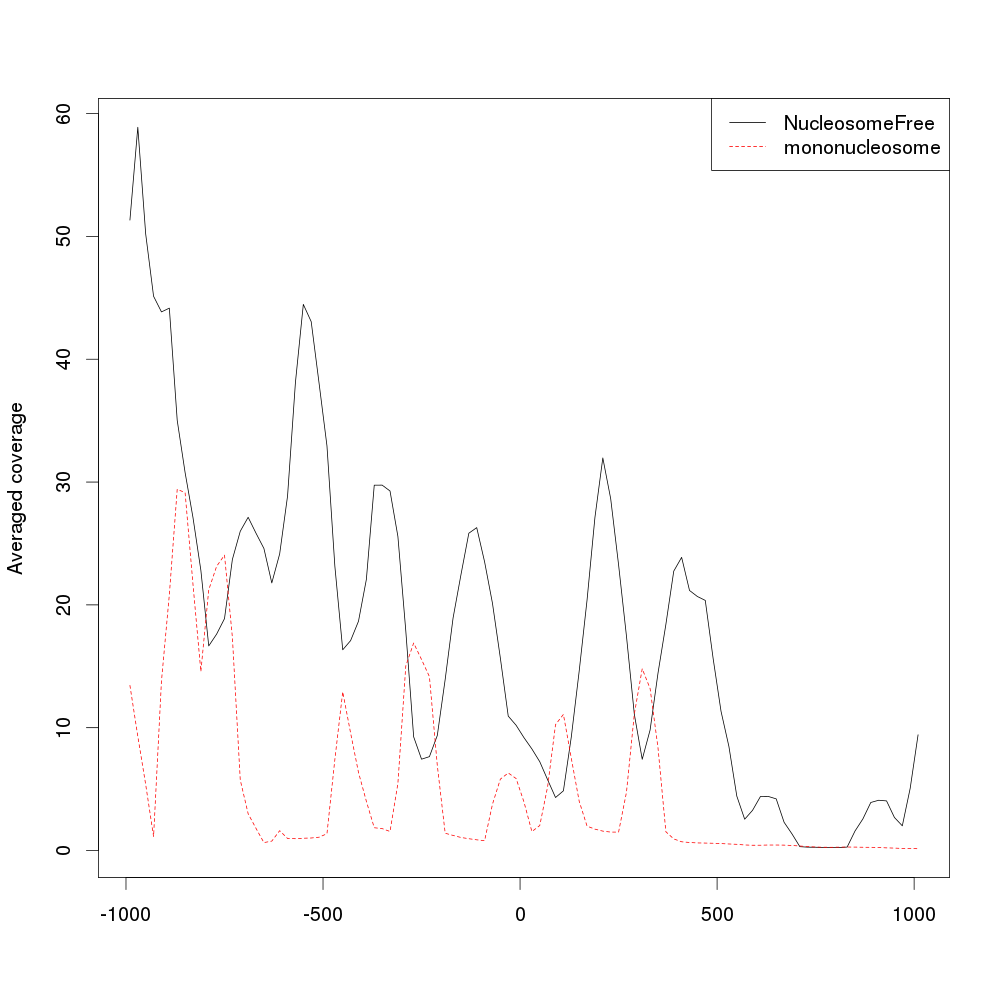

Supplement: S2 File — (ZIP) [file pone.0232332.s012.zip › nucleosome_positioning/SRR8932925_nucleosome_distribution.png]

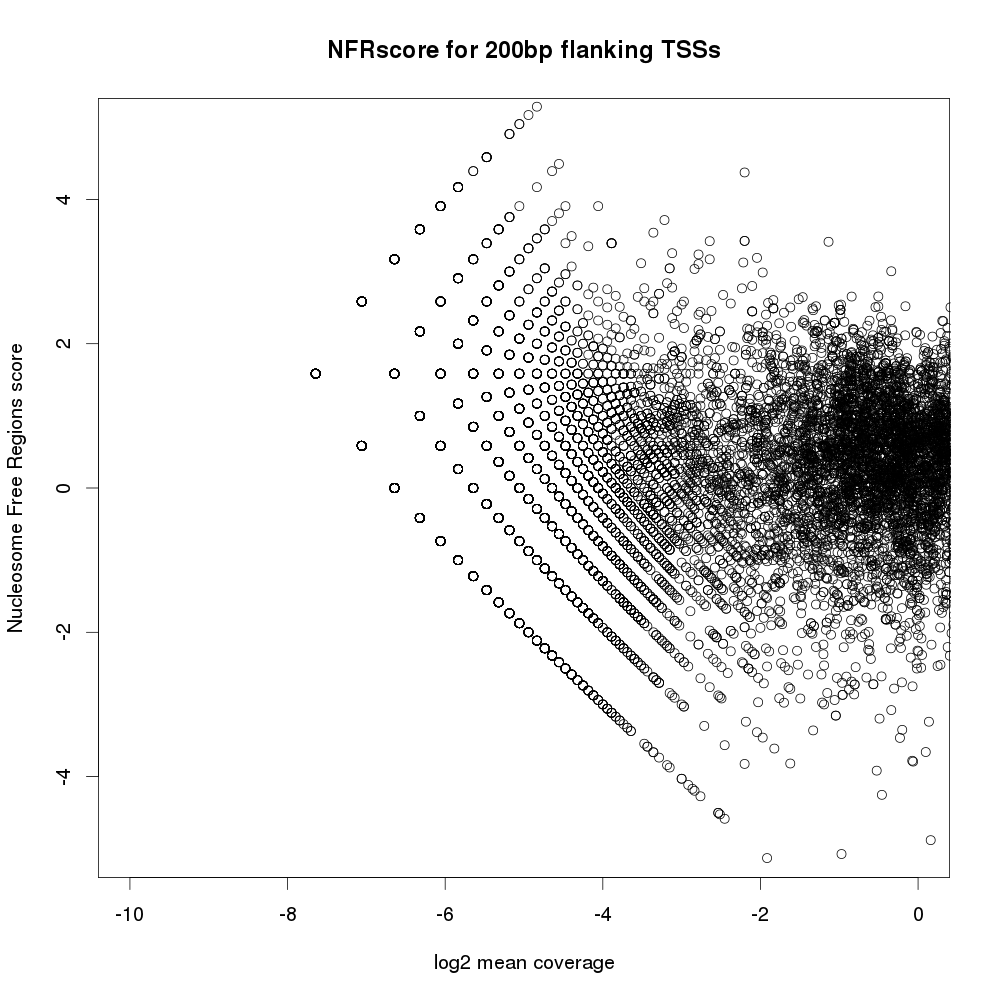

Supplement: S2 File — (ZIP) [file pone.0232332.s012.zip › nucleosome_positioning/SRR7140573_NFRscore.png]

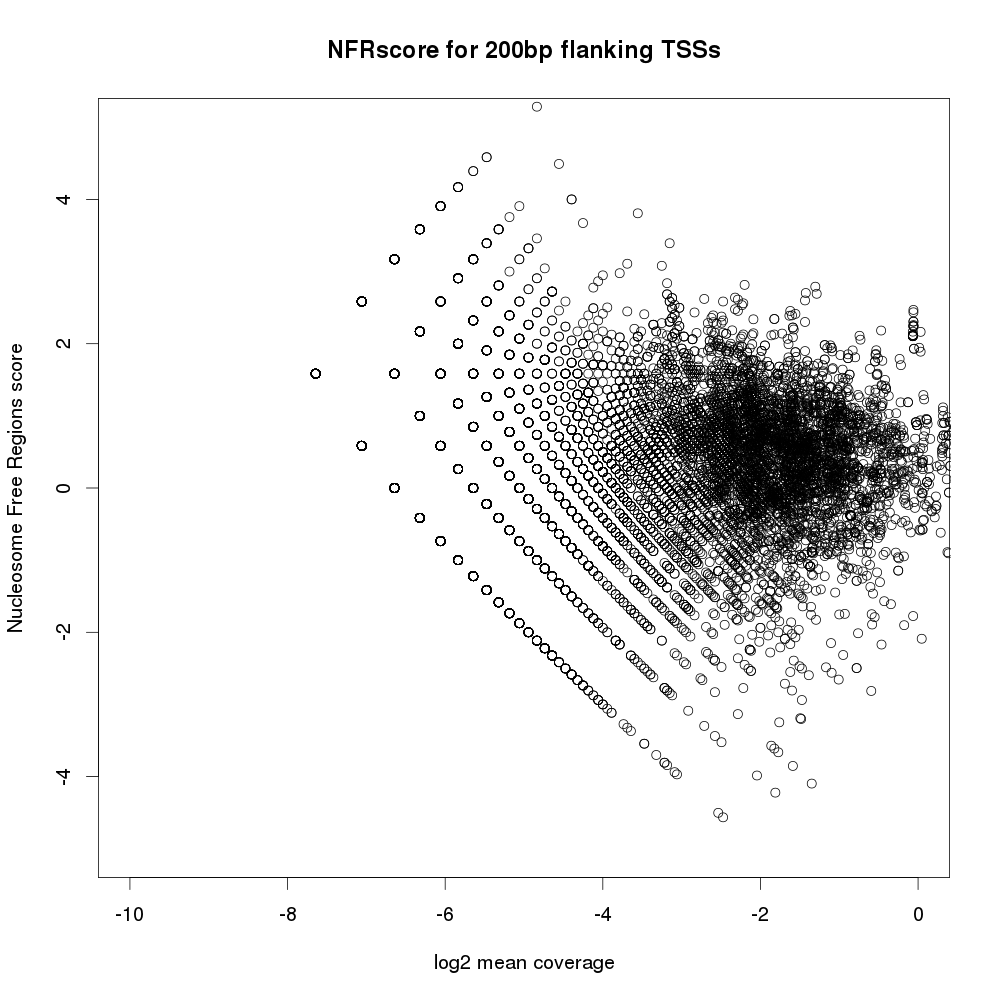

Supplement: S2 File — (ZIP) [file pone.0232332.s012.zip › nucleosome_positioning/SRR1822166_NFRscore.png]

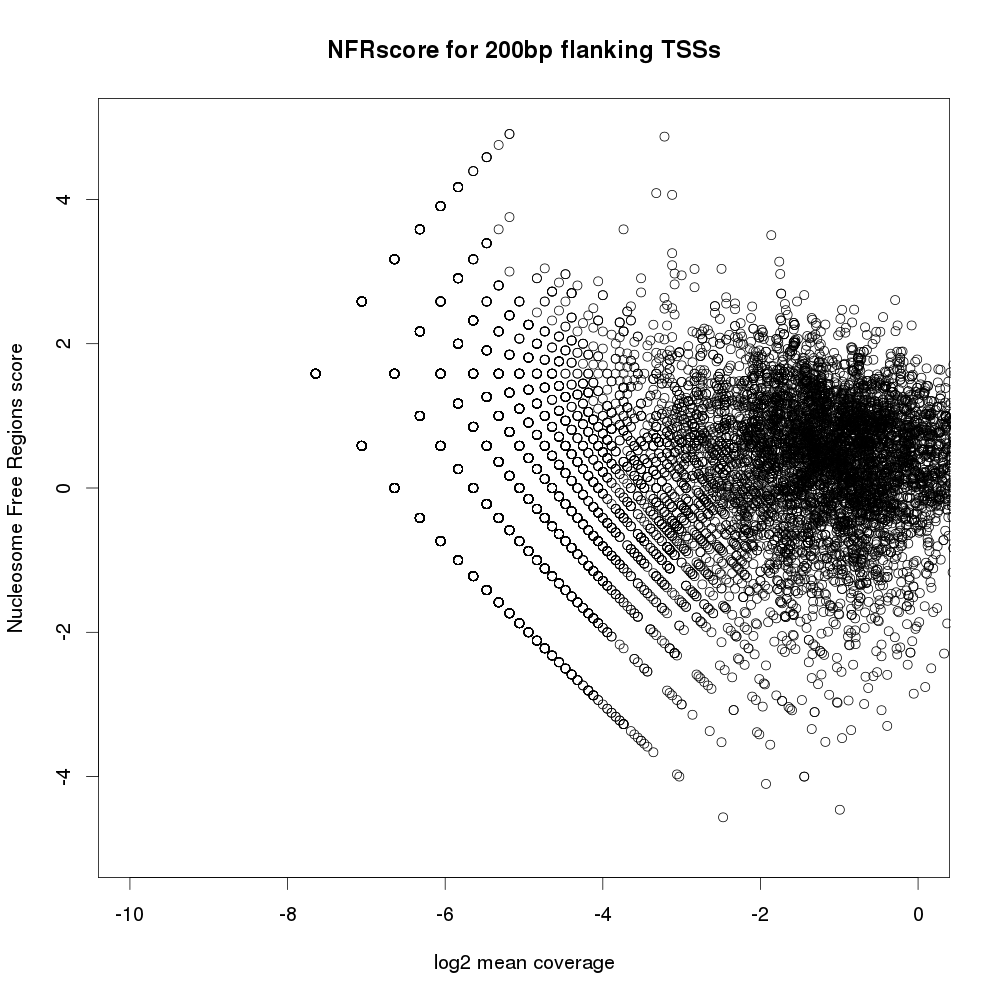

Supplement: S2 File — (ZIP) [file pone.0232332.s012.zip › nucleosome_positioning/SRR7140571_NFRscore.png]

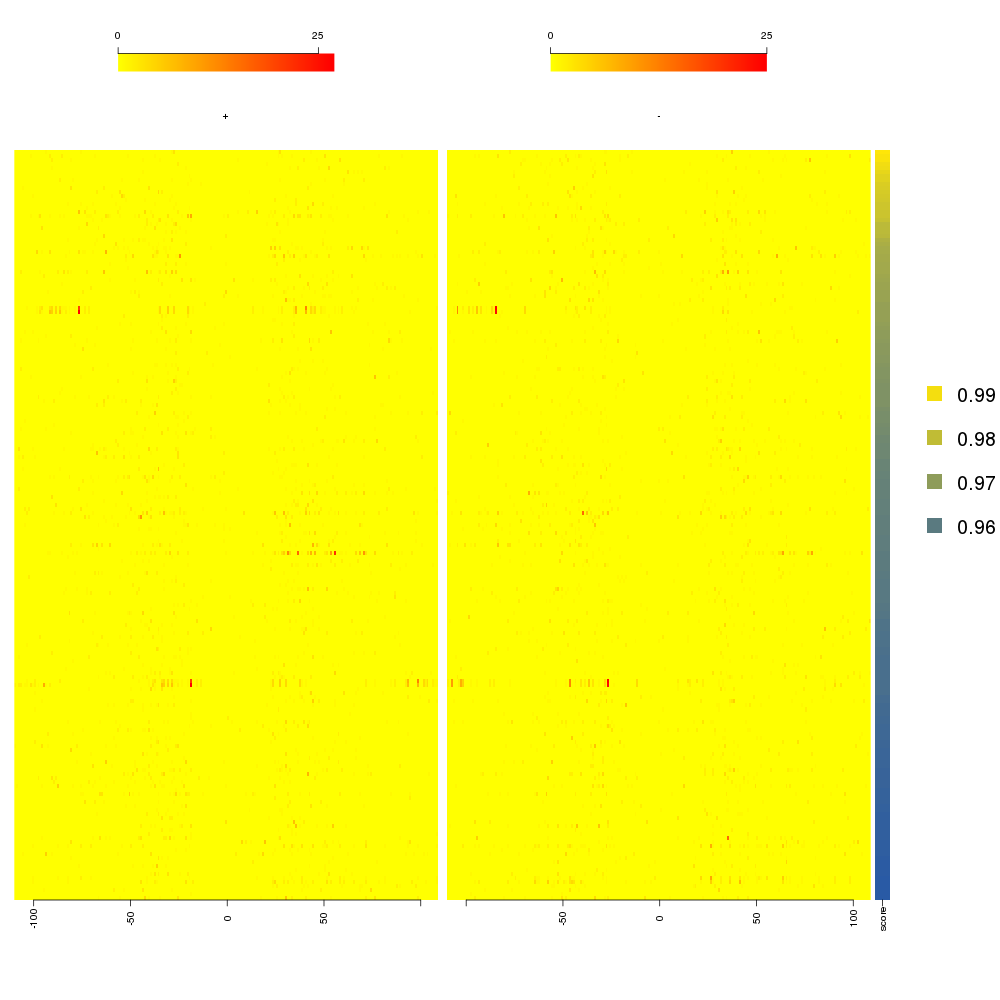

Supplement: S2 File — (ZIP) [file pone.0232332.s012.zip › nucleosome_positioning/SRR1822166_feature_aligned_heatmap.png]

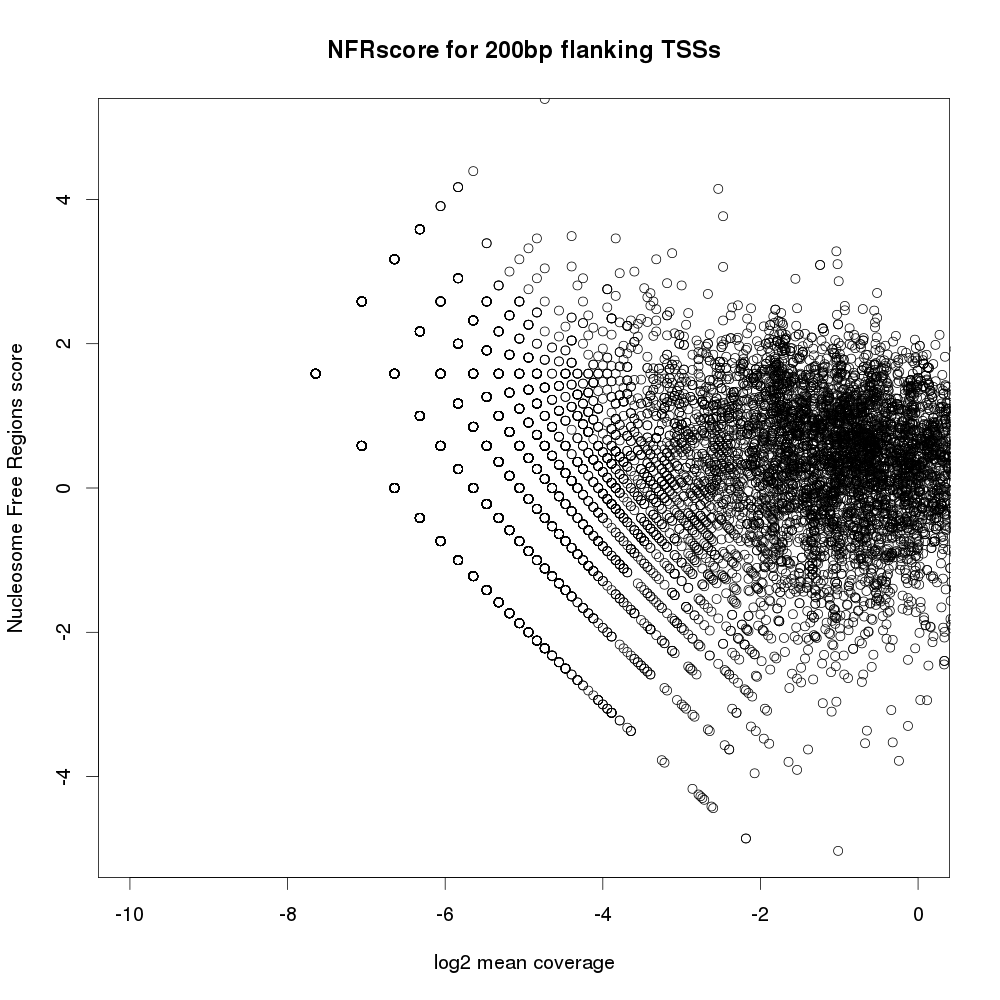

Supplement: S2 File — (ZIP) [file pone.0232332.s012.zip › nucleosome_positioning/SRR8932925_NFRscore.png]

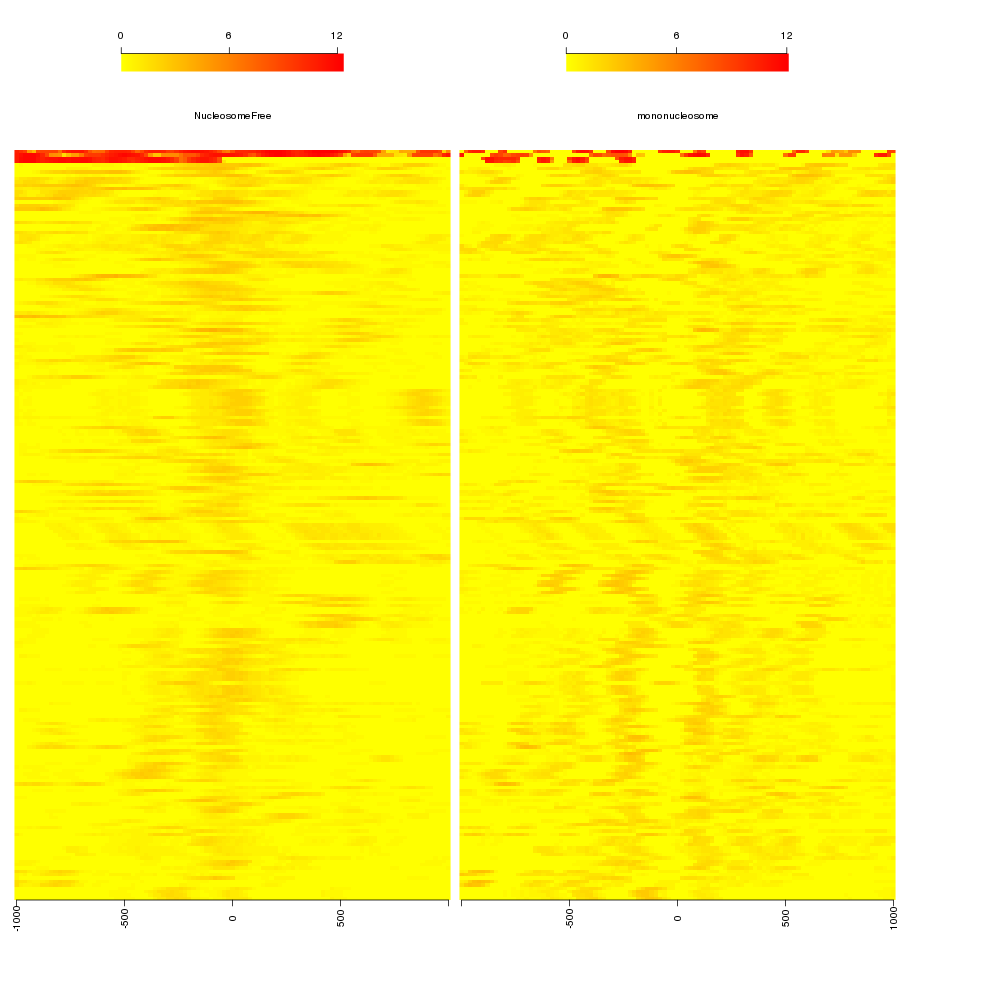

Supplement: S2 File — (ZIP) [file pone.0232332.s012.zip › nucleosome_positioning/SRR3622819_nucleosome_heatmap.png]

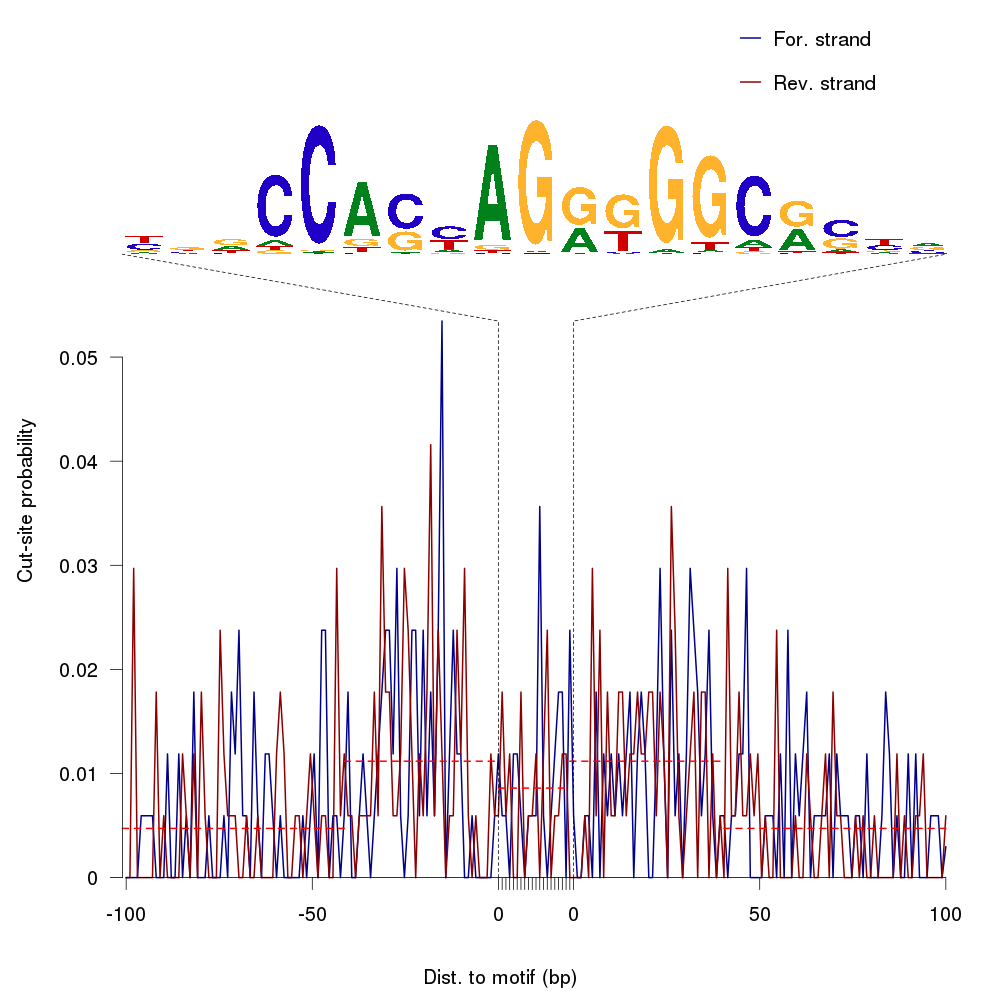

Supplement: S2 File — (ZIP) [file pone.0232332.s012.zip › nucleosome_positioning/SRR891275_footprint_plot.png]

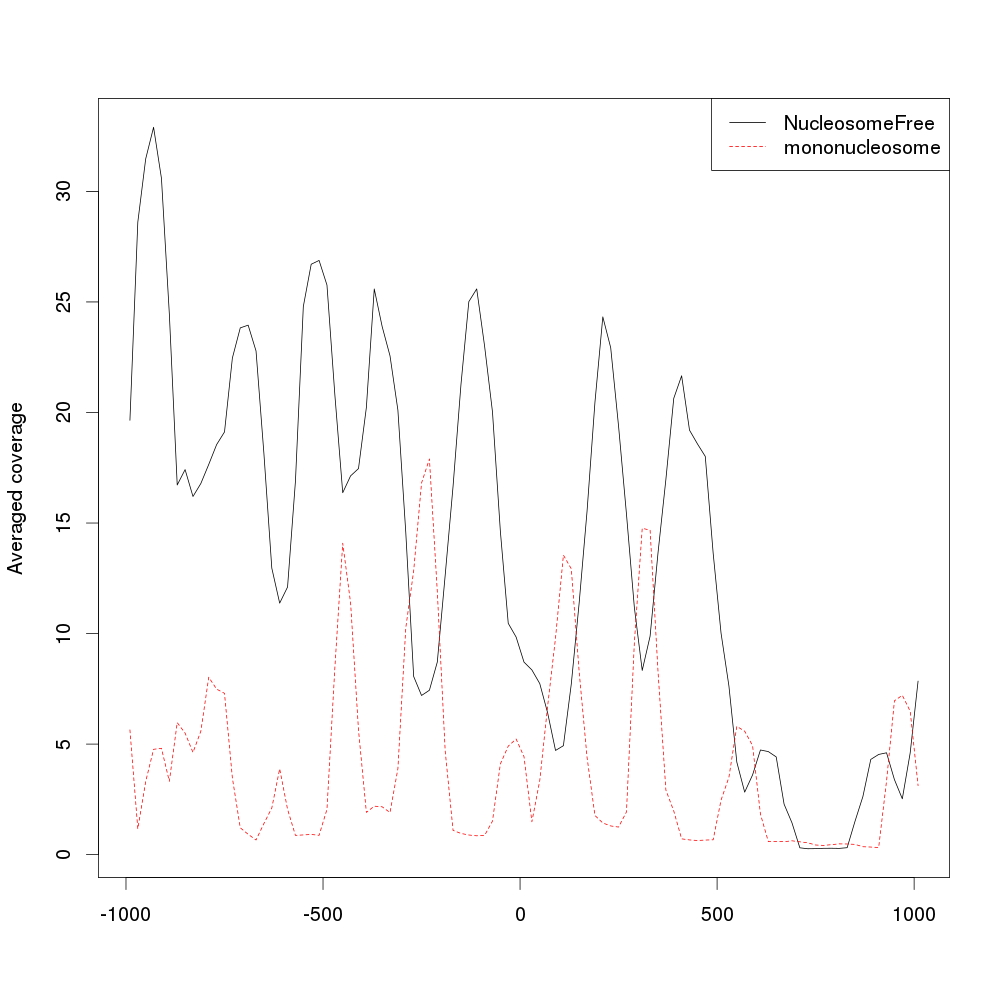

Supplement: S2 File — (ZIP) [file pone.0232332.s012.zip › nucleosome_positioning/SRR5876158_nucleosome_distribution.png]

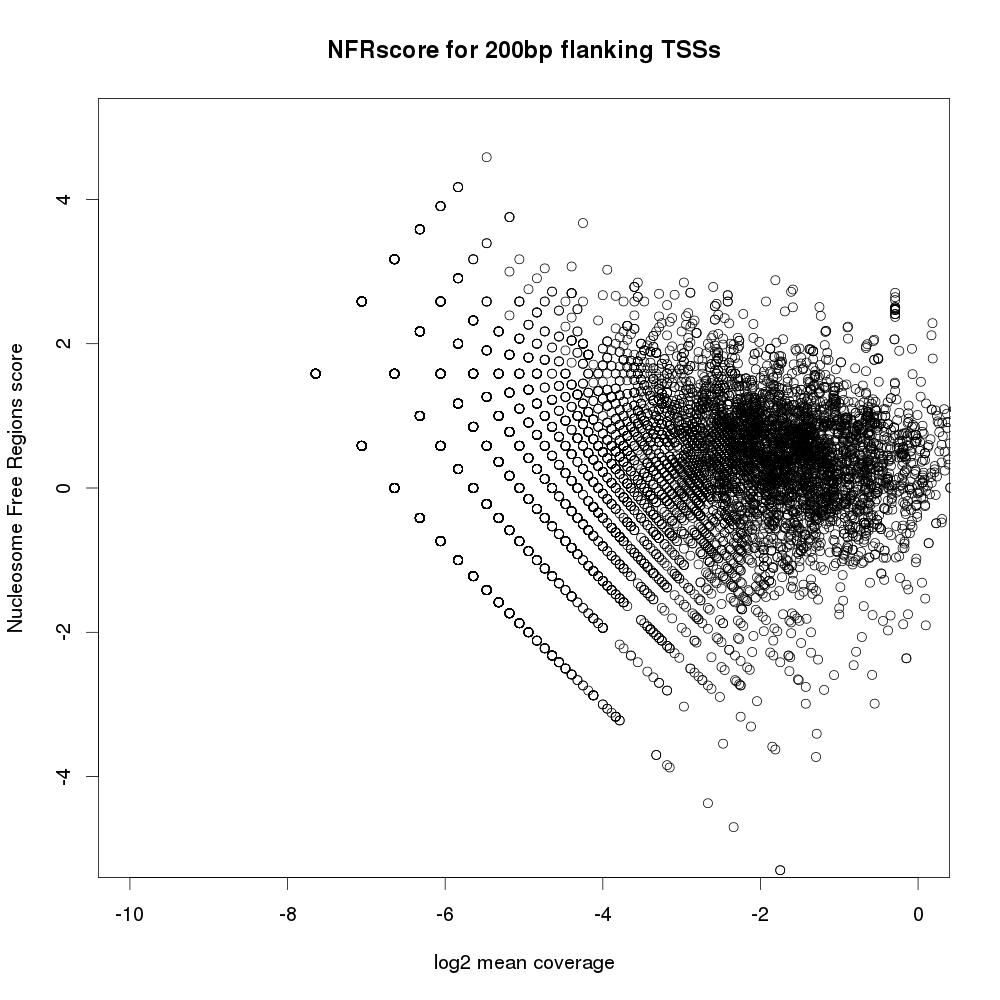

Supplement: S2 File — (ZIP) [file pone.0232332.s012.zip › nucleosome_positioning/SRR1822167_NFRscore.png]

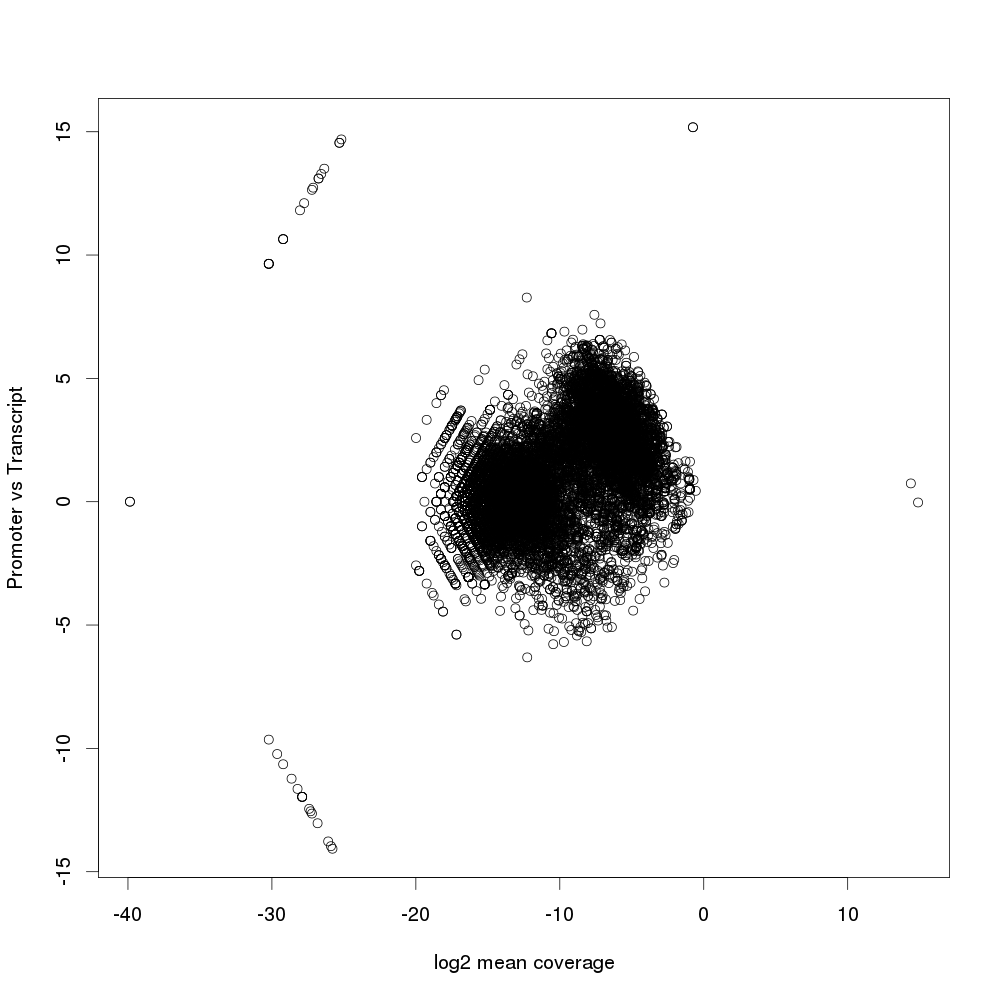

Supplement: S2 File — (ZIP) [file pone.0232332.s012.zip › nucleosome_positioning/SRR5063984_pt_score.png]

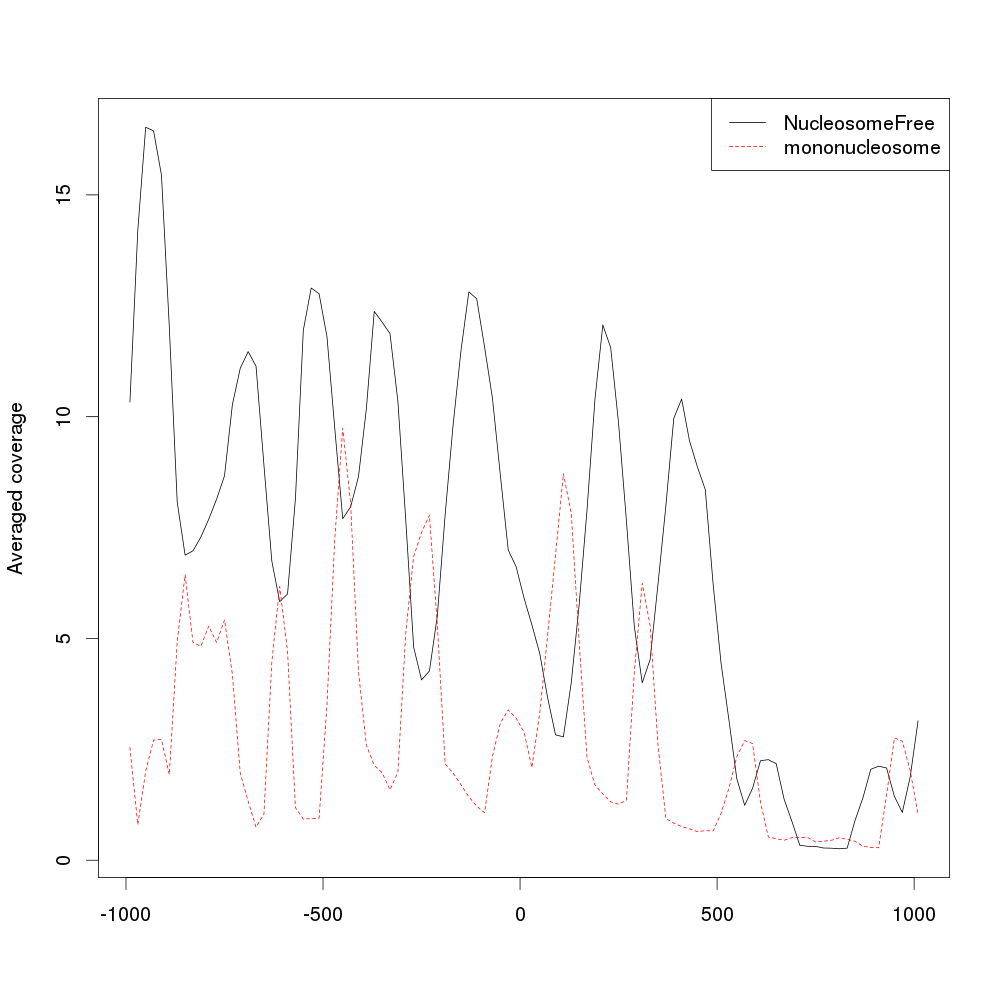

Supplement: S2 File — (ZIP) [file pone.0232332.s012.zip › nucleosome_positioning/SRR891276_nucleosome_distribution.png]

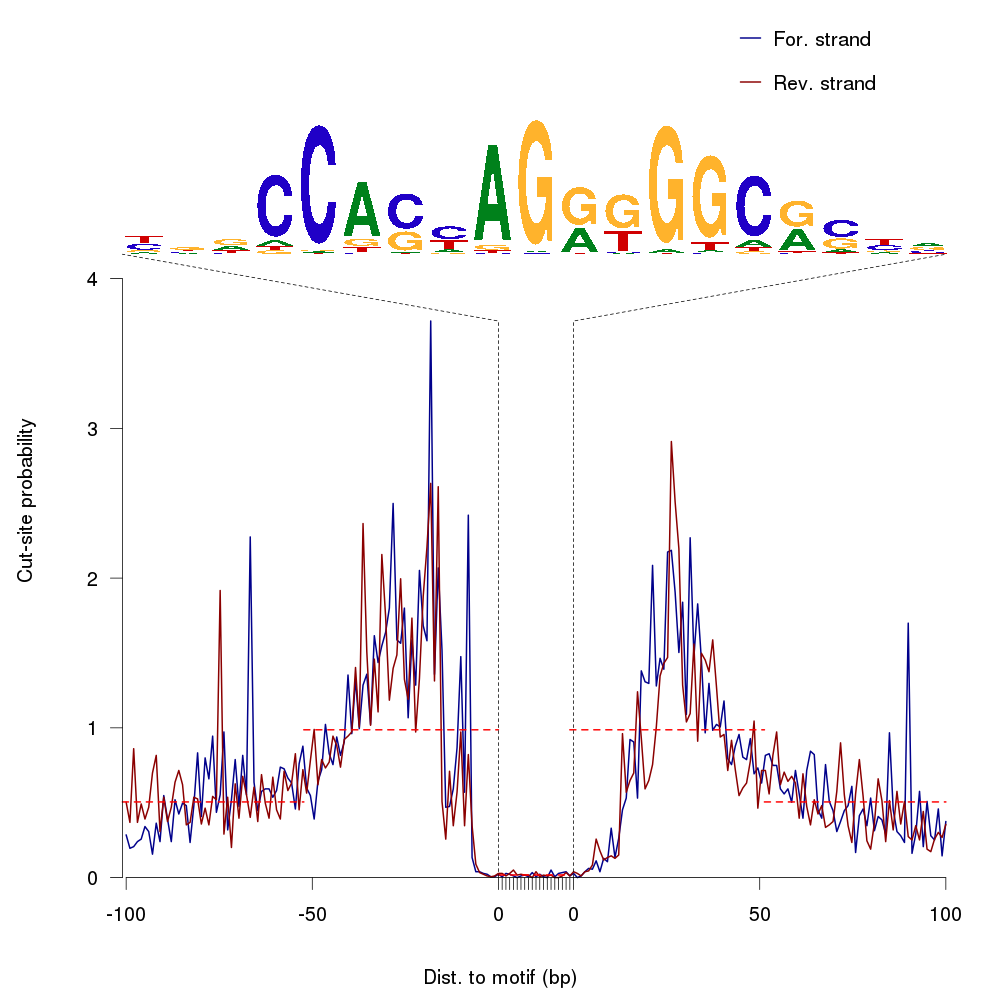

Supplement: S2 File — (ZIP) [file pone.0232332.s012.zip › nucleosome_positioning/SRX6443491_footprint_plot.png]

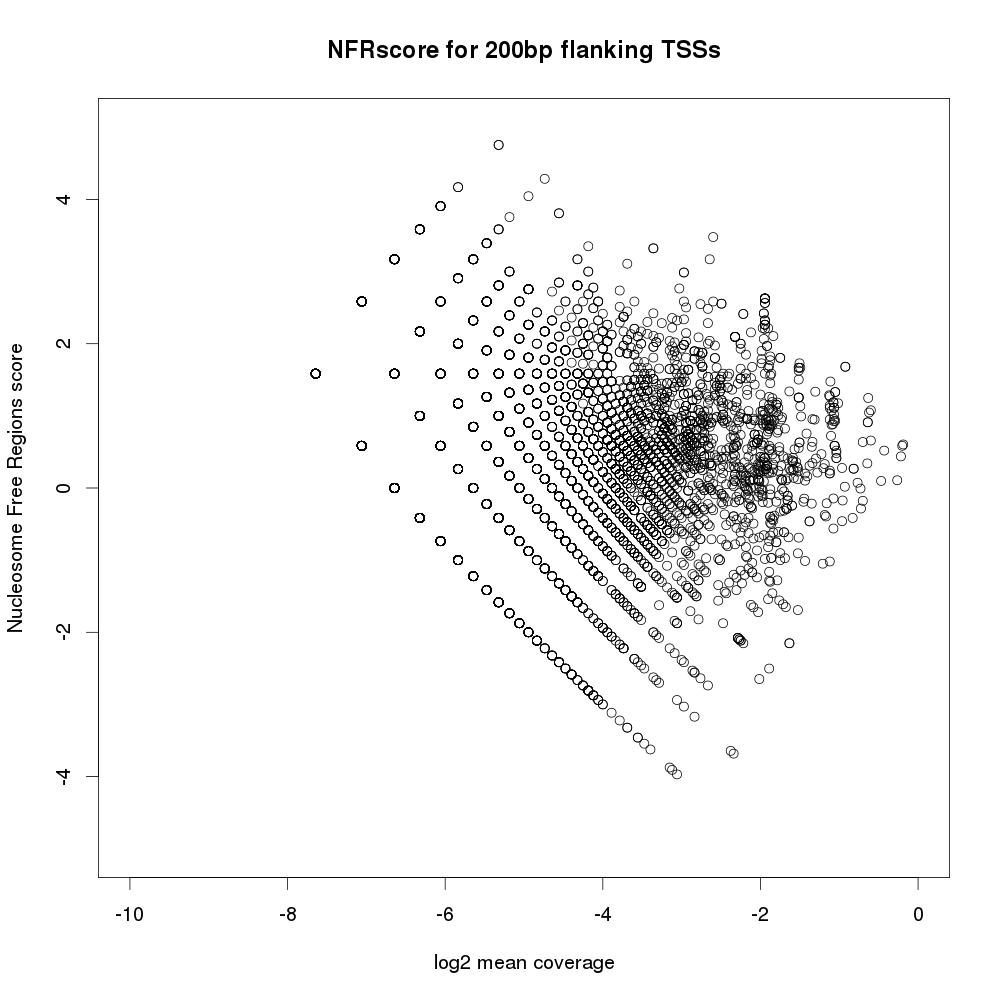

Supplement: S2 File — (ZIP) [file pone.0232332.s012.zip › nucleosome_positioning/SRR891276_NFRscore.png]

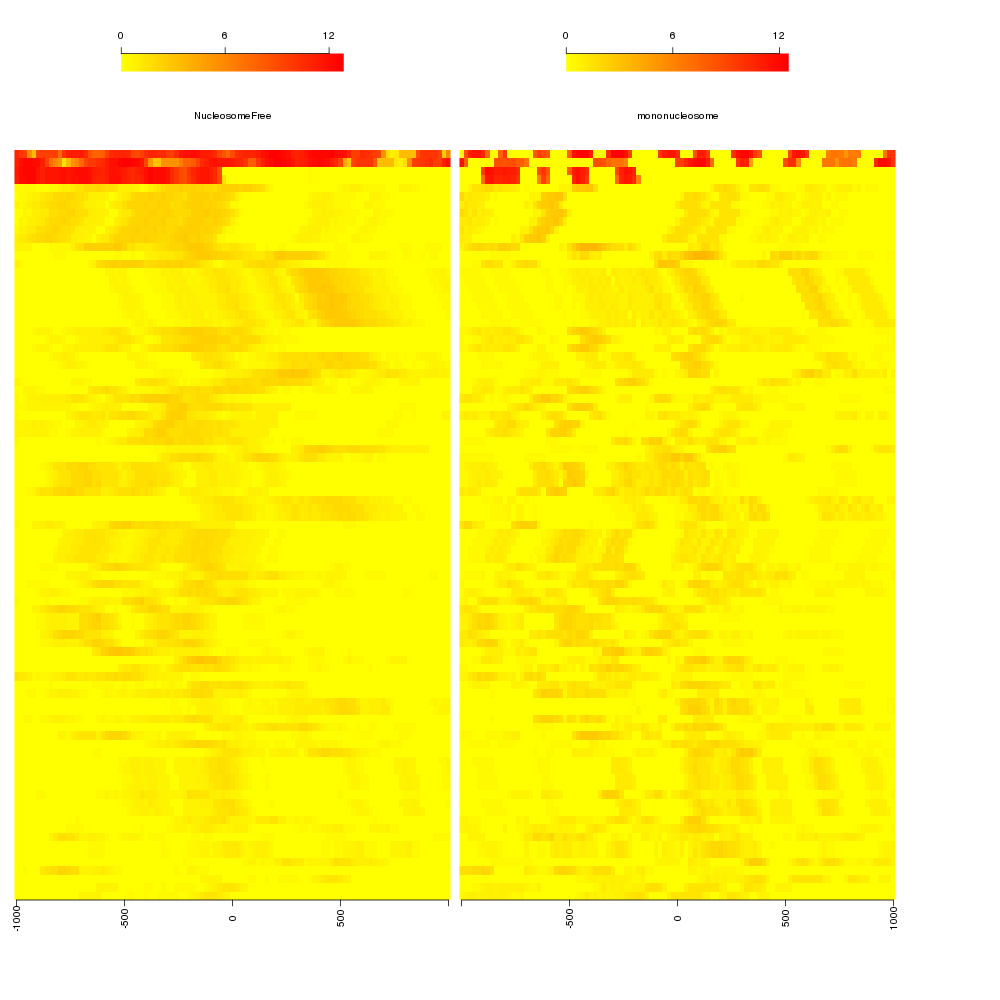

Supplement: S2 File — (ZIP) [file pone.0232332.s012.zip › nucleosome_positioning/SRR5128074_nucleosome_heatmap.png]

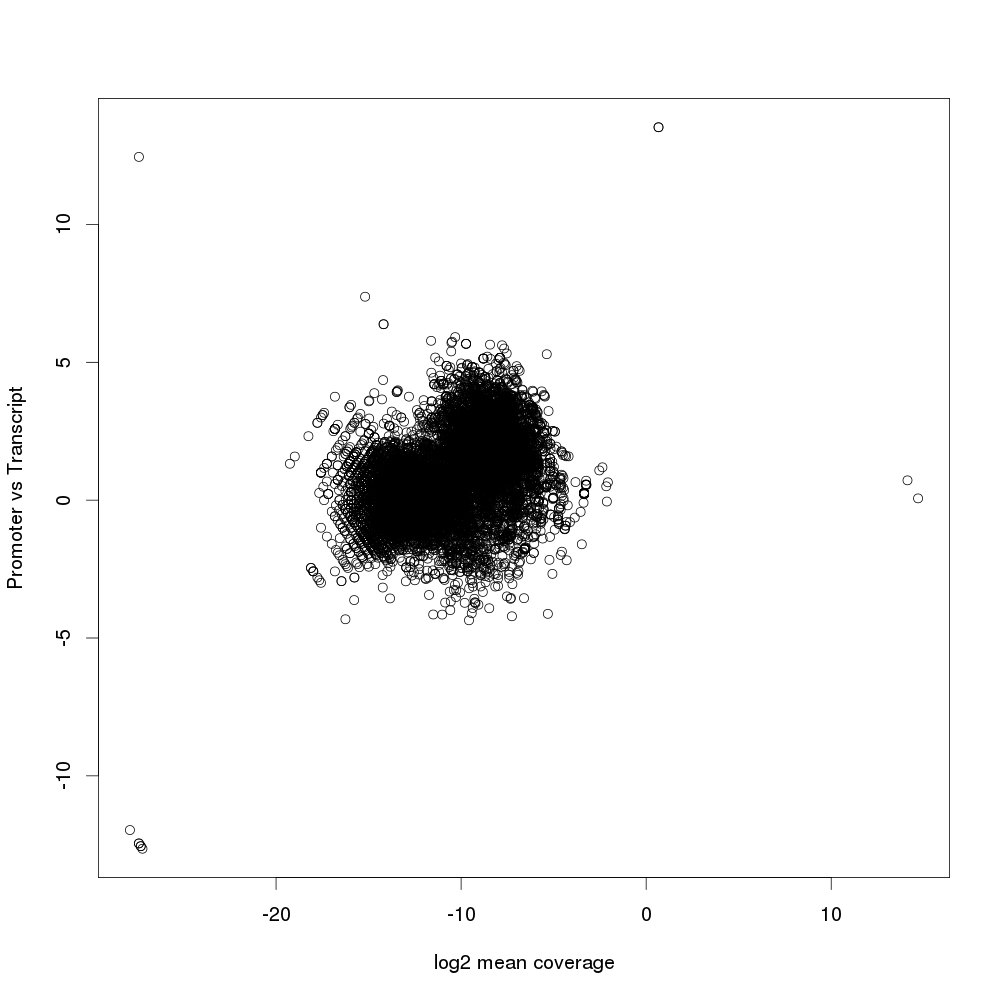

Supplement: S2 File — (ZIP) [file pone.0232332.s012.zip › nucleosome_positioning/SRR5063985_pt_score.png]

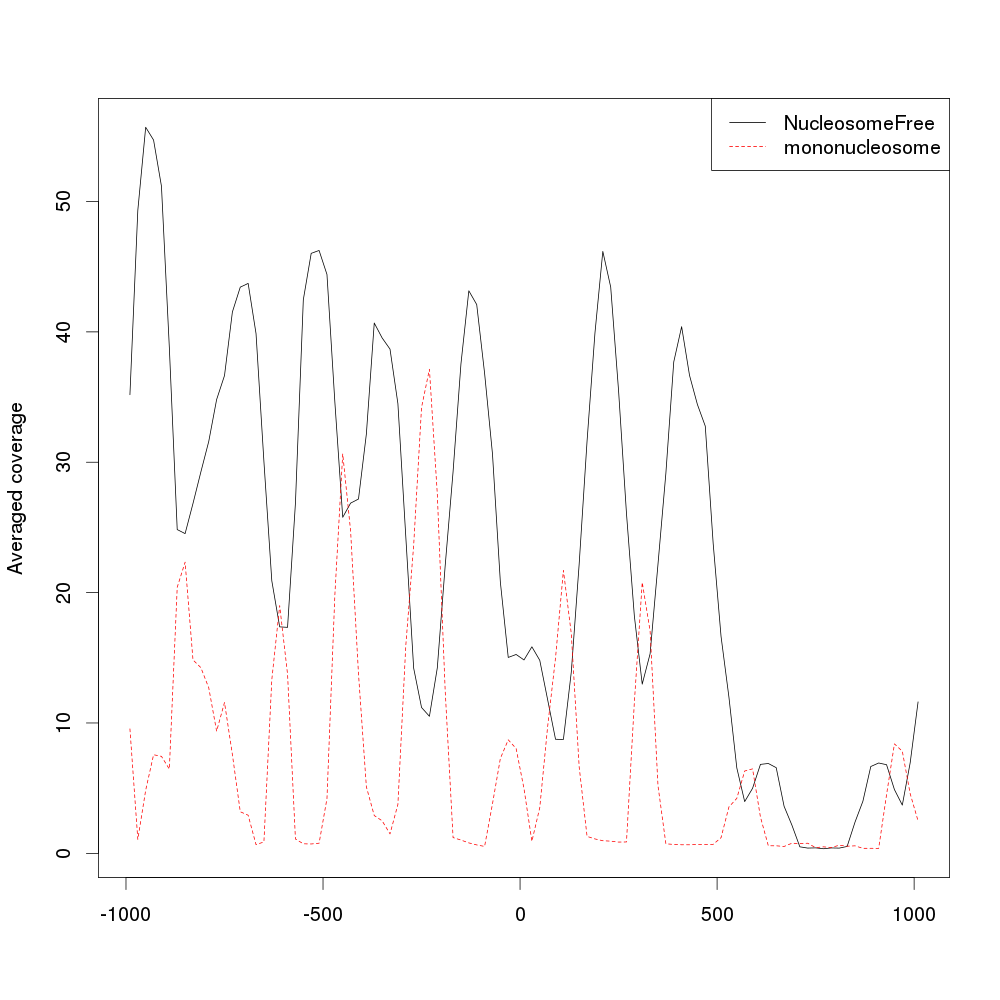

Supplement: S2 File — (ZIP) [file pone.0232332.s012.zip › nucleosome_positioning/SRR3622819_nucleosome_distribution.png]

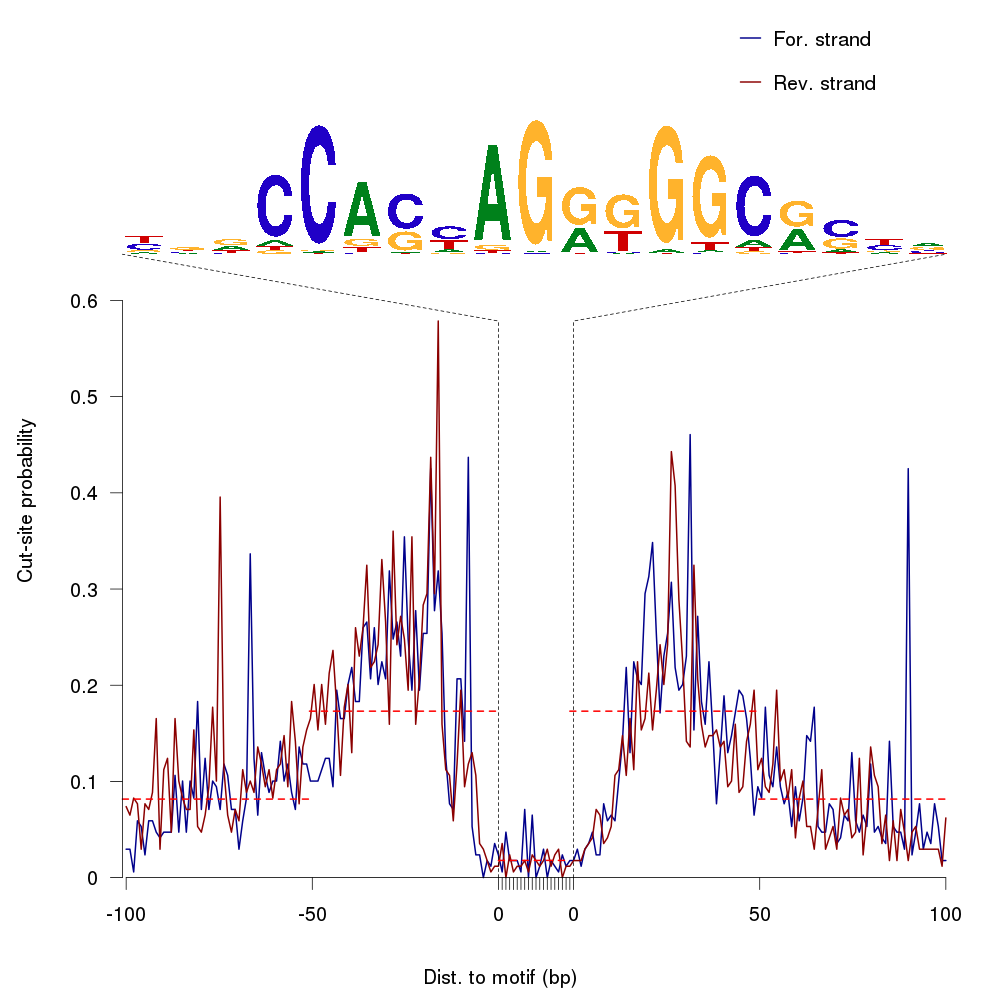

Supplement: S2 File — (ZIP) [file pone.0232332.s012.zip › nucleosome_positioning/SRR5063985_footprint_plot.png]

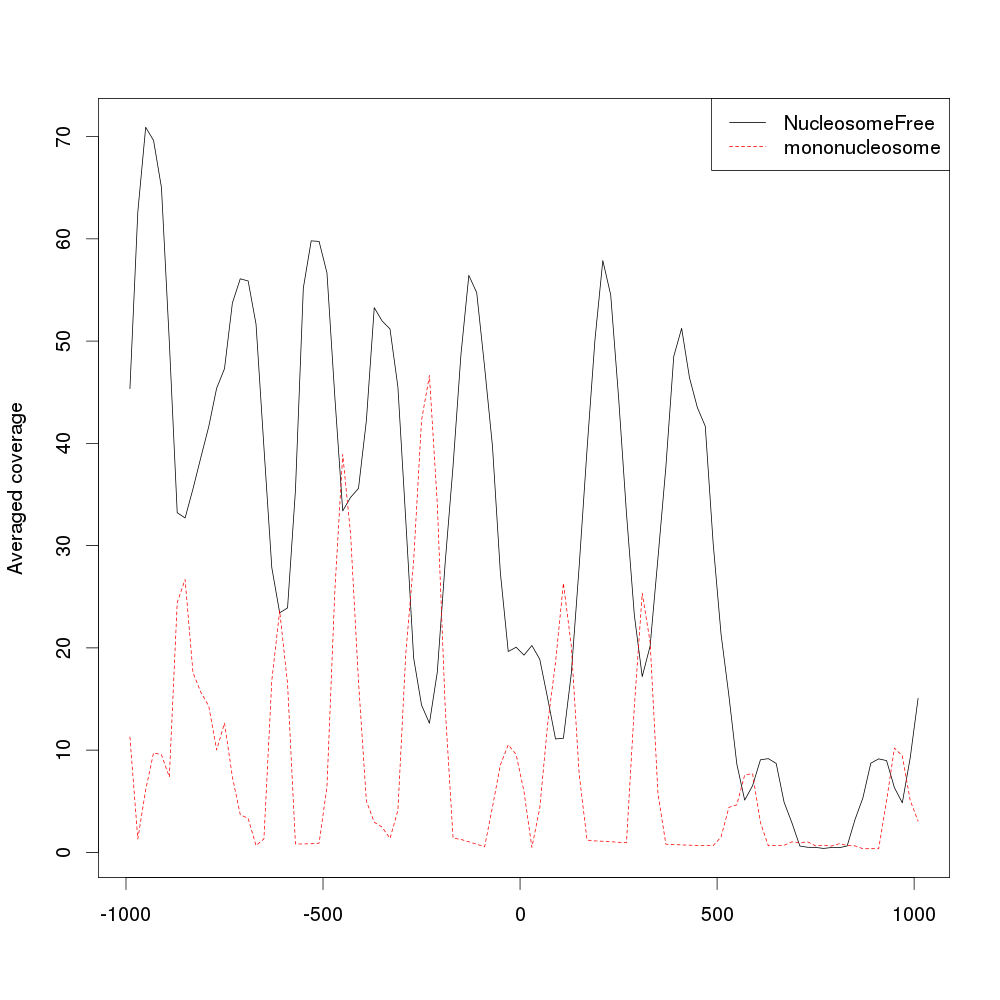

Supplement: S2 File — (ZIP) [file pone.0232332.s012.zip › nucleosome_positioning/SRR3622817_nucleosome_distribution.png]

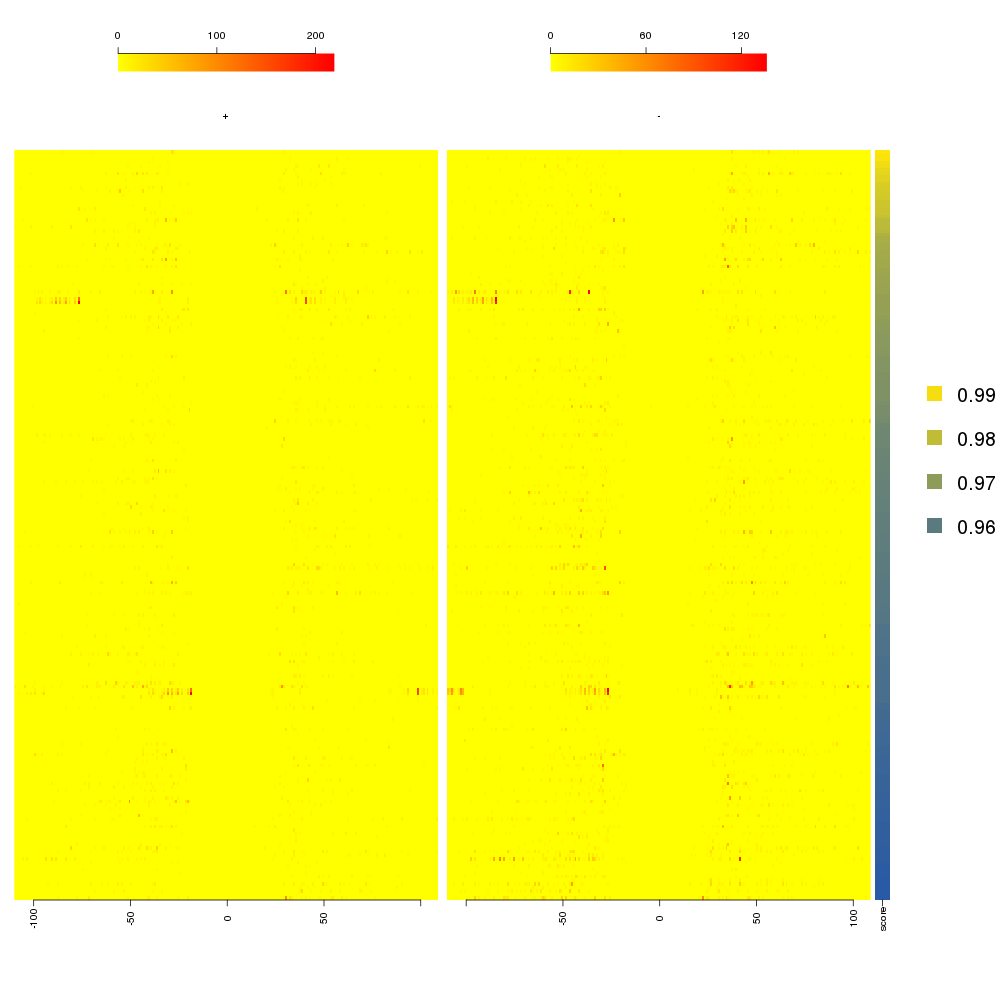

Supplement: S2 File — (ZIP) [file pone.0232332.s012.zip › nucleosome_positioning/SRX6443490_feature_aligned_heatmap.png]

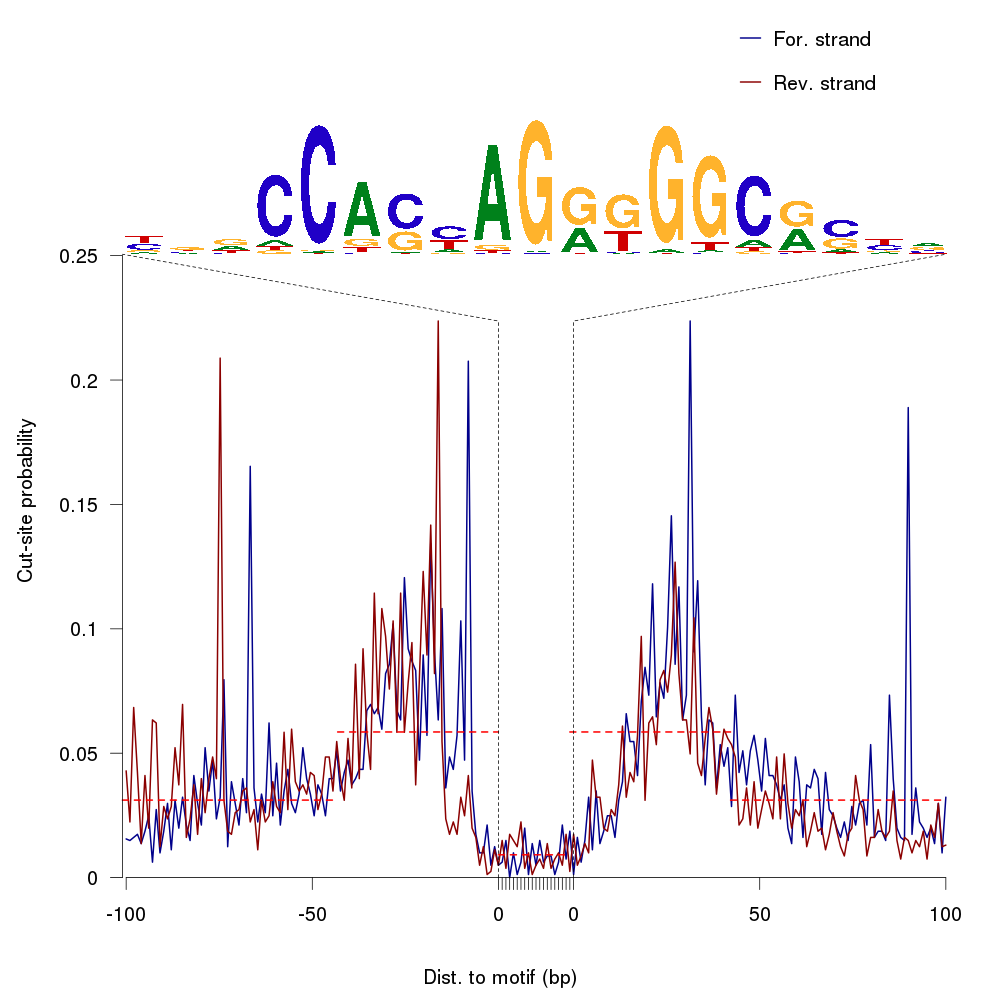

Supplement: S2 File — (ZIP) [file pone.0232332.s012.zip › nucleosome_positioning/SRR8932925_footprint_plot.png]

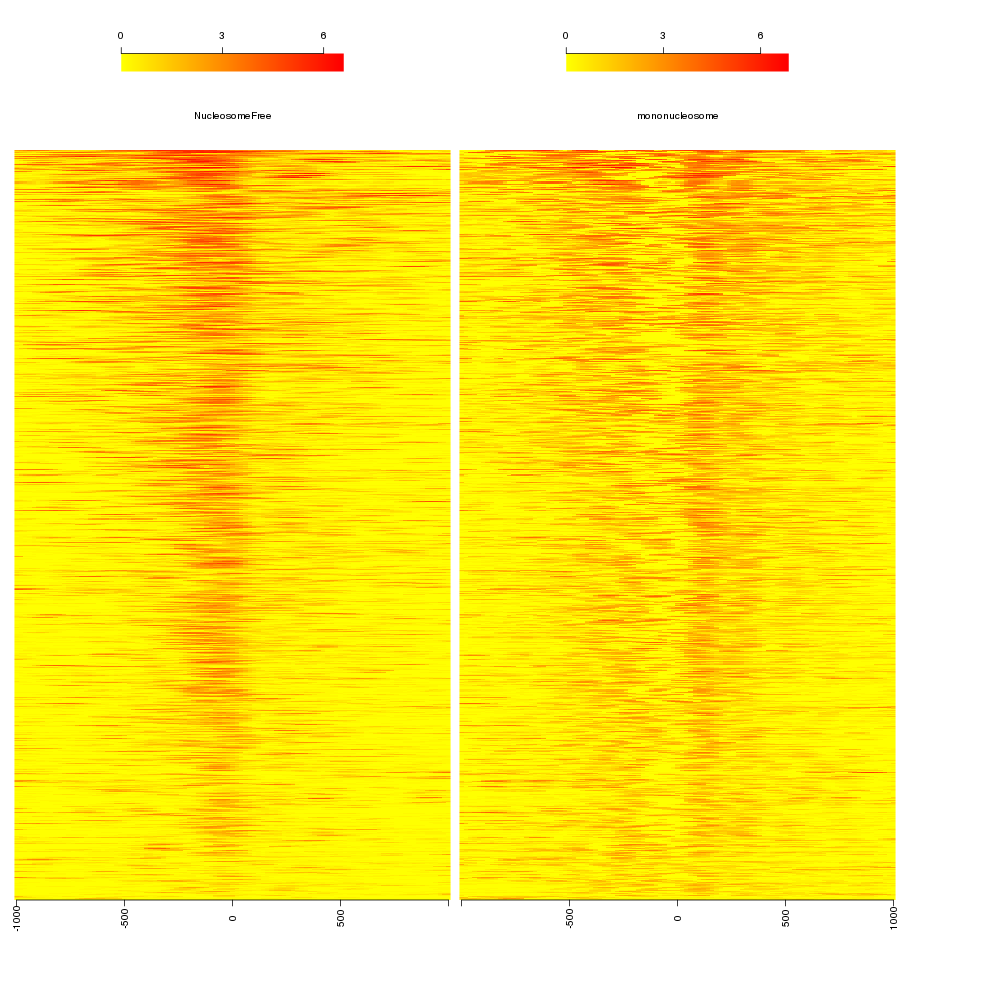

Supplement: S2 File — (ZIP) [file pone.0232332.s012.zip › nucleosome_positioning/SRX6443488_nucleosome_heatmap.png]

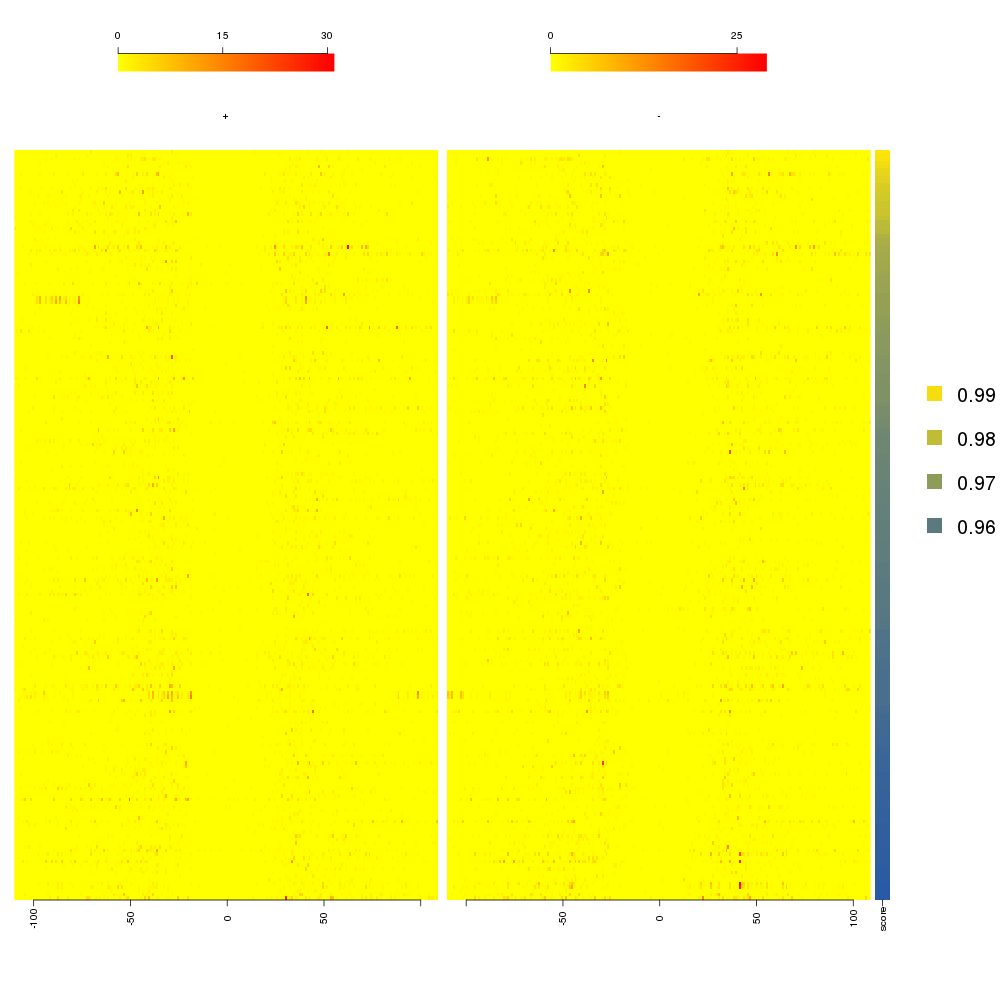

Supplement: S2 File — (ZIP) [file pone.0232332.s012.zip › nucleosome_positioning/SRR6216226_feature_aligned_heatmap.png]

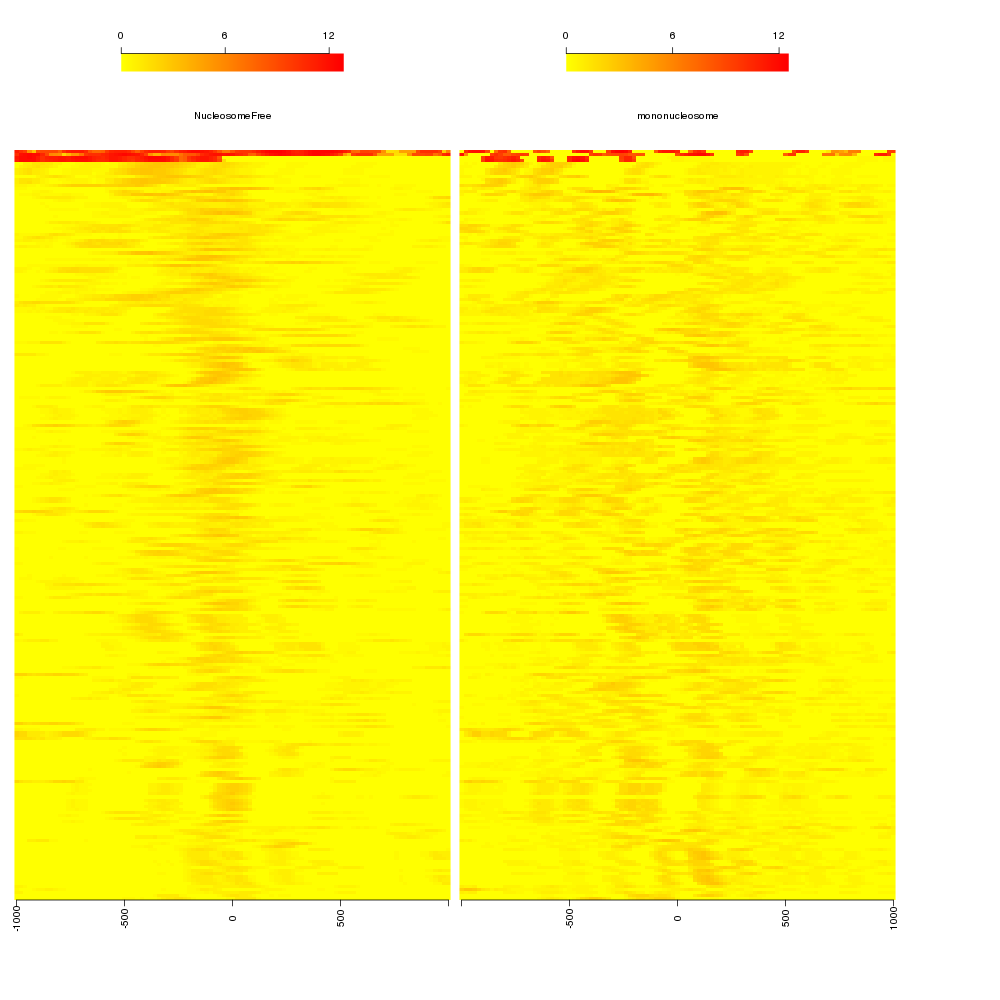

Supplement: S2 File — (ZIP) [file pone.0232332.s012.zip › nucleosome_positioning/SRR1822168_nucleosome_heatmap.png]

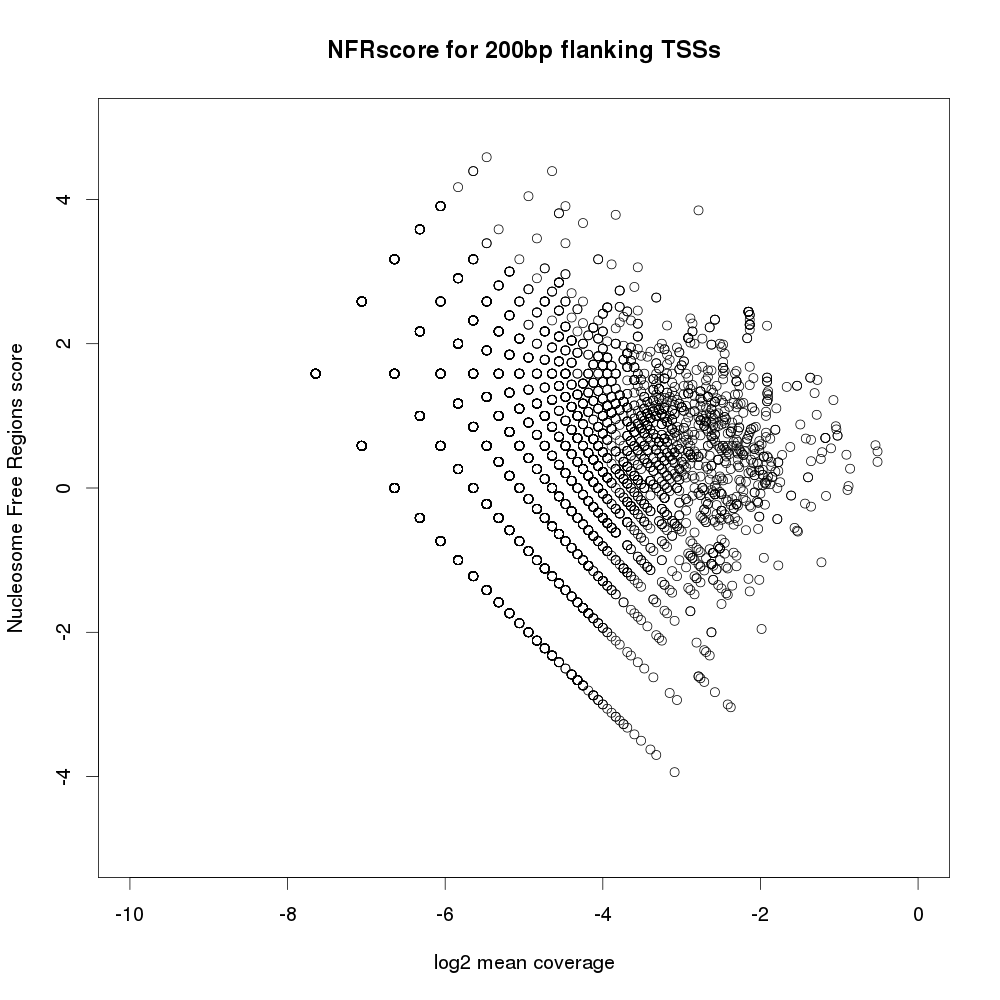

Supplement: S2 File — (ZIP) [file pone.0232332.s012.zip › nucleosome_positioning/SRR891275_NFRscore.png]

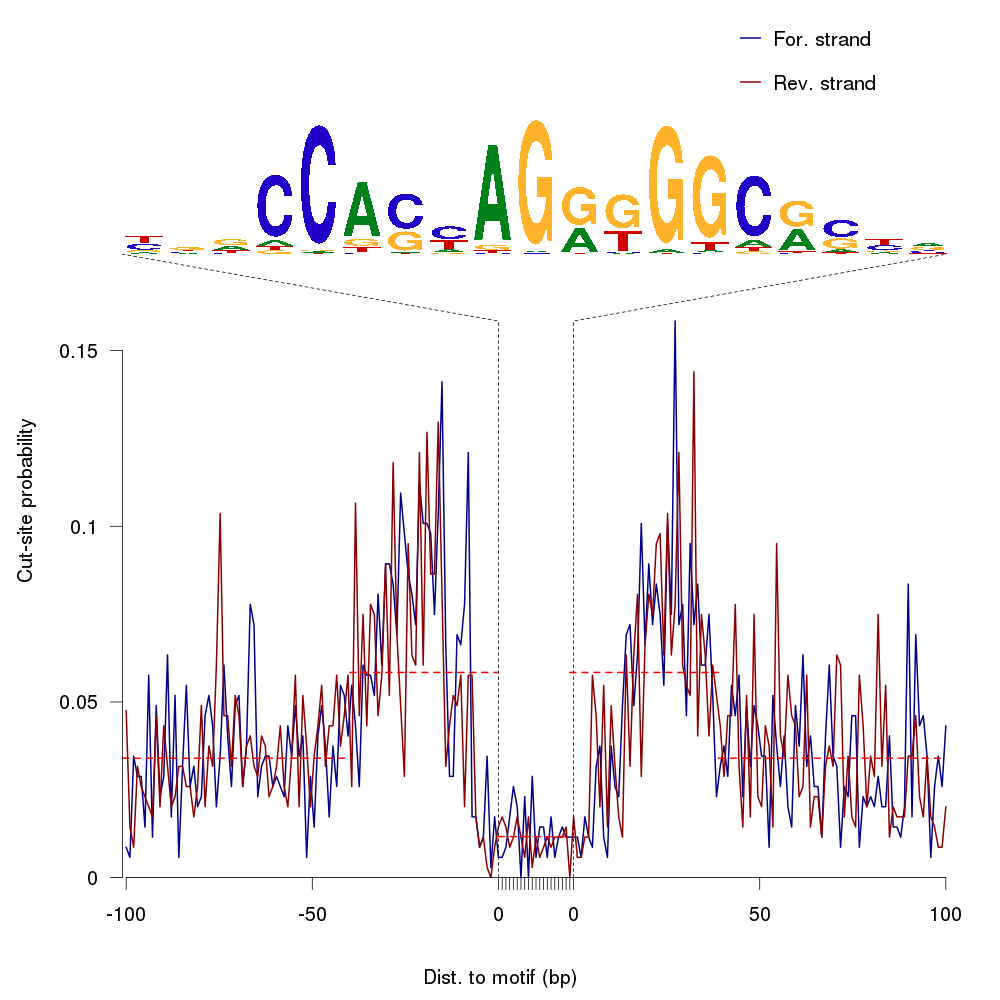

Supplement: S2 File — (ZIP) [file pone.0232332.s012.zip › nucleosome_positioning/SRR3622819_footprint_plot.png]

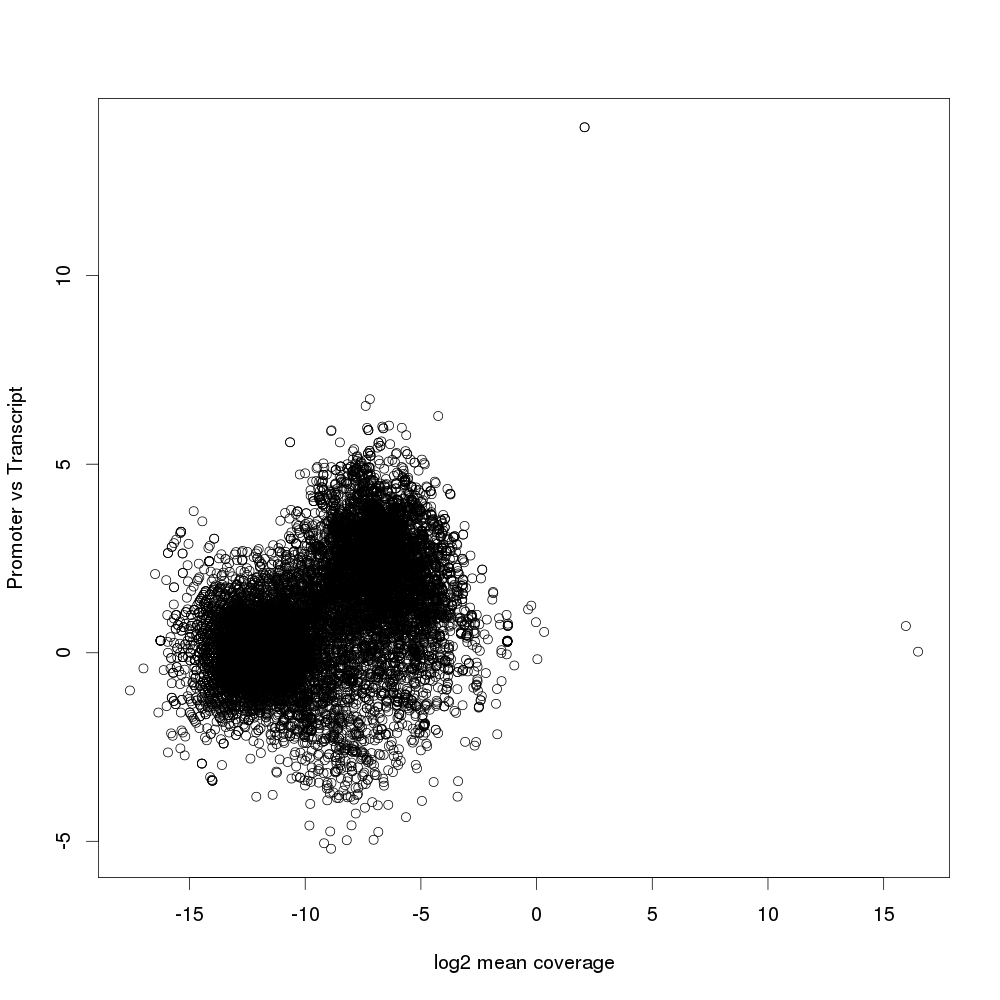

Supplement: S2 File — (ZIP) [file pone.0232332.s012.zip › nucleosome_positioning/SRR5063986_pt_score.png]

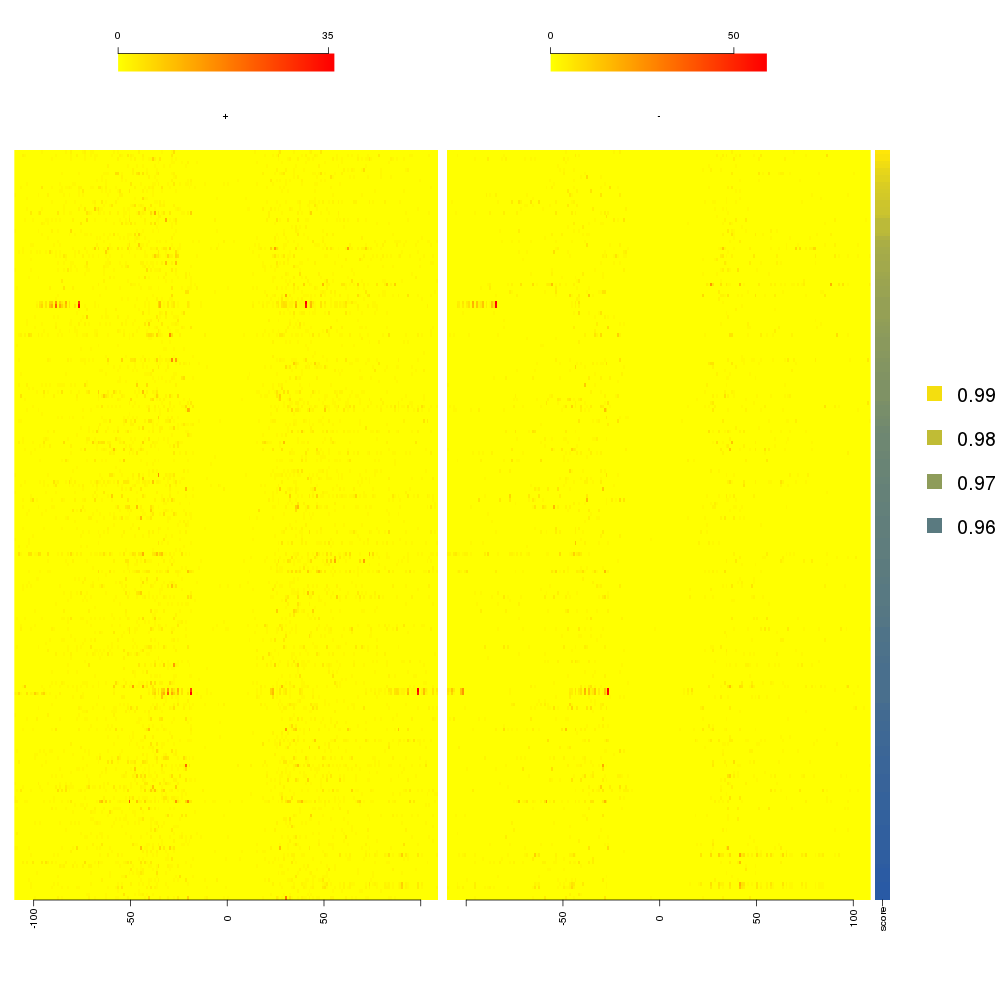

Supplement: S2 File — (ZIP) [file pone.0232332.s012.zip › nucleosome_positioning/SRR5063986_feature_aligned_heatmap.png]

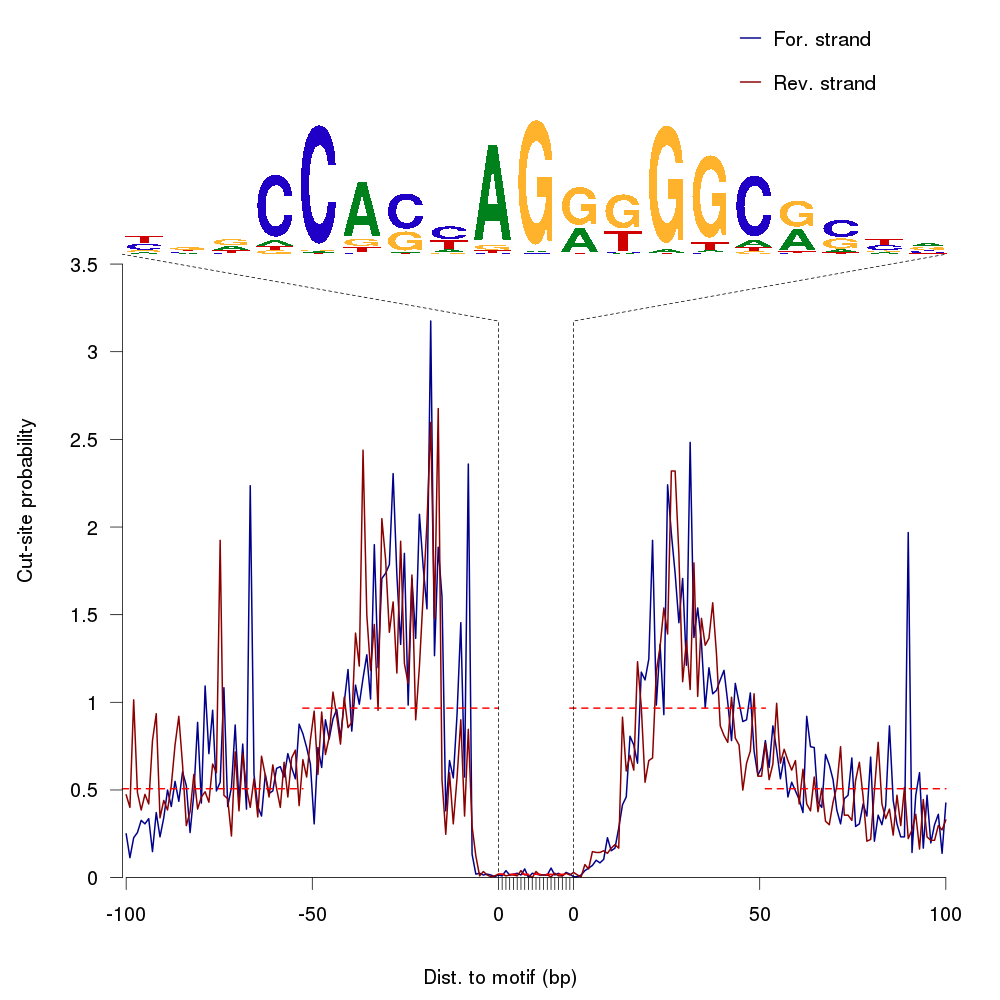

Supplement: S2 File — (ZIP) [file pone.0232332.s012.zip › nucleosome_positioning/SRX6443489_footprint_plot.png]

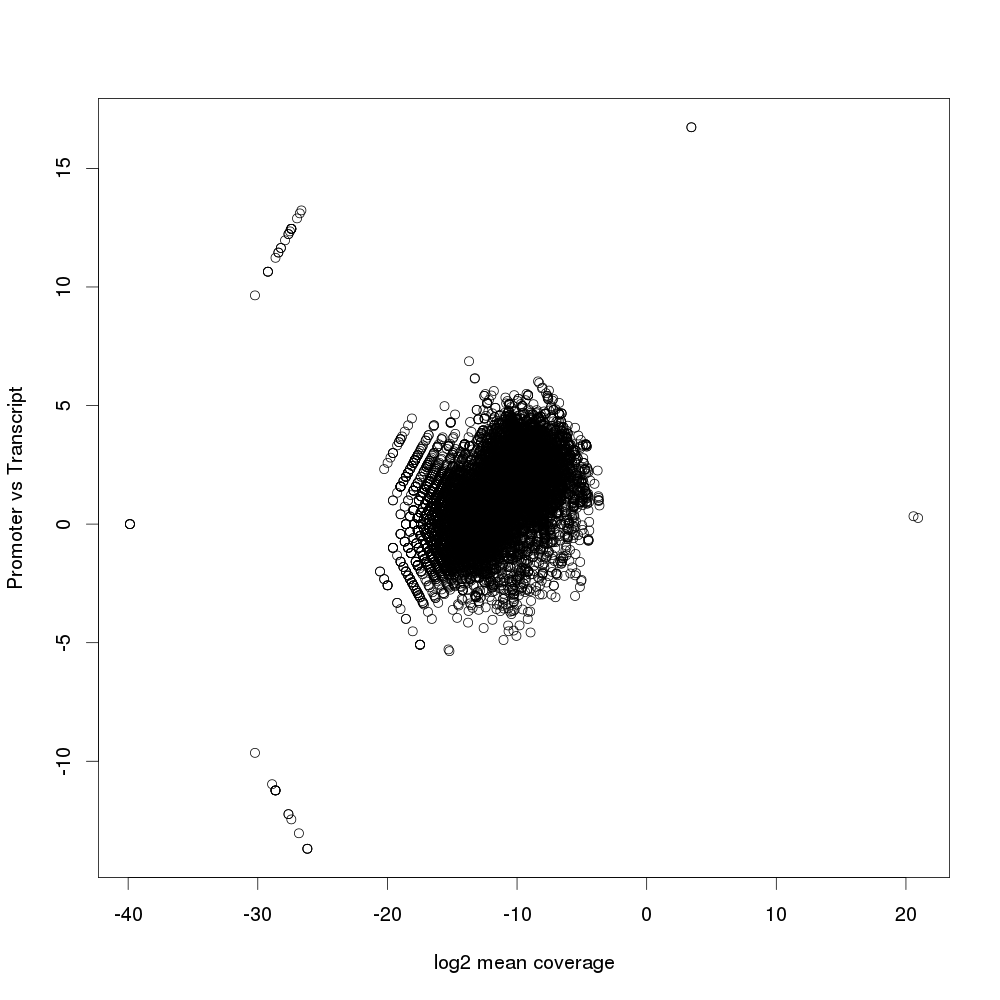

Supplement: S2 File — (ZIP) [file pone.0232332.s012.zip › nucleosome_positioning/SRR1822167_pt_score.png]

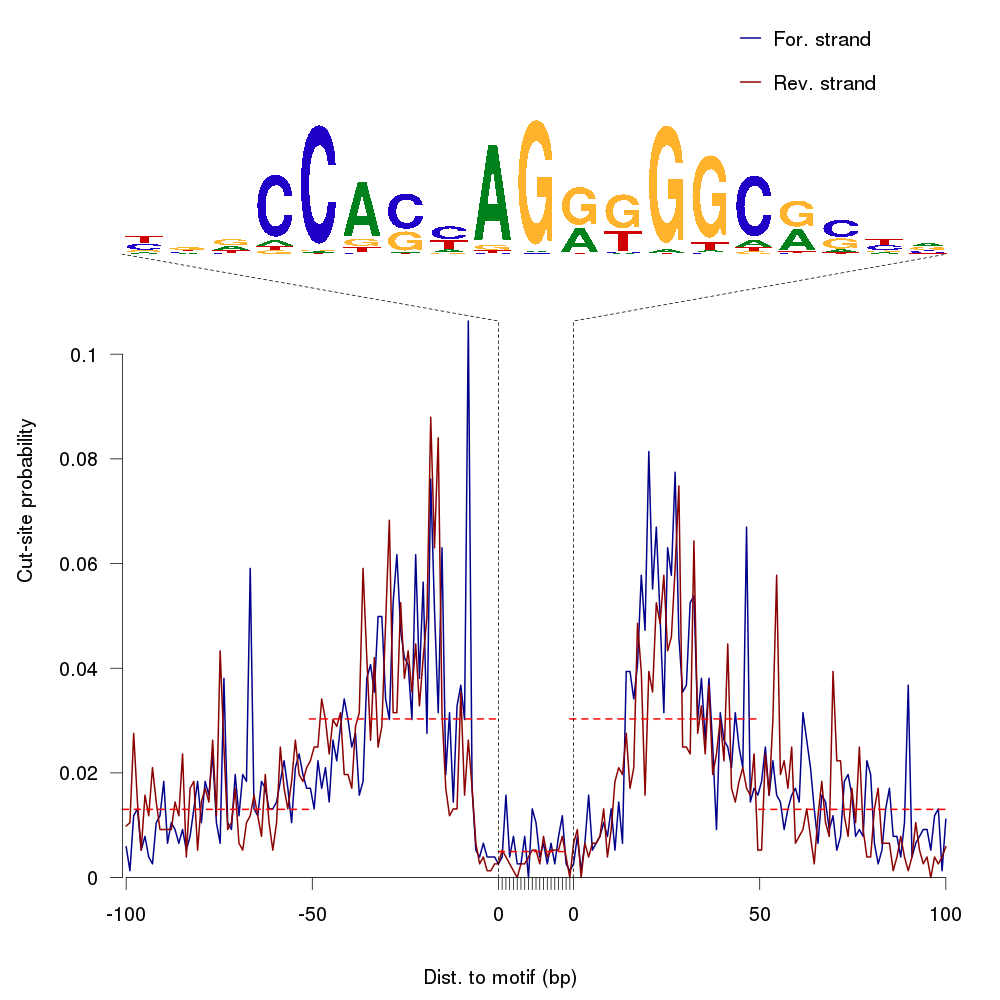

Supplement: S2 File — (ZIP) [file pone.0232332.s012.zip › nucleosome_positioning/SRR1822167_footprint_plot.png]

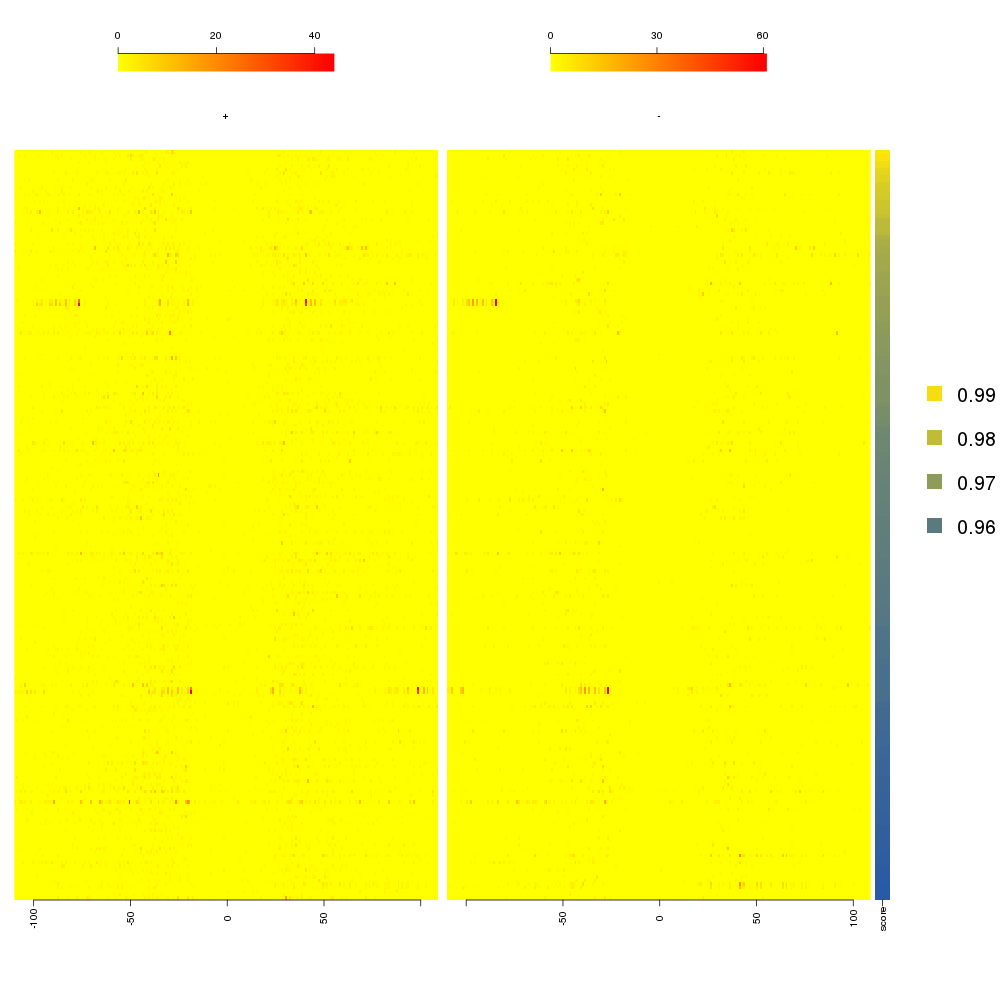

Supplement: S2 File — (ZIP) [file pone.0232332.s012.zip › nucleosome_positioning/SRR5063984_feature_aligned_heatmap.png]

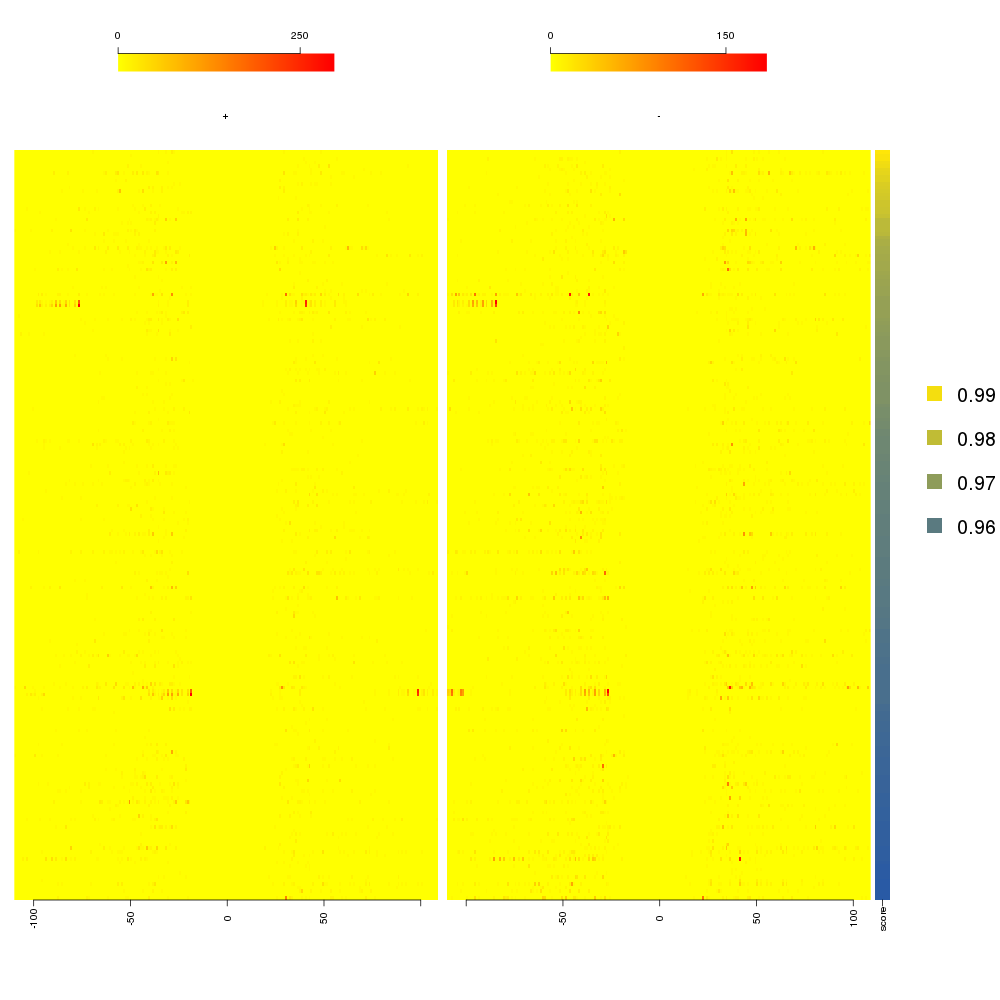

Supplement: S2 File — (ZIP) [file pone.0232332.s012.zip › nucleosome_positioning/SRX6443488_feature_aligned_heatmap.png]

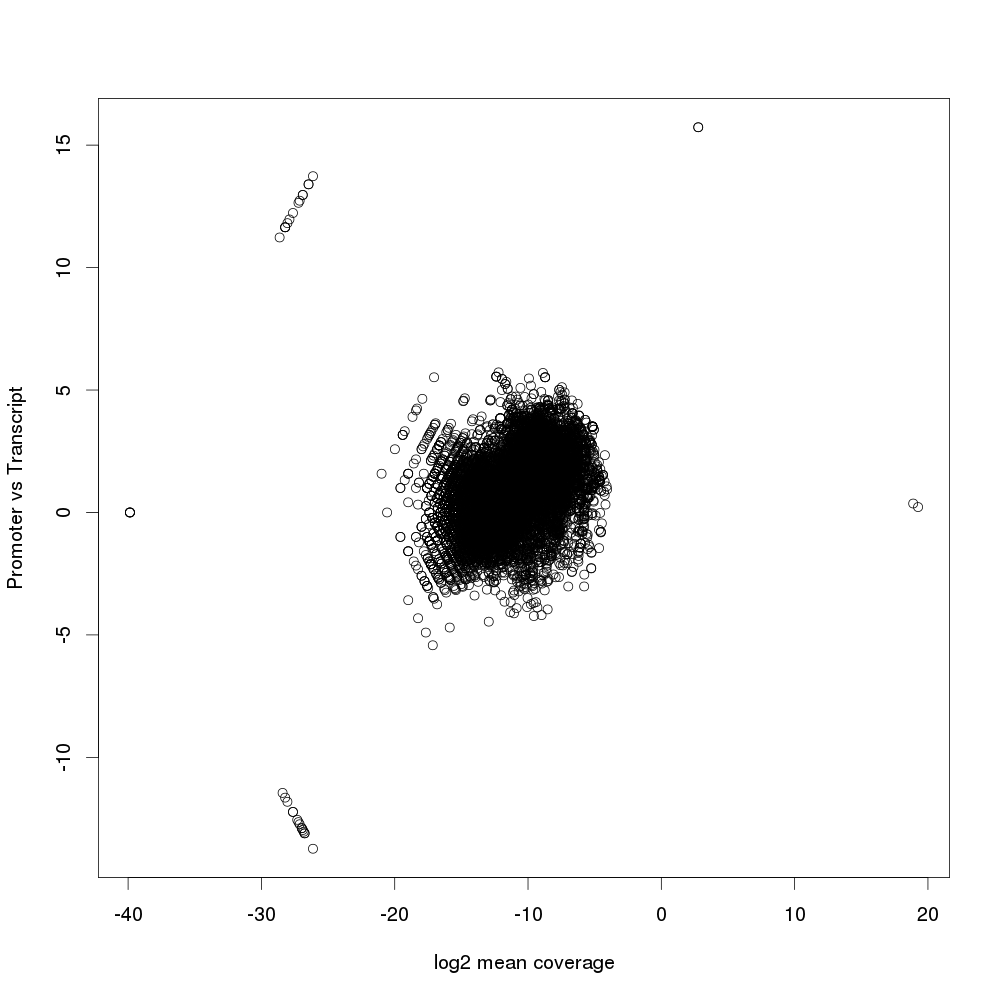

Supplement: S2 File — (ZIP) [file pone.0232332.s012.zip › nucleosome_positioning/SRR1822166_pt_score.png]

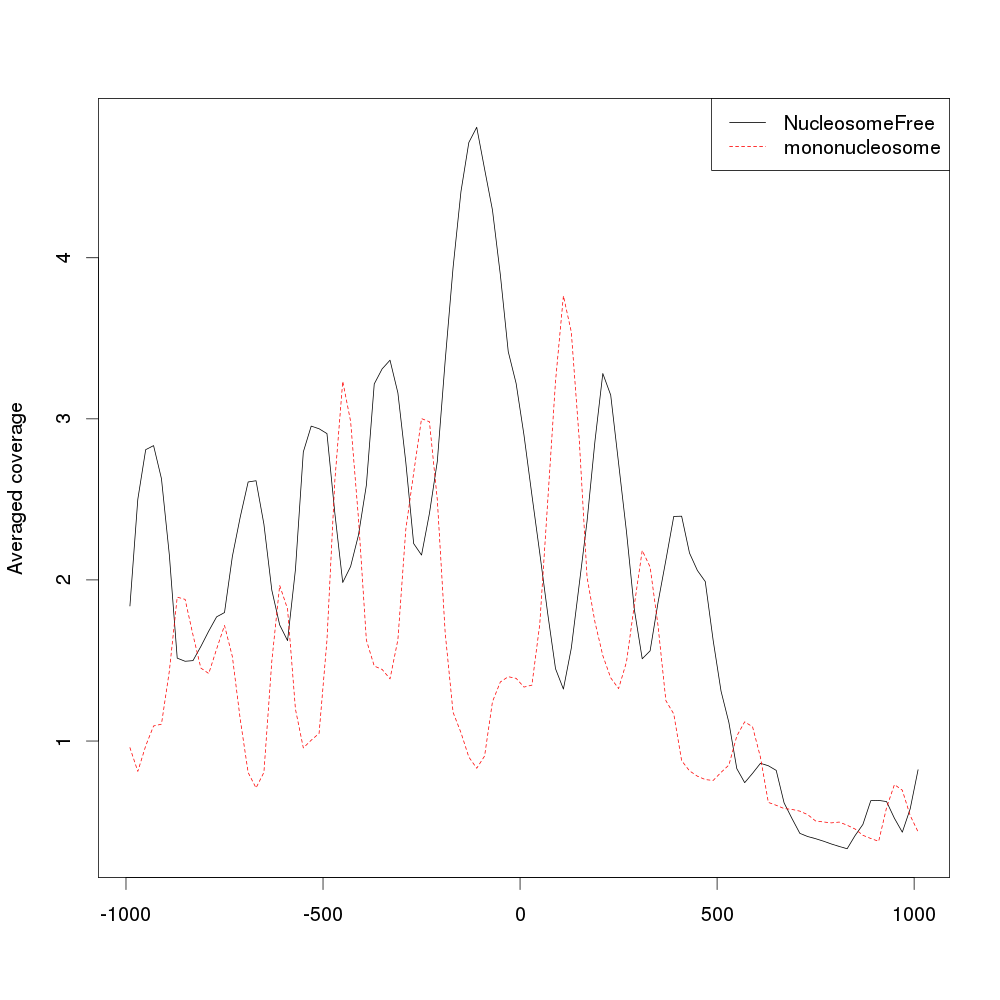

Supplement: S2 File — (ZIP) [file pone.0232332.s012.zip › nucleosome_positioning/SRR5007259_nucleosome_distribution.png]

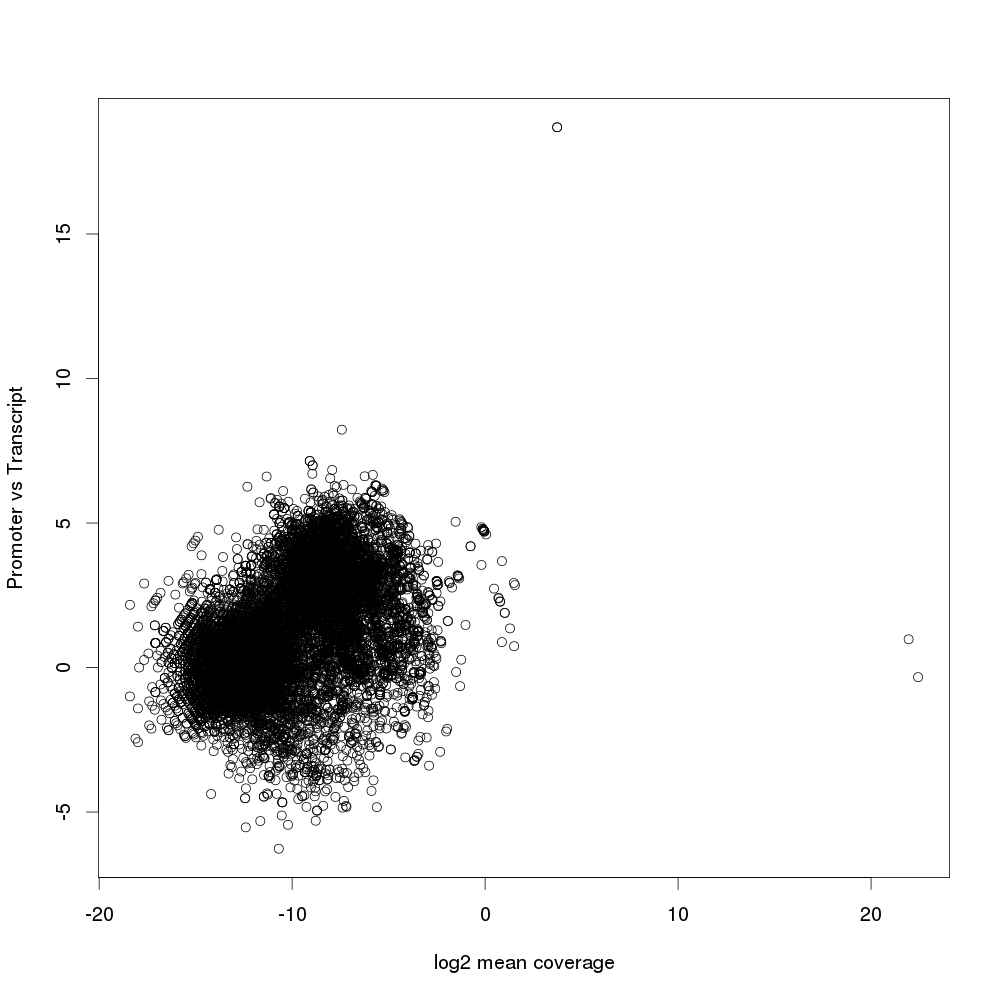

Supplement: S2 File — (ZIP) [file pone.0232332.s012.zip › nucleosome_positioning/SRR8932925_pt_score.png]

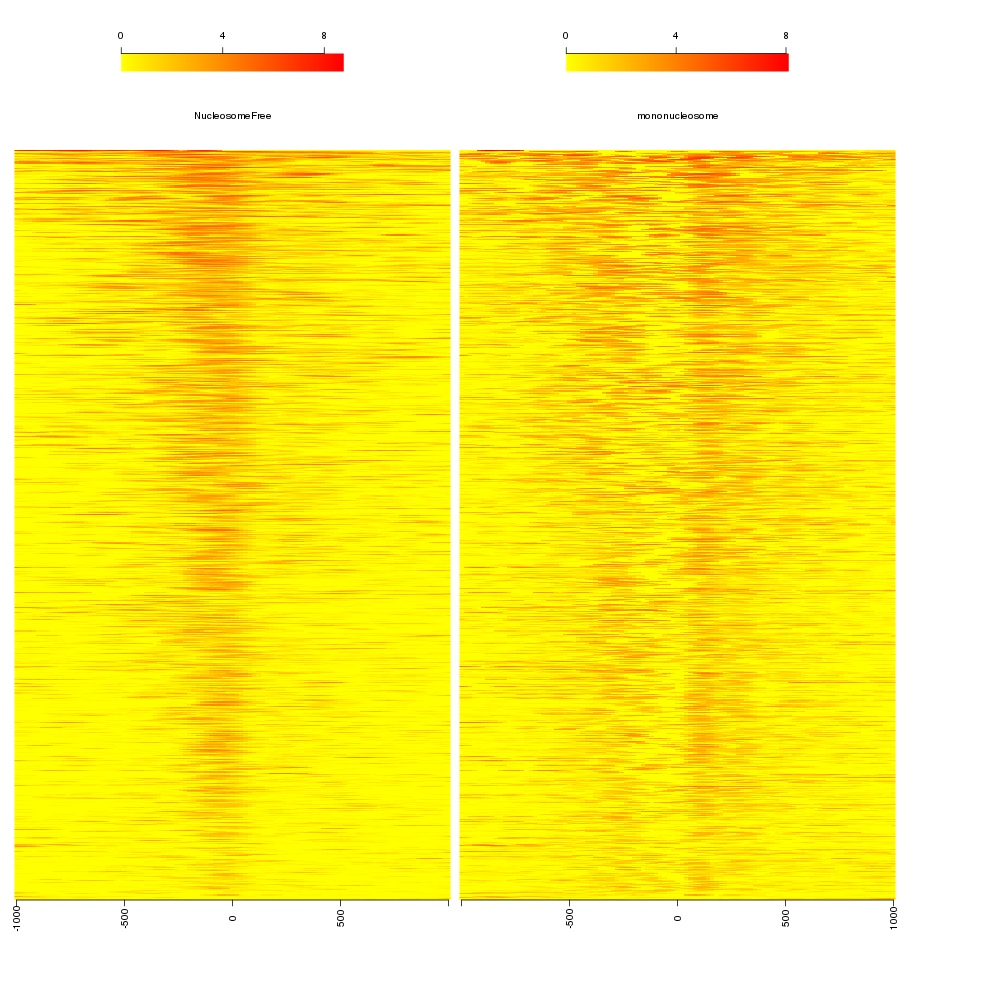

Supplement: S2 File — (ZIP) [file pone.0232332.s012.zip › nucleosome_positioning/SRX6443489_nucleosome_heatmap.png]

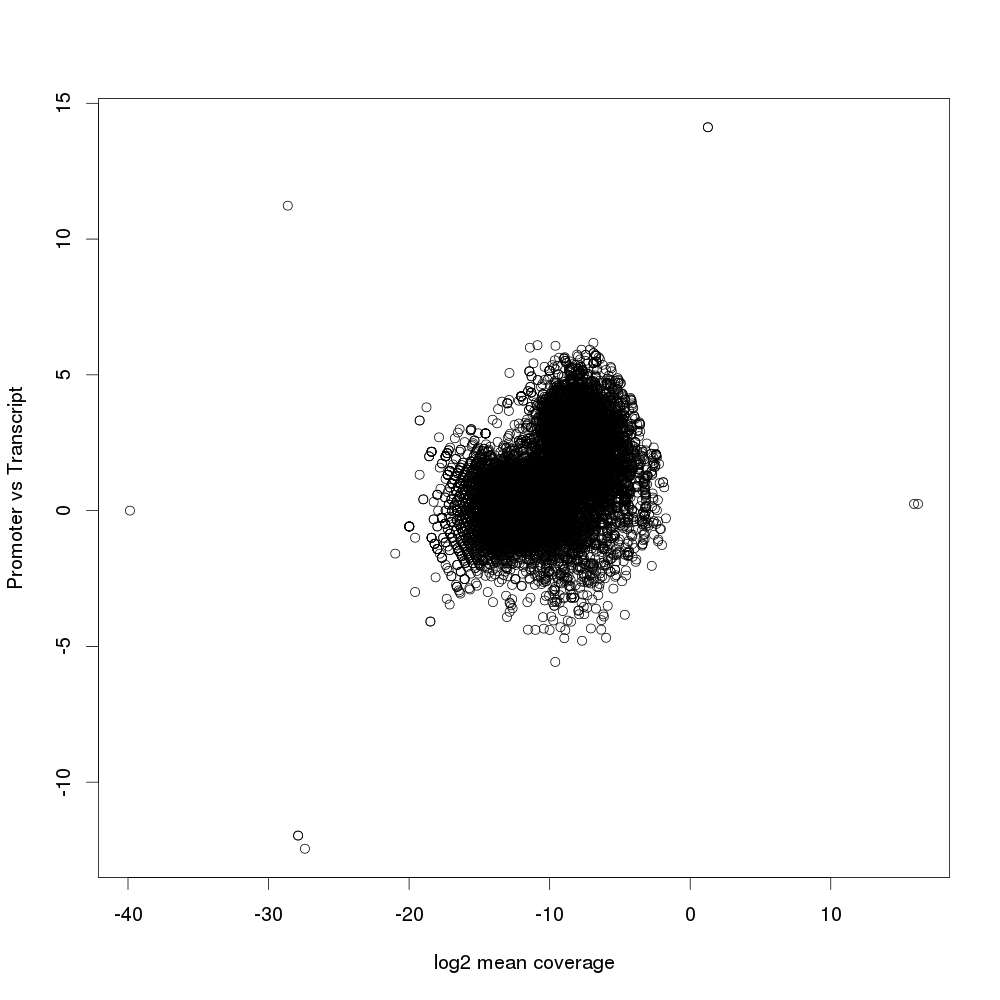

Supplement: S2 File — (ZIP) [file pone.0232332.s012.zip › nucleosome_positioning/SRR7140571_pt_score.png]

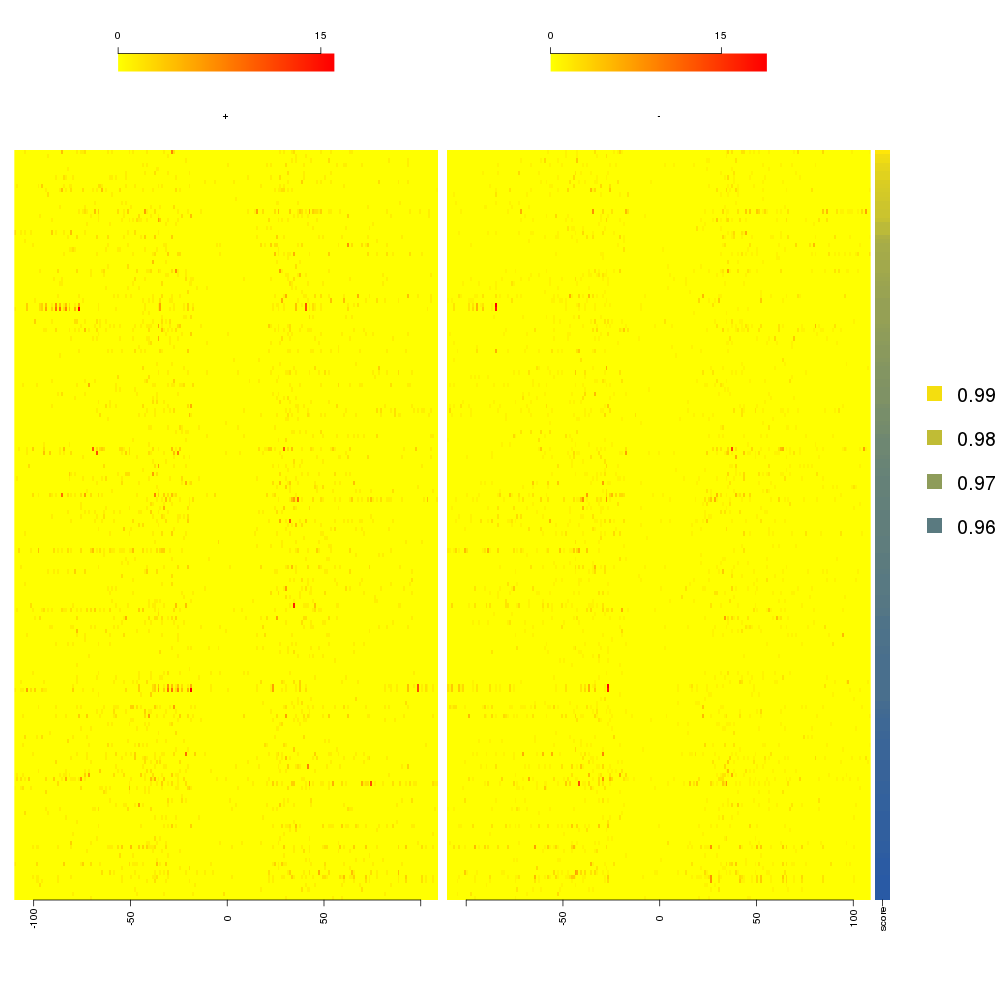

Supplement: S2 File — (ZIP) [file pone.0232332.s012.zip › nucleosome_positioning/SRR5876159_feature_aligned_heatmap.png]

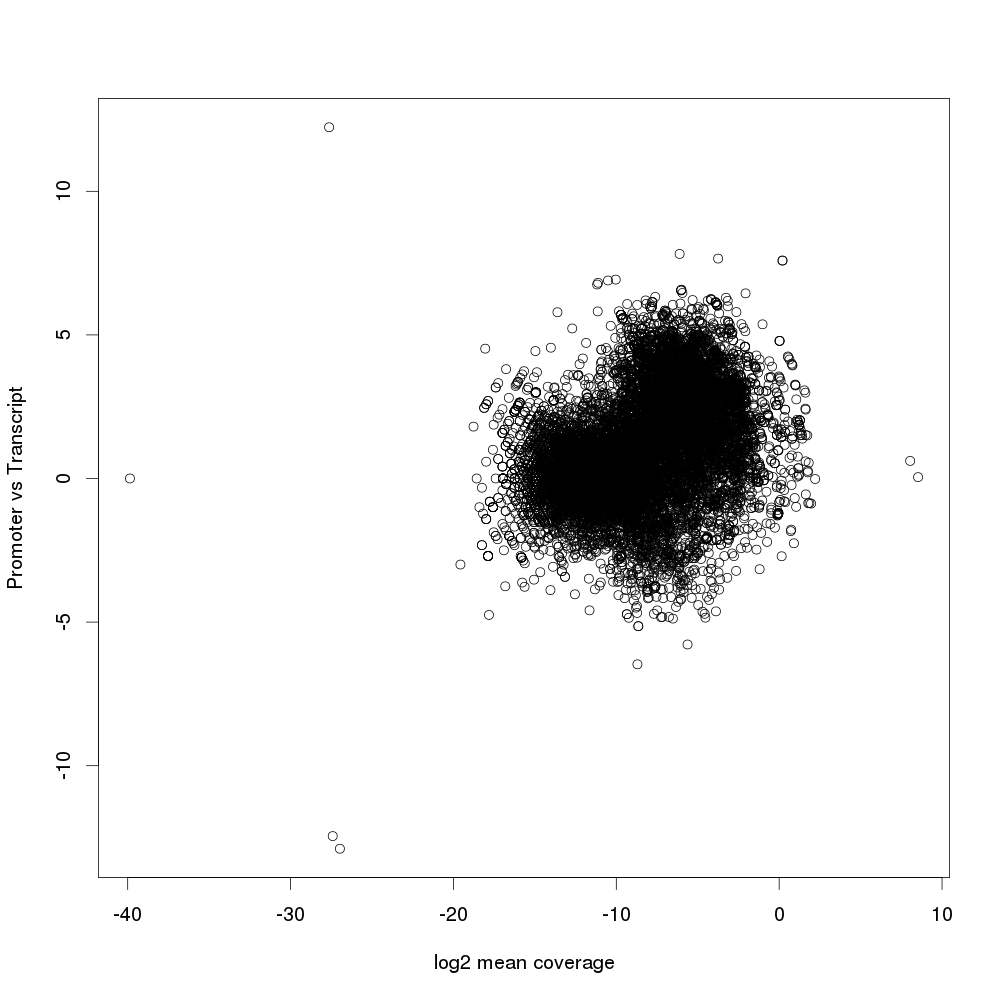

Supplement: S2 File — (ZIP) [file pone.0232332.s012.zip › nucleosome_positioning/SRX6443491_pt_score.png]

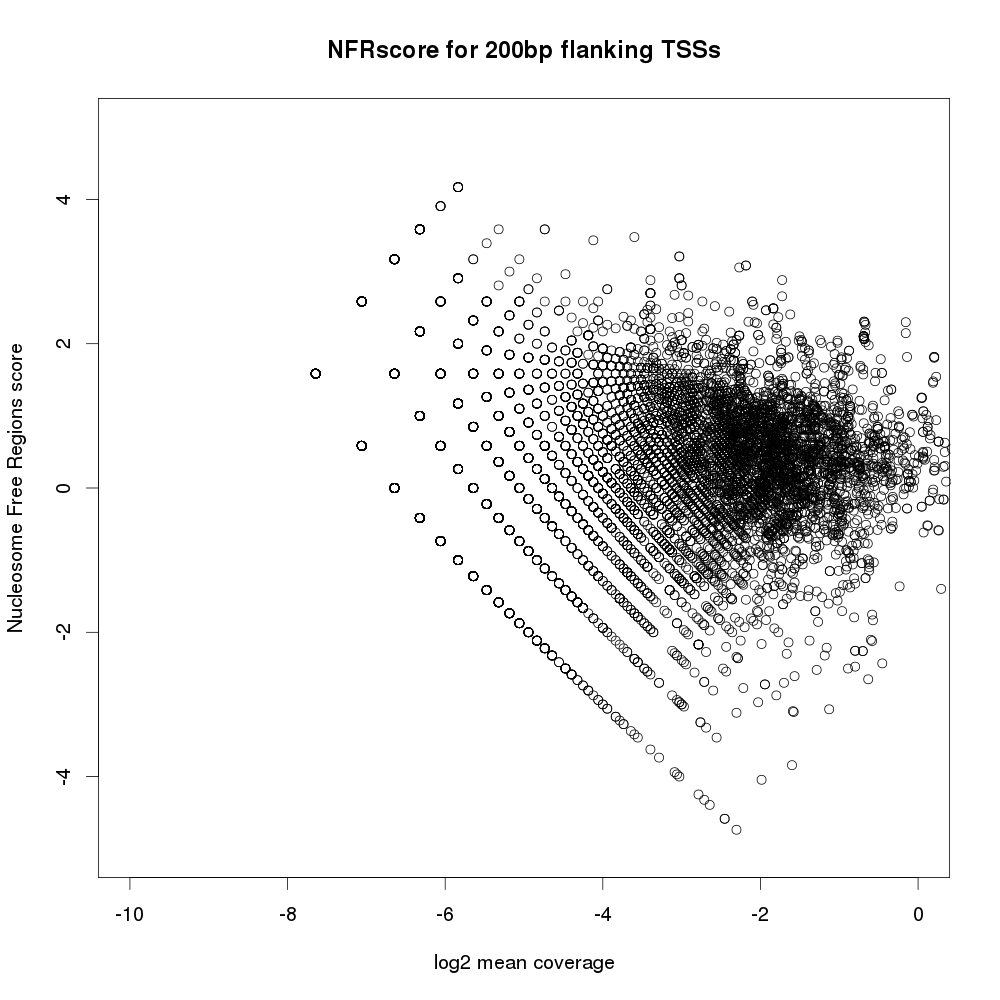

Supplement: S2 File — (ZIP) [file pone.0232332.s012.zip › nucleosome_positioning/SRR1822168_NFRscore.png]

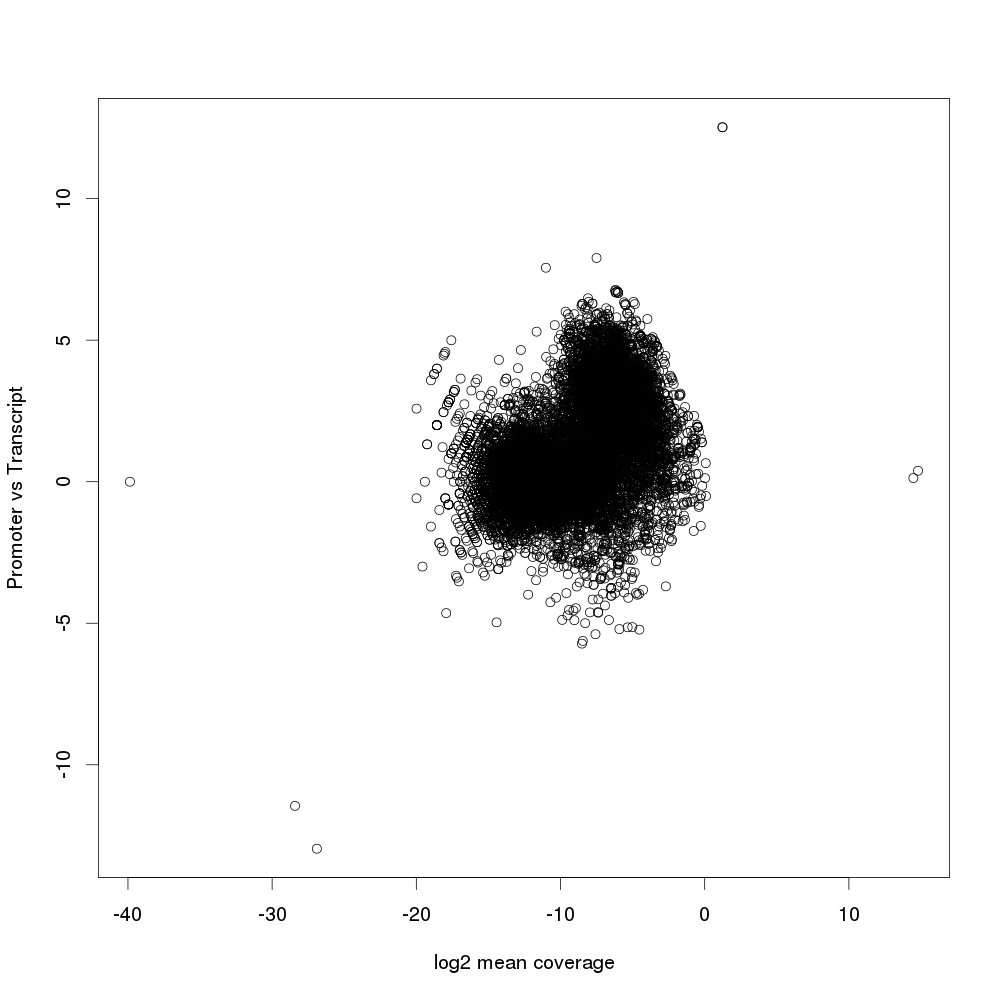

Supplement: S2 File — (ZIP) [file pone.0232332.s012.zip › nucleosome_positioning/SRR7140573_pt_score.png]

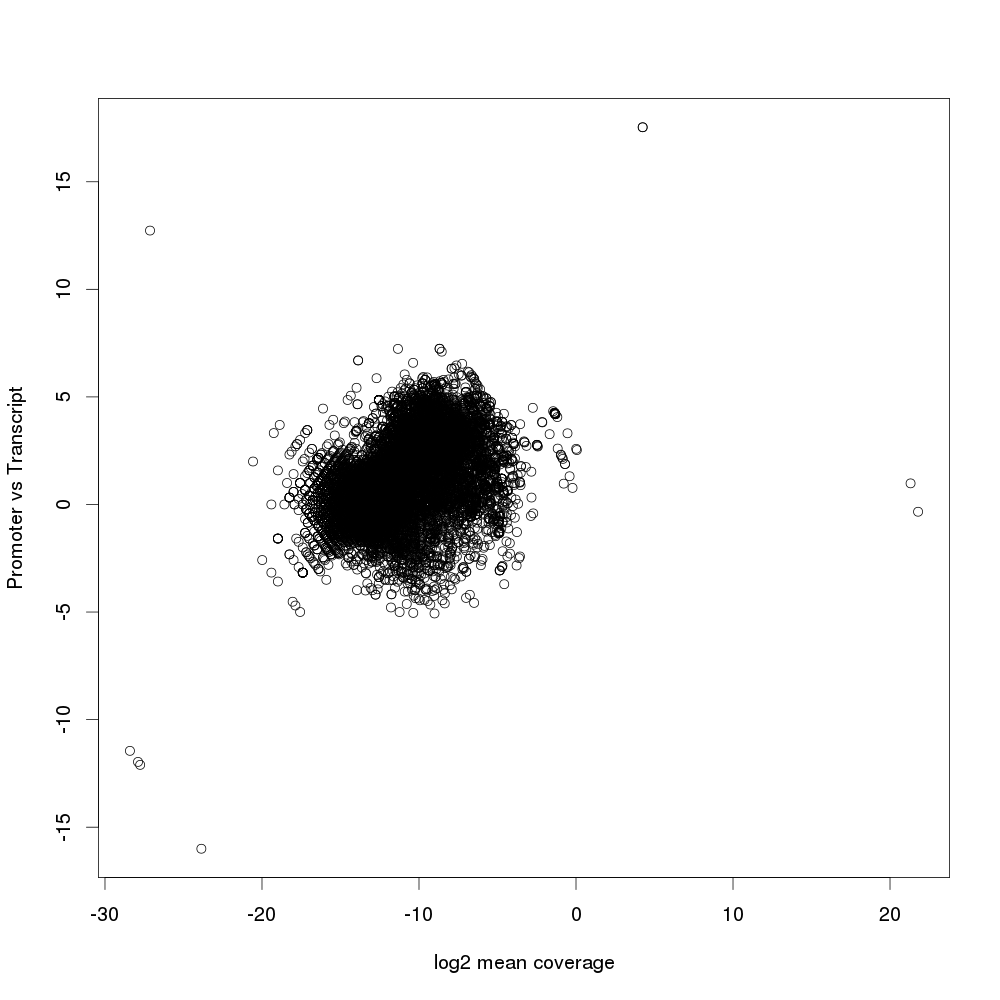

Supplement: S2 File — (ZIP) [file pone.0232332.s012.zip › nucleosome_positioning/SRR8932927_pt_score.png]

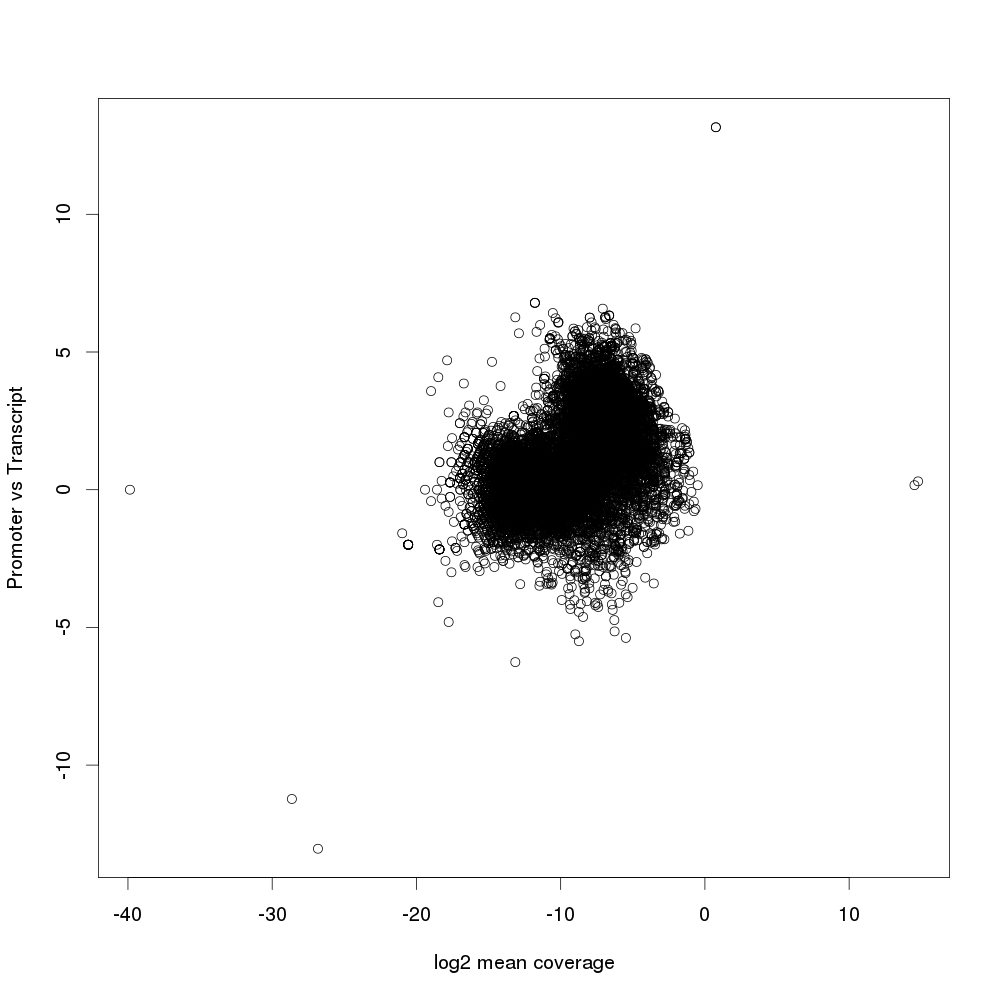

Supplement: S2 File — (ZIP) [file pone.0232332.s012.zip › nucleosome_positioning/SRR7140572_pt_score.png]

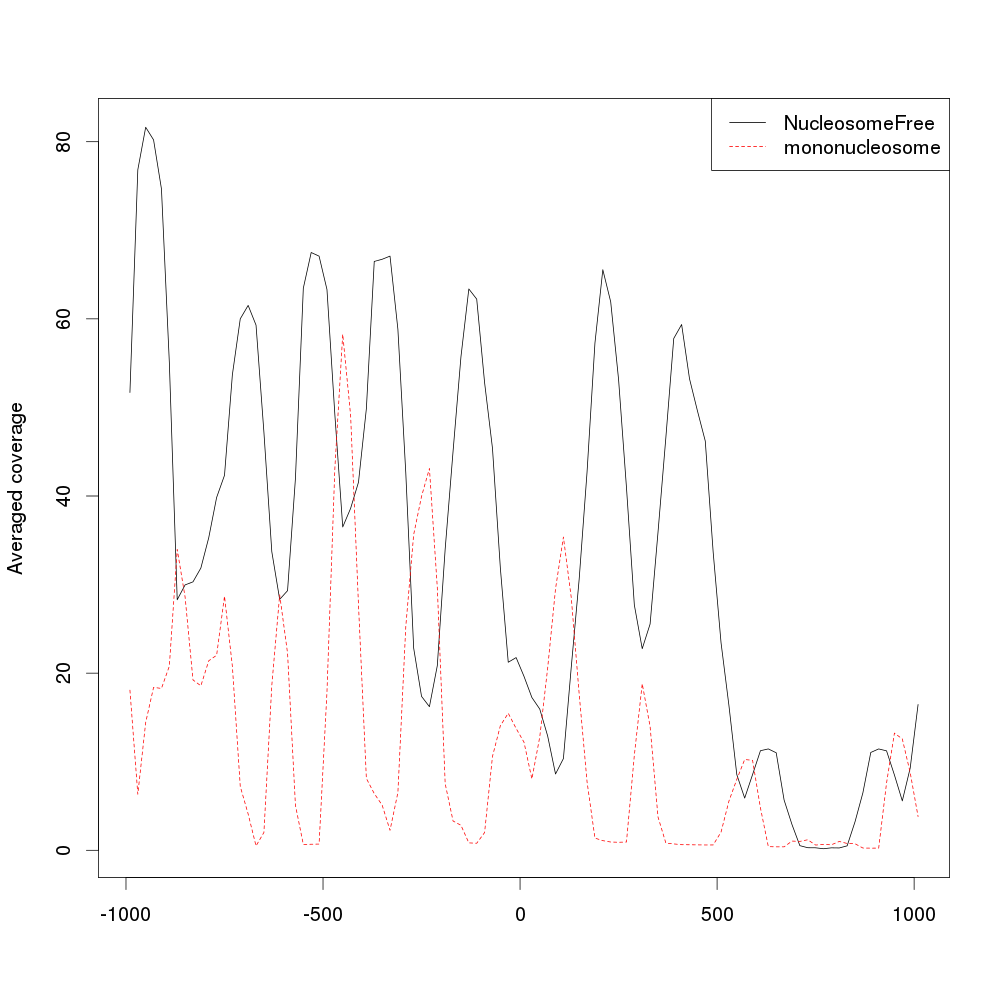

Supplement: S2 File — (ZIP) [file pone.0232332.s012.zip › nucleosome_positioning/SRR1822167_nucleosome_distribution.png]

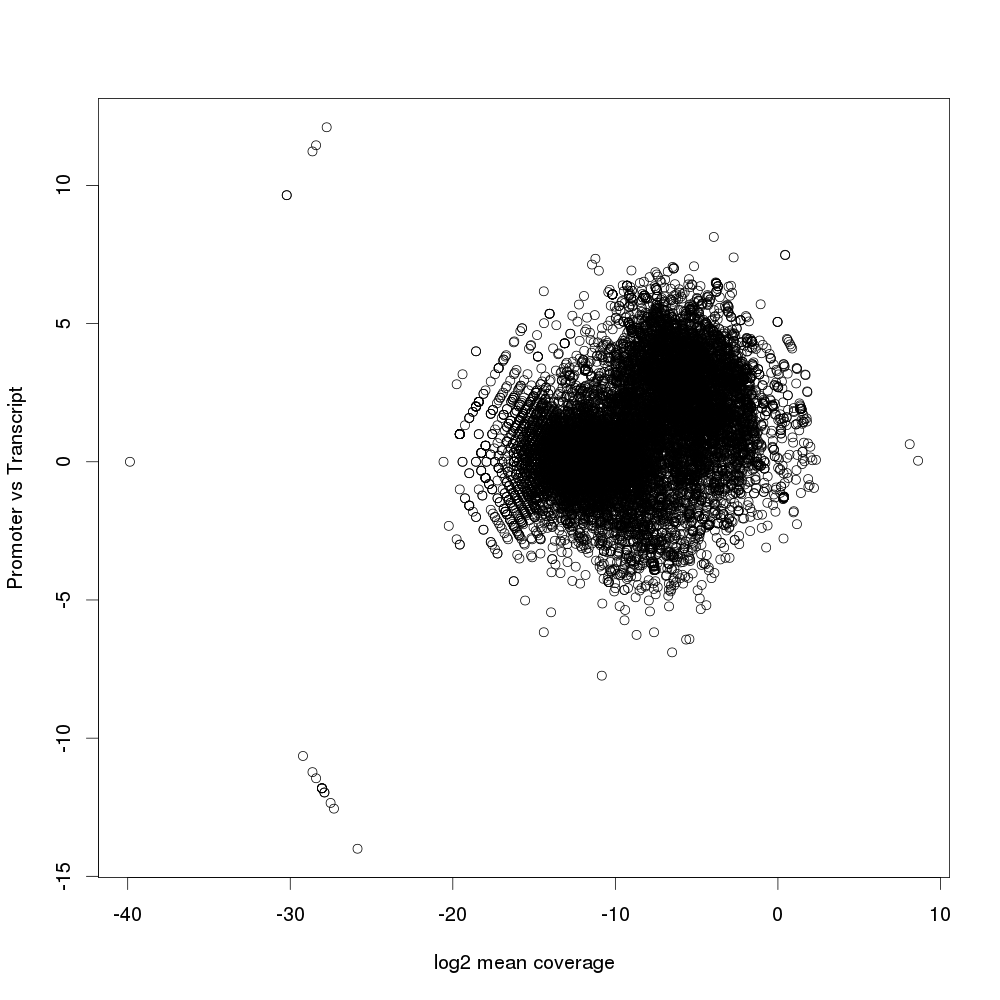

Supplement: S2 File — (ZIP) [file pone.0232332.s012.zip › nucleosome_positioning/SRX6443490_pt_score.png]

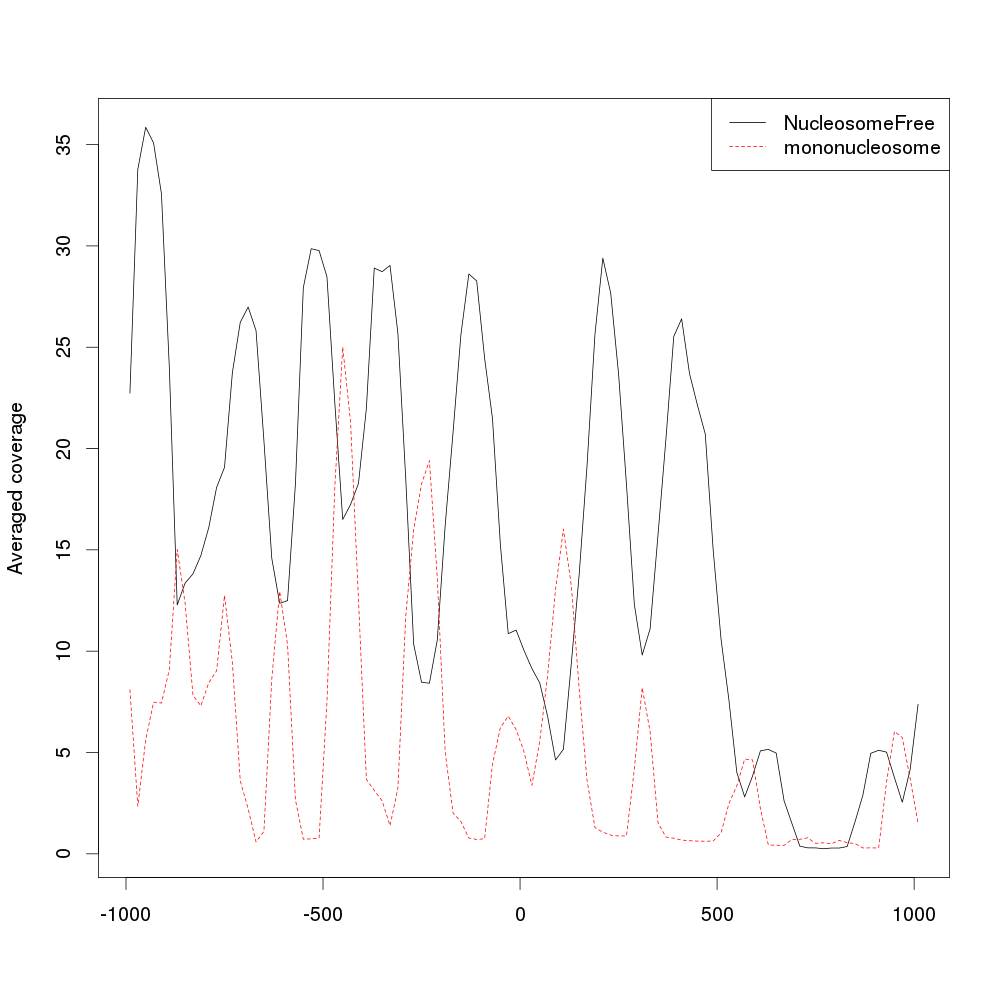

Supplement: S2 File — (ZIP) [file pone.0232332.s012.zip › nucleosome_positioning/SRR1822165_nucleosome_distribution.png]

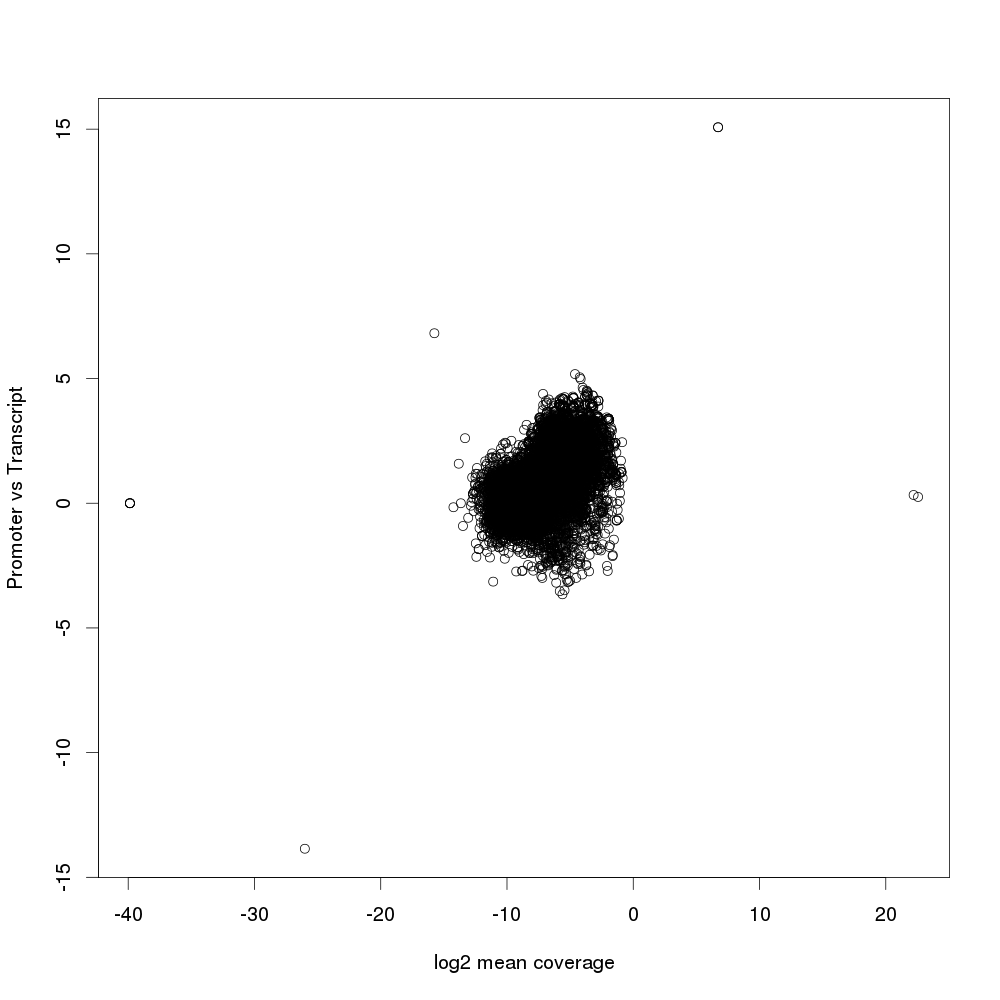

Supplement: S2 File — (ZIP) [file pone.0232332.s012.zip › nucleosome_positioning/SRR1822165_pt_score.png]

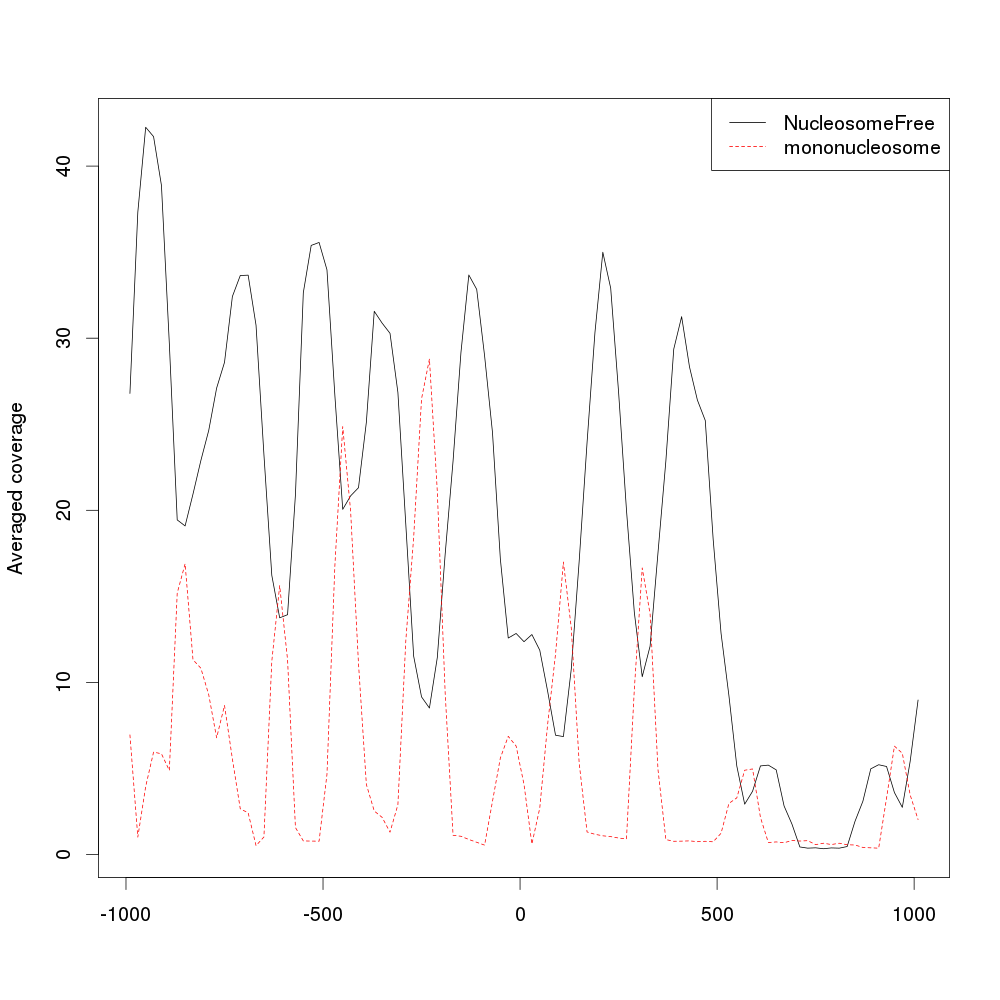

Supplement: S2 File — (ZIP) [file pone.0232332.s012.zip › nucleosome_positioning/SRR3622818_nucleosome_distribution.png]

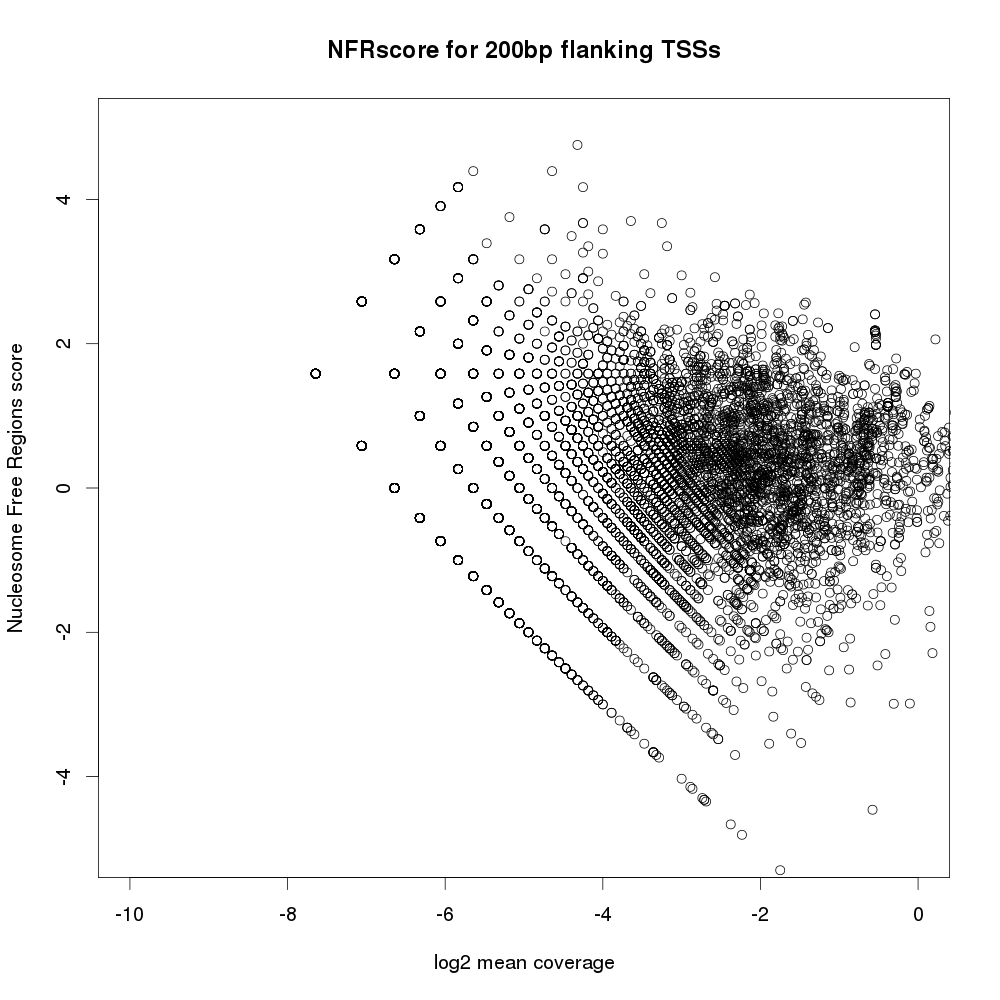

Supplement: S2 File — (ZIP) [file pone.0232332.s012.zip › nucleosome_positioning/SRR5876158_NFRscore.png]

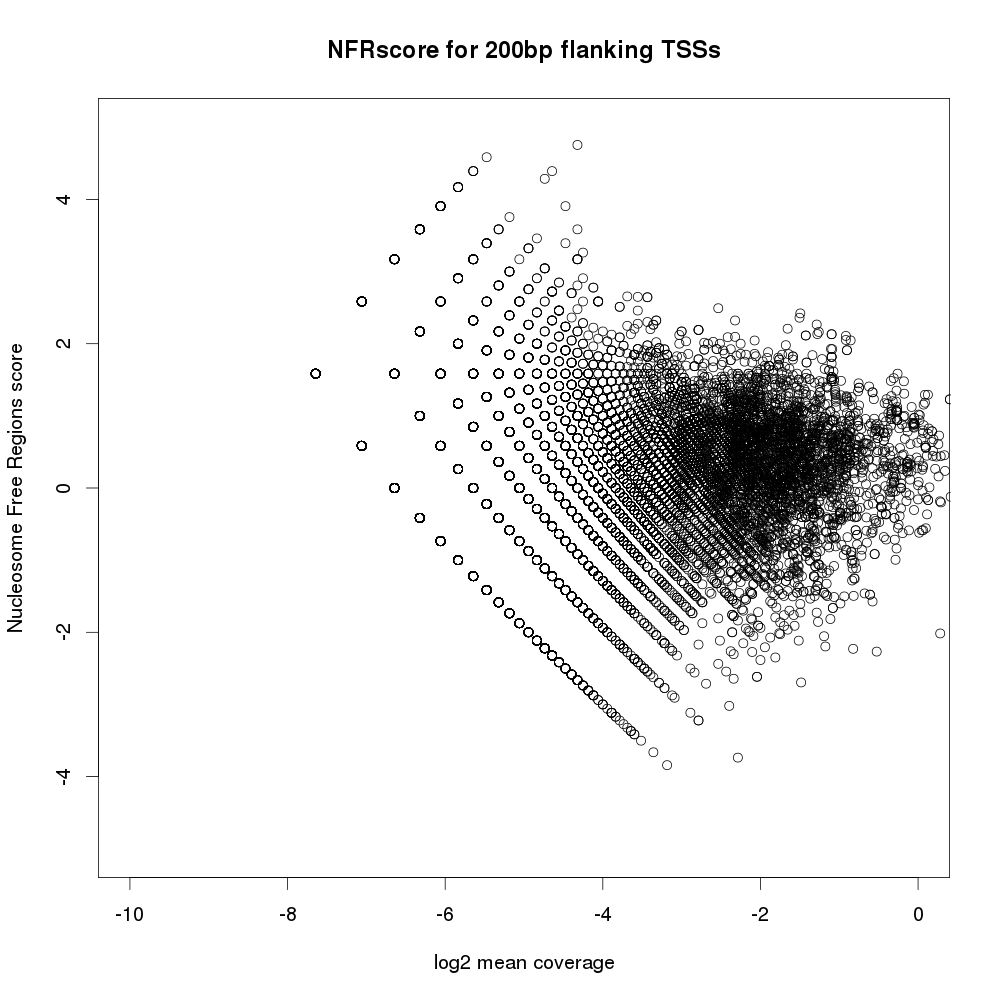

Supplement: S2 File — (ZIP) [file pone.0232332.s012.zip › nucleosome_positioning/SRR3622819_NFRscore.png]

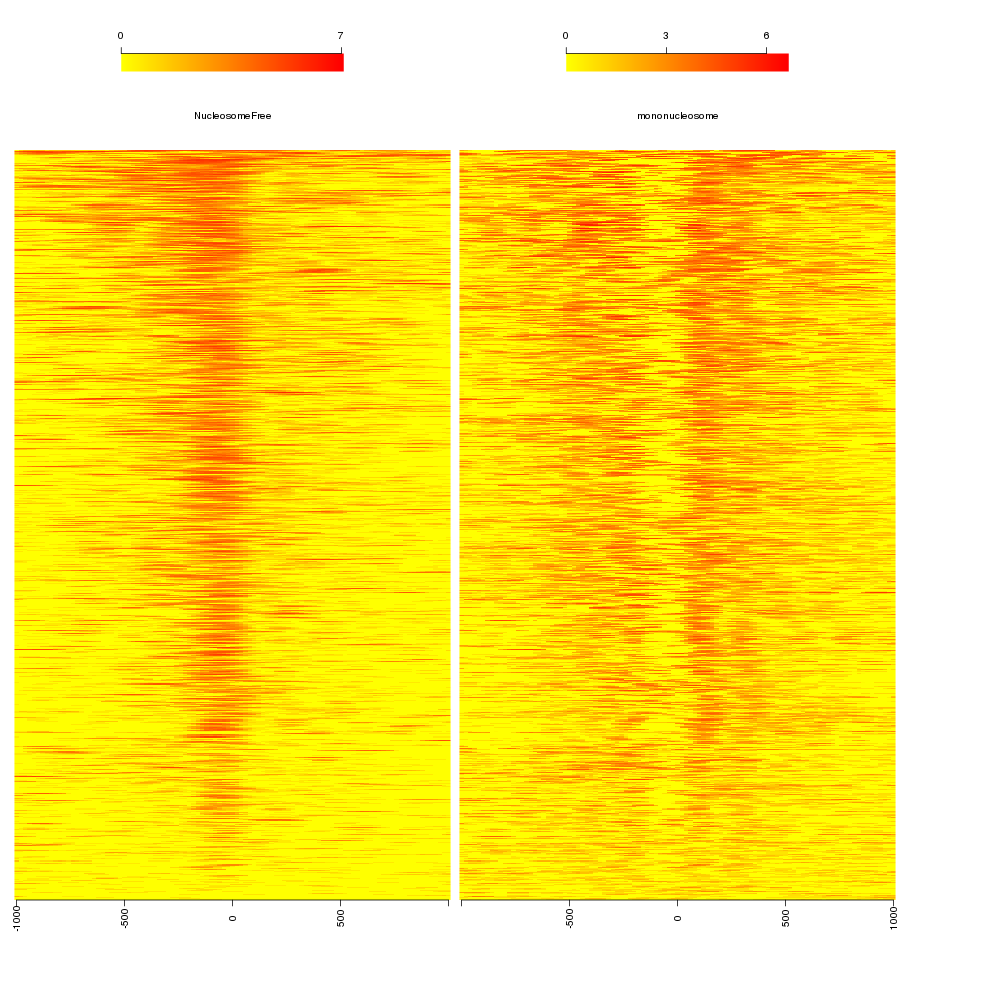

Supplement: S2 File — (ZIP) [file pone.0232332.s012.zip › nucleosome_positioning/SRR6216227_nucleosome_heatmap.png]

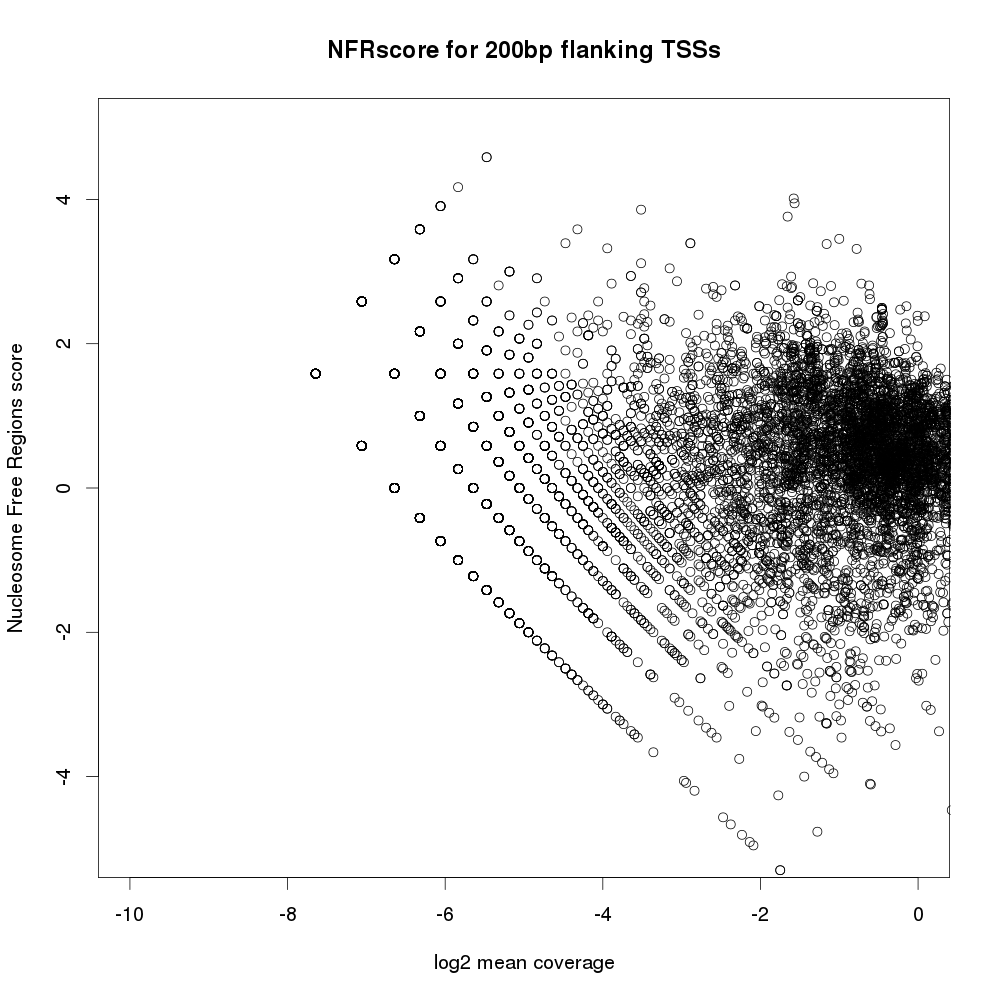

Supplement: S2 File — (ZIP) [file pone.0232332.s012.zip › nucleosome_positioning/SRR6216226_NFRscore.png]

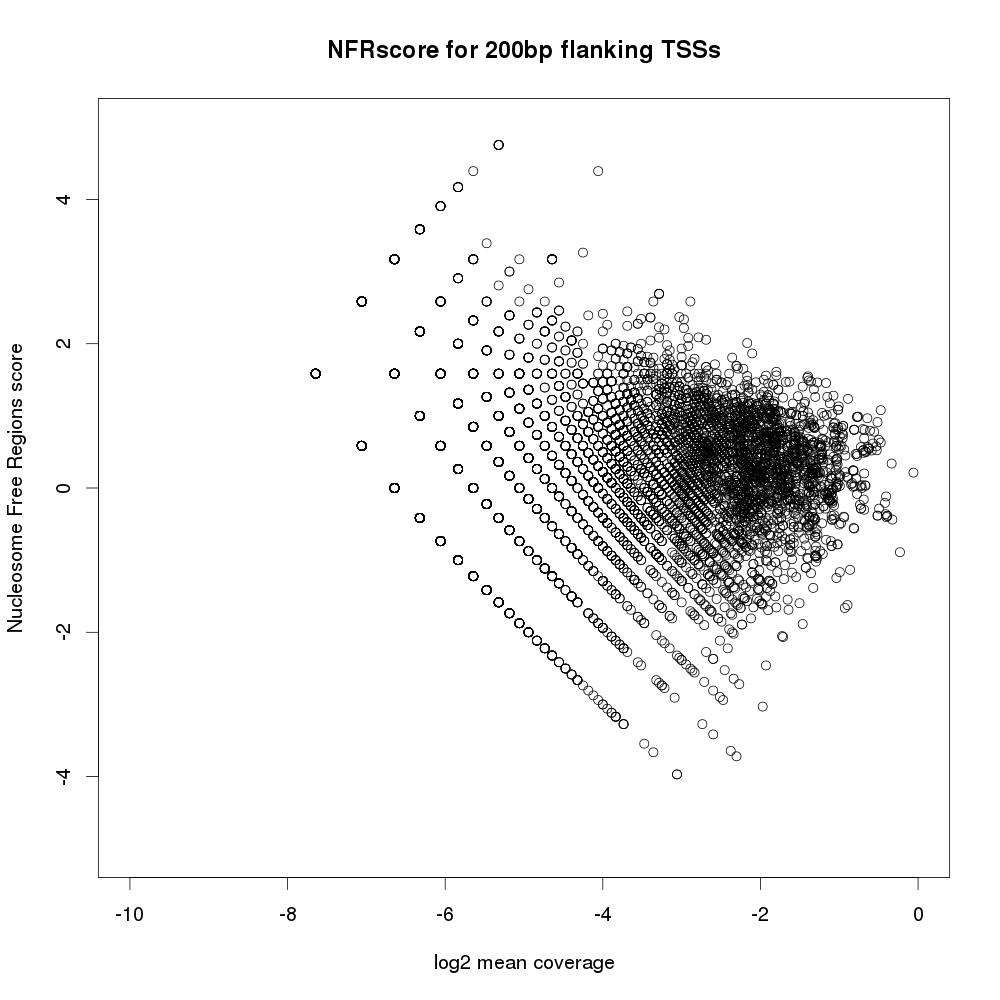

Supplement: S2 File — (ZIP) [file pone.0232332.s012.zip › nucleosome_positioning/SRR5128074_NFRscore.png]

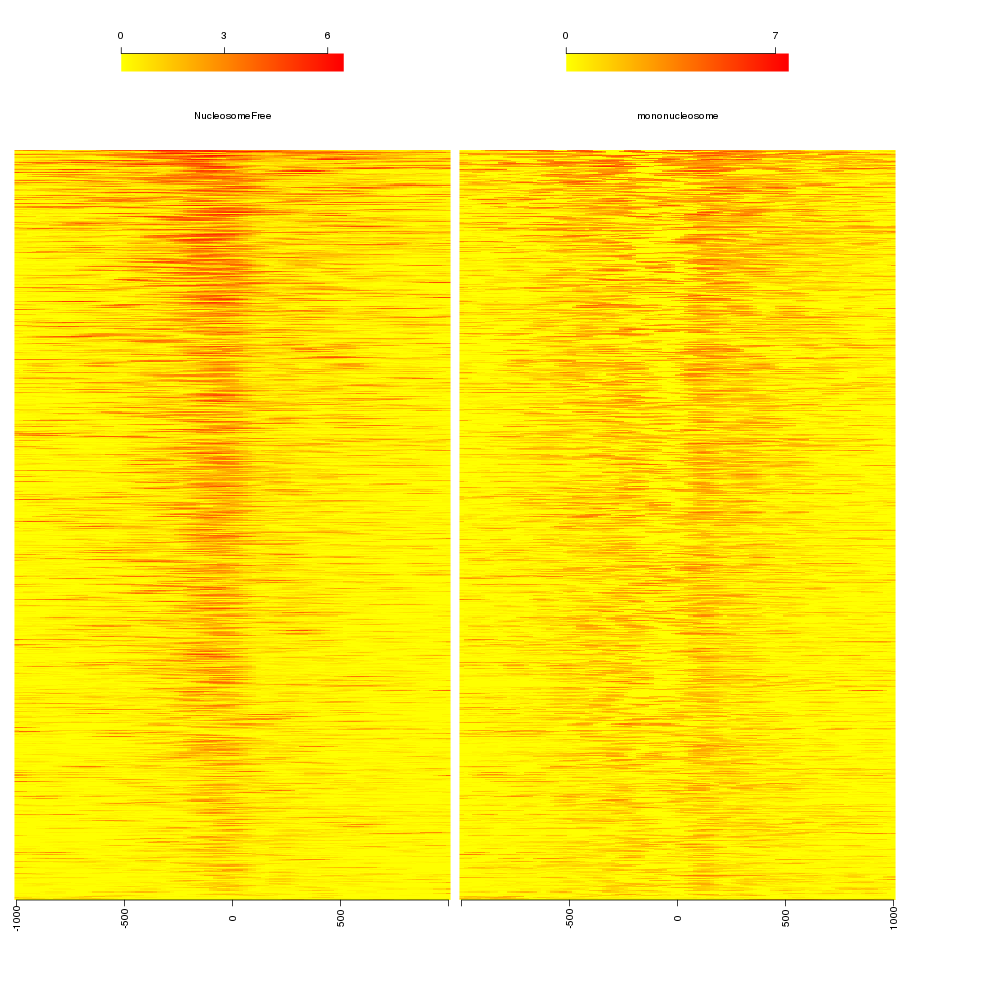

Supplement: S2 File — (ZIP) [file pone.0232332.s012.zip › nucleosome_positioning/SRX6443491_nucleosome_heatmap.png]

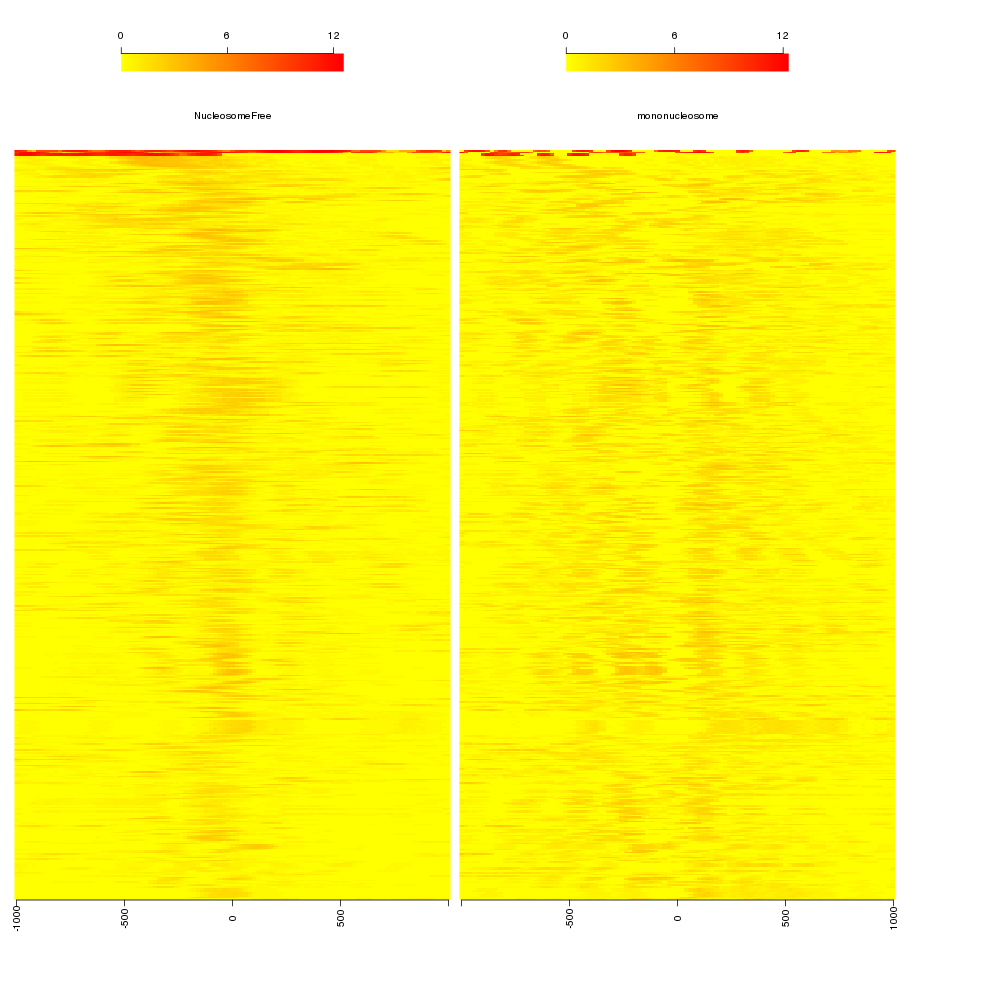

Supplement: S2 File — (ZIP) [file pone.0232332.s012.zip › nucleosome_positioning/SRR1822166_nucleosome_heatmap.png]

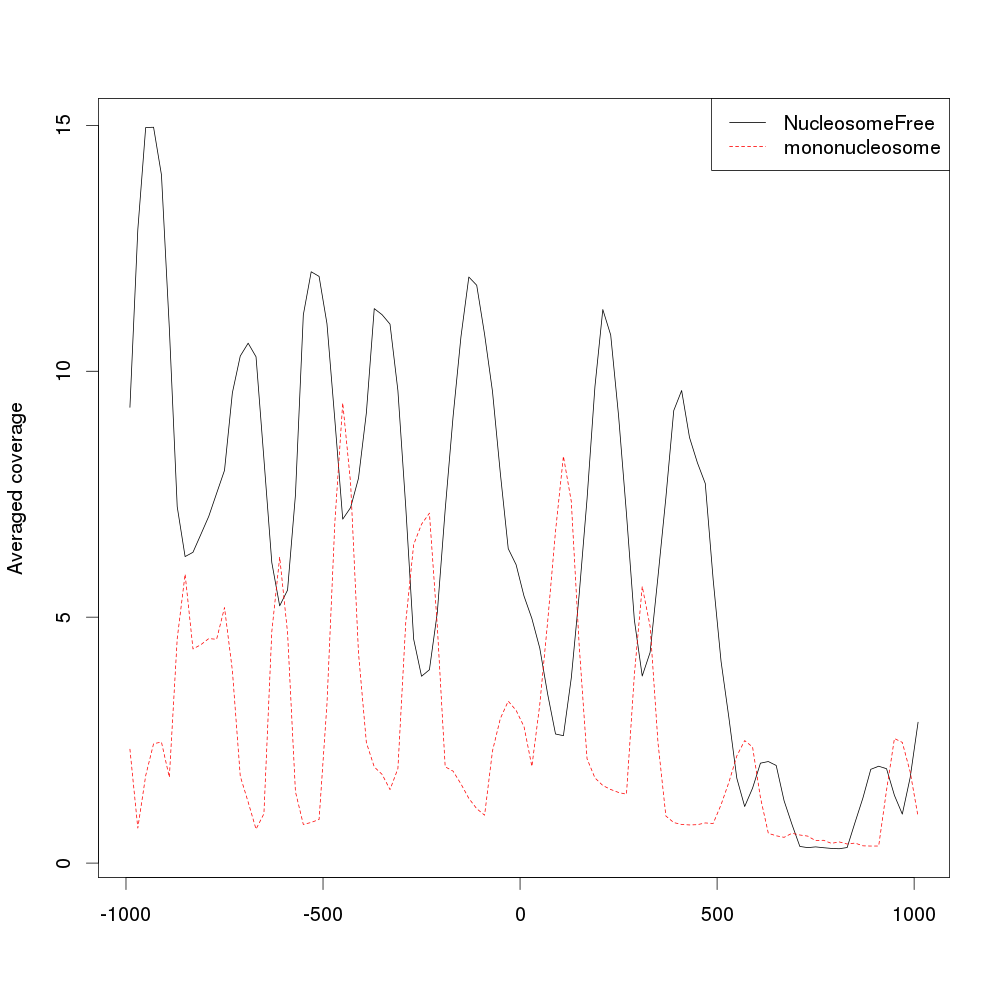

Supplement: S2 File — (ZIP) [file pone.0232332.s012.zip › nucleosome_positioning/SRR891275_nucleosome_distribution.png]

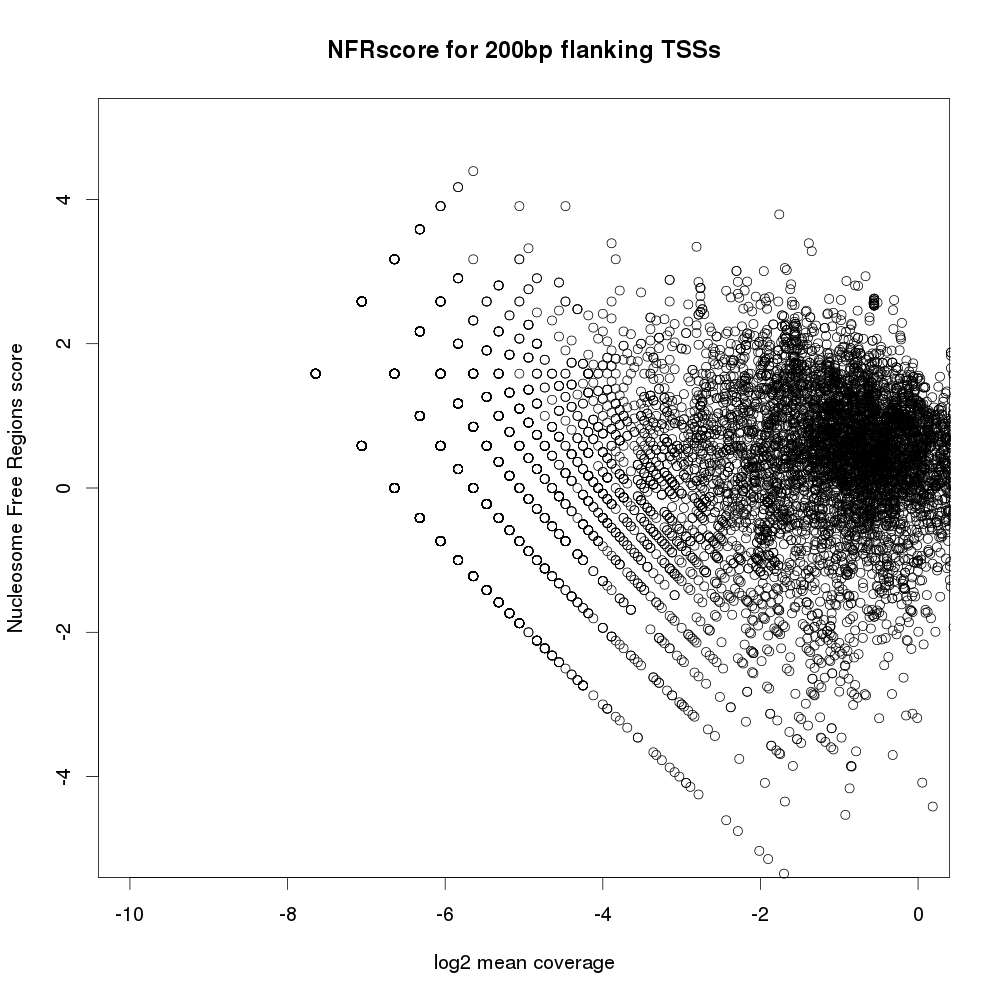

Supplement: S2 File — (ZIP) [file pone.0232332.s012.zip › nucleosome_positioning/SRR6216227_NFRscore.png]

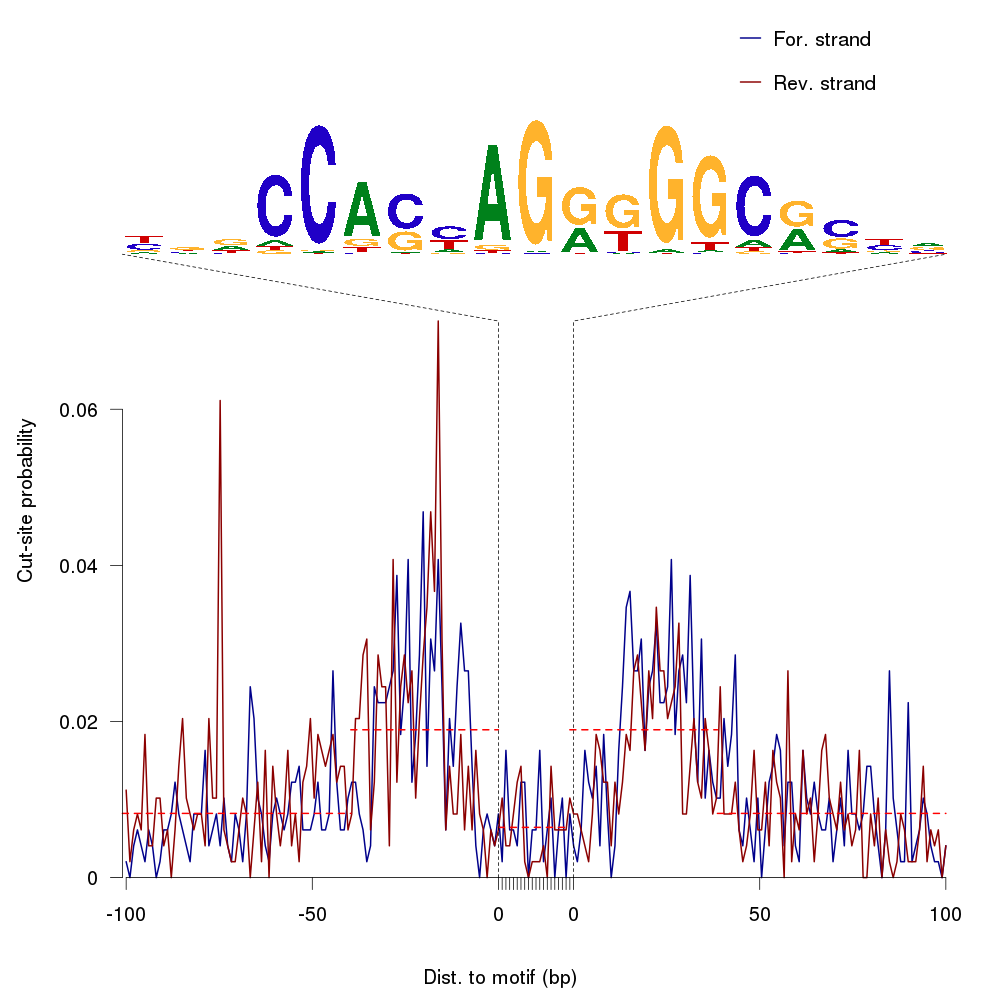

Supplement: S2 File — (ZIP) [file pone.0232332.s012.zip › nucleosome_positioning/SRR5128074_footprint_plot.png]

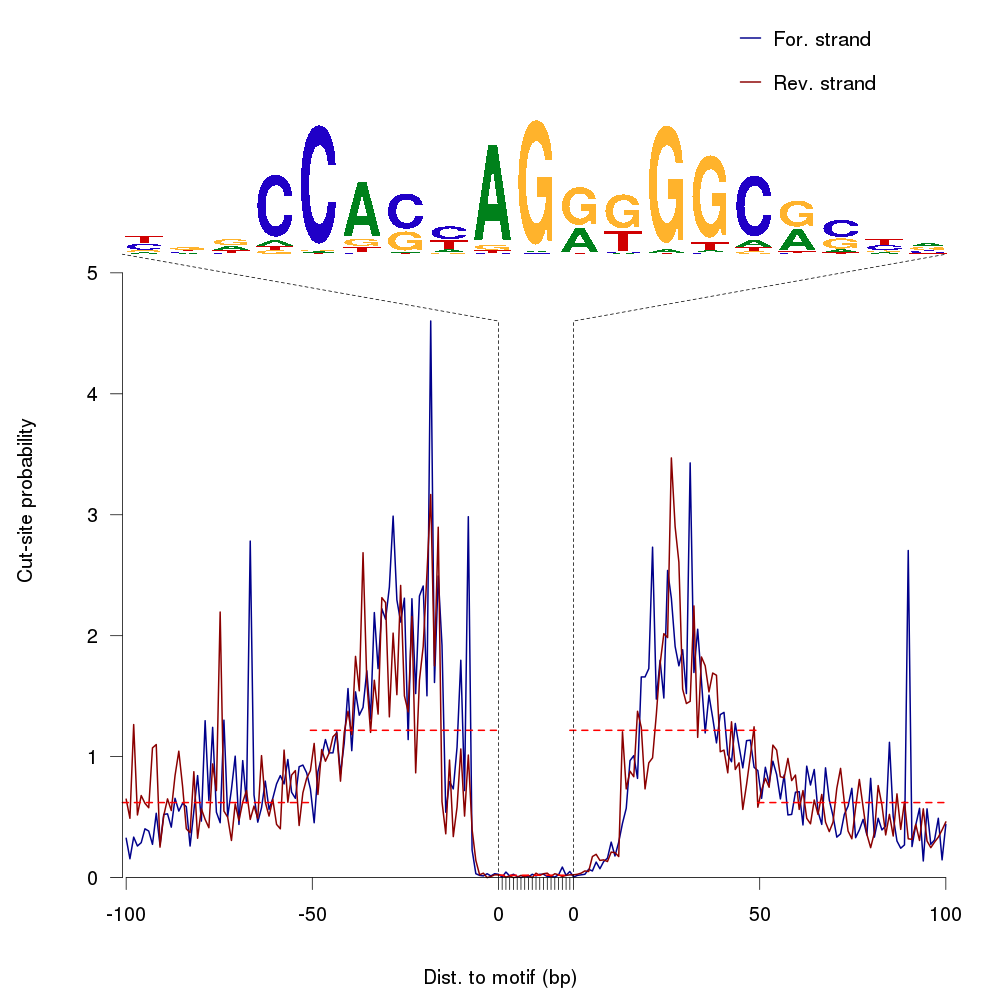

Supplement: S2 File — (ZIP) [file pone.0232332.s012.zip › nucleosome_positioning/SRX6443488_footprint_plot.png]

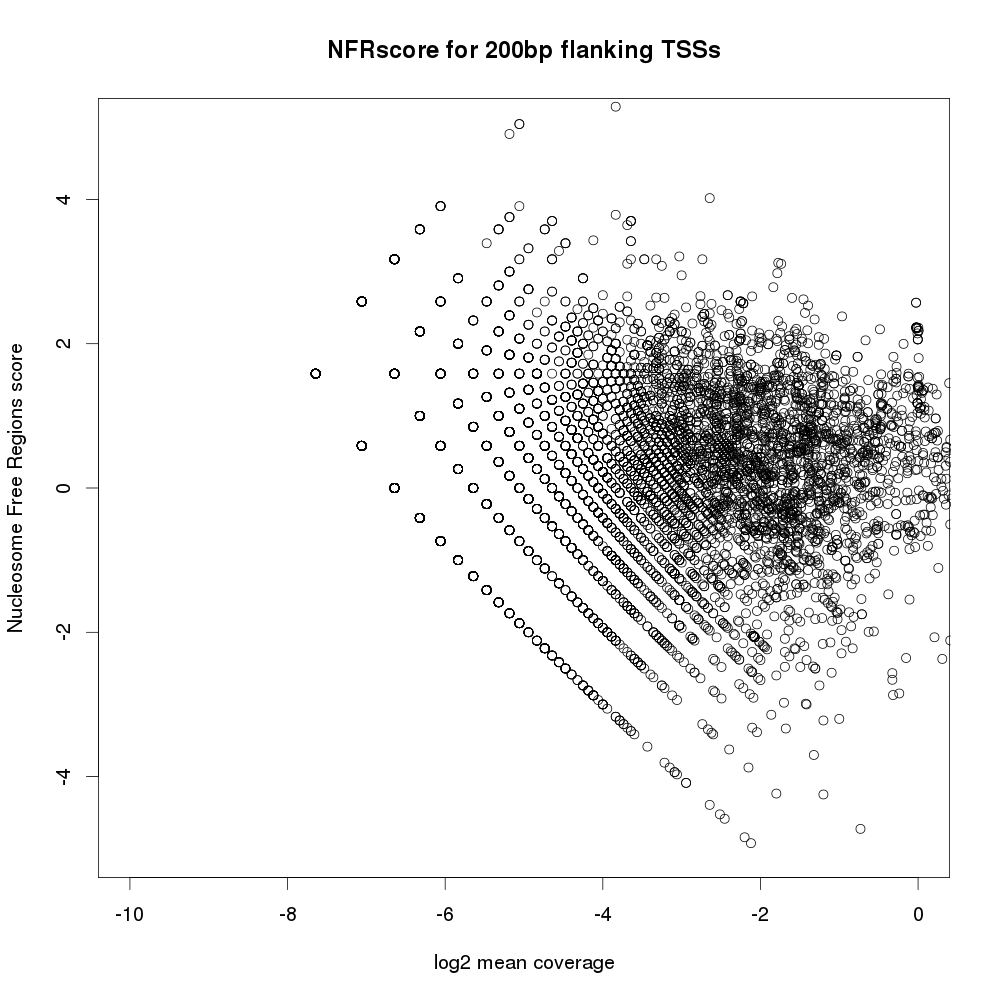

Supplement: S2 File — (ZIP) [file pone.0232332.s012.zip › nucleosome_positioning/SRR5876159_NFRscore.png]

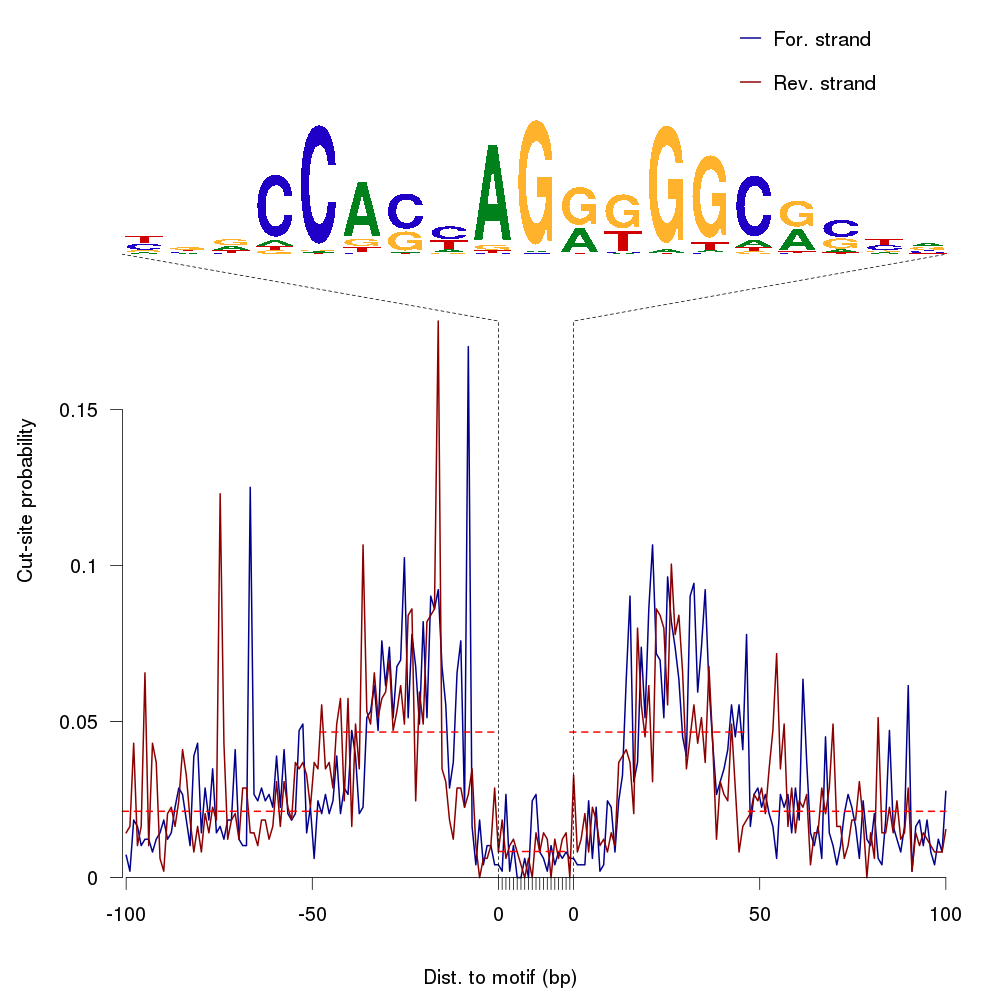

Supplement: S2 File — (ZIP) [file pone.0232332.s012.zip › nucleosome_positioning/SRR1822166_footprint_plot.png]

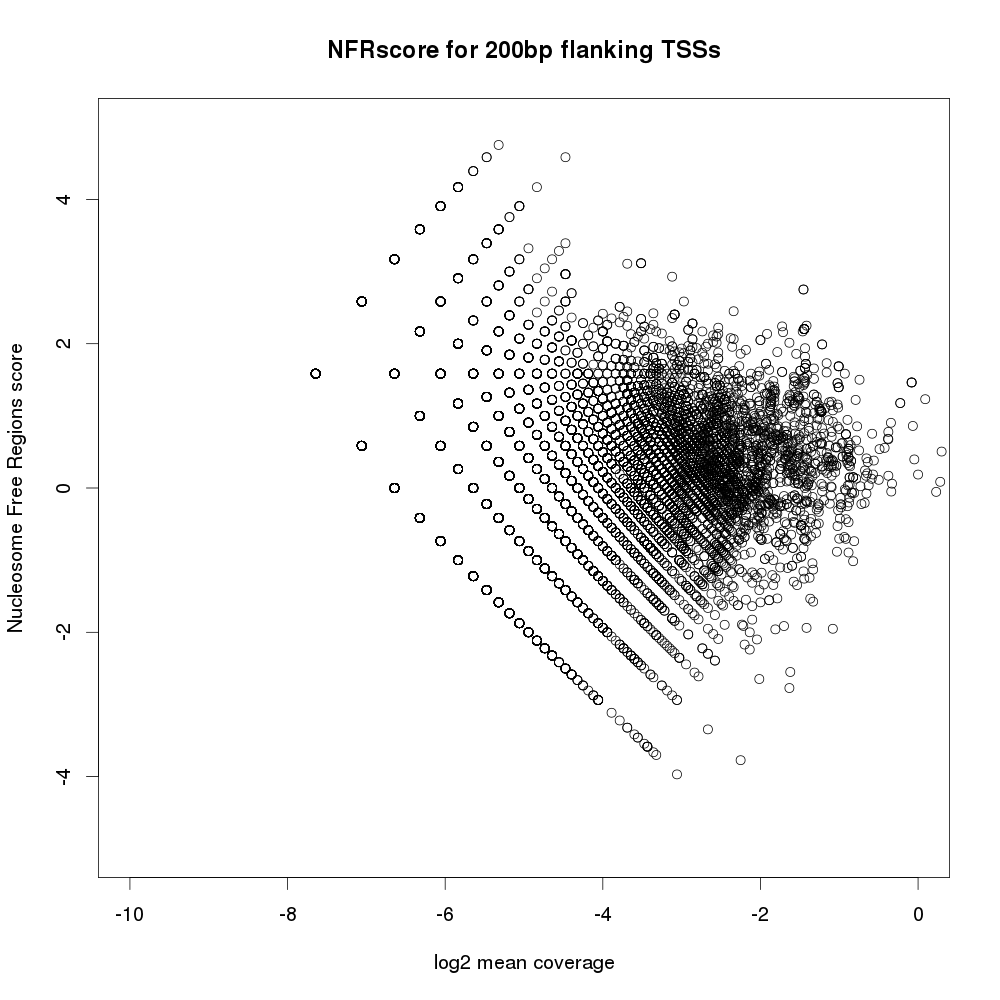

Supplement: S2 File — (ZIP) [file pone.0232332.s012.zip › nucleosome_positioning/SRR3622818_NFRscore.png]

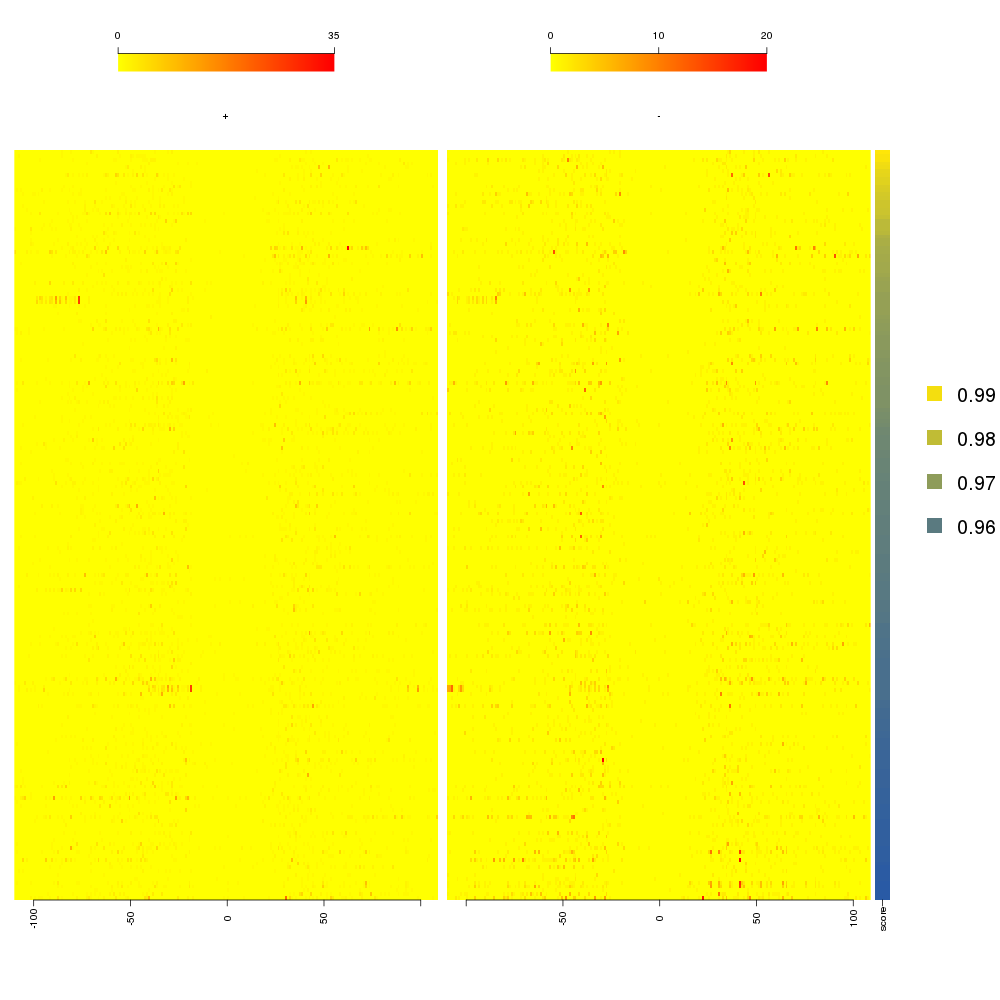

Supplement: S2 File — (ZIP) [file pone.0232332.s012.zip › nucleosome_positioning/SRR6216227_feature_aligned_heatmap.png]

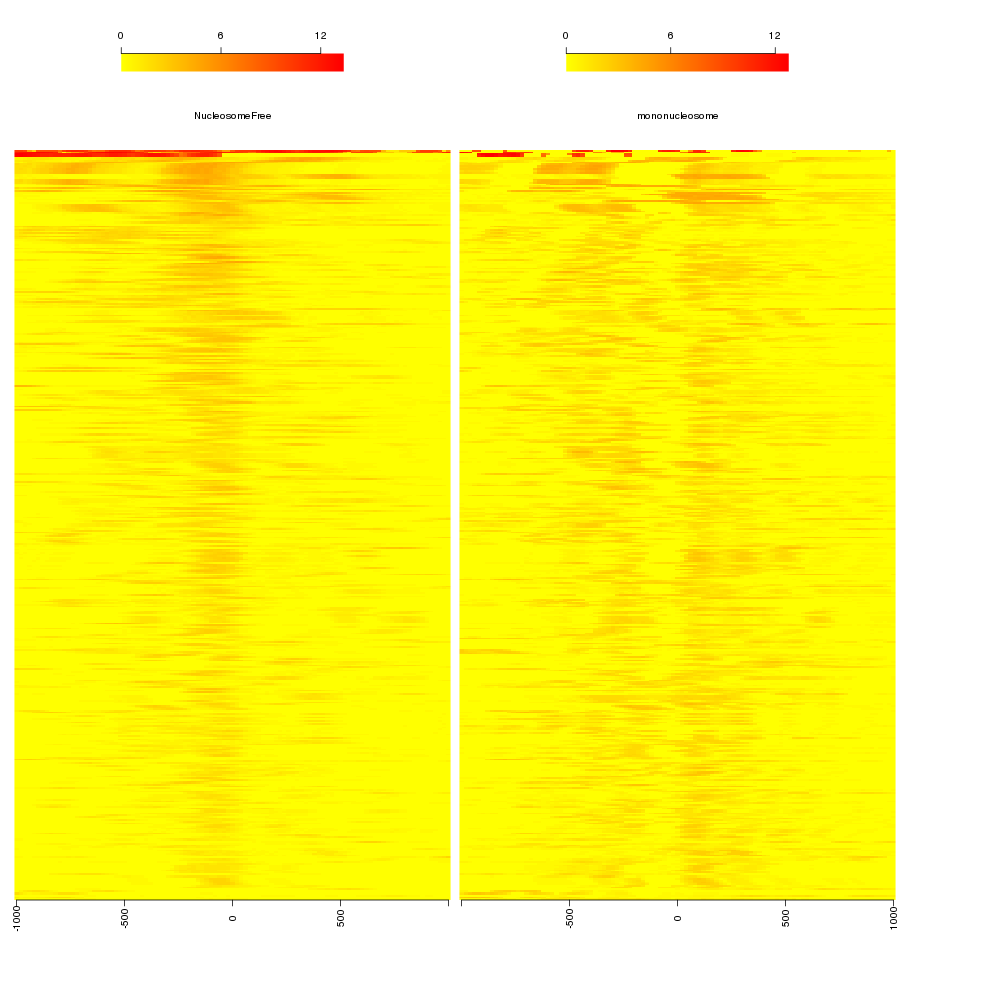

Supplement: S2 File — (ZIP) [file pone.0232332.s012.zip › nucleosome_positioning/SRR8932925_nucleosome_heatmap.png]

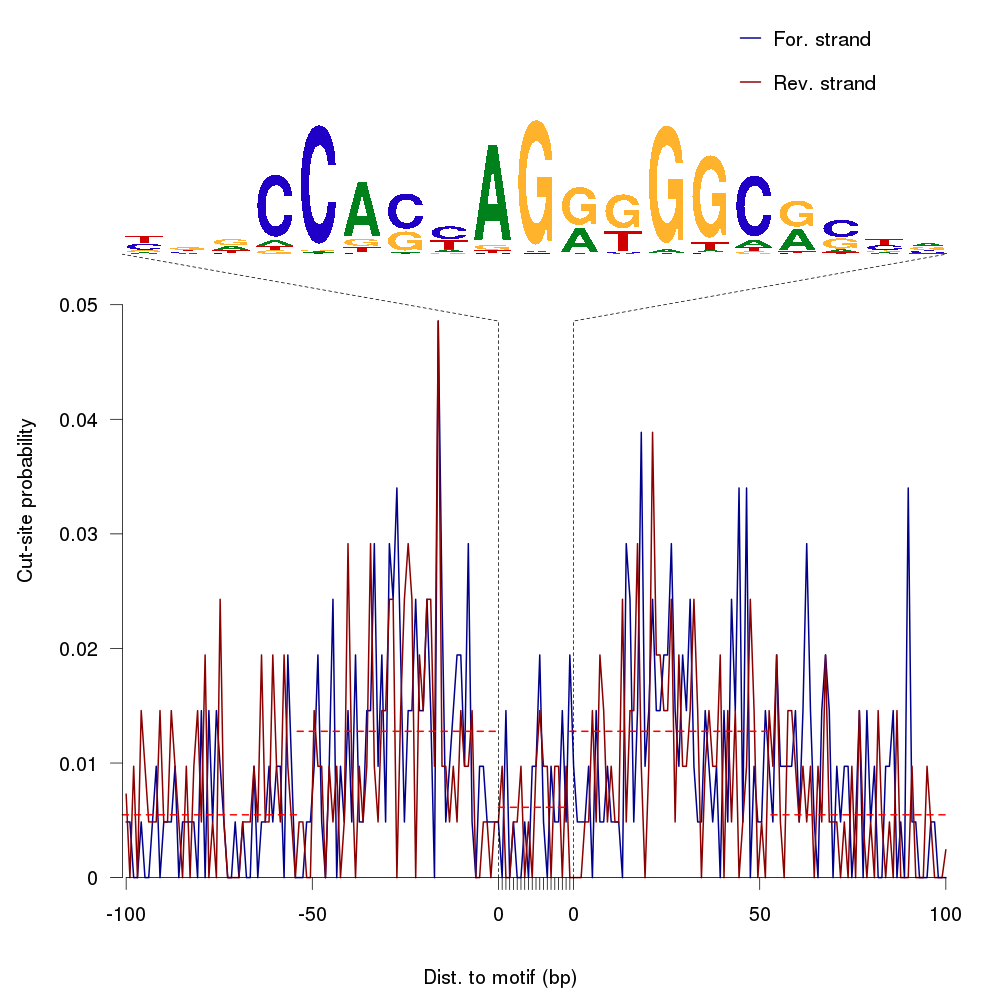

Supplement: S2 File — (ZIP) [file pone.0232332.s012.zip › nucleosome_positioning/SRR891276_footprint_plot.png]

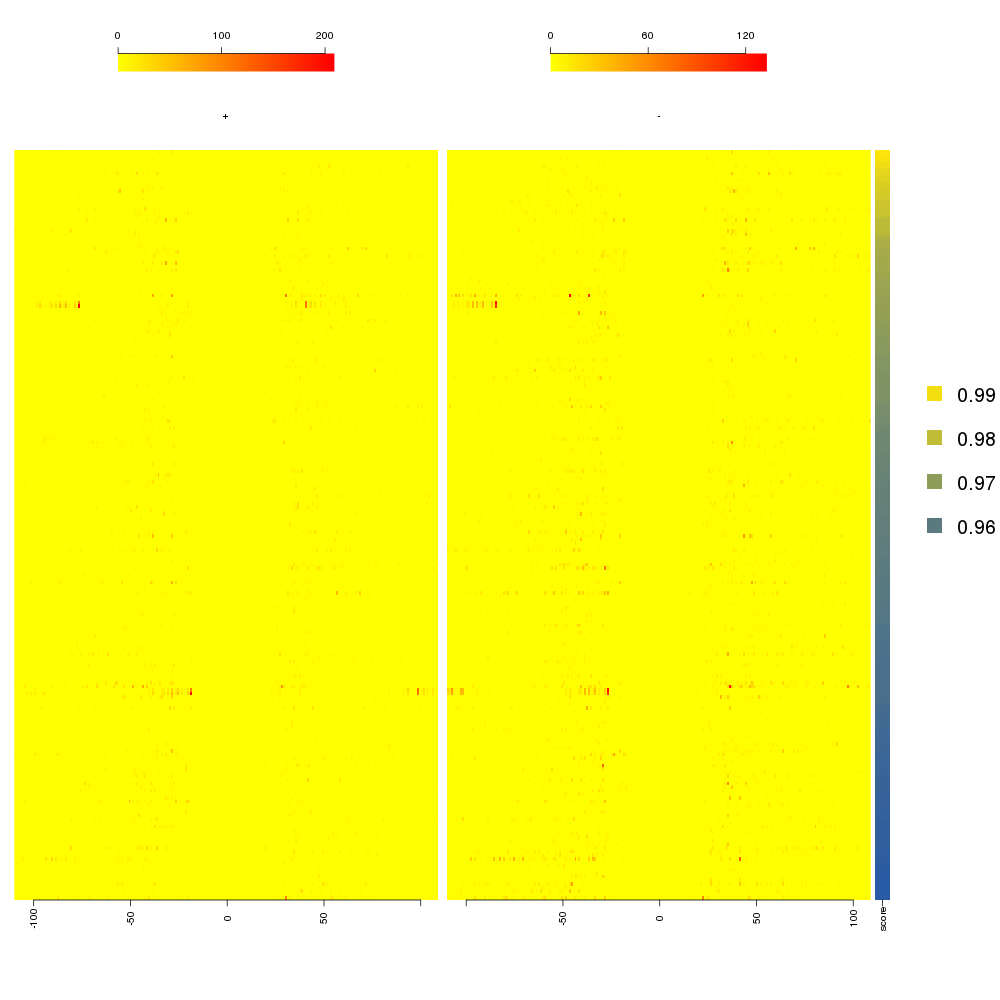

Supplement: S2 File — (ZIP) [file pone.0232332.s012.zip › nucleosome_positioning/SRX6443491_feature_aligned_heatmap.png]

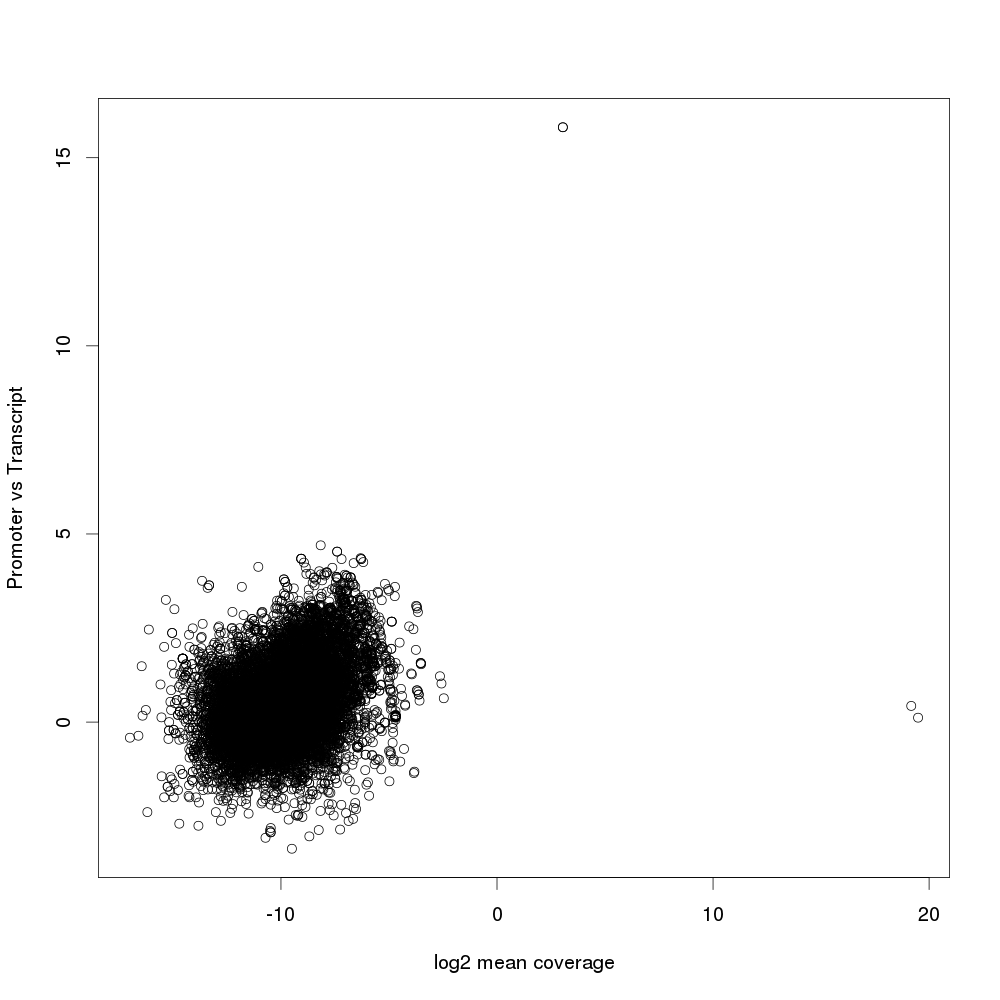

Supplement: S2 File — (ZIP) [file pone.0232332.s012.zip › nucleosome_positioning/SRR3622817_pt_score.png]

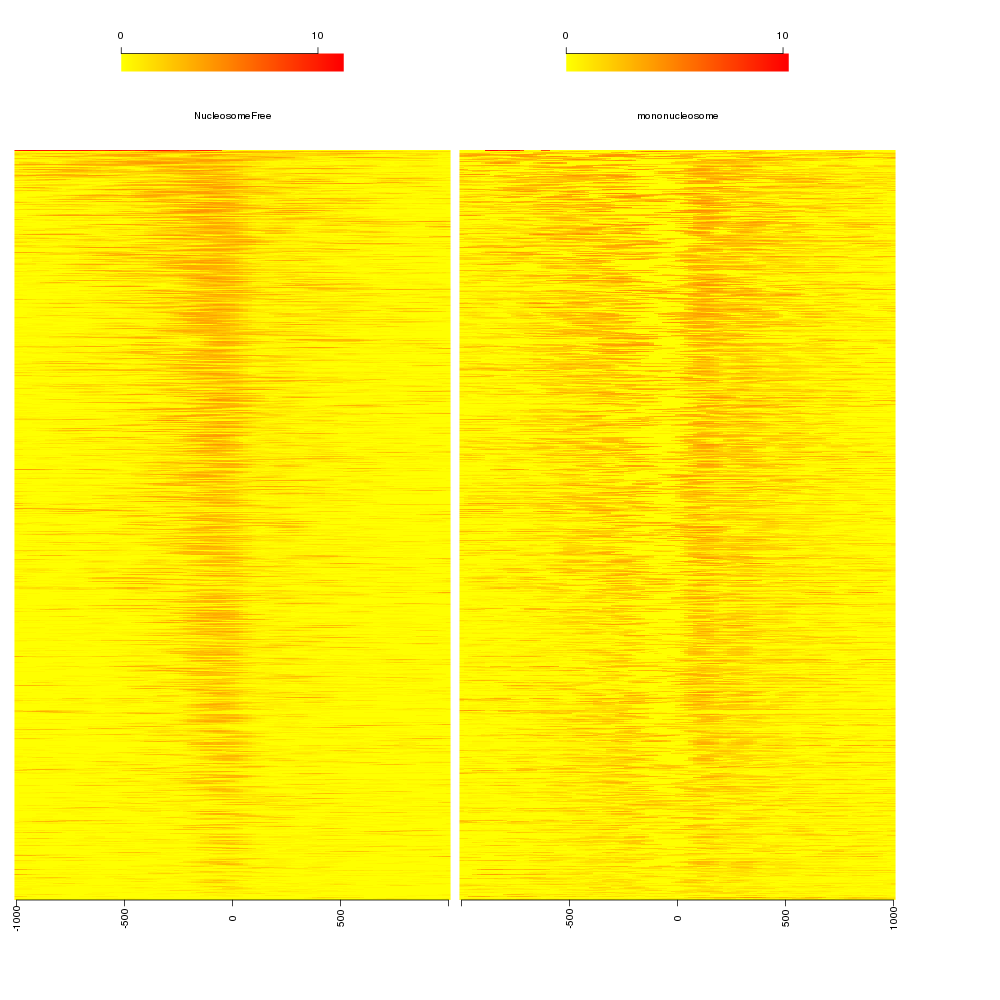

Supplement: S2 File — (ZIP) [file pone.0232332.s012.zip › nucleosome_positioning/SRR5063984_nucleosome_heatmap.png]

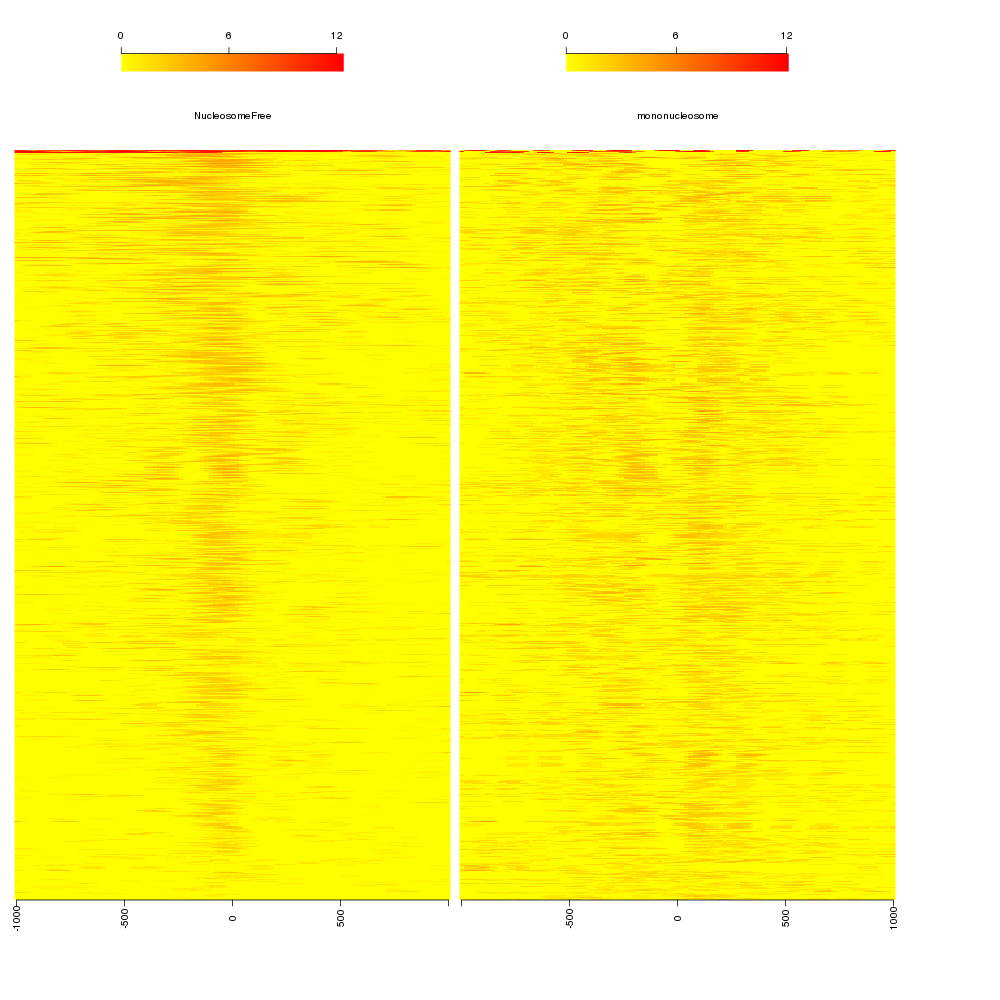

Supplement: S2 File — (ZIP) [file pone.0232332.s012.zip › nucleosome_positioning/SRR891275_nucleosome_heatmap.png]

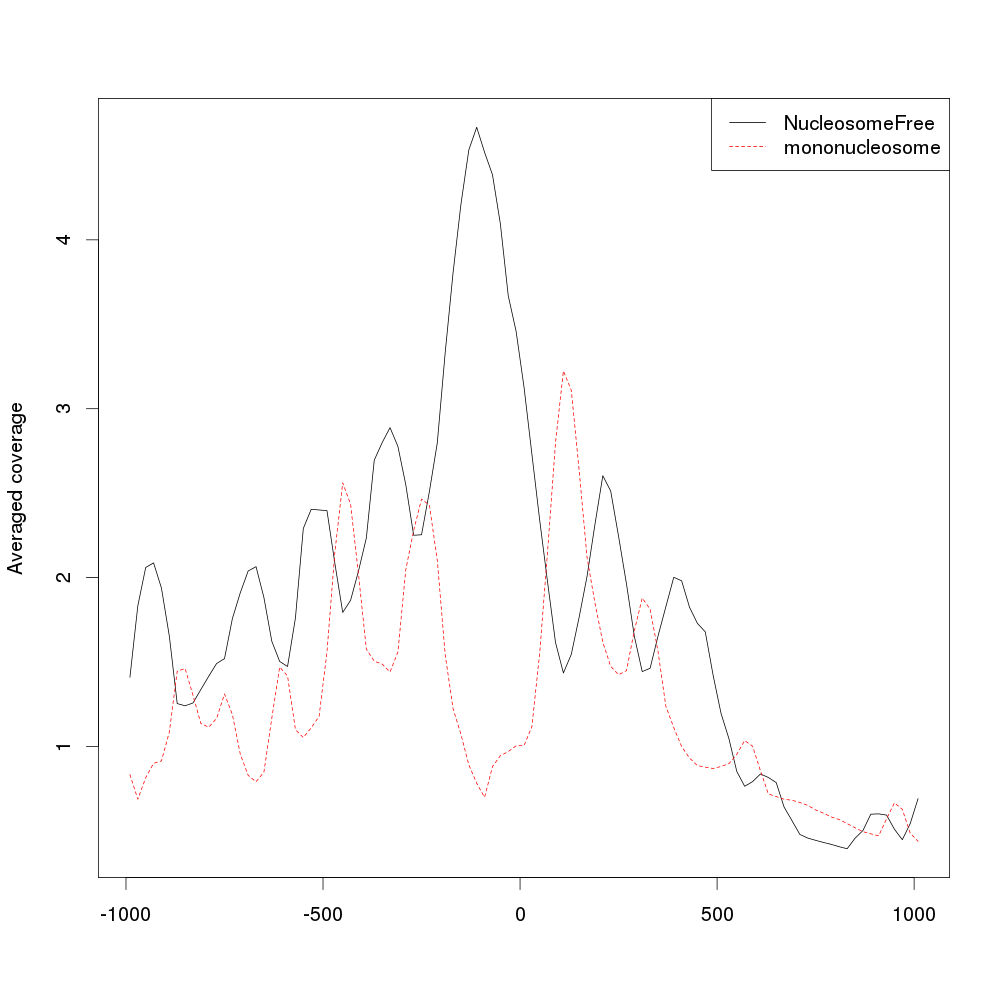

Supplement: S2 File — (ZIP) [file pone.0232332.s012.zip › nucleosome_positioning/SRR5007258_nucleosome_distribution.png]

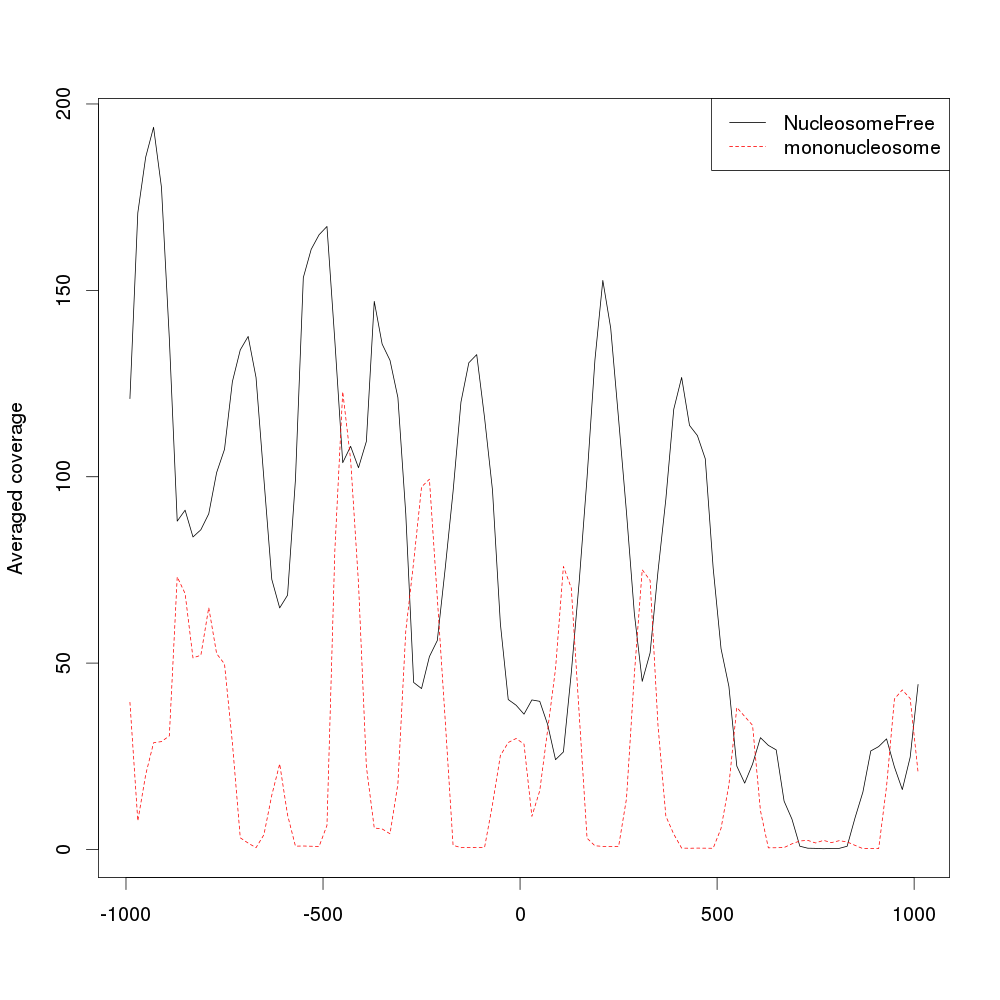

Supplement: S2 File — (ZIP) [file pone.0232332.s012.zip › nucleosome_positioning/SRR5128074_nucleosome_distribution.png]
